# The protecting-group free selective 3′-functionalization of nucleosides†
†Electronic supplementary information (ESI) available. CCDC 1525736–1525737. For ESI and crystallographic data in CIF or other electronic format see DOI: 10.1039/c6sc05081f



**DOI:** 10.1039/c6sc05081f

**Published:** 2017-01-18

**Authors:** Jamie M. McCabe Dunn, Mikhail Reibarkh, Edward C. Sherer, Robert K. Orr, Rebecca T. Ruck, Bryon Simmons, Ana Bellomo

**Affiliations:** a Department of Process Research & Development , MRL , Merck & Co., Inc. , Rahway , NJ 07065 , USA . Email: Jamie.mccabe.dunn@merck.com ; Email: mikhail_reibarkh@merck.com; b Department of Modelling and Informatics , MRL , Merck & Co., Inc. , Rahway , NJ 07065 , USA

## Abstract

The direct and chemoselective 3′-phosphoramidation, phosphorylation and acylation of nucleosides are described. Upon the discovery of a novel 3′-phosphorylamidation of therapeutic nucleoside analogues with DBU, we explored the mechanism of this rare selectivity through a combination of NMR spectroscopy and computational studies. The NMR and computational findings allowed us to develop a predictive computational model that accurately assesses the potential for 3′-functionalization for a broad range of nucleosides and nucleoside mimetics. The synthetic utility of this model was exemplified by demonstration on a broad scope of nucleosides and electrophiles yielding targets that were previously only accessible *via* a protection/deprotection sequence or an enzymatic approach.

## Abstract 2

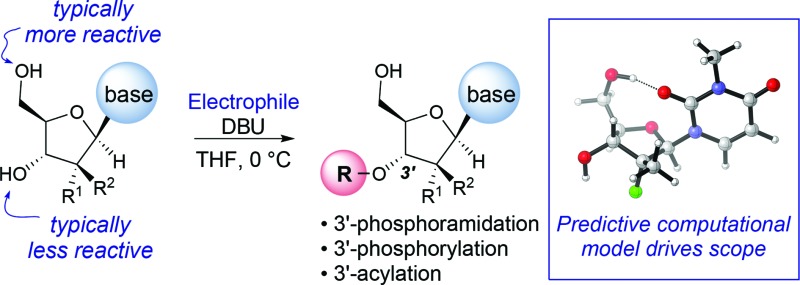
The direct and chemoselective 3′-phosphoramidation, phosphorylation and acylation of nucleosides are described.

## Introduction

**Fig. 1:**
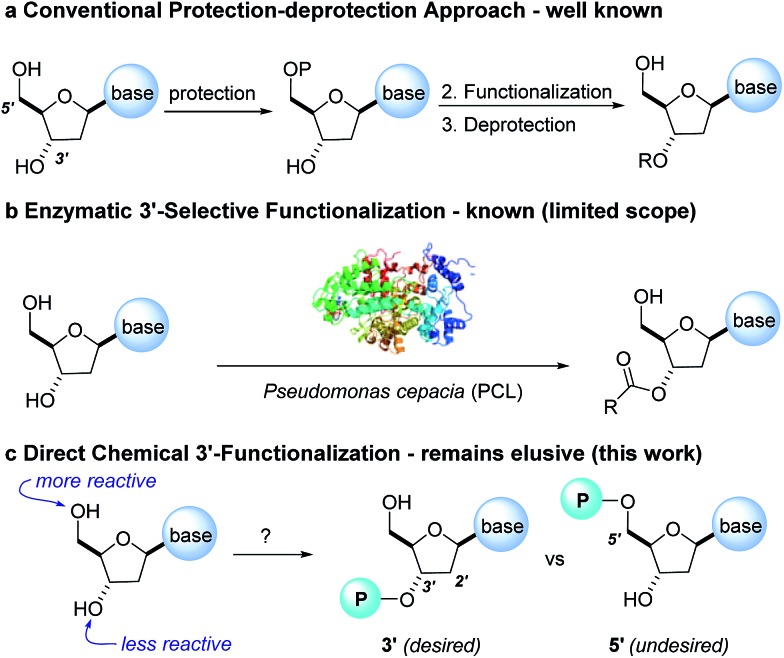
Current methods to obtain 3′ functionalized nucleosides.

The development of chemoselective, atom-economical reactions represents a persistent challenge in complex molecular synthesis.^1^ This task becomes even more difficult when one has to functionalize a less reactive group in the presence of a more reactive group. This is certainly the case for the selective functionalization of the secondary 3′-hydroxyl group of a nucleoside over the primary 5′-hydroxyl group. Owing to the growing importance of HCV, HIV and oncology prodrugs,^2^ there have been a number of publications that focus on direct, chemoselective reactions to provide 5′-ProTides.^3^ To the contrary, efficient syntheses of 3′-ProTides, have remained relatively unexplored (Fig. 1).^4^ These intermediates were a key feature of our design to synthesize cyclic prodrugs. This paucity of reports can be attributed to the challenge of achieving high chemoselectivity for functionalization of the 3′-position and concomitant need to utilize a tedious protection–deprotection sequence to obtain the desired product (Fig. 1a).^5^

Notably, enzymes are known to provide exquisite selectivity in functionalization reactions of biological molecules through a series of site-specific interactions between the protein and the substrate. For example, lipase from *Pseudomonas cepacia* (PCL) facilitates selective 3′-acylation of nucleosides. Hydrogen bonding interactions between the PCL and 5′-hydroxyl group of the nucleoside are believed to be responsible for inhibiting reactivity of the 5′-hydroxyl, while favouring reactivity at the 3′-hydroxyl (Fig. 1b).^6,7^ In this work, we report the non-enzymatic highly chemoselective 3′-functionalization of nucleosides. A combination of NMR spectroscopy and computational studies enabled development of a detailed mechanistic understanding of the selectivity. As a result we developed a predictive computational model that accurately assesses the potential for 3′-selectivity for a broad range of nucleosides and nucleoside mimetics.

## Results and discussion

Given literature precedent that strong organometallic bases provide undesired 5′-phosphorylation of nucleosides,^3^ we initiated a base screen that focused on the use of organic bases to mediate the preferred 3′-phosphorylation.‡
‡Ross and co-workers observed trace 3′-phosphorylation and phosphorous epimerization upon treatment of nucleoside PSI-6206 (**1a**) with DBU and DMAP. Treatment of a pharmaceutically relevant nucleoside, PSI-6206 (**1a**), and phosphoramide **4** in THF at 0 °C with a variety of organic bases led to a range of performances (Table 1). We observed no reactivity when using a relatively weak organic base, diisopropylethylamine (DIPEA), (entry 1). Switching to the more basic tetramethylguanidine (TMG), we observed promising selectivity (6 : 1) favouring 3′-phosphorylation albeit with epimerization of the phosphoramide P-stereocenter and a corresponding 40 : 60 diastereomeric mixture of 3′-phosphorylated products that likely occurred *via* a nucleophilic addition pathway prior to coupling (entry 2). Gratifyingly, when less nucleophilic bases, such as DBU and DBN, were employed, high selectivity to the desired 3′-phosphorylated product was observed with minimal epimerization at phosphorus (entries 3 and 4). Using an even stronger organic base, P_2_Et,^8^ led to reversion to 5′-phosphorylated selectivity as well as complete epimerization at phosphorus (entry 5).^9^ This reversal in selectivity is thought to arise from a mechanism similar to the one observed with strong organometallic bases.

**Table 1:** Base screen

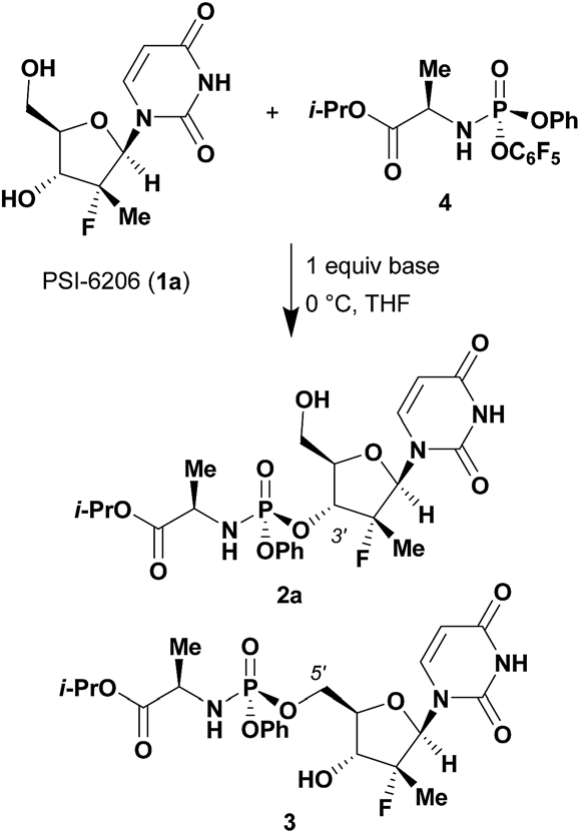				
Entry	Base	p*K*_a_ ^*a*^	Ratio (**2a** : **3**)	dr (**2a**/*p-epi*-**2a**)
1	DIPEA	18.8	NR	NA
2	TMG	23.3	6 : 1	40 : 60
3	DBU	24.3	70 : 1	95 : 5
4	DBN	23.9	36 : 1	95 : 5
5	P_2_Et	32.9	1 : 3.7	50 : 50

^*a*^p*K*_a_ measured in acetonitrile.^11^

Having identified DBU as the base that provided the ideal combination of excellent 3′-chemoselectivity and high phosphorus diastereoselectivity, we sought to find optimal reaction conditions to selectively phosphorylate nucleoside PSI-6206 (**1a**). We identified two key parameters for this reaction: solvent polarity and temperature. A moderately non-polar solvent, tetrahydrofuran (THF), was identified as the optimal solvent for chemo- and diastereoselectivity while polar aprotic solvents, such as NMP, led to diminution of the 3′-chemoselectivity. When salt additives such as MgBr_2_·Et_2_O or MgCl_2_ were used, minimal 3′-phosphorylation was observed while 5′-phosphorylation was the major product in a complex reaction mixture. Temperature also played a critical role in controlling the diastereoselectivity: as the temperature was increased, a corresponding decrease in diastereoselectivity was observed. Under optimized conditions, treatment of a mixture of PSI-6206 (**1a**) and phosphoramide **4** in THF at 0 °C with 1.0–1.05 equivalents of DBU provided the desired 3′-phosphorylated product **2a** in an impressive 92% isolated yield and 95 : 5 dr (Table 2, entry 1).^10^

**Fig. 2:**
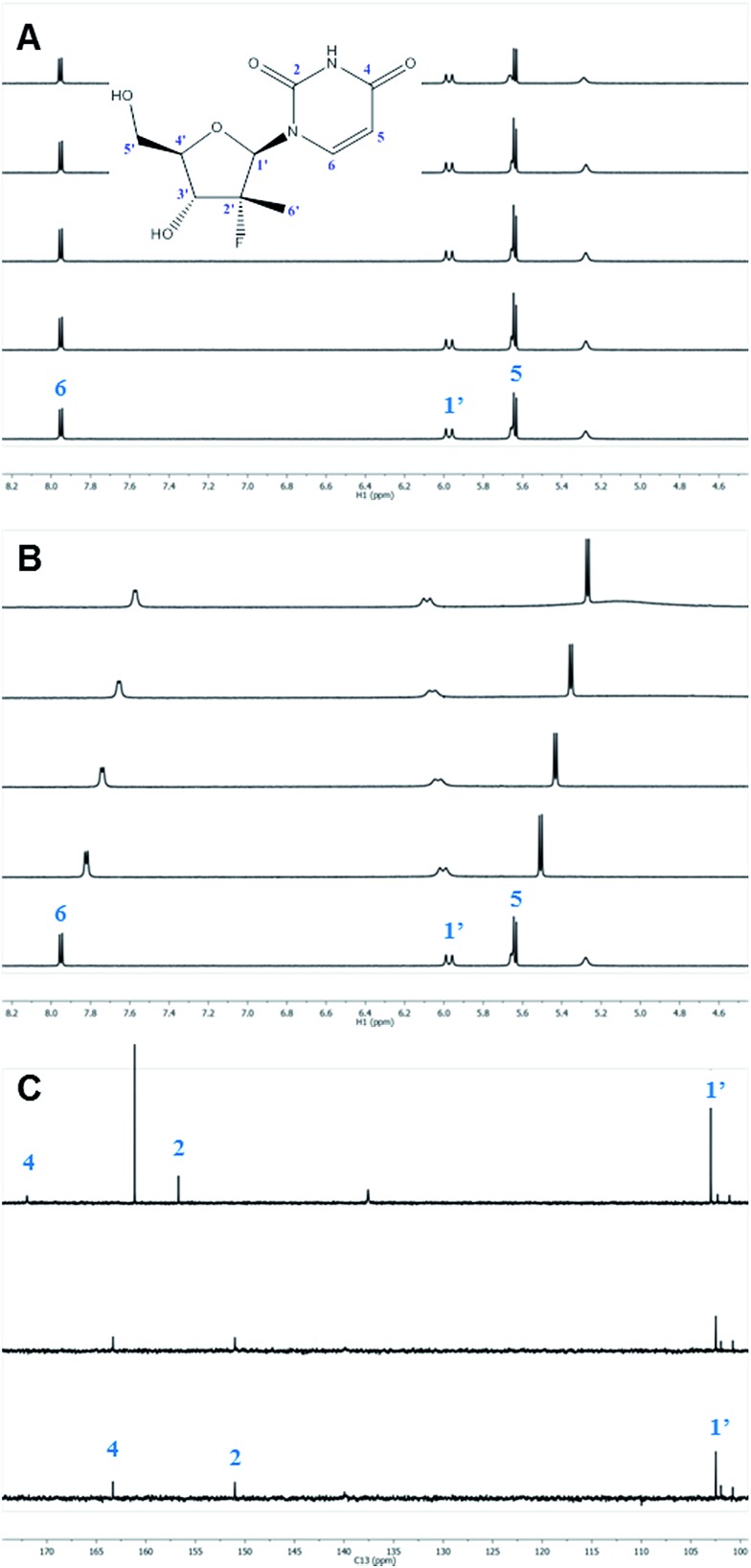
NMR titration of **1a** with DIPEA and DBU. (A) ^1^H NMR spectra of **1a** in the presence of 0, 0.5, 1, 2 and 5 equivalents of DIPEA. (B) ^1^H NMR spectra of **1a** in the presence of 0, 0.5, 1, 2 and 5 equivalents of DBU. (C) ^13^C NMR spectra of **1a** (bottom), **1a** with 5 equivalents of DIPEA (middle) and **1a** with 5 equivalents of DBU (top).

While excellent experimental results were achieved, we sought to understand the molecular mechanism of this unprecedented non-enzymatic 3′-chemoselectivity in order to better apply this discovery to other nucleosides. An extensive NMR study of the DBU–nucleoside **1a** binary system was initiated in order to determine what, if any, effect DBU had on the nucleoside. NMR titration experiments in which the ^1^H, ^13^C and ^19^F NMR chemical shifts of **1a** were monitored in the independent presence of increasing amounts of DBU, or DIPEA as negative control, provided clues as to the role of DBU. As expected from the base screening results, systematic titration of up to 5 equivalents of DIPEA into a solution of **1a** had no effect on its ^1^H and ^13^C NMR chemical shifts (Fig. 2A and C). In contrast, titrating the same molar amounts of DBU resulted in significant changes in the ^1^H and ^13^C NMR spectra of **1a** (Fig. 2B and C).^12^

**Fig. 3:**
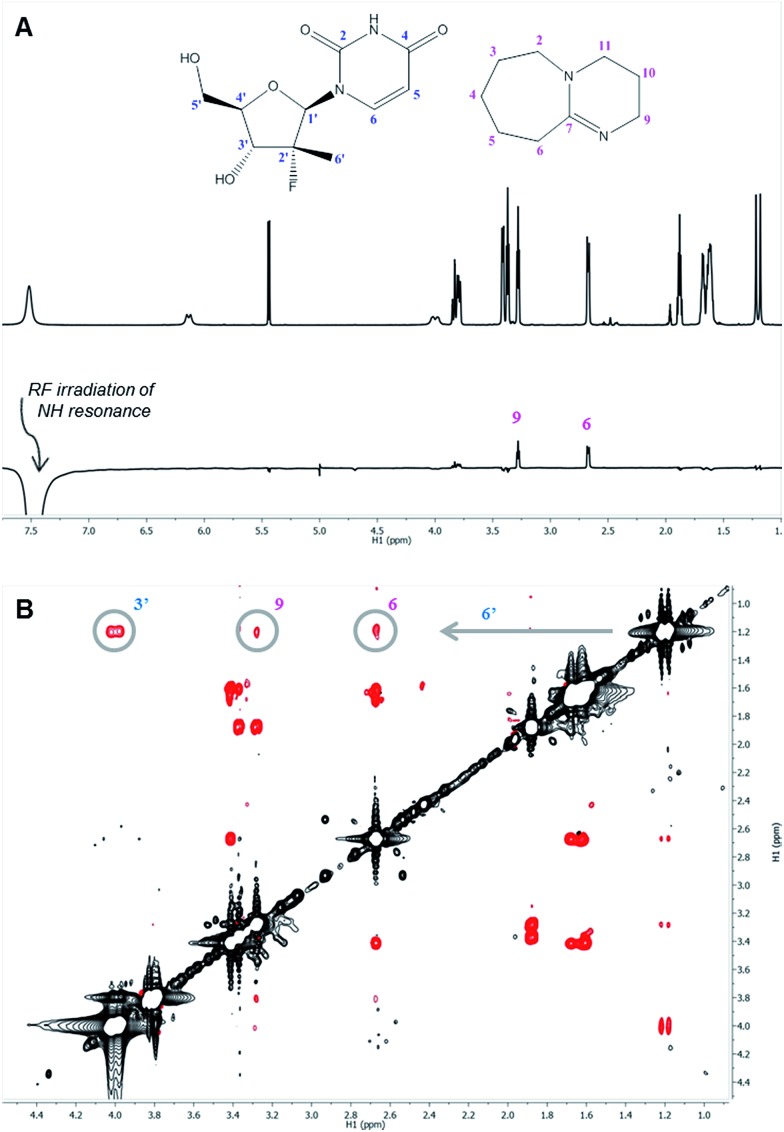
NOE studies of **1a** complex with DBU. (A) Superimposed ^1^H and 1D NOE spectra of 1 : 1 mixture of **1a** and DBU. Strong NOE to DBU methylenes 6 and 9 indicates protonation of N-8 of DBU. (B) 2D ^1^H–^1^H NOESY spectrum of 1 : 1 mixture of **1a** and DBU. Intermolecular NOEs between 6′ methyl and DBU methylenes 6 and 9, as well as intramolecular NOE between 6′ methyl and 3′ methine are shown.

The ^1^H nuclei most sensitive to the DBU titration were both uracil protons, with Δ*δ* of 0.40 ppm ^13^ each, while the 1′ and 4′ protons showed a significant, albeit smaller, effect with Δ*δ* of 0.12 ppm. The effect of DBU on ^13^C chemical shifts of **1a** was far more pronounced: C-4′ exhibited a Δ*δ* of 0.6 ppm, C-5 of uracil had Δ*δ* of 2.4 ppm, and carbonyls C-2 and C-4 of uracil had Δ*δ* of –5.7 ppm and –8.7 ppm, respectively. Since ^13^C chemical shifts are typically insensitive to the macro-environment, such strong changes suggested a specific interaction between DBU and the nucleoside **1a**. To further probe this hypothesis, we conducted 1D NOE and 2D NOESY experiments on the DBU/**1a** mixture. Selective irradiation of the NH resonance yielded strong NOEs to the 6- and 9-methylene groups of DBU (Fig. 3A), providing direct evidence that the uracil NH of **1a** is likely to be fully deprotonated by DBU. Furthermore, the 2D NOESY data (Fig. 3B) revealed unexpected intermolecular NOEs between the 2′-methyl of **1a** and the 6- and 9-methylenes of DBU.

Taken collectively, the NOE data not only demonstrated proton transfer from the NH uracil, but revealed a stable acid–base complex formed between the nucleoside **1a** and DBU.^14^ The observed effects of DBU and DIPEA on **1a** in solution correlated well with the previously observed reactivities and suggest that deprotonation of the NH uracil is essential for reactivity since weak bases like DIPEA, which are unable to deprotonate the uracil, failed to promote reaction conversion (Table 1).

Once DBU binding and formation of an acid–base complex with **1a** had been verified independently by NOE and diffusion data, further analysis of the DBU-induced ^13^C chemical shift changes of **1a** was performed. While the largest changes (C-2, C-4 and C-6 of the uracil) were attributed to the deprotonation of the uracil NH, some additional effects were observed. In particular, significant Δ*δ* of the 4′ carbon, which is not relevant to the uracil deprotonation, suggested that formation of a complex with DBU induces a conformational change of **1a**. Additionally, a very large difference of 3 ppm between Δ*δ* values of uracil carbonyls C-2 and C-4 was observed,^15^ suggesting that one of the carbonyls (likely O-2) was involved in an H-bonding interaction.

**Fig. 4:**
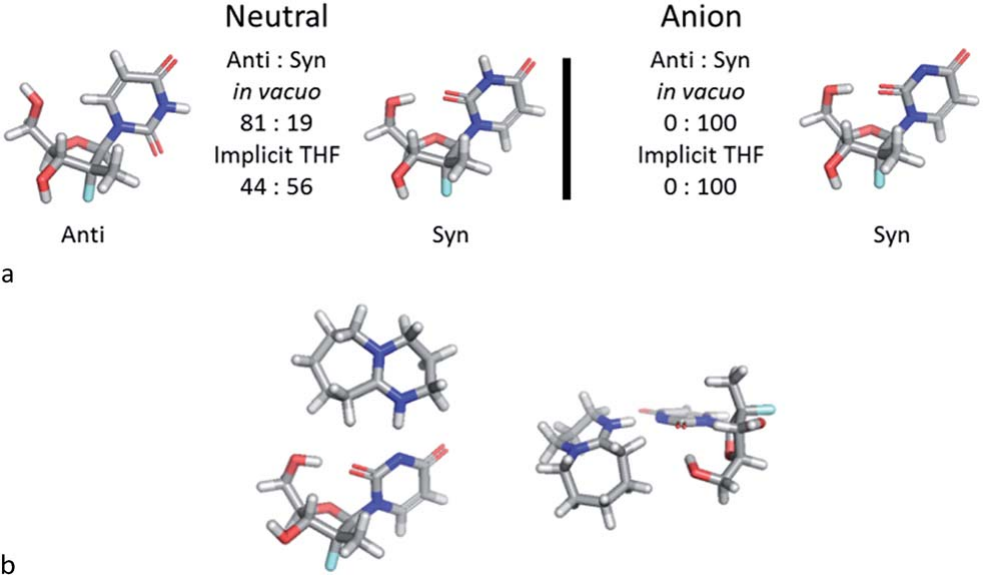
(a) Relative ratios for *syn* and *anti* conformations. (b) Two views of the lowest energy DBU complex.

The discovery of the acid–base complex between DBU and **1a**, as well as observation of a putative H-bonding interaction caused by DBU complexation, suggested that our observed nucleoside 3′-selectivity could arise *via* a similar pathway to the *Pseudomonas cepacia* lipase. To probe this hypothesis, we initiated computational studies aimed at augmenting the findings in the NMR studies. We developed a computational model to evaluate the solution state conformational distribution of nucleoside PSI-6206 (**1a**) using density functional theory (M06-2X/6-31+G** *in vacuo* or implicit THF). As expected, evaluating just the neutral form of nucleoside **1a** revealed no conformational preference that would drive selectivity to afford the desired 3′-phosphorylated product, since low energy conformations identified the nucleoside base existed in both *syn* and *anti* forms (Fig. 4a).^16^ To the contrary, analysis of the conformational space of the deprotonated uridine suggested a dramatic change in conformational preference under these conditions, altering the distribution between the two main *syn* and *anti* conformations from a ∼20/80 to 100/0 ratio *in vacuo*.

In the dominant conformation of the anion, an intramolecular hydrogen bond is formed between the 5′-hydroxyl group and the O-2 of the uracil base. This result is consistent with the NMR spectroscopic observations and leads to a folded structure for the nucleoside (Fig. 4a). Furthermore, the lowest energy conformation of the DBU acid–base complex with nucleoside **1a** places DBU on top of the nucleoside (Fig. 4b), consistent with experimentally observed intermolecular NOEs.

**Fig. 5:**
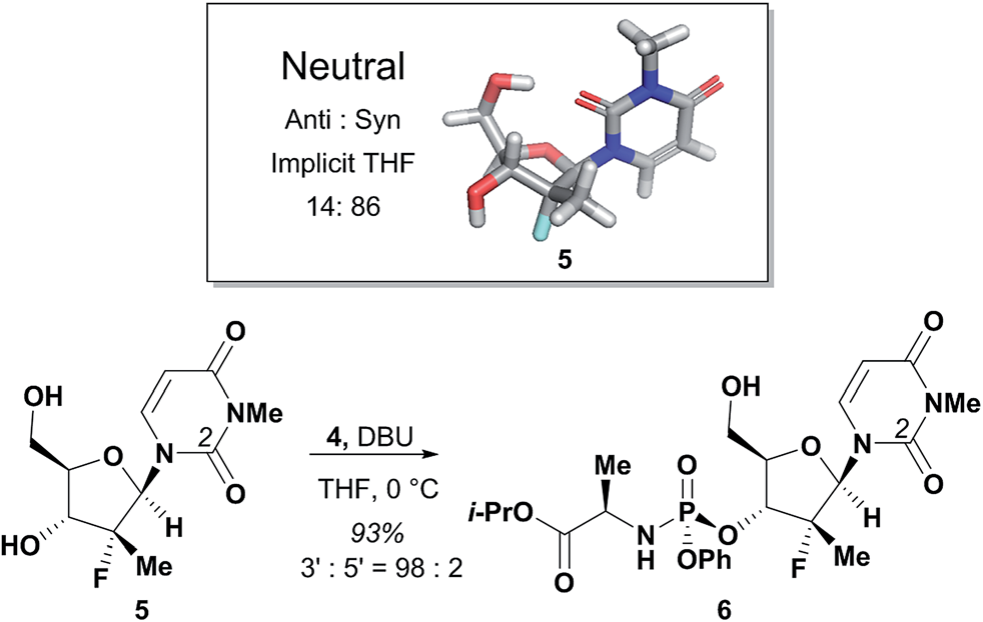
Phosphorylation of methylated NH uracil **5**.

The agreement between the computational and NMR studies suggested the existence of three possible factors contributing to the experimentally observed selectivity: (1) conformational preferences (hydrogen bond) that cause the nucleoside to fold in such a way to effectively block the approach to the 5′-hydroxyl; (2) complexation of the DBU with the nucleoside that essentially blocks the approach to the 5′-hydroxyl; (3) or a combination of both conformation and complexation. Experimental data obtained during optimization supported that the hydrogen bond between the O-2 and 5′-hydroxyl is a contributing factor for the observed selectivity, since polar additives such as NMP or MgBr_2_ degraded 3′-selectivity.^17^ In an attempt to determine if the complexation of DBU played a role in the selectivity, we synthesized *N*-methyl-uridine **5**.^18^ Although it cannot be deprotonated by DBU in the same fashion as **1a**, a computational investigation of the conformational preference of *N*-methyl-uridine **5** revealed that the favoured conformation of the neutral state in implicit solvent maintained the intramolecular hydrogen bond to the O-5′. Exposure of nucleoside **5** and phosphoramide **4** to our optimized reaction conditions provided exclusively the 3′-phosphorylated material in 93% yield and excellent 3′-selectivity (98 : 2) (Fig. 5). These results established the conformational preference as the sole driving force for the observed selectivity. Given this conclusion, we reasoned that 3′-selective functionalization of any nucleoside could be achieved if the conformational distributions energetically favoured the H-bonded conformation.

Computational analysis of a variety of custom nucleosides was employed to evaluate their Boltzmann conformational distributions and predict their corresponding selectivities (Table 2). For all 2′-doubly modified uridine nucleosides, the deprotonated distributions were 100% *syn* with intramolecular hydrogen bonds to the O-5′ hydroxyl and O-2 on the uridine.^19^ As predicted, changing from F to Cl still afforded the product with the desired 3′-selectivity in 89% yield (Table 2, entry 1 & 2). Furthermore, 2′-substitution with –CCH, –CN or –N_3_ also provided good yields of the 3′-phosphorylated products (Table 2, entries 3–5).

**Table 2:** Scope of the 3′-functionalization of nucleosides

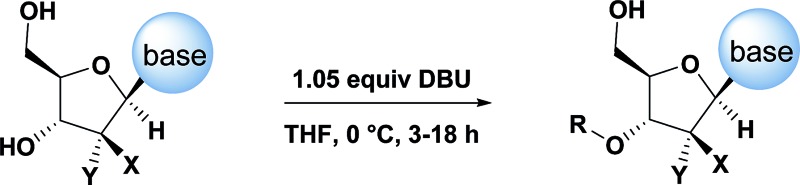					
Entry	Nucleoside	Electrophile	Product	Yield^*e*^	3′ : 5′^*g*^
1^*a*^	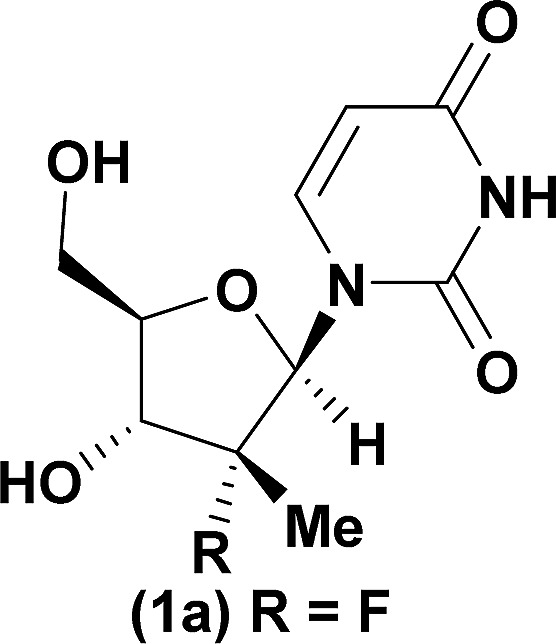	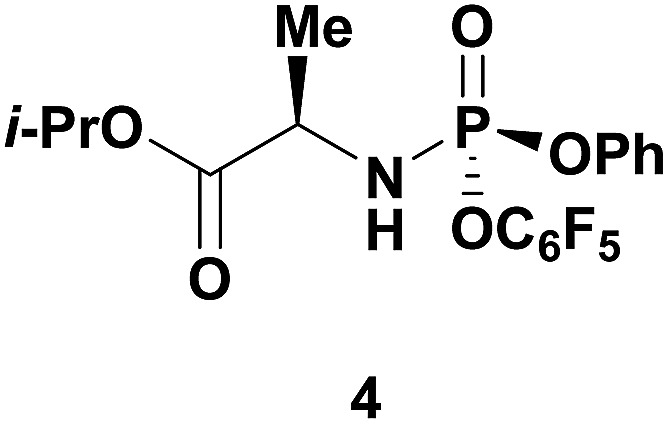	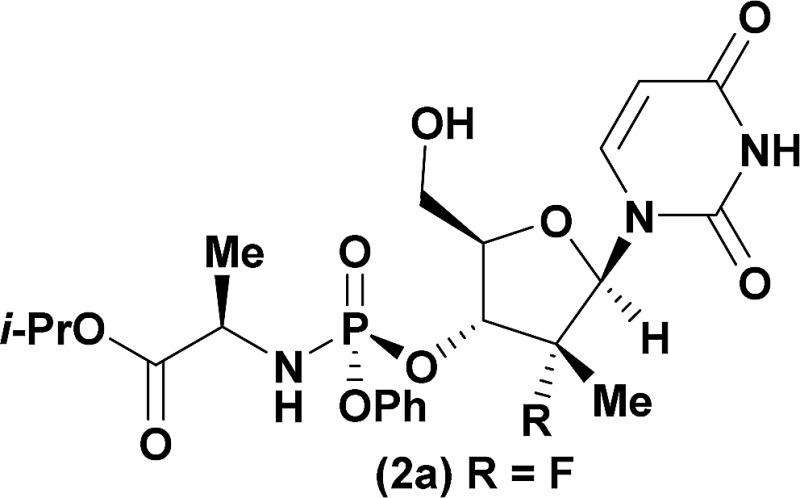	92	98 : 2
2^*a*^	(**1b**) R = Cl	**4**	(**2b**) R = Cl	89	98 : 2
3^*a*^	(**1c**) R = –CCH	**4**	(**2c**) R = –CCH	74	92 : 8
4^*a*^	(**1d**) R = N_3_	**4**	(**2d**) R = N_3_	62	91 : 9
5^*a*^	(**1e**) R = CN	**4**	(**2e**) R = CN	58	97 : 3
6	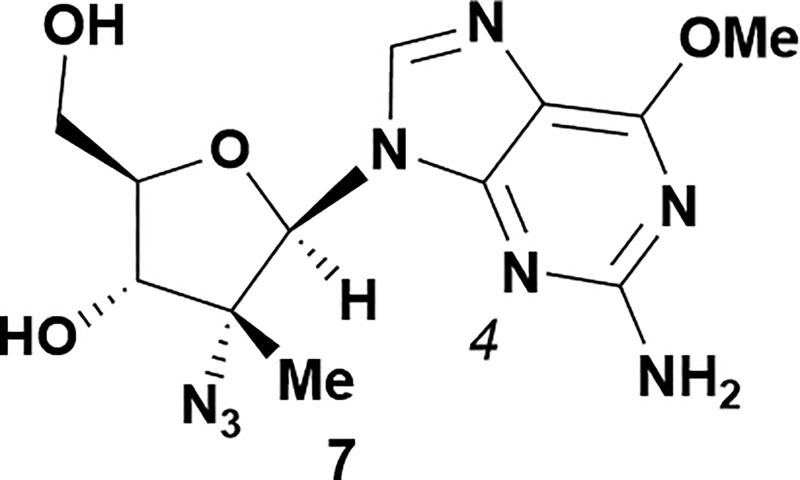	**4**	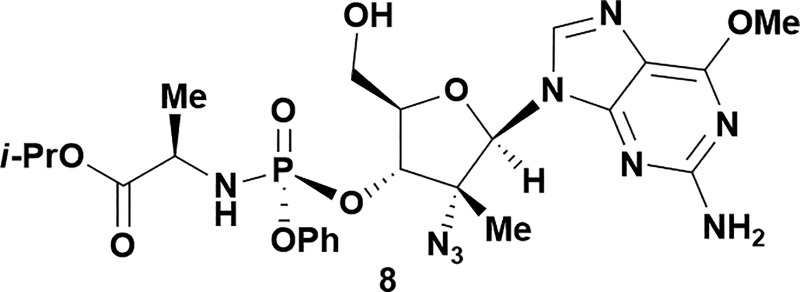	84	ND
7	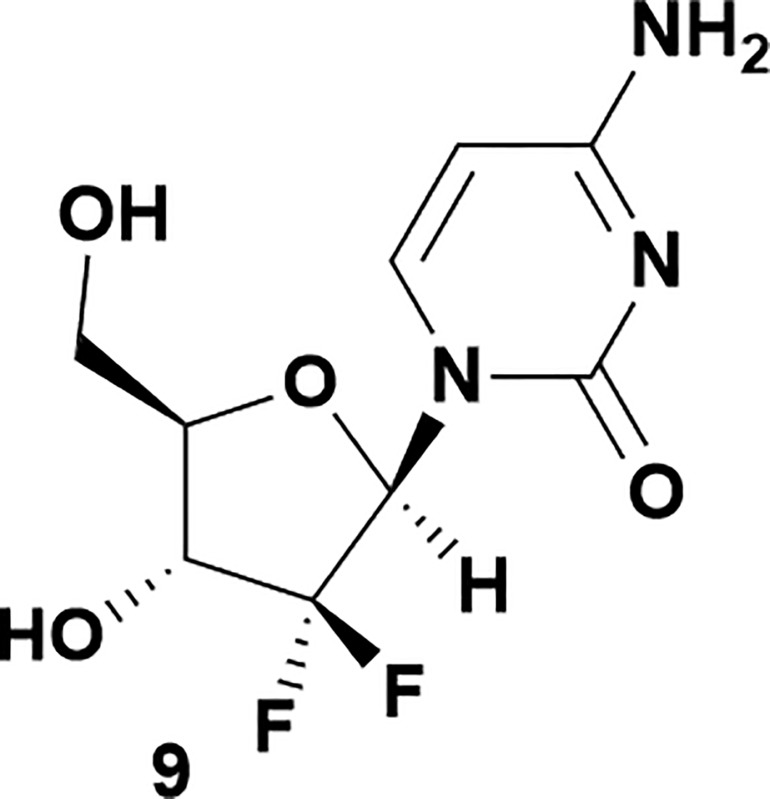	**4**	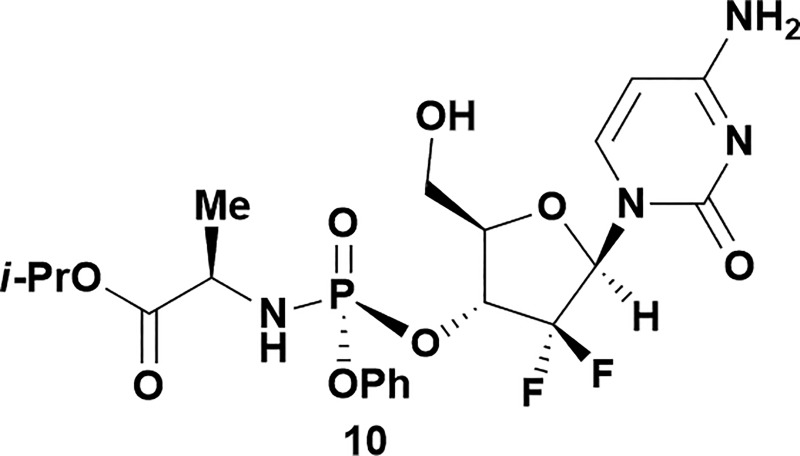	85^*f*^	ND
8^*a*^ ^,^ ^*b*^	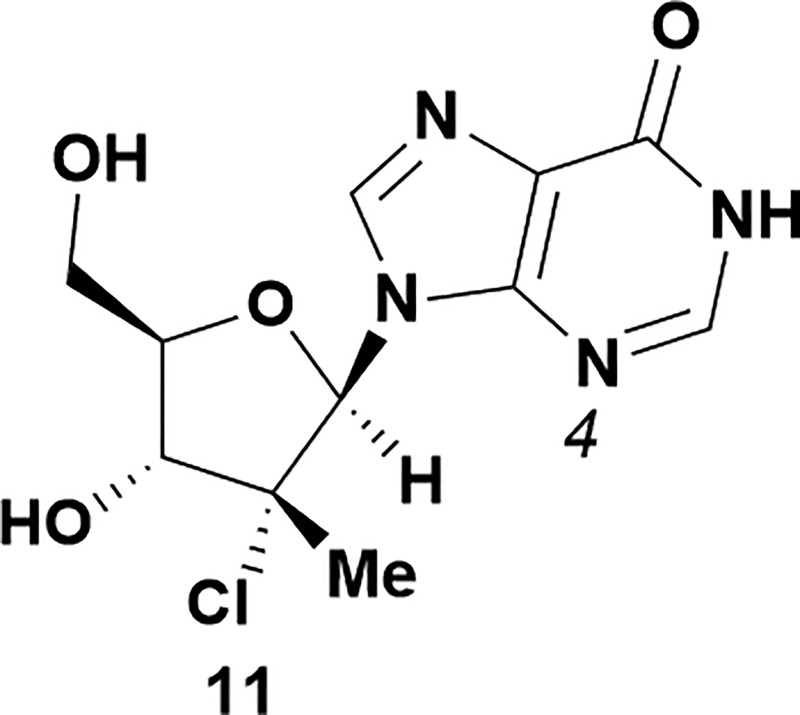	**4**	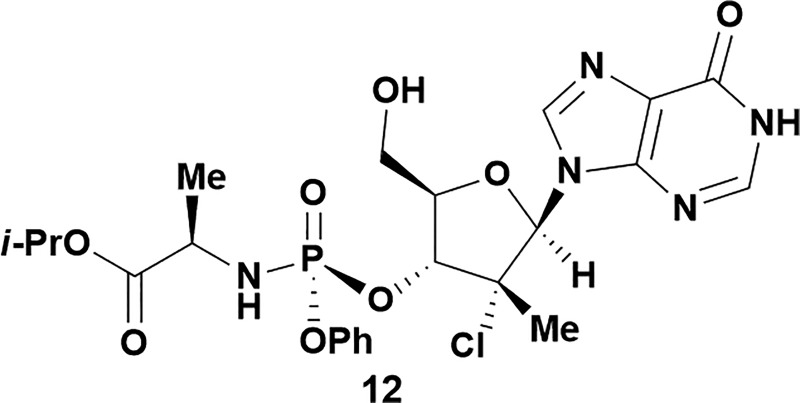	57	93 : 7
9	**5**	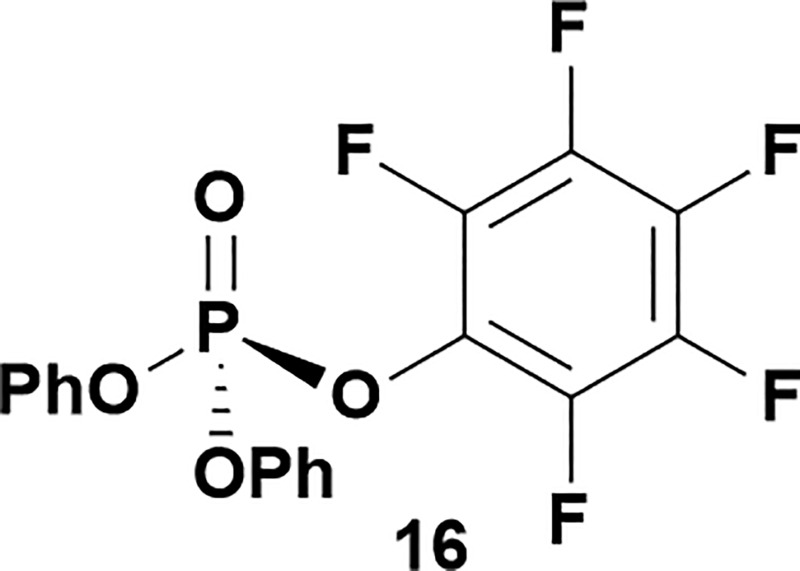	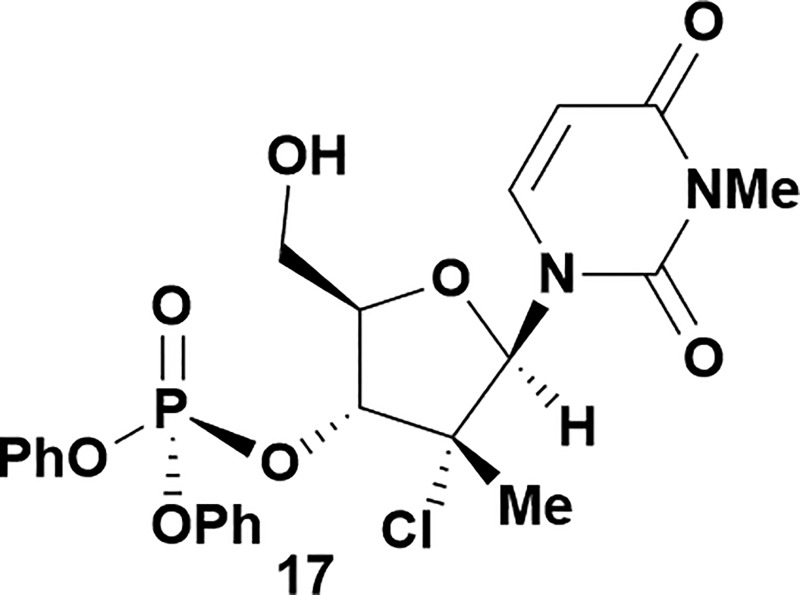	71	96 : 4
10^*c*^	**5**	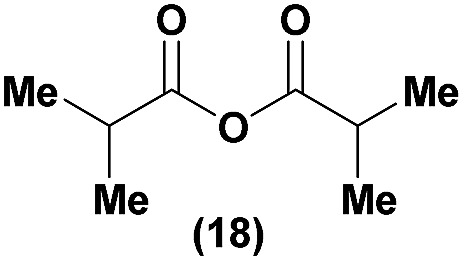	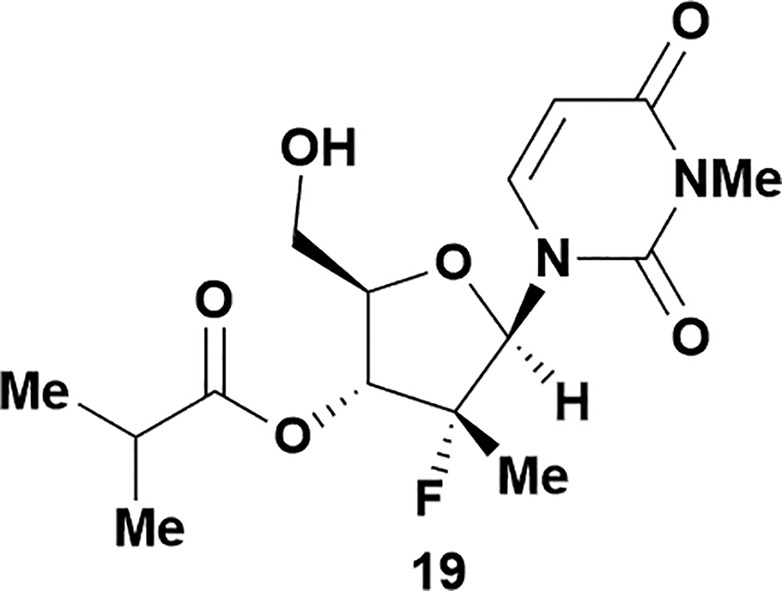	59 (68 brsm)^*d*^	99 : 1

^*a*^Anions are expected to be formed and serve as the structures modelled in the conformational analysis.

^*b*^2 equivalents of DBU used and temperature lowered to –15 °C.

^*c*^Temperature lowered to –15 °C.

^*d*^Based on recovered starting material (brsm).

^*e*^Isolated yields of pure 3′-phosphorated product and major *p*-epimer.

^*f*^Assay yield.

^*g*^3′ : 5′ selectivity determined by HPLC or UPLC, if labelled ND we were unable to resolve or detect the 5′-product the peaks by LC.

Analysis of non-uridine nucleosides revealed nucleoside-dependent H-bonding interactions that could direct 3′- or 5′-selectivity in that guanosine **7**, cytidine **9** and inosine **11** all displayed a similar conformational preference to uridine **5**: the 5′-hydroxyl group forms a hydrogen bond with the respective base.^20^ Furthermore, information gathered from the computational studies completely correlated with the experimental results. As predicted, the hydrogen bonding interaction between the N-4 of the guanosine and the 5′-hydroxyl group of guanosine **7** directs selective 3′-phosphorylation to give an 84% yield of the desired ProTide **8** (Table 2, entry 6). Likewise, the hydrogen bond conformation observed with the pyrimidin-1-one of cytidine **9** and N-4 purine of inosine **11** directed selective 3′-phosphorylation to give cytidine **10** and inosine **12** in 85% and 57% yields, respectively (Table 2, entries 7 and 8). It is remarkable that nucleosides that possess such diversity at the base are able to exhibit such exquisite selectivity for 3′-functionalization, providing a much broader substrate scope that would typically be expected through enzyme mediated reactions.

Conversely, analysis of α-thymidine (**13**) identified a 3′-hydroxyl group hydrogen bond with the thymidine base, leading to a prediction of a preference for 5′-phosphorylation. Consistent with our prediction, exposure of α-thymidine (**13**) to the same reaction conditions afforded the 5′-phosphorylation product **14** selectively in 53% yield (eqn (1)). Lastly, any nucleoside that possessed a 2′-hydroxyl group, such as cytidine (**15**) featured a preferred H-bonding interaction between the 2′-hydroxyl and the cytidine base. We envisioned that these 2′-hydroxyl nucleosides were unlikely to afford the desired 3′-selectivity in the phosphorylation; as expected, attempts to phosphorylate cytidine (**15**) yielded a complex mixture of phosphorylated products (eqn (2)).1
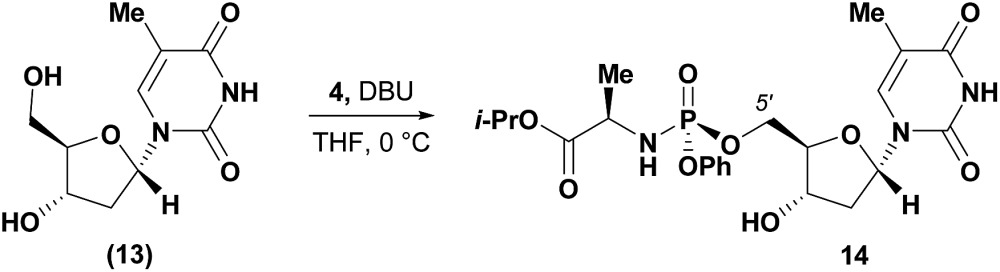

2
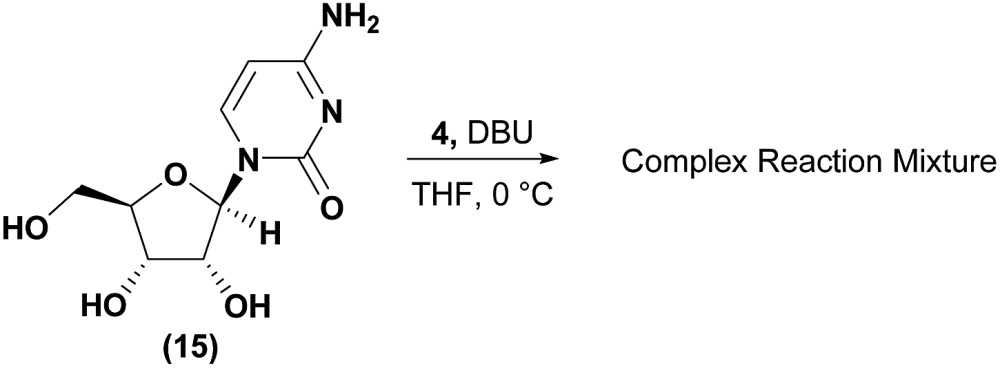



Having established confidence in the predictability of the 3′-selectivity of the phosphorylation, we hypothesized that this selectivity should be independent of the partner electrophile and serve as a general strategy for selective 3′-functionalization of nucleosides. Changing from the phosphoramide **4** to phosphonate **16** had no effect on the selectivity: treatment of nucleoside **5** with phosphate **16** and DBU afforded a 71% yield of the desired 3′-phosphorylated product **17** (Table 2, entry 9). An even more exciting result was achieved by moving away from phosphonates completely. Reacting nucleoside **5** with isobutyric anhydride (**18**) in the presence of DBU afforded 59% yield (68% brsm^21^) of 3′-isobutyl ester **19** (Table 2, entry 10). This result demonstrates that selectivity of the nucleoside 3′-functionalization is driven exclusively by the ability of the 5′-hydroxyl to form an intramolecular H-bond with the nucleic base and is independent of the partner electrophile, and provides a novel, simple and general approach to the acylation of nucleosides that is complementary to the enzymatic approach.^22^

## Conclusions

In conclusion, we have developed a novel direct 3′-phosphorylamidation of a series of nucleosides in the presence of DBU with selectivities that complement those observed in enzyme-catalysed reactions. Extensive NMR spectroscopy and computational studies provided mechanistic insight into the origin of this selectivity *via* an intramolecular hydrogen bond between the 5′-hydroxyl and the nucleoside base in a structure consistent with that known for a 3′-selective lipase. This determination led to the development of a simple predictive computational model based on conformational analysis for 3′-functionalization of nucleoside. This important finding not only accurately predicted observed selectivity of a diverse collection of nucleosides, but also enabled the extension of the scope to phosphate and acetate electrophiles. The broad implication of these findings on selective functionalization of nucleosides in the absence of protection–deprotection sequences is expected to find much use in the synthesis of these important therapeutics.
