# Supplementary material for: The protecting-group free selective 3′-functionalization of nucleosides
Source: Chem Sci. 2017 Jan 18;8(4):2804–10. doi: 10.1039/c6sc05081f (PMC5426439; doi:10.1039/c6sc05081f)
Supplement: Supplementary file 1 [file SC-008-C6SC05081F-s001.pdf]

Electronic Supplementary Material (ESI) for Chemical Science.
This journal is © The Royal Society of Chemistry 2017

The Protecting-Group Free Selective 3'-Functionalization of Nucleosides

Jamie M. McCabe Dunn*[a], Mikhail Reibarkh*[a], Edward C. Sherer[b], Robert K. Orr[a], Rebecca T. Ruck[a], Bryon Simmons[a], Ana Bellomo[a]

a. Department of Process Research & Development, MRL, Merck & Co., Inc., Rahway, NJ 07065, USA

b. Department of Modelling and Informatics, MRL, Merck & Co., Inc., Rahway, NJ 07065, USA

General ……………………………………………………………………………………S1

General Procedure A: Preparation of 3'-Phosphorylated Nucleosides ……………………S1

Preparation of 3'-acetylated Nucleosides……………………………………………………S8

NMR Studies ……………………………………………………………………………………S9

NMR Sprectra for new compounds……………………………………………………………S12

X-ray Data for Compound **4** and **2a**…………………………………………………………S49

Computational Data……………………………………………………………………………S53

General. Commercial grade reagents and solvents were used without further purification. ^{1}H NMR and ^{13}C NMR spectra were measured with a 400 MHz spectrometer or 500 MHz spectrometer, as specified. High resolution mass spectra (HRMS) were obtained on Accurate-Mass Time-of-Flight (TOF) mass spectrometer and reported as m/z (relative intensity). Accurate masses are reported for the molecular ion (M+1). Purifications were carried out by flash column chromatography on a Teledyne Isco CombiFlash R_f using a gradient elution of 0-5% MeOH/DCM unless otherwise specified.

General Procedure A: Preparation of 3'-Phosphorylated Nucleosides.

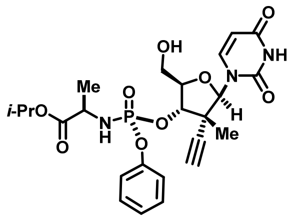

2c

Preparation of isopropyl ((*R*)-(((*2R*,3S,4R,5R)-5-(2,4-dioxo-3,4-dihydropyrimidin-1(2H)-yl)-4-ethynyl-2-(hydroxymethyl)-4-methyltetrahydrofuran-3-yl)oxy)(phenoxy)phosphoryl)-D-alaninate (2c). To a solution of (*R*)-isopropyl 2-(((*R*)-

(perfluorophenoxy)(phenoxy)phosphoryl)amino)propanoate (**4**) (0.43 g, 0.94 mmol) and 1-((*2R,3R,4S,5R*)-3-ethynyl-4-hydroxy-5-(hydroxymethyl)-3-methyltetrahydrofuran-2-yl)pyrimidine-2,4(1H,3H)-dione (**1c**) (0.25 g, 0.94 mmol) in THF (9 ml) at 0 °C was added DBU (0.15 ml, 0.99 mmol). The reaction was stirred at 0 °C for 3 h and then quenched with addition of 0.5 M citric acid. The bi-phasic mixture was then diluted with EtOAc and the organic layer was separated and the aqueous layer was back extracted once with EtOAc. The combined organic layers were washed with 10% brine, dried over $MgSO_4$, filtered and concentrated under reduced pressure. The residue was purified by silica gel chromatography to give 0.37 g (74%) of isopropyl ((*R*)-(((*2R,3S,4R,5R*)-5-(2,4-dioxo-3,4-dihydropyrimidin-1(2H)-yl)-4-ethynyl-2-(hydroxymethyl)-4-methyltetrahydrofuran-3-yl)oxy)(phenoxy)phosphoryl)-D-alaninate (**2c**): ^{1}H NMR (500 MHz, d_6-*DMSO*): δ 1.18 (dd, J = 6.2, 3.9 Hz, 6 H), 1.22 (s, 3 H), 1.27 (d, J = 7.1 Hz, 3 H), 3.46 (s, 1 H), 3.69-3.65 (m, 1 H), 3.90-3.81 (m, 2 H), 4.03 (d, J = 8.5 Hz, 1 H), 4.60 (t, J = 8.8 Hz, 1 H), 4.90-4.85 (m, 1 H), 5.36 (t, J = 4.5 Hz, 1 H), 5.69 (d, J = 8.1 Hz, 1 H), 6.21-6.14 (m, 2 H), 7.25-7.18 (m, 3 H), 7.39 (t, J = 7.8 Hz, 2 H), 8.10 (d, J = 8.1 Hz, 1 H), 11.49 (s, 1 H). ^{13}C NMR (126 MHz, d_6-*DMSO*): δ 172.3 (d, J = 6.4 Hz), 162.8, 150.6 (d, J = 6.7 Hz), 150.5, 140.0, 129.6 (2C), 124.7, 120.2 (d, J = 4.8 Hz, 2C), 101.7, 89.4, 82.9, 81.9 (d, J = 4.9 Hz), 76.6, 76.2 (d, J = 5.1 Hz), 68.0, 57.9, 49.9, 46.6 (d, J = 4.0 Hz), 21.5, 21.4, 19.8 (d, J = 5.9 Hz), 19.0. ^{31}P NMR (203 MHz, DMSO-*d*$_6$) δ 3.45. MS = 536.33 (M+1). HRMS calcd. For $C_{24}H_{30}N_3O_9P$ ($[M + H]^+$) 536.1798, found 536.1806.

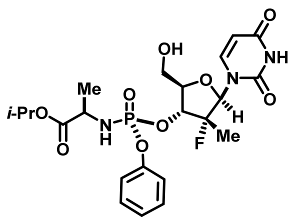

2a

Isopropyl ((*R*)-(((*2R,3R,4R,5R*)-5-(2,4-dioxo-3,4-dihydropyrimidin-1(2H)-yl)-4-fluoro-2-(hydroxymethyl)-4-methyltetrahydrofuran-3-yl)oxy)(phenoxy)phosphoryl)-D-alaninate (2a). According to the general procedure A, treatment of (*R*)-isopropyl 2-(((*R*)-(perfluorophenoxy)(phenoxy)phosphoryl)amino)propanoate **(4)** (0.44 g, 0.96 mmol) and 1-((*2R,3R,4R,5R*)-3-fluoro-4-hydroxy-5-(hydroxymethyl)-3-methyltetrahydrofuran-2-yl)pyrimidine-2,4(1H,3H)-dione **(1a)** (0.25 g, 0.96 mmol) with DBU (0.15 ml, 1.01 mmol) gave 0.47 g (92%) of isopropyl ((*R*)-(((*2R,3R,4R,5R*)-5-(2,4-dioxo-3,4-dihydropyrimidin-1(2H)-yl)-4-fluoro-2-(hydroxymethyl)-4-methyltetrahydrofuran-3-yl)oxy)(phenoxy)phosphoryl)-D-alaninate **(2a)**: ^{1}H NMR (500 MHz, *CDCl$_3$*): δ 1.25 (dd, J = 6.3, 2.7 Hz, 6 H), 1.44-1.40 (m, 6 H), 3.52 (d, J = 13.4 Hz, 1 H), 3.85 (d, J = 13.4 Hz, 1 H), 4.06-3.97 (m, 2 H), 4.48 (dd, J = 12.5, 9.8 Hz, 1 H), 4.71-4.63 (m, 1 H), 5.05-5.00 (m, 1 H), 5.74 (d, J = 8.2 Hz, 1 H), 6.17 (d, J = 18.0 Hz, 1 H), 7.22 (dd, J = 13.1, 7.5 Hz, 3 H), 7.35 (t, J = 7.8 Hz, 2 H), 7.97 (d, J = 8.2 Hz, 1 H), 9.99 (s, 1 H). ^{13}C NMR (126 MHz, *CDCl$_3$*): δ 172.9 (d, J = 8.3 Hz), 163.5, 150.7, 150.1 (d, J = 7.5 Hz), 139.7, 129.9 (2C), 125.8 (d, J = 1.4 Hz), 120.7 (d, J = 4.5 Hz, 2C), 103.1, 99.7 (dd, J = 186.9, 5.6 Hz), 88.7 (br d, J = 38.2 Hz), 80.7 (d, J = 3.1 Hz), 73.9 (dd, J = 15.1, 4.3 Hz), 69.8, 58.6, 50.5,

21.8, 21.7, 21.1 (d, J = 4.2 Hz), 16.6 (d, J = 25.3 Hz). ^{31}P NMR (203 MHz, Chloroform-*d*) δ 4.75. MS = 530.33 (M+1). HRMS calcd. For $C_{22}H_{29}FN_3O_9P$ ($[M + H]^+$) 530.1703, found 530.1708.

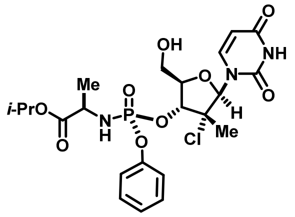

2b

Isopropyl ((*R*)-(((*2R*,3R,4R,5R)-4-chloro-5-(2,4-dioxo-3,4-dihydropyrimidin-1(2H)-yl)-2-(hydroxymethyl)-4-methyltetrahydrofuran-3-yl)oxy)(phenoxy)phosphoryl)-D-alaninate (2b). According to the general procedure A, treatment of (*R*)-isopropyl 2-(((*R*)-(perfluorophenoxy)(phenoxy)phosphoryl)amino)propanoate **(4)** (0.41 g, 0.90 mmol) and 1-((*2R,3R,4R,5R*)-3-chloro-4-hydroxy-5-(hydroxymethyl)-3-methyltetrahydrofuran-2-yl)pyrimidine-2,4(1H,3H)-dione **(1b)** (0.25 g, 0.90 mmol) with DBU (0.14 ml, 0.95 mmol) gave 0.44 g (89%) of isopropyl ((*R*)-(((*2R,3R,4R,5R*)-4-chloro-5-(2,4-dioxo-3,4-dihydropyrimidin-1(2H)-yl)-2-(hydroxymethyl)-4-methyltetrahydrofuran-3-yl)oxy)(phenoxy)phosphoryl)-D-alaninate **(2b)**: ^{1}H NMR (500 MHz, d$_6$-*DMSO*): δ 1.18 (dd, J = 6.3, 2.3 Hz, 6 H), 1.27 (d, J = 7.1 Hz, 3 H), 1.57 (s, 3 H), 3.66-3.62 (m, 1 H), 3.88-3.83 (m, 2 H), 4.08 (d, J = 9.0 Hz, 1 H), 4.78 (br s, 1 H), 4.91-4.86 (m, 1 H), 5.48 (s, 1 H), 5.72 (d, J = 8.2 Hz, 1 H), 6.35-6.29 (m, 2 H), 7.26-7.19 (m, 3 H), 7.40 (t, J = 7.8 Hz, 2 H), 8.18 (d, J = 8.3 Hz, 1 H), 11.55 (s, 1 H). ^{13}C NMR (126 MHz, d$_6$-*DMSO*): δ 172.3 (d, J = 5.9 Hz), 162.8, 150.6, 150.5 (d, J = 6.6 Hz), 139.3, 129.7 (2C), 124.8, 120.2 (d, J = 4.9 Hz, 2C), 102.2, 91.1, 81.1, 75.8, 74.7 (br s), 68.0, 57.6, 50.0, 22.4, 21.5, 21.4, 19.7 (d, J = 6.3 Hz). ^{31}P NMR (203 MHz, DMSO-*d$_6$*) δ 3.28. MS = 546.30 (M+1). HRMS calcd. For $C_{22}H_{29}ClN_3O_9P$ ($[M + H]^+$) 546.1408, found 546.1414.

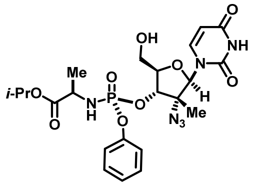

2d

Isopropyl ((*R*)-(((*2R,3S,4R,5R*)-4-azido-5-(2,4-dioxo-3,4-dihydropyrimidin-1(2H)-yl)-2-(hydroxymethyl)-4-methyltetrahydrofuran-3-yl)oxy)(phenoxy)phosphoryl)-D-alaninate (2d). According to the general procedure A, treatment of (*R*)-isopropyl 2-(((*R*)-(perfluorophenoxy)(phenoxy)phosphoryl)amino)propanoate **(4)** (0.40 g, 0.88 mmol) and 1-((*2R,3R,4S,5R*)-3-azido-4-hydroxy-5-(hydroxymethyl)-3-methyltetrahydrofuran-2-yl)pyrimidine-2,4(1H,3H)-dione **(1d)** (0.25 g, 0.88 mmol) with DBU (0.14 ml, 0.93 mmol) gave 0.30 g (62%) of isopropyl ((*R*)-(((*2R,3S,4R,5R*)-4-azido-5-(2,4-dioxo-3,4-dihydropyrimidin-1(2H)-yl)-2-(hydroxymethyl)-4-methyltetrahydrofuran-3-yl)oxy)(phenoxy)phosphoryl)-D-alaninate **(2d)**: ^{1}H NMR (400 MHz, *CDCl$_3$*) δ 9.72 (s, 1H), 8.03 (d, *J* = 8.1 Hz, 1H), 7.47 – 7.30 (m, 2H), 7.27 – 7.20 (m, 3H), 5.89 (s, 1H), 5.75 (d, *J* = 8.0 Hz, 1H), 5.04 (hept, *J* = 6.3 Hz, 1H), 4.70 (t, *J* = 9.1 Hz, 1H), 4.43 (dd, *J* = 12.6, 9.8 Hz, 1H), 4.10 – 3.96 (m, 1H), 3.90 (d, *J* = 8.9 Hz, 1H), 3.81 (d, *J* = 13.5 Hz, 1H), 3.58 – 3.49 (m, 1H), 1.45 – 1.39 (m, 6H), 1.26 (dd, *J* = 6.2, 4.1 Hz, 6H). ^{13}C NMR (101 MHz, *CDCl$_3$*) δ 172.7 (d, *J* = 8.3 Hz), 163.3, 150.6,

150.0 (d, *J* = 7.4 Hz), 139.6, 129.9 (2C), 125.9, 120.6 (d, *J* = 4.2 Hz, 2C), 102.9, 89.0, 81.3, 76.4 (d, *J* = 4.4 Hz), 69.8, 69.7 (d, J = 6.3), 58.6, 50.5, 21.6, 21.6, 21.1 (d, *J* = 3.7 Hz), 15.8. ^{31}P NMR (203 MHz, DMSO-d_6) δ 3.55. MS = 553.35 (M+1). HRMS calcd. For $C_{22}H_{29}N_6O_9P$ ($[M + H]^+$) 553.1812, found 553.1819.

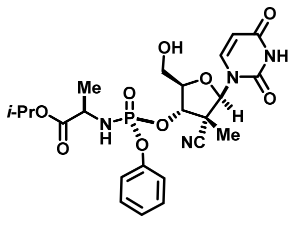

2e

Isopropyl ((*R*)-(((*2R,3S,4R,5R*)-4-cyano-5-(2,4-dioxo-3,4-dihydropyrimidin-1(2H)-yl)-2-(hydroxymethyl)-4-methyltetrahydrofuran-3-yl)oxy)(phenoxy)phosphoryl)-D-alaninate (2e). According to the general procedure A, treatment of (*R*)-isopropyl 2-(((*R*)-(perfluorophenoxy)(phenoxy)phosphoryl)amino) propanoate **(4)** (0.42 g, 0.94 mmol) and (*2R,3R,4S,5R*)-2-(2,4-dioxo-3,4-dihydropyrimidin-1(2H)-yl)-4-hydroxy-5-(hydroxymethyl)-3-methyltetrahydrofuran-3-carbonitrile **(1c)** (0.25 g, 0.94 mmol) with DBU (0.15 ml, 0.98 mmol) gave 0.29 g (58%) of Isopropyl ((*R*)-(((*2R,3S,4R,5R*)-4-cyano-5-(2,4-dioxo-3,4-dihydropyrimidin-1(2H)-yl)-2-(hydroxymethyl)-4-methyltetrahydrofuran-3-yl)oxy)(phenoxy)phosphoryl)-D-alaninate **(2c)**: ^{1}H NMR (500 MHz, *CDCl$_3$*): δ 1.29 (dd, J = 12.0, 6.2 Hz, 6 H), 1.44 (s, 3H), 1.46 (d, J = 8.0 Hz, 3 H), 3.51 (dd, J = 13.5, 2.1 Hz, 1 H), 3.83 (d, J = 13.5, 1 H), 3.95 (d, J = 8.2 Hz, 1 H), 4.13-4.05 (m, 1 H), 4.40 (dd, J = 12.9, 9.8 Hz, 1 H), 4.73 (t, J = 8.6 Hz, 1 H), 5.09-5.04 (m, 1 H), 5.77 (d, J = 8.2 Hz, 1 H), 6.38 (s, 1 H), 7.27-7.24 (m, 3 H), 7.40 (t, J = 7.8 Hz, 2 H), 7.97 (d, J = 8.2 Hz, 1 H), 9.37 (s, 1 H). ^{13}C NMR (126 MHz, *CDCl$_3$*): δ 172.9 (d, J = 8.1 Hz), 162.9, 150.3, 150.0 (d, J = 7.6 Hz), 139.5, 130.1 (2C), 126.1, 120.9 (d, J = 4.4 Hz, 2C), 118.0, 103.1, 87.1, 82.7 (d, J = 2.3 Hz), 76.0 (d, J = 4.3 Hz), 70.1, 58.5, 50.5, 48.1 (d, J = 6.1 Hz), 21.8, 21.8, 21.1 (d, J = 4.3 Hz), 17.0. MS = 537.32 (M+1). ^{31}P NMR (203 MHz, DMSO-d_6) δ 3.44. (major 95%). ^{31}P NMR (203 MHz, DMSO-d_6) δ 2.94. (minor 5%) HRMS calcd. For $C_{23}H_{29}N_4O_9P$ ($[M + H]^+$) 537.1750, found 537.1765.

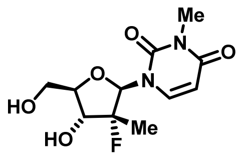

5

1-((2R,3R,4R,5R)-3-fluoro-4-hydroxy-5-(hydroxymethyl)-3-methyltetrahydrofuran-2-yl)-3-methylpyrimidine-2,4(1H,3H)-dione (5): To a solution of 1-((2R,3R,4R,5R)-3-fluoro-4-hydroxy-5-(hydroxymethyl)-3-methyltetrahydrofuran-2-yl)pyrimidine-2,4(1H,3H)-dione **(1a)** (1.0 g, 3.8 mmol) in DMF (15 ml) was added trimethylsulfonium hydroxide (20.0 ml, 4.0 mmol) and the vial was heated to 70 °C and monitored by HPLC. After 24 h added 1 ml more of trimethylsulfonium hydroxide and placed back at 70 °C. After 4 days quenched with water. The bi-phasic mixture was then diluted with EtOAc and the organic layer was separated and the aqueous layer was back extracted once with EtOAc. The combined organic layers were washed with saturated sodium thiosulfate, dried over $MgSO_4$, filtered and concentrated under reduced pressure. The residue was purified by silica gel chromatography to give

0.71 g (67%) of 1-((2R,3R,4R,5R)-3-fluoro-4-hydroxy-5-(hydroxymethyl)-3-methyltetrahydrofuran-2-yl)-3-methylpyrimidine-2,4(1H,3H)-dione **(5)**: ^{1}H NMR (400 MHz, DMSO-d_6) δ 8.04 (d, *J* = 8.2 Hz, 1H), 6.04 (d, *J* = 18.6 Hz, 1H), 5.80 (d, *J* = 8.1 Hz, 1H), 5.68 (d, *J* = 6.4 Hz, 1H), 5.33 (s, 1H), 3.84 (dt, *J* = 14.0, 6.4 Hz, 3H), 3.69 – 3.57 (m, 1H), 3.17 (s, 3H), 1.25 (d, *J* = 22.5 Hz, 3H). ^{13}C NMR (101 MHz, DMSO-d_6) δ 162.3, 151.3, 138.3, 102.3, 101.5, 100.5, 82.2, 70.7 (d, *J* = 17.3 Hz), 58.7, 27.7, 16.9 (d, *J* = 25.1 Hz). LRMS = 275.31 (M+1). HRMS calcd. For $C_{11}H_{15}FN_2O_5$ ([M + H]+) 275.1043, found 275.1050

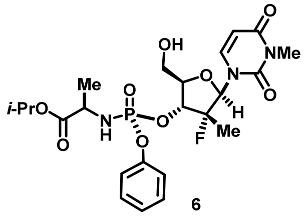

6

Isopropyl ((*R*)-(((*2R,3R,4R,5R*)-4-fluoro-2-(hydroxymethyl)-4-methyl-5-(3-methyl-2,4-dioxo-3,4-dihydropyrimidin-1(2H)-yl)tetrahydrofuran-3-yl)oxy)(phenoxy)phosphoryl)-D-alaninate (6). According to the general procedure A, treatment of (*R*)-isopropyl 2-(((*R*)-(perfluorophenoxy)(phenoxy)phosphoryl)amino)propanoate **(4)** (0.41 g, 0.91 mmol) and 1-((*2R,3R,4R,5R*)-3-fluoro-4-hydroxy-5-(hydroxymethyl)-3-methyltetrahydrofuran-2-yl)-3-methylpyrimidine-2,4(1H,3H)-dione **(5)** (0.25 g, 0.91 mmol) with DBU (0.14 ml, 0.96 mmol) gave 0.46 g (93%) of isopropyl ((R)-(((*2R,3R,4R,5R*)-4-fluoro-2-(hydroxymethyl)-4-methyl-5-(3-methyl-2,4-dioxo-3,4-dihydropyrimidin-1(2H)-yl)tetrahydrofuran-3-yl)oxy)(phenoxy)phosphoryl)-D-alaninate **(6)**: ^{1}H NMR (500 MHz, *CDCl$_3$*): δ 1.21 (dd, J = 6.2, 2.0 Hz, 6 H), 1.39-1.35 (m, 6 H), 3.29 (s, 3 H), 3.87-3.52 (m, 3 H), 3.98-3.91 (m, 2 H), 4.38 (dd, J = 12.5, 9.8 Hz, 1 H), 4.68-4.60 (m, 1 H), 5.00-4.95 (m, 1 H), 5.75 (d, J = 8.2 Hz, 1 H), 6.17 (d, J = 18.2 Hz, 1 H), 7.19-7.16 (m, 3 H), 7.32 (t, J = 7.8 Hz, 2 H), 7.87 (d, J = 8.2 Hz, 1 H). ^{13}C NMR (126 MHz, *CDCl$_3$*): δ 172.7 (d, J = 8.3 Hz), 162.6, 151.2, 150.1 (d, J = 7.3 Hz), 137.3, 129.8 (d, J = 1.1 Hz, 2C), 125.7 (d, J = 1.4 Hz), 120.6 (d, J = 4.5 Hz, 2C), 102.3, 99.6 (dd, J = 186.9, 5.7 Hz), 89.3 (br d, J = 38.8 Hz), 80.5 (d, J = 2.7 Hz), 73.8 (dd, J = 16.6, 4.7 Hz), 69.6, 58.6, 50.4 (d, J = 1.0 Hz), 27.7, 21.7, 21.6, 20.9 (d, J = 4.3 Hz), 16.3 (d, J = 25.4 Hz). ^{31}P NMR (203 MHz, DMSO-d_6) δ 3.64. MS = 544.36 (M+1). HRMS calcd. For $C_{23}H_{31}FN_3O_9P$ ($[M + H]^+$) 544.1860, found 544.1880.

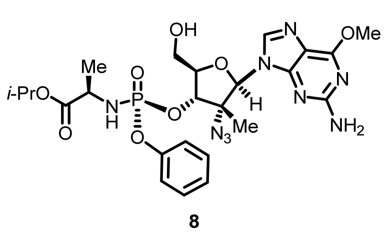

8

Isopropyl ((*R*)-(((*2R,3S,4R,5R*)-5-(2-amino-6-methoxy-9H-purin-9-yl)-4-azido-2-(hydroxymethyl)-4-methyltetrahydrofuran-3-yl)oxy)(phenoxy)phosphoryl)-D-alaninate (8). According to the general procedure A, treatment of (*R*)-isopropyl 2-(((*R*)-(perfluorophenoxy)(phenoxy)phosphoryl)amino) propanoate **(4)** (0.34 g, 0.74 mmol) and (*2R,3S,4R,5R*)-5-(2-amino-6-methoxy-9H-purin-9-yl)-4-azido-2-(hydroxymethyl)-4-methyltetrahydrofuran-3-ol **(7)** (0.25 g, 0.74 mmol) with DBU (0.12 ml, 0.78 mmol) gave 0.38 g (84%) of isopropyl ((*R*)-(((*2R,3S,4R,5R*)-5-(2-amino-6-methoxy-9H-purin-9-yl)-4-azido-2-(hydroxymethyl)-4-

methyltetrahydrofuran-3-yl)oxy)(phenoxy)phosphoryl)-D-alaninate **(8)**: ^{1}H NMR (400 MHz, *CDCl$_3$*) δ 7.90 (s, 1H), 7.37 – 7.24 (m, 4H), 7.21 – 7.14 (m, 1H), 5.75 (s, 1H), 5.44 (t, J = 9.1 Hz, 1H), 5.10 (bs, 2H), 5.00 (h, J = 6.3 Hz, 1H), 4.23 – 4.12 (m, 2H), 4.05 (s, 3H), 4.05 – 3.93 (m, 2H), 3.79 (dd, J = 13.1, 2.1 Hz, 1H), 1.40 (d, J = 7.0 Hz, 3H), 1.25 (s, 3H), 1.24 – 1.19 (m, 6H). ^{13}C NMR (101 MHz, *CDCl$_3$*) δ 172.6 (d, *J* = 8.3 Hz), 161.9, 159.3, 152.5, 150.4 (d, *J* = 6.9 Hz), 137.8, 129.8 (2C), 125.3, 120.2 (d, *J* = 4.8 Hz, 2C), 116.0, 90.7, 81.6 (d, *J* = 3.7 Hz), 77.1 (d, *J* = 4.1 Hz), 70.2 (d, *J* = 4.9 Hz), 69.5, 59.5, 54.0, 50.6 (d, *J* = 1.5 Hz), 21.6, 21.6, 21.2 (d, *J* = 4.3 Hz), 16.3. ^{31}P NMR (203 MHz, DMSO-*d*$_6$) δ 3.53. MS = 606.41 (M+1). HRMS calcd. For $C_{24}H_{32}N_9O_8P$ ([M + H]$^+$) 606.2189, found 606.2206.

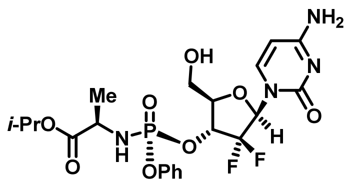

10

Isopropyl ((((2R,3R,5R)-5-(4-amino-2-oxopyrimidin-1(2H)-yl)-4,4-difluoro-2-(hydroxymethyl)tetrahydrofuran-3-yl)oxy)(phenoxy)phosphoryl)-D-alaninate (10): To a solution of (*R*)- 4-amino-1-((2R,4R,5R)-3,3-difluoro-4-hydroxy-5-(hydroxymethyl)tetrahydrofuran-2-yl)pyrimidin-2(1H)-one **(9)** (0.50 g, 1.90 mmol) and (R)-isopropyl 2-(((R)-(perfluorophenoxy)(phenoxy)phosphoryl)amino) propanoate **(4)** (0.86 g, 1.90 mmol) in THF (15 ml) and NMP (2.5 ml) at 0 °C was added DBU (0.20 ml, 1.99 mmol). The reaction was stirred at 0 °C for 3 h and then quenched with addition of 0.5 M citric acid. The bi-phasic mixture was then diluted with EtOAc and the organic layer was separated and the aqueous layer was back extracted once with EtOAc. The combined organic layers were washed with 10% LiCl solution 3 times the combined organic layers were azeoptroped with IPAc and amorphous material came out of solution and was filtered to give 0.66 g, 62% isolated yield. The mother liquors were analyzed and contained 0.24 g of isopropyl ((((2R,3R,5R)-5-(4-amino-2-oxopyrimidin-1(2H)-yl)-4,4-difluoro-2-(hydroxymethyl)tetrahydrofuran-3-yl)oxy)(phenoxy)phosphoryl)-D-alaninate **(10)** to give an assay yield of 85**%:** ^{1}H NMR (400 MHz, Acetonitrile-*d*$_3$) δ 7.70 – 7.57 (m, 1H), 7.47 – 7.37 (m, 2H), 7.32 – 7.19 (m, 3H), 6.64 (s, 1H), 6.29 (s, 1H), 6.24 – 6.14 (m, 1H), 5.87 (d, *J* = 7.5 Hz, 1H), 5.16 – 5.01 (m, 1H), 4.95 (hept, *J* = 6.3 Hz, 1H), 4.65 (s, 1H), 4.05 (dt, *J* = 8.1, 2.7 Hz, 1H), 3.96 (t, *J* = 8.0 Hz, 1H), 3.91 – 3.84 (m, 1H), 3.80 (s, 1H), 3.71 (dd, *J* = 12.1, 3.0 Hz, 1H), 1.36 (dd, *J* = 7.1, 0.9 Hz, 3H), 1.21 (s, 3H), 1.20 (s, 3H). ^{13}C NMR (101 MHz, Acetonitrile-*d$_3$*) δ 172.48 (d, J = 6.5 Hz), 166.32, 155.25, 150.59 (d, J = 6.8 Hz), 141.50, 129.82 (2C), 125.26, 123.05 (dd, J = 264.7, 260.1 Hz), 120.27 (d, J = 4.7 Hz, 2C), 94.84, 84.91, 79.48 (d, J = 4.3 Hz), 71.97 (td, J = 23.3, 4.4 Hz), 68.84, 58.91, 50.51, 20.87, 20.83, 19.75 (d, J = 5.6 Hz). ^{31}P NMR (203 MHz, Acetonitrile-*d*$_3$) δ 2.94. MS = 533.44 (M+1). HRMS calcd. For $C_{21}H_{27}F_2N_4O_8P$ ([M + H]$^+$) 533.1613, found 533.1618.

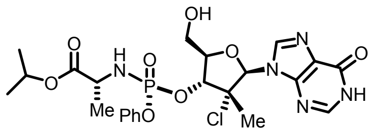

12

Isopropyl ((R)-(((2R,3R,4R,5R)-4-chloro-2-(hydroxymethyl)-4-methyl-5-(6-oxo-1,6-dihydro-9H-purin-9-yl)tetrahydrofuran-3-yl)oxy)(phenoxy)phosphoryl)-D-alaninate (12). To a solution of 9-((2R,3R,4R,5R)-3-chloro-4-hydroxy-5-(hydroxymethyl)-3-methyltetrahydrofuran-2-yl)-1H-purin-6(9H)-one **(11)** (0.24 g, 0.74 mmol) and (R)-isopropyl 2-(((R)-(perfluorophenoxy) (phenoxy)phosphoryl)amino)propanoate **(4)** (0.34 g, 0.74 mmol) in THF (7 ml) and NMP (1 ml) at -15 °C was added DBU (0.13 ml, 0.89 mmol) dropwise. The reaction was stirred at -15 °C for 4 h and then an additional 0.1 ml of DBU was added and the reaction was left at -15 °C overnight. The next morning the reaction was quenched with addition of 0.1 M HCl. The bi-phasic mixture was then diluted with EtOAc and the organic layer was separated and the aqueous layer was back extracted once with EtOAc. The combined organic layers were washed with 10% brine, dried over $MgSO_4$, filtered and concentrated under reduced pressure. The residue was purified by silica gel chromatography to give 0.24 g (57%) of isopropyl ((R)-(((2R,3R,4R,5R)-4-chloro-2-(hydroxymethyl)-4-methyl-5-(6-oxo-1,6-dihydro-9H-purin-9-yl)tetrahydrofuran-3-yl)oxy)(phenoxy)phosphoryl)-D-alaninate **(12)**: ^{1}H NMR (400 MHz, DMSO-d_6) δ 12.44 (s, 1H), 8.59 (s, 1H), 8.12 (s, 1H), 7.40 (dd, *J* = 8.6, 7.2 Hz, 2H), 7.26 (dt, *J* = 8.6, 1.2 Hz, 2H), 7.24 – 7.18 (m, 1H), 6.47 (s, 1H), 6.28 (dd, *J* = 13.2, 10.2 Hz, 1H), 5.43 (t, *J* = 4.5 Hz, 1H), 5.15 (t, *J* = 9.1 Hz, 1H), 4.87 (hept, *J* = 6.3 Hz, 1H), 4.19 (ddd, *J* = 8.8, 3.2, 2.1 Hz, 1H), 3.95 – 3.80 (m, 2H), 3.75 (dt, *J* = 12.6, 3.6 Hz, 1H), 1.35 (s, 3H), 1.27 (dd, *J* = 7.1, 1.0 Hz, 3H), 1.18 (d, *J* = 1.6 Hz, 3H), 1.16 (d, *J* = 1.6 Hz, 3H). ^{13}C NMR (101 MHz, DMSO-d_6) δ 172.82 (d, *J* = 5.6 Hz), 156.94, 151.00 (d, *J* = 6.8 Hz), 148.19, 146.89, 138.19, 130.12 (2C), 125.29, 124.78, 120.64 (d, *J* = 4.6 Hz, 2C), 91.59, 82.30 (d, *J* = 5.2 Hz), 76.24 (d, *J* = 4.2 Hz), 75.47 (d, *J* = 5.0 Hz), 68.48, 58.82, 50.49, 22.87, 21.92, 21.86, 20.17 (d, *J* = 6.6 Hz). ^{31}P NMR (203 MHz, DMSO-d_6) δ 3.28. HRMS calcd. For $C_{23}H_{29}ClN_5O_8P$ ($[M + H]^+$) 570.1520, found 570.1533.

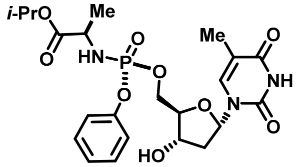

14

Isopropyl ((R)-(((2R,3S,5S)-3-hydroxy-5-(5-methyl-2,4-dioxo-3,4-dihydropyrimidin-1(2H)-yl)tetrahydrofuran-2-yl)methoxy)(phenoxy)phosphoryl)-D-alaninate (14): According to the general procedure A, treatment of α-thymidine **(13)** (0.20 g, 0.82 mmol) and (R)-isopropyl 2-(((R)-(perfluorophenoxy) (phenoxy)phosphoryl)amino)propanoate **(4)** (0.37 g, 0.82 mmol) THF (8 ml) and NMP (1ml) with DBU (0.13 ml, 0.87 mmol) gave 0.56 g of a (3:1) p-epi diastereomeric mixture of **14** and contaminated with citric acid. An NMR yield was calculated to provide 0.224 g (53%) of isopropyl ((R)-(((2R,3S,5S)-3-hydroxy-5-(5-methyl-2,4-dioxo-3,4-dihydropyrimidin-1(2H)-yl)tetrahydrofuran-2-yl)methoxy)(phenoxy)phosphoryl)-D-alaninate **(14)**. The material was

separated on SFC to provide clean NMR spectra of the major diastereomer: ^{1}H NMR (400 MHz, Methanol-d_4) δ 7.76 (d, *J* = 1.3 Hz, 1H), 7.39 (dd, *J* = 8.7, 7.0 Hz, 2H), 7.32 – 7.13 (m, 3H), 6.20 (dd, *J* = 7.6, 3.1 Hz, 1H), 4.99 (hept, *J* = 6.3 Hz, 1H), 4.44 (dp, *J* = 3.8, 2.1 Hz, 1H), 4.34 (dt, *J* = 6.4, 2.4 Hz, 1H), 4.16 (qdd, *J* = 11.1, 6.1, 3.7 Hz, 2H), 3.93 (dq, *J* = 10.0, 7.1 Hz, 1H), 2.48 (ddd, *J* = 14.2, 7.5, 6.3 Hz, 1H), 1.98 (dt, *J* = 14.5, 2.9 Hz, 1H), 1.89 (d, *J* = 1.2 Hz, 3H), 1.37 (dd, *J* = 7.1, 1.0 Hz, 3H), 1.23 (dd, *J* = 6.3, 1.0 Hz, 6H). ^{13}C NMR (101 MHz, Methanol-d_4) δ 173.05 (d, *J* = 5.4 Hz), 165.11, 150.96, 150.82 (d, *J* = 7.1 Hz), 137.34, 129.47 (2C), 124.85 (d, *J* = 1.3 Hz), 120.04 (d, *J* = 4.6 Hz, 2C), 109.55, 86.88 (d, *J* = 8.0 Hz), 86.72, 70.99, 68.77, 66.53 (d, *J* = 5.6 Hz), 50.24, 39.97, 20.59, 20.54, 19.19 (d, *J* = 6.4 Hz), 11.18. ^{31}P NMR (203 MHz, Methanol-d_4) δ 3.65. MS = 512.45 (M+1). HRMS calcd. For $C_{22}H_{30}N_3O_9P$ ([M + H]+) 512.1798, found 512.1800.

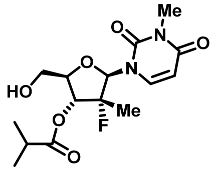
19

(2R,3R,4R,5R)-4-Fluoro-2-(hydroxymethyl)-4-methyl-5-(3-methyl-2,4-dioxo-3,4-dihydropyrimidin-1(2H)-yl)tetrahydrofuran-3-yl isobutyrate (19): To a solution of 1-((2R,3R,4R,5R)-3-fluoro-4-hydroxy-5-(hydroxymethyl)-3-methyltetrahydrofuran-2-yl)-3-methylpyrimidine-2,4(1H,3H)-dione **(5)** (0.22 g, 0.79 mmol) in THF (8 ml) at -15 °C was added DBU (0.13 ml, 0.83 mmol) dropwise and then isobutyric anhydride **(18)** (0.15 ml, 0.91 mmol) was added dropwise. The reaction was stirred at -15 °C for 1 h and then quenched with addition of 0.5 M citric acid. The bi-phasic mixture was then diluted with EtOAc and the organic layer was separated and the aqueous layer was back extracted once with EtOAc. The combined organic layers were washed with 10% brine, dried over $MgSO_4$, filtered and concentrated under reduced pressure. The residue was purified by silica gel chromatography to give 0.160 g (59%) of (2R,3R,4R,5R)-4-Fluoro-2-(hydroxymethyl)-4-methyl-5-(3-methyl-2,4-dioxo-3,4-dihydropyrimidin-1(2H)-yl)tetrahydrofuran-3-yl isobutyrate **(19)** and 0.025 g (11%) of **5** for a *'based on recovered starting material'* yield of 68%: ^{1}H NMR (400 MHz, *CDCl$_3$*) δ 7.98 (d, *J* = 8.1 Hz, 1H), 6.24 (d, *J* = 18.2 Hz, 1H), 5.81 (d, *J* = 8.1 Hz, 1H), 5.09 (dd, *J* = 23.0, 9.4 Hz, 1H), 4.16 (dd, *J* = 9.4, 1.6 Hz, 1H), 4.07 (dd, *J* = 13.2, 1.8 Hz, 1H), 3.71 (dd, *J* = 13.2, 2.1 Hz, 1H), 3.33 (s, 3H), 2.70 (hept, *J* = 7.0 Hz, 1H), 1.35 (d, *J* = 22.1 Hz, 3H), 1.22 (dd, *J* = 7.0, 5.9 Hz, 6H). ^{13}C NMR (101 MHz, *CDCl$_3$*) δ 177.20, 162.64, 151.17, 137.35, 102.28, 100.13 (d, *J* = 186.0 Hz), 89.90 (d, *J* = 39.1 Hz), 79.91, 70.80 (d, *J* = 16.1 Hz), 59.60, 33.82, 27.76, 18.95, 18.80, 16.94 (d, *J* = 25.3 Hz). HRMS calcd. For $C_{15}H_{21}FN_2O_6$ ($[M + H]^+$) 345.1462, found 345.1463.

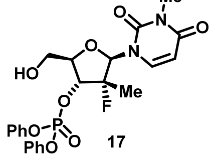
17

(2R,3R,4R,5R)-4-Fluoro-2-(hydroxymethyl)-4-methyl-5-(3-methyl-2,4-dioxo-3,4-dihydropyrimidin-1(2H)-yl)tetrahydrofuran-3-yl diphenyl phosphate (17): According to the general procedure A, treatment of perfluorophenyl diphenyl phosphate **(16)**

(0.23 g, 0.55 mmol) and 1-((2R,3R,4R,5R)-3-fluoro-4-hydroxy-5-(hydroxymethyl)-3-methyltetrahydrofuran-2-yl)-3-methylpyrimidine-2,4(1H,3H)-dione **(5)** (0.15 g, 0.55 mmol) with DBU (0.09 ml, 0.58 mmol) gave 0.20 g (71%) of (2R,3R,4R,5S)-4-fluoro-2-(hydroxymethyl)-4-methyl-5-(3-methyl-2,4-dioxo-3,4-dihydropyrimidin-1(2H)-yl)tetrahydrofuran-3-yl diphenyl phosphate **(17)**: ^{1}H NMR (400 MHz, DMSO-d_6) δ 7.97 (d, *J* = 8.2 Hz, 1H), 7.63 – 7.36 (m, 4H), 7.36 – 7.21 (m, 6H), 6.12 (d, *J* = 19.9 Hz, 1H), 5.85 (d, *J* = 8.1 Hz, 1H), 5.41 (s, 1H), 5.20 – 4.86 (m, 1H), 4.19 (d, *J* = 9.0 Hz, 1H), 3.99 – 3.74 (m, 1H), 3.60 (ddd, *J* = 12.6, 4.9, 3.4 Hz, 1H), 3.16 (s, 3H), 1.32 (d, *J* = 23.0 Hz, 3H). ^{13}C NMR (126 MHz, Acetonitrile-d_3) δ 163.90, 152.36, 151.16 (dd, *J* = 7.5, 2.0 Hz), 139.14, 131.18 (d, *J* = 2.9 Hz), 127.09 (t, *J* = 1.5 Hz), 121.05 (d, *J* = 4.4 Hz), 102.73, 101.60 (d, *J* = 4.0 Hz), 100.13 (d, *J* = 3.9 Hz), 91.05, 81.16 (d, *J* = 5.6 Hz), 76.83 (dd, *J* = 16.0, 5.4 Hz), 59.02, 28.10, 17.35 (d, *J* = 25.1 Hz). ^{31}P NMR (203 MHz, DMSO-d_6) δ -12.06. HRMS calcd. For $C_{23}H_{24}FN_2O_8P$ ([M + H]$^+$) 507.1332, found 507.1343.

NMR studies. NMR studies of **1a** have been conducted using 600 MHz Agilent VNMRS spectrometer equipped with 3mm cryoprobe. All samples used for NMR titration experiments were in prepared in d6-DMSO with 20mM concentration of **1a**. DIPEA and DBU concentrations varied from 10mM to 100mM, corresponding to 0.5 to 5 molar equivalents. All NOE experiments have been performed using a sample containing 200mM of **1a** and 200mM of DBU in CD_3CN. 1D NOE experiment utilized continuous-wave irradiation (at the NH frequency of 7.51 ppm) with mixing time of 3s and 160 transients, for a total experiment time of 15 minutes. 2D NOESY experiment was acquired with relaxation delay 2s and mixing time 400ms, using 400 × 2048 points with 8 transients accumulated per each t_1 increment for a total experiment time of 2 hours and 20 minutes.

Fully asigned ^{1}H and ^{13}C NMR spectra of **1a** in d6-DMSO are shown below.

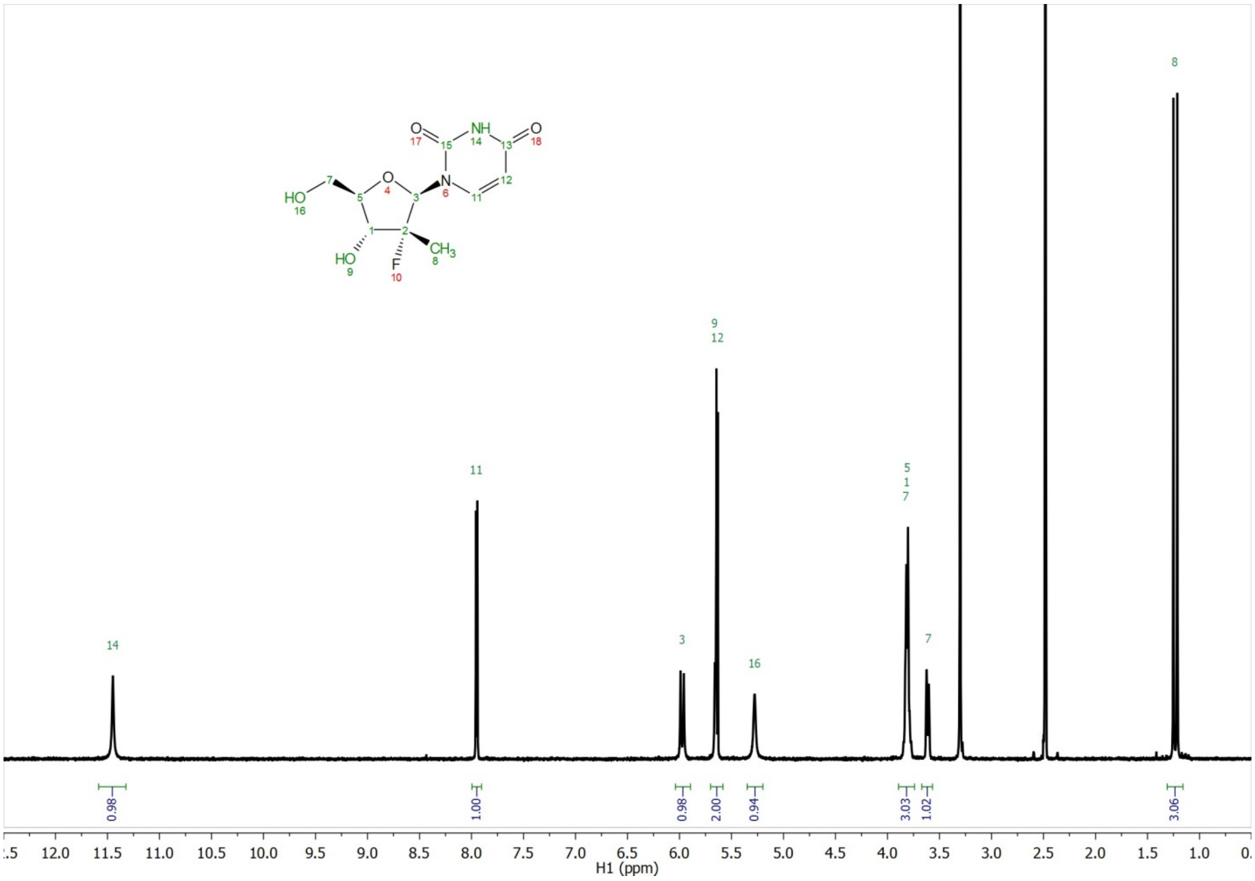
HO
HO
F
CH3
O
NH
N
14
11
3
9
12
16
5
1
7
8
0.98
1.00
0.98
2.00
0.94
3.03
1.02
3.06
12.0 11.5 11.0 10.5 10.0 9.5 9.0 8.5 8.0 7.5 7.0 6.5 6.0 5.5 5.0 4.5 4.0 3.5 3.0 2.5 2.0 1.5 1.0
H1 (ppm)

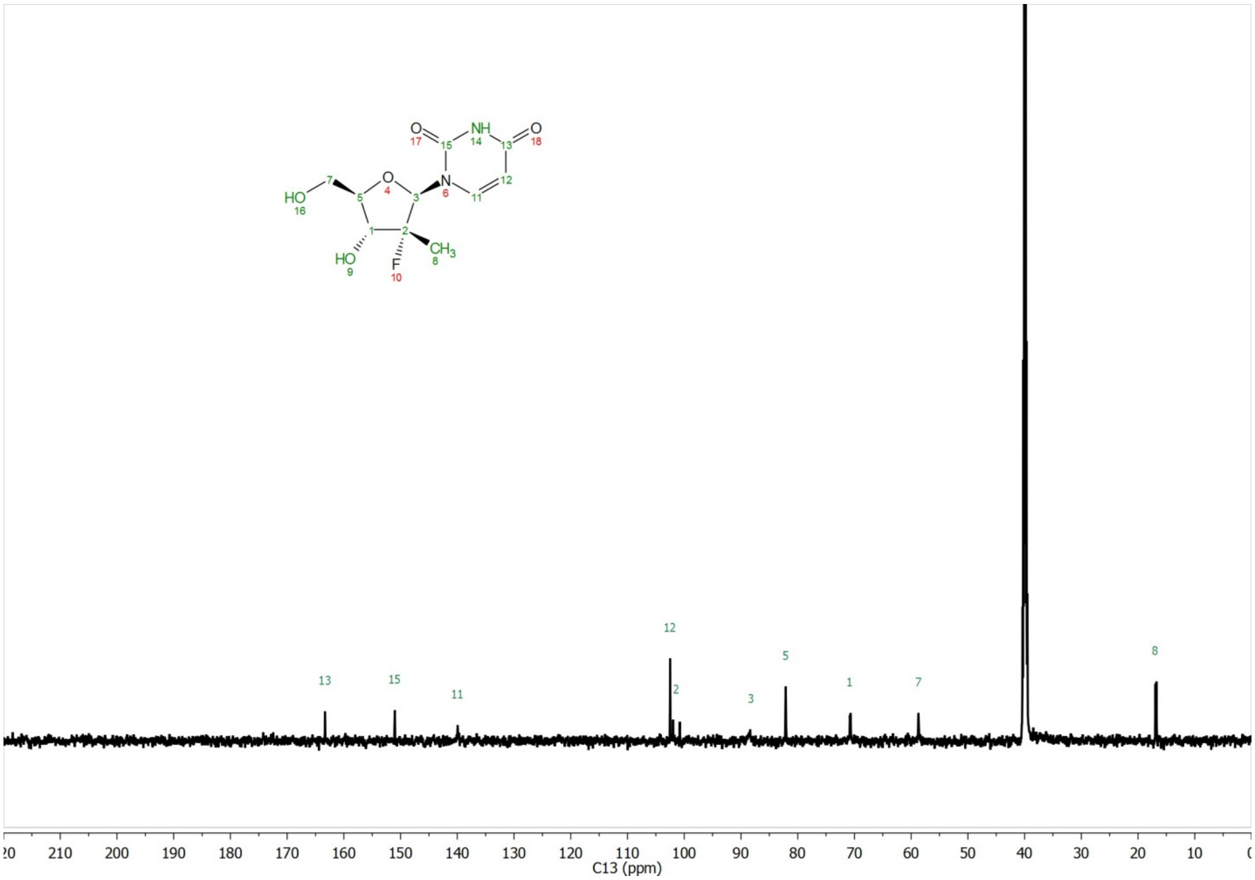

O
17
15
NH
14
13
O
18
12
HO
16
7
5
O
4
3
N
6
11
1
2
HO
9
F
10
CH3
8
12
2
5
13
15
11
3
1
7
8
C13 (ppm)

```
Current Data Parameters
NAME     0357314-0182-pdt
EXPNO                  5
PROCNO                 1

F2 - Acquisition Parameters
Date_           20150625
Time                8.29
INSTRUM            spect
PROBHD   5 mm PAQNP 1H/
PULPROG             zg30
TD                 65536
SOLVENT             DMSO
NS                    16
DS                     0
SWH            10000.000 Hz
FIDRES          0.152588 Hz
AQ             3.2767999 sec
RG                   181
DW                50.000 usec
DE                  6.50 usec
TE                 294.8 K
D1            1.00000000 sec
TD0                    1

======== CHANNEL f1 ========
SFO1         500.1330885 MHz
NUC1                  1H
P1                 10.00 usec
PLW1         39.99399948 W

F2 - Processing parameters
SI                 65536
SF           500.1300000 MHz
WDW                   EM
SSB      0
LB                  0.30 Hz
GB       0
PC                  1.00
```

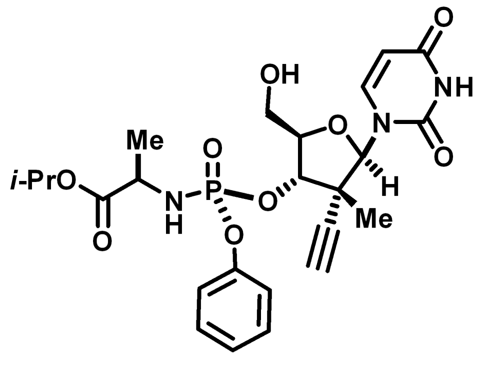

2c

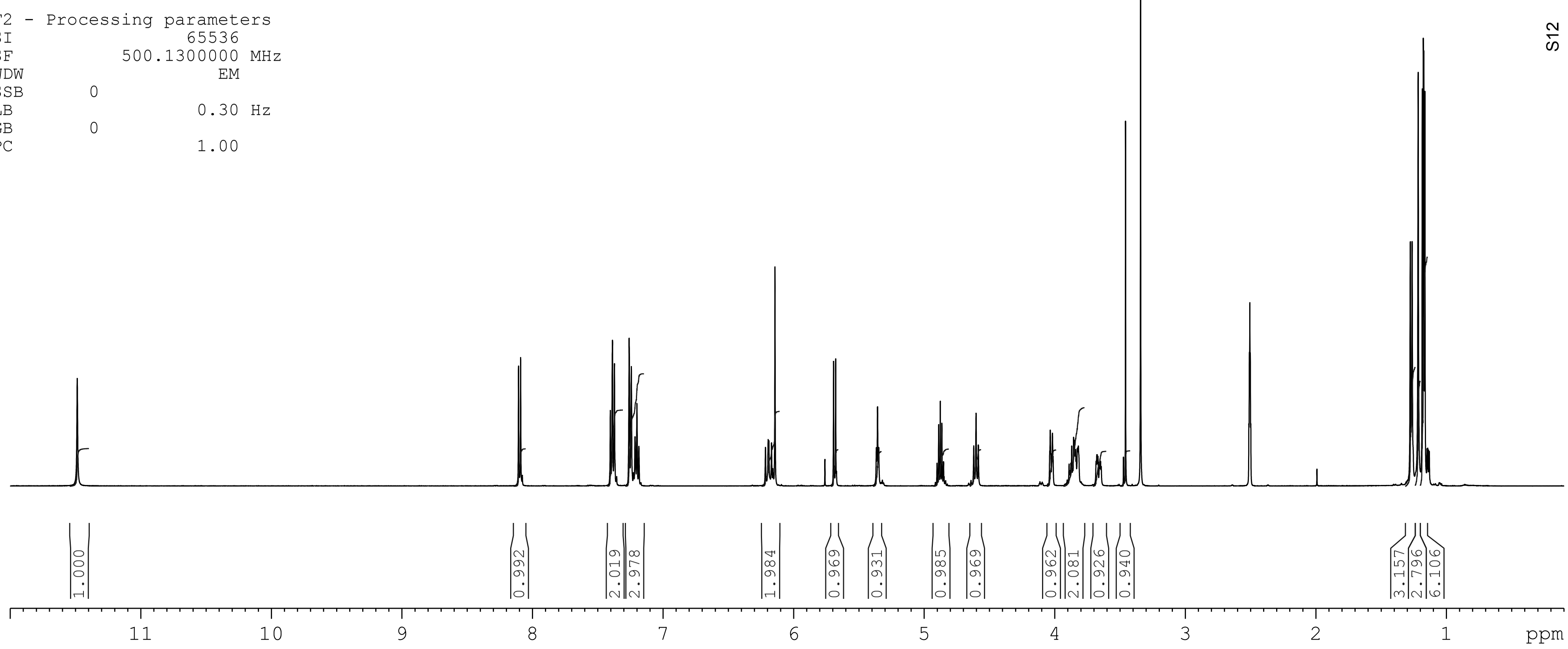

MRL_C13_8K DMSO /kwa2i jmccabed 42

2c

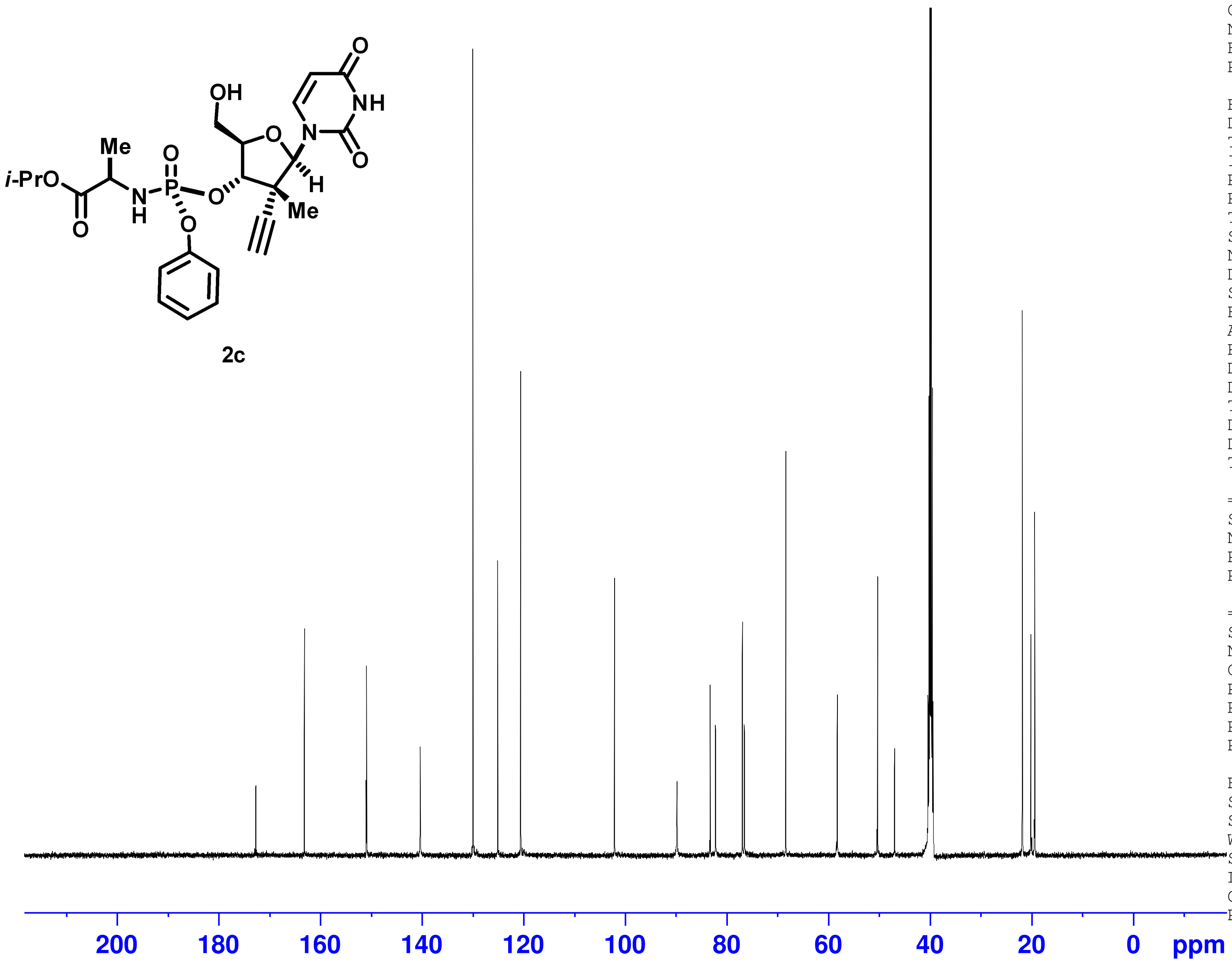


```
Current Data Parameters
NAME          0357314-0182-pdt
EXPNO                        6
PROCNO                       1

F2 - Acquisition Parameters
Date_               20150625
Time                   22.53
INSTRUM                spect
PROBHD     5 mm PAQNP 1H/
PULPROG                 zgpg
TD                     65536
SOLVENT                 DMSO
NS                      8192
DS                         0
SWH               29761.904 Hz
FIDRES             0.454131 Hz
AQ                1.1010048 sec
RG                       812
DW                    16.800 usec
DE                      6.50 usec
TE                     296.2 K
D1               0.50000000 sec
D11              0.03000000 sec
TD0                        1

======== CHANNEL f1 ========
SFO1          125.7703637 MHz
NUC1                   13C
P1                    13.30 usec
PLW1          29.99200058 W

======== CHANNEL f2 ========
SFO2          500.1320005 MHz
NUC2                    1H
CPDPRG[2           waltz16
PCPD2                 80.00 usec
PLW2          30.00000000 W
PLW12          0.67500001 W
PLW13          0.43200001 W

F2 - Processing parameters
SI                     32768
SF            125.7577890 MHz
WDW                       EM
SSB      0
LB                      1.00 Hz
GB       0
PC                      1.40
```

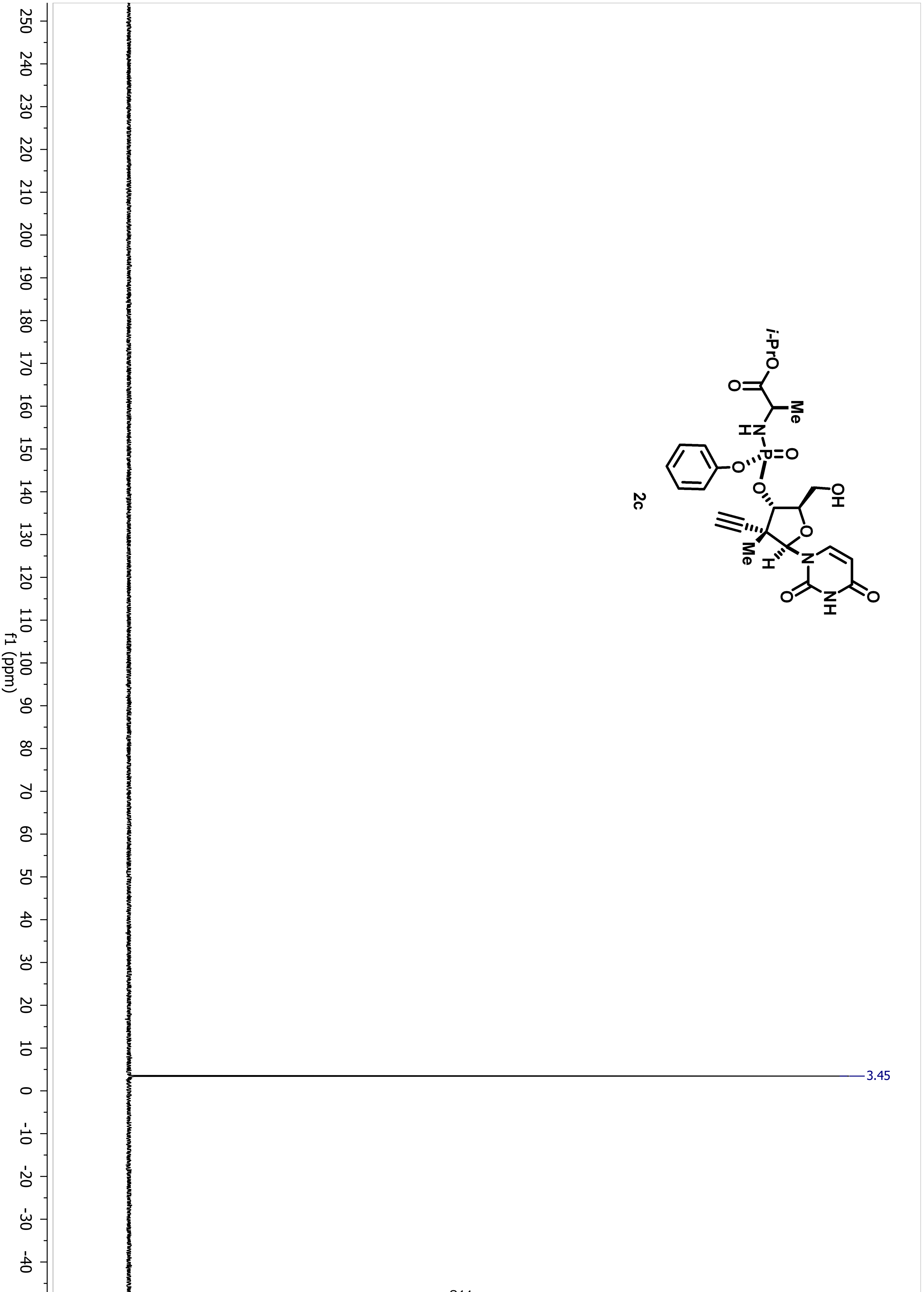
i-PrO
O
Me
H
N
P
O
O
O
OH
O
Me
H
N
O
NH
O
2c
3.45
f1 (ppm)
250 240 230 220 210 200 190 180 170 160 150 140 130 120 110 100 90 80 70 60 50 40 30 20 10 0 -10 -20 -30 -40 -50

```
Current Data Parameters
NAME      0357314-0190-pdt
EXPNO                    1
PROCNO                   1

F2 - Acquisition Parameters
Date_             20150629
Time                  8.33
INSTRUM              spect
PROBHD   5 mm PAQNP 1H/
PULPROG               zg30
TD                   65536
SOLVENT              CDCl3
NS                      16
DS                       0
SWH              10000.000 Hz
FIDRES            0.152588 Hz
AQ               3.2767999 sec
RG                    80.6
DW                  50.000 usec
DE                    6.50 usec
TE                   294.7 K
D1              1.00000000 sec
TD0                      1

======== CHANNEL f1 ========
SFO1          500.1330885 MHz
NUC1                    1H
P1                   10.00 usec
PLW1          39.99399948 W

F2 - Processing parameters
SI                   65536
SF            500.1300000 MHz
WDW                     EM
SSB      0
LB                    0.30 Hz
GB       0
PC                    1.00
```

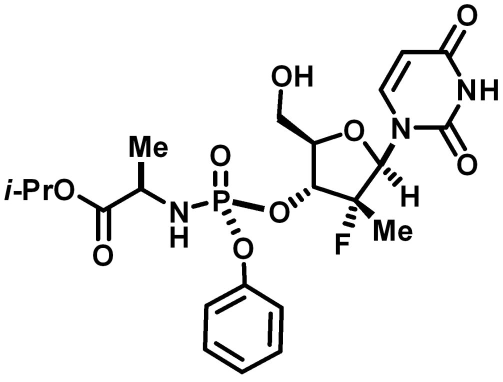

2a

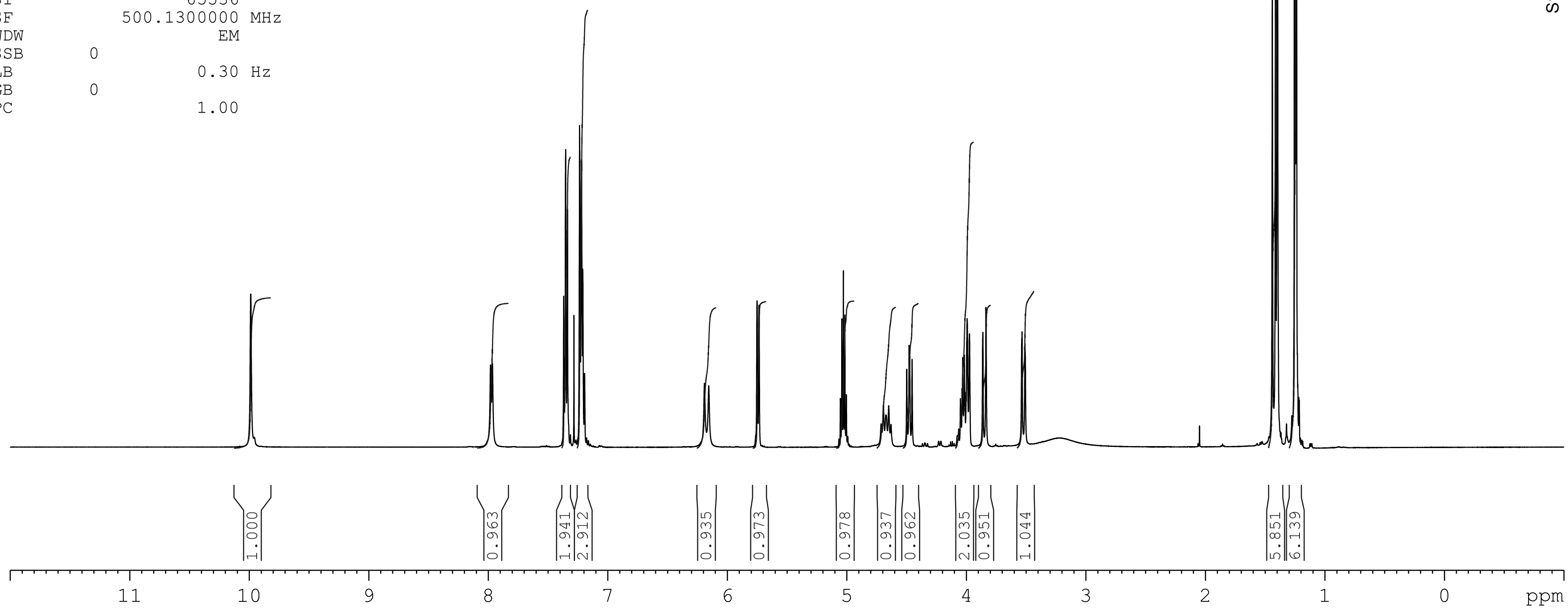

MRL_C13_256 CDCl3 /kwa2i jmccabed 15

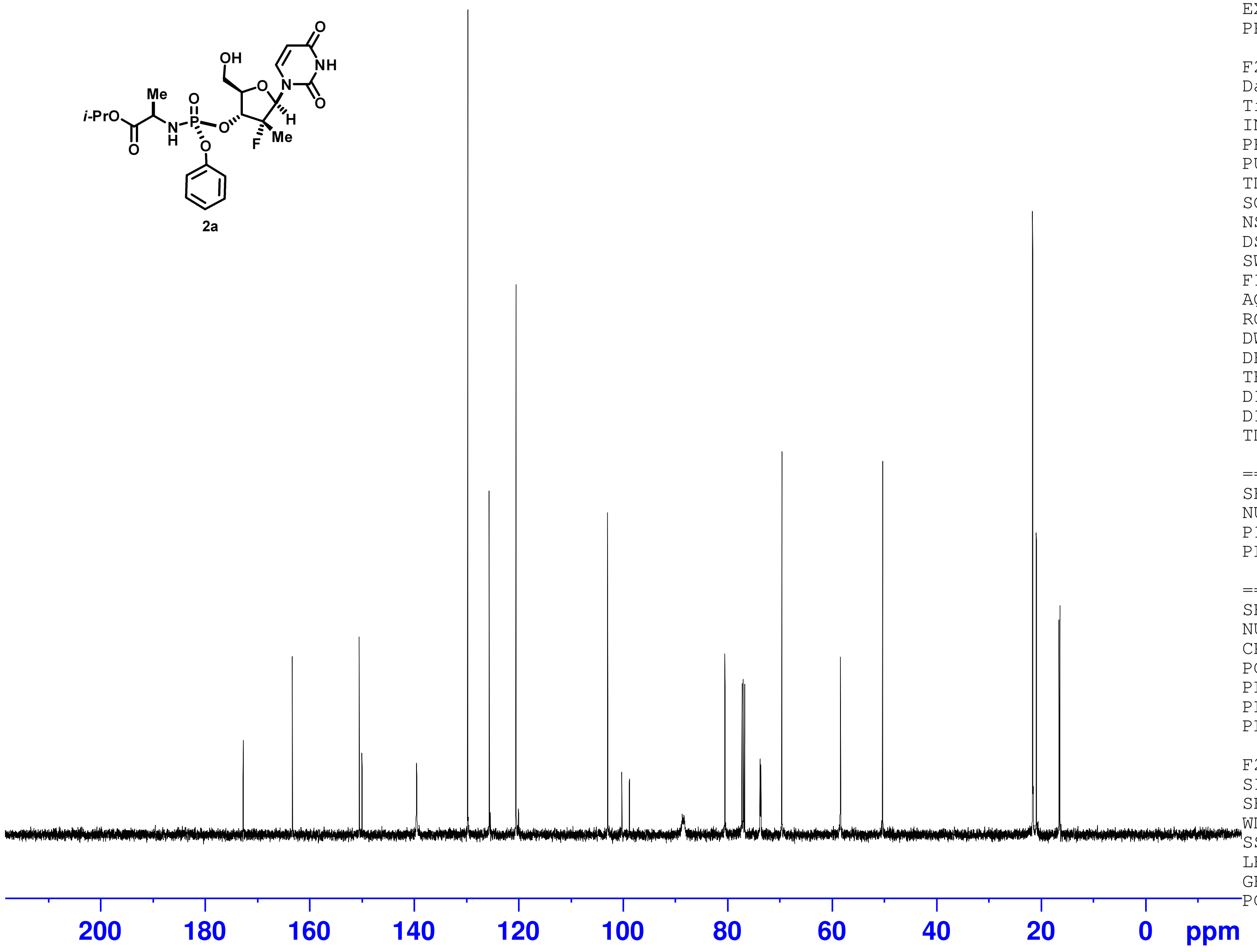


```
Current Data Parameters
NAME       0357314-0190-pdt
EXPNO                     2
PROCNO                    1

F2 - Acquisition Parameters
Date_              20150629
Time                   8.43
INSTRUM               spect
PROBHD    5 mm PAQNP 1H/
PULPROG                zgpg
TD                    65536
SOLVENT               CDCl3
NS                      256
DS                        8
SWH               29761.904 Hz
FIDRES             0.454131 Hz
AQ                1.1010048 sec
RG                      812
DW                   16.800 usec
DE                     6.50 usec
TE                    295.8 K
D1               0.50000000 sec
D11              0.03000000 sec
TD0                       1

======== CHANNEL f1 ========
SFO1            125.7703637 MHz
NUC1                    13C
P1                    13.30 usec
PLW1            29.99200058 W

======== CHANNEL f2 ========
SFO2            500.1320005 MHz
NUC2                     1H
CPDPRG[2            waltz16
PCPD2                 80.00 usec
PLW2            30.00000000 W
PLW12            0.67500001 W
PLW13            0.43200001 W

F2 - Processing parameters
SI                    32768
SF              125.7577890 MHz
WDW                      EM
SSB       0
LB                     1.00 Hz
GB        0
PC                     1.40
```

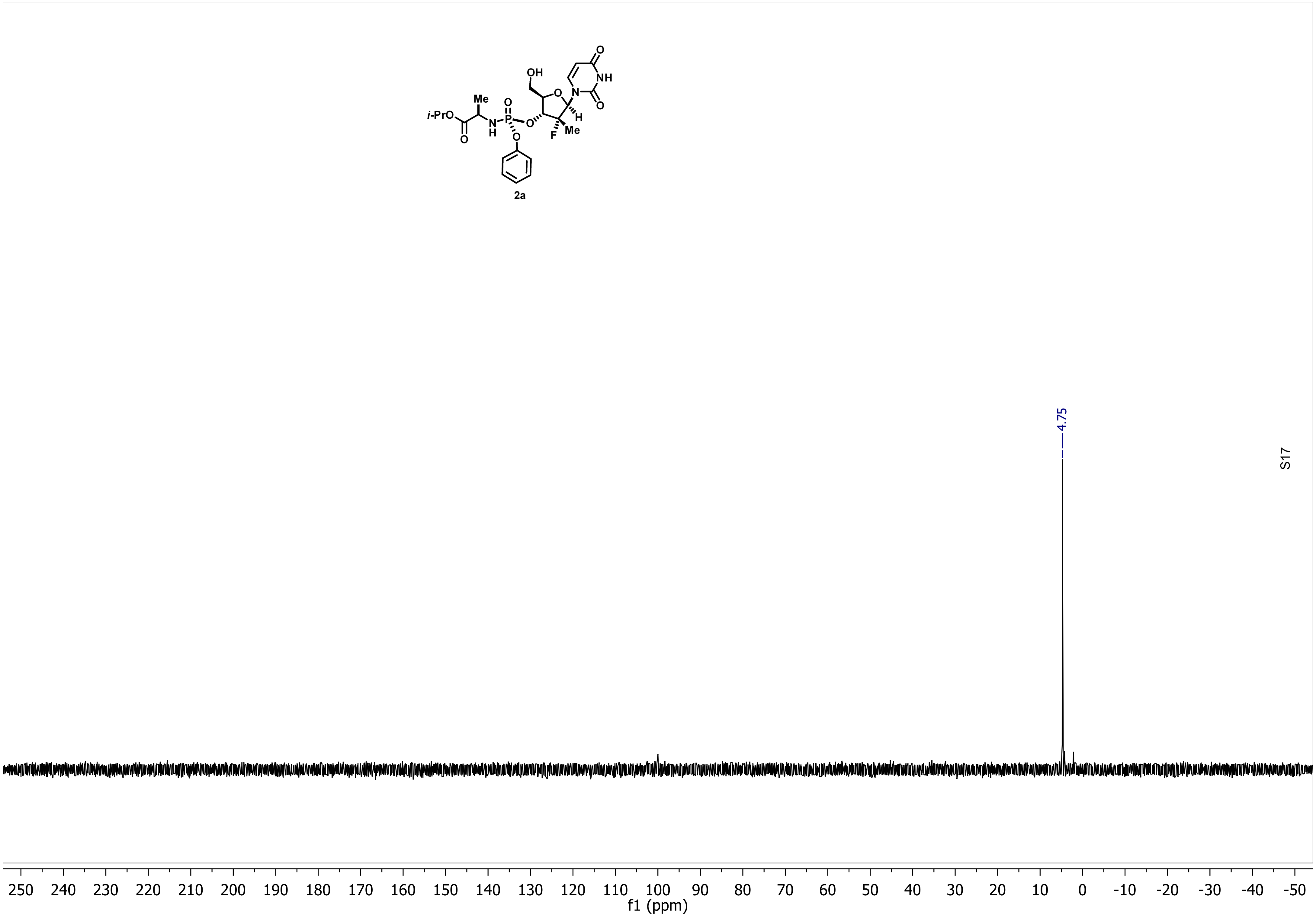
2a
4.75
250 240 230 220 210 200 190 180 170 160 150 140 130 120 110 100 90 80 70 60 50 40 30 20 10 0 -10 -20 -30 -40 -50
f1 (ppm)

spH1_16green DMSO /kwa1g jmccabed 85

0361768-0066

Current Data Parameters
NAME 0361768-0066
EXPNO 1
PROCNO 1

F2 - Acquisition Parameters
Date_ 20150624
Time_ 10.30
INSTRUM spect
PROBHD 5 mm PADUL 13C
PULPROG zg30
TD 65536
SOLVENT DMSO
NS 16
DS 0
SWH 10330.578 Hz
FIDRES 0.157632 Hz
AQ 3.1719425 sec
RG 512
DW 48.400 usec
DE 6.00 usec
TE 294.4 K
D1 1.00000000 sec
TD0 1

======== CHANNEL f1 ========
NUC1 1H
P1 8.00 usec
PL1 -1.00 dB
SFO1 500.2060888 MHz

F2 - Processing parameters
SI 32768
SF 500.2030000 MHz
WDW EM
SSB 0
LB 0.20 Hz
GB 0
PC 1.00

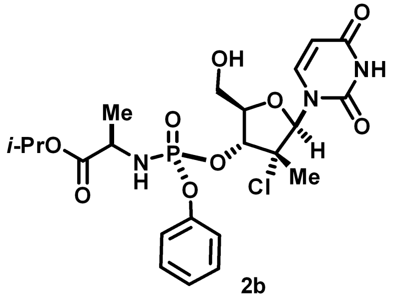

2b

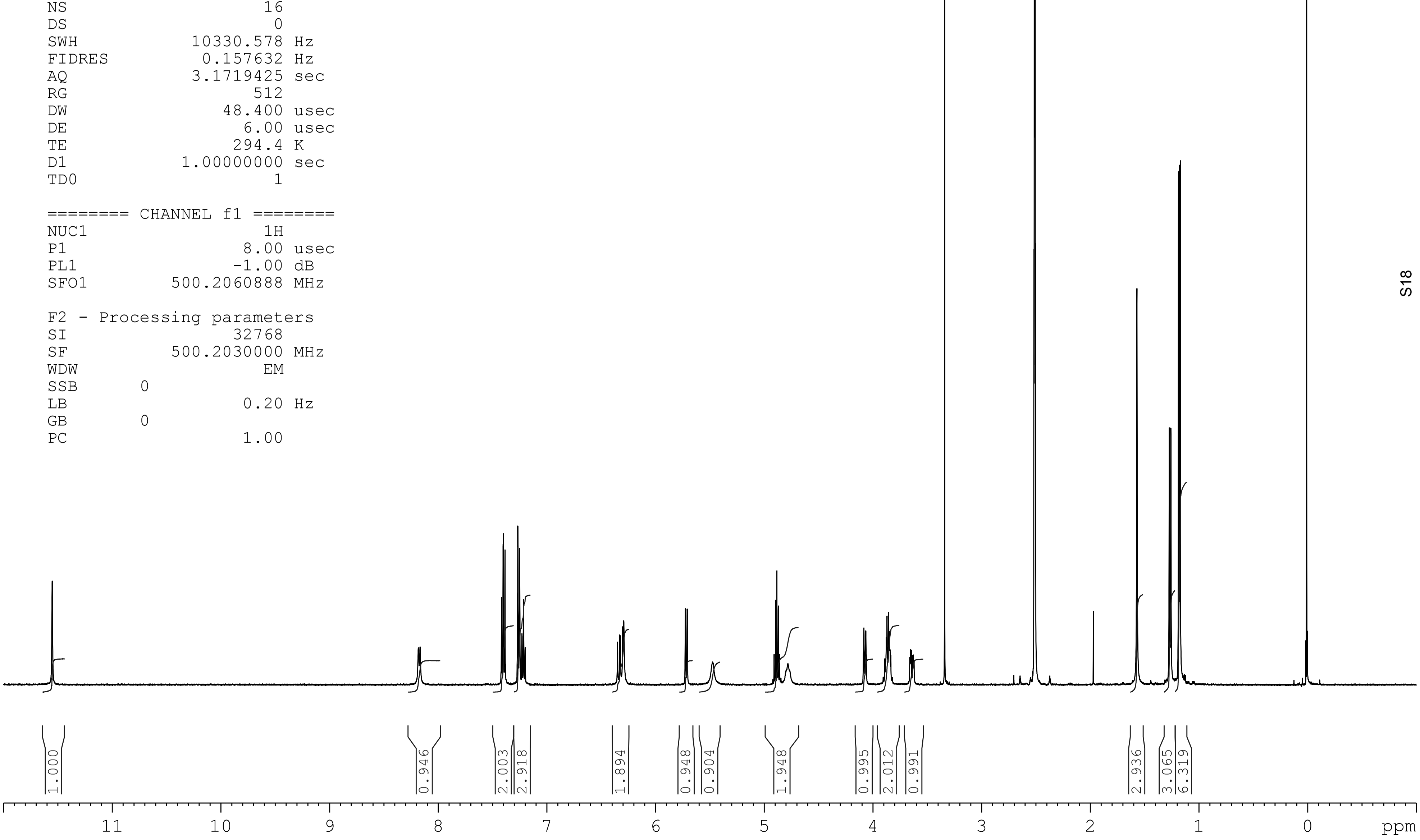

MRL_C13_8K DMSO /kwa2i jmccabed 21

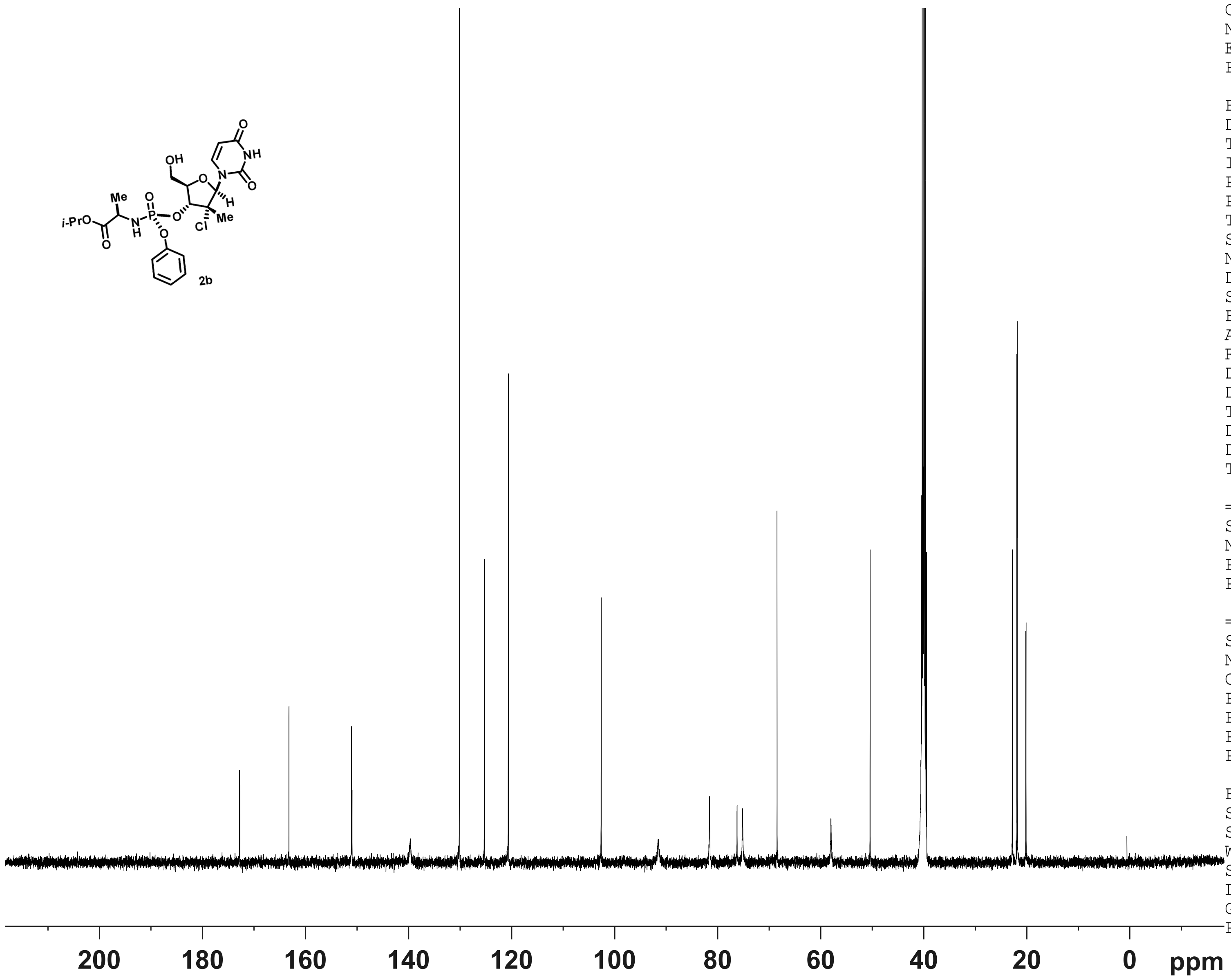

Current Data Parameters	
NAME	0361768-0066-pdt
EXPNO	1
PROCNO	1
F2 - Acquisition Parameters	
Date_	20150624
Time	22.52
INSTRUM	spect
PROBHD	5 mm PAQNP 1H/
PULPROG	zgpg
TD	65536
SOLVENT	DMSO
NS	8192
DS	0
SWH	29761.904 Hz
FIDRES	0.454131 Hz
AQ	1.1010048 sec
RG	812
DW	16.800 usec
DE	6.50 usec
TE	300.0 K
D1	0.50000000 sec
D11	0.03000000 sec
TD0	1
======== CHANNEL f1 ========	
SFO1	125.7703637 MHz
NUC1	13C
P1	13.30 usec
PLW1	29.99200058 W
======== CHANNEL f2 ========	
SFO2	500.1320005 MHz
NUC2	1H
CPDPRG[2	waltz16
PCPD2	80.00 usec
PLW2	30.00000000 W
PLW12	0.67500001 W
PLW13	0.43200001 W
F2 - Processing parameters	
SI	32768
SF	125.7577890 MHz
WDW	EM
SSB	0
LB	1.00 Hz
GB	0
PC	1.40

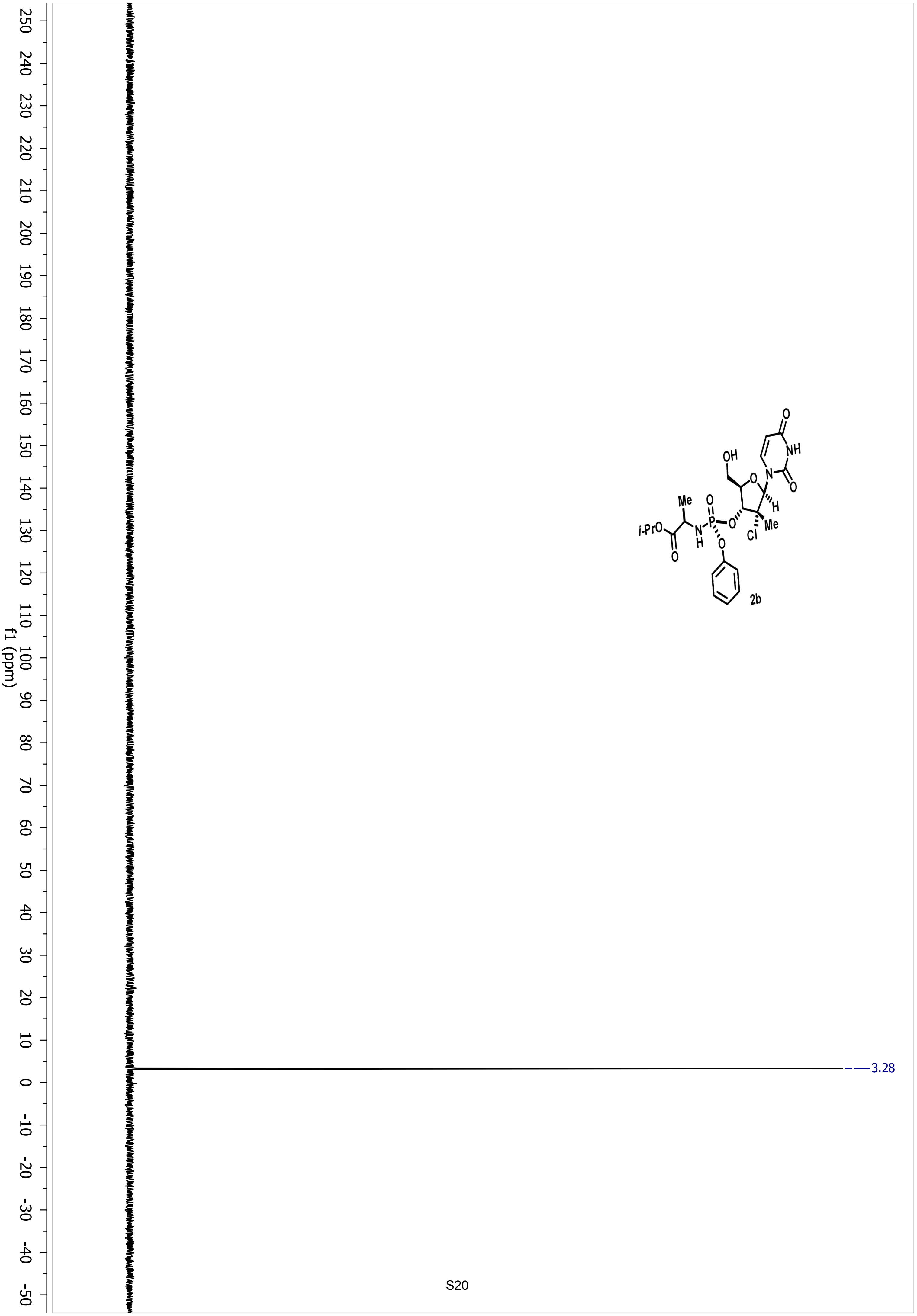

3.28
f1 (ppm)
250 240 230 220 210 200 190 180 170 160 150 140 130 120 110 100 90 80 70 60 50 40 30 20 10 0 -10 -20 -30 -40 -50
i-PrO
Me
NH
O
P
OH
NH
Cl
Me
H
2b

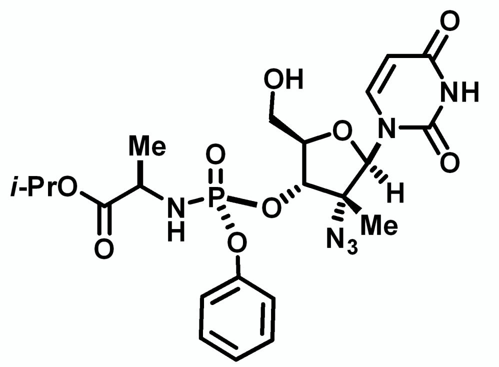

2d

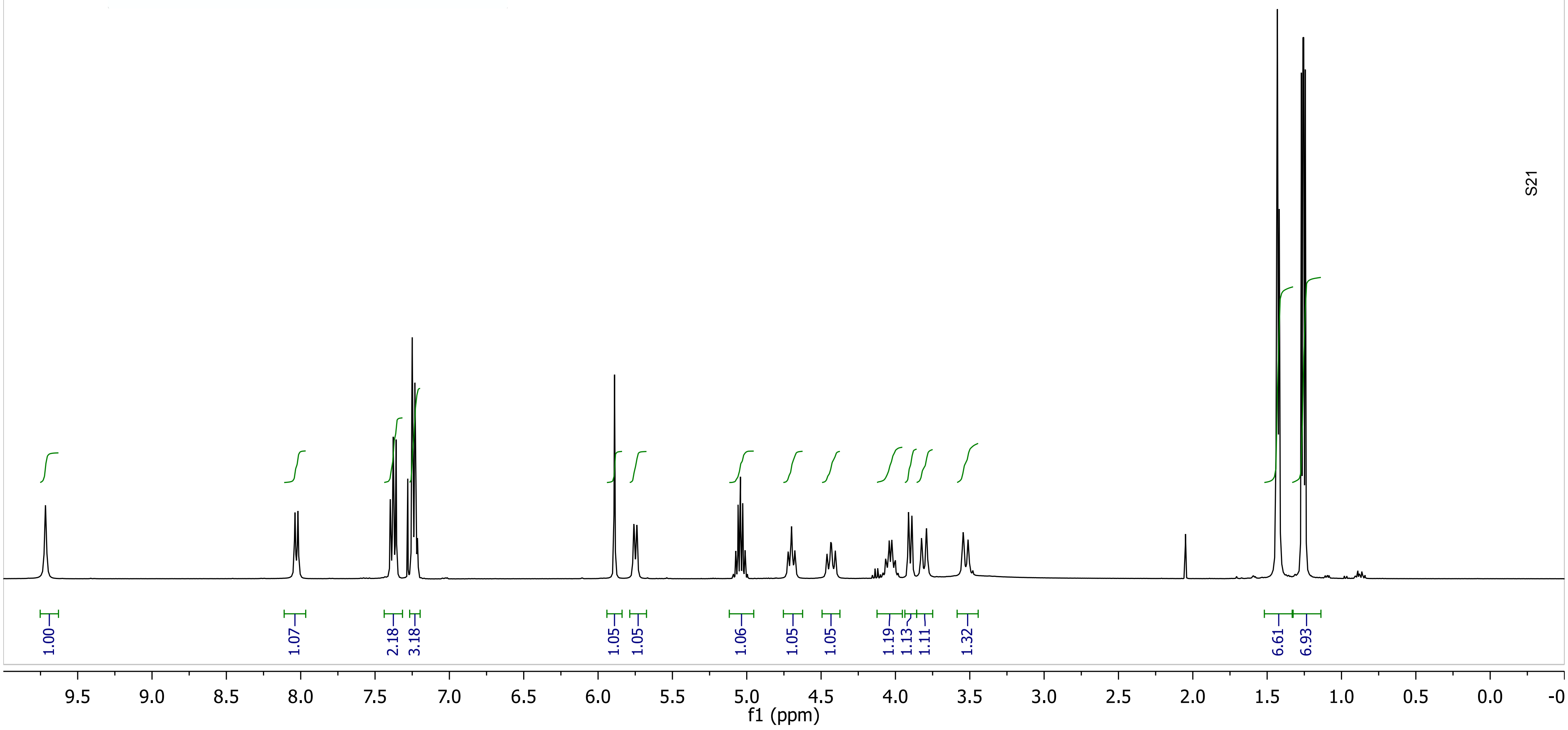

1.00
1.07
2.18
3.18
1.05
1.05
1.06
1.05
1.05
1.19
1.13
1.11
1.32
6.61
6.93
9.5 9.0 8.5 8.0 7.5 7.0 6.5 6.0 5.5 5.0 4.5 4.0 3.5 3.0 2.5 2.0 1.5 1.0 0.5 0.0 -0
f1 (ppm)

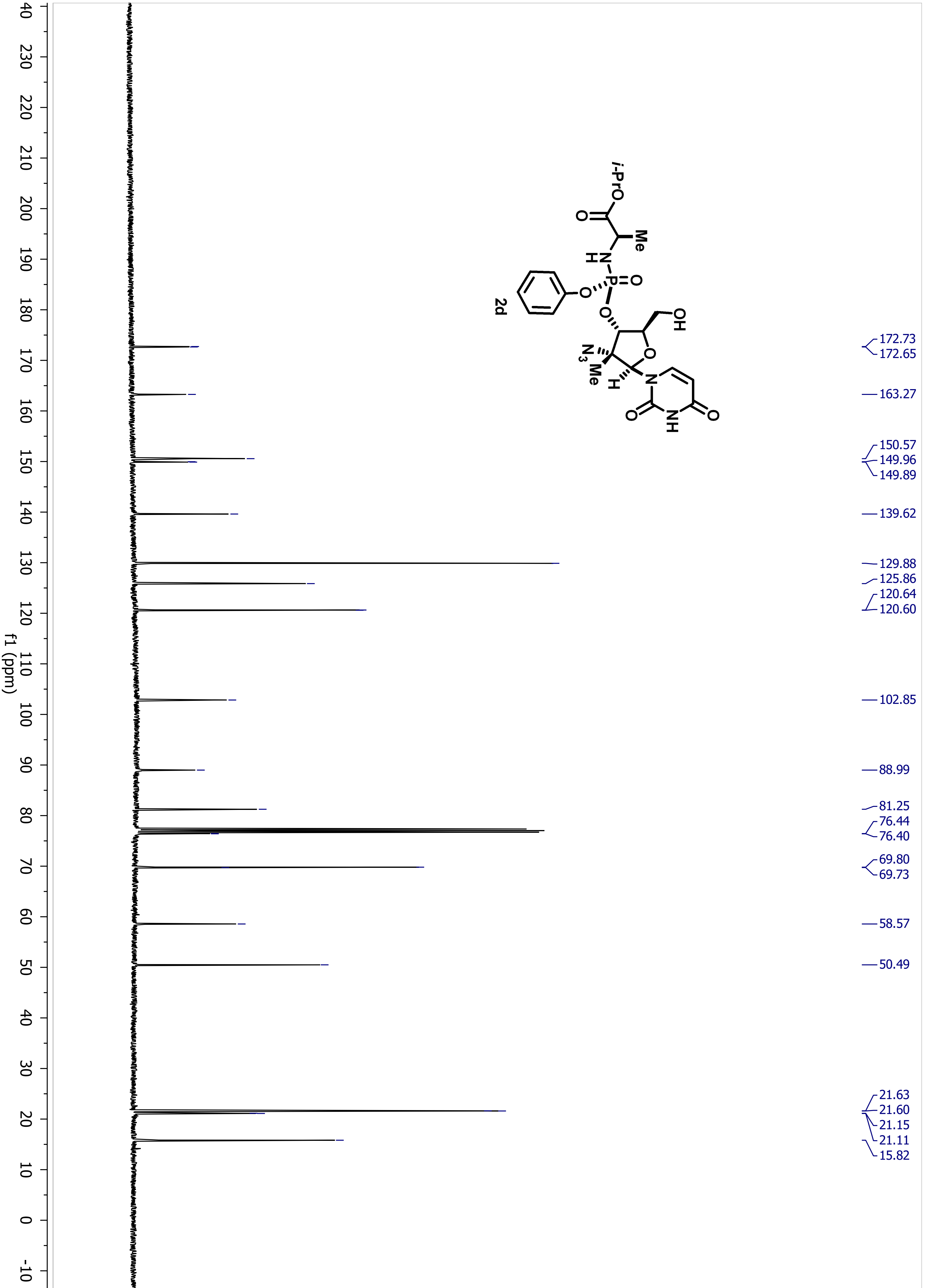
2d
i-PrO
Me
172.73
172.65
163.27
150.57
149.96
149.89
139.62
129.88
125.86
120.64
120.60
102.85
88.99
81.25
76.44
76.40
69.80
69.73
58.57
50.49
21.63
21.60
21.15
21.11
15.82
f1 (ppm)

^{31}P NMR (203 MHz, DMSO-d_6) δ 3.55

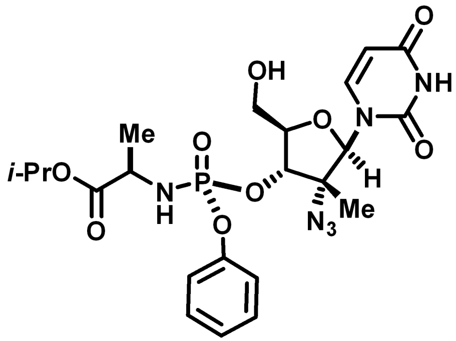

2d

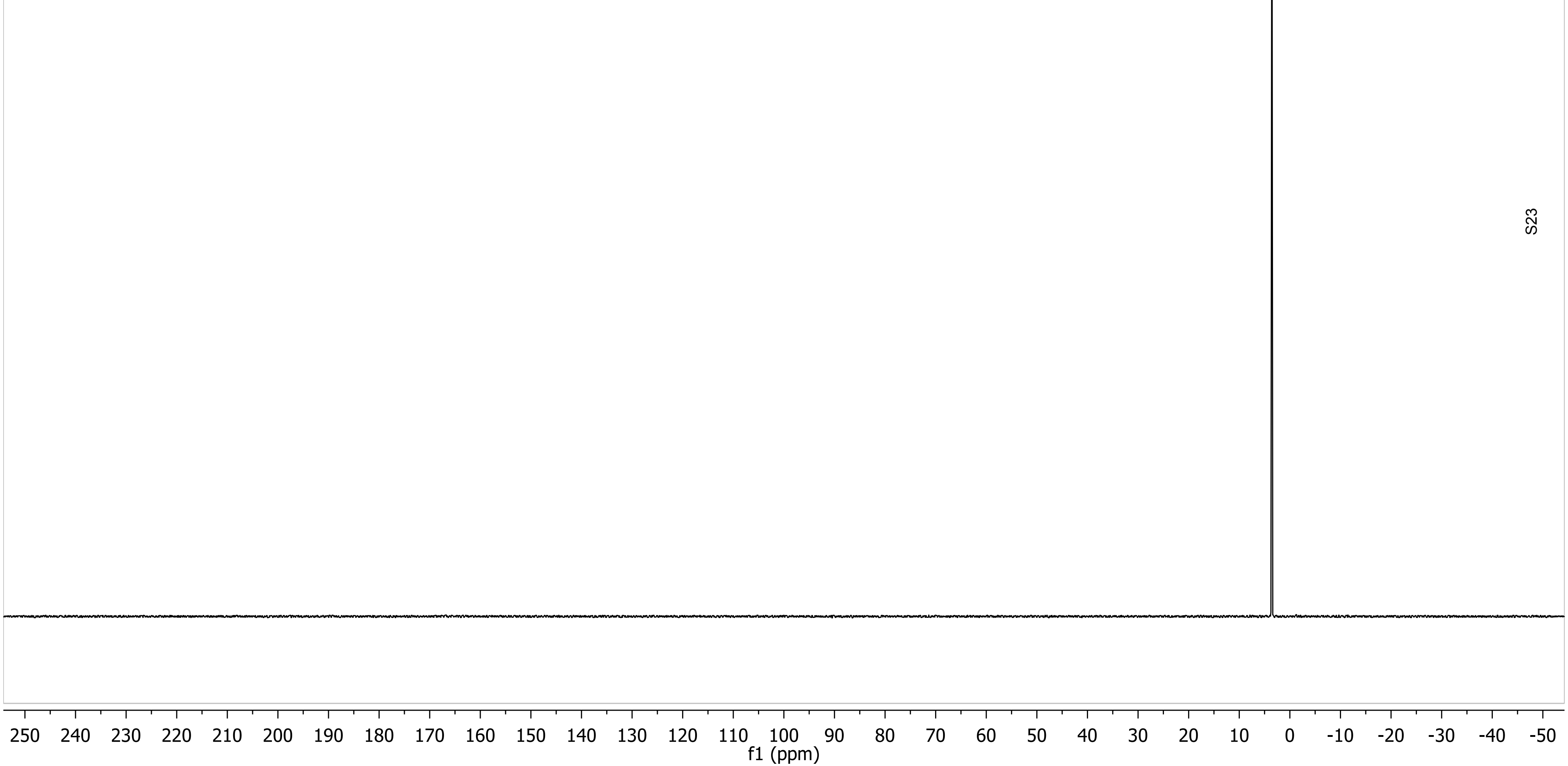

spH1_16green CDCl3 /kwa1g jmccabed 91

0357314-0111-f18

```
Current Data Parameters
NAME     0357314-0111-f18
EXPNO                   1
PROCNO                  1

F2 - Acquisition Parameters
Date_           20150624
Time               11.37
INSTRUM            spect
PROBHD   5 mm PADUL 13C
PULPROG             zg30
TD                 65536
SOLVENT            CDCl3
NS                    16
DS                     0
SWH          10330.578 Hz
FIDRES        0.157632 Hz
AQ           3.1719425 sec
RG                   181
DW              48.400 usec
DE                6.00 usec
TE               294.4 K
D1          1.00000000 sec
TD0                    1

======== CHANNEL f1 ========
NUC1                  1H
P1                  8.00 usec
PL1                -1.00 dB
SFO1         500.2060888 MHz

F2 - Processing parameters
SI                 32768
SF           500.2030000 MHz
WDW                   EM
SSB      0
LB                  0.20 Hz
GB       0
PC                  1.00
```

2e

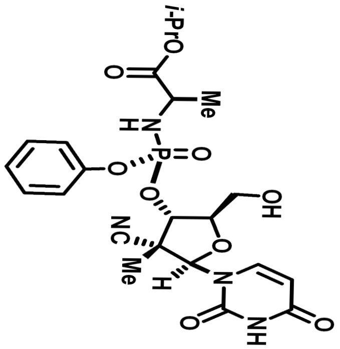

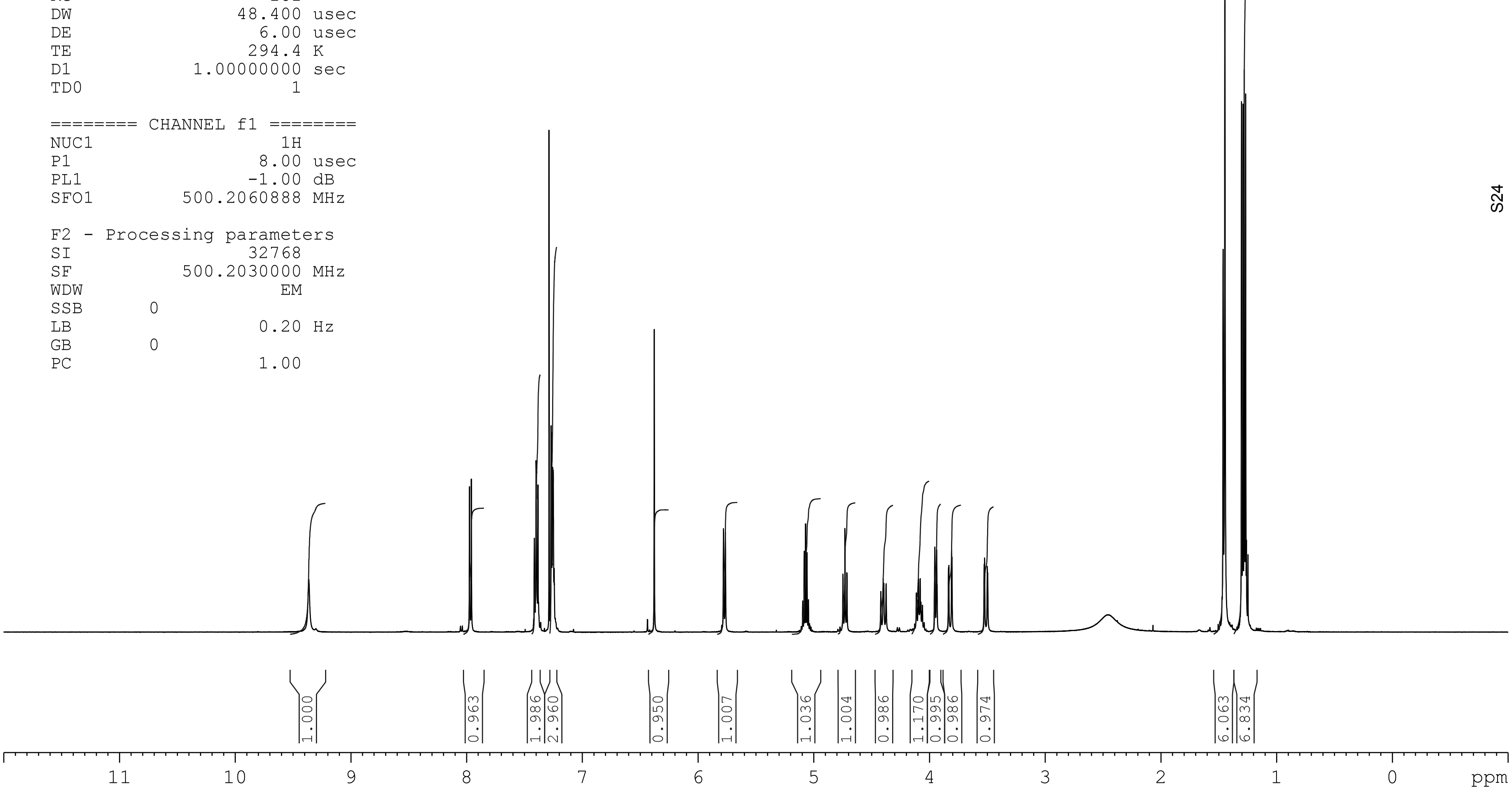

pC13_16k CDCl3 /kwa1g jmccabed 9

```
Current Data Parameters
NAME     0357314-0111-CN_2
EXPNO                    1
PROCNO                   1

F2 - Acquisition Parameters
Date_             20150624
Time                 19.08
INSTRUM              spect
PROBHD   5 mm PADUL 13C
PULPROG            zgpg45
TD                  65536
SOLVENT             CDCl3
NS                  16384
DS                      0
SWH             30303.031 Hz
FIDRES           0.462388 Hz
AQ              1.0813440 sec
RG                 5792.6
DW                 16.500 usec
DE                   6.00 usec
TE                  295.5 K
D1             0.00100000 sec
D11            0.03000000 sec
D12            0.00002000 sec
L2                    128

======== CHANNEL f1 ========
NUC1                  13C
P1                   5.50 usec
PL1                 -2.00 dB
SFO1          125.7899270 MHz

======== CHANNEL f2 ========
CPDPRG[2          waltz16
NUC2                   1H
PCPD2               85.00 usec
PL2                 -1.00 dB
PL12                19.53 dB
PL13                17.00 dB
SFO2          500.2055010 MHz

F2 - Processing parameters
SI                  32768
SF            125.7577390 MHz
WDW                    EM
SSB      0
LB                   1.00 Hz
GB       0
PC                   1.00
```

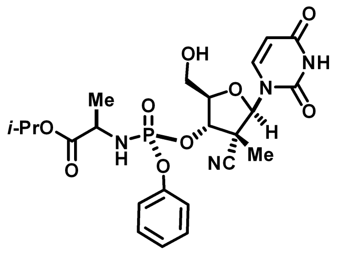

2e

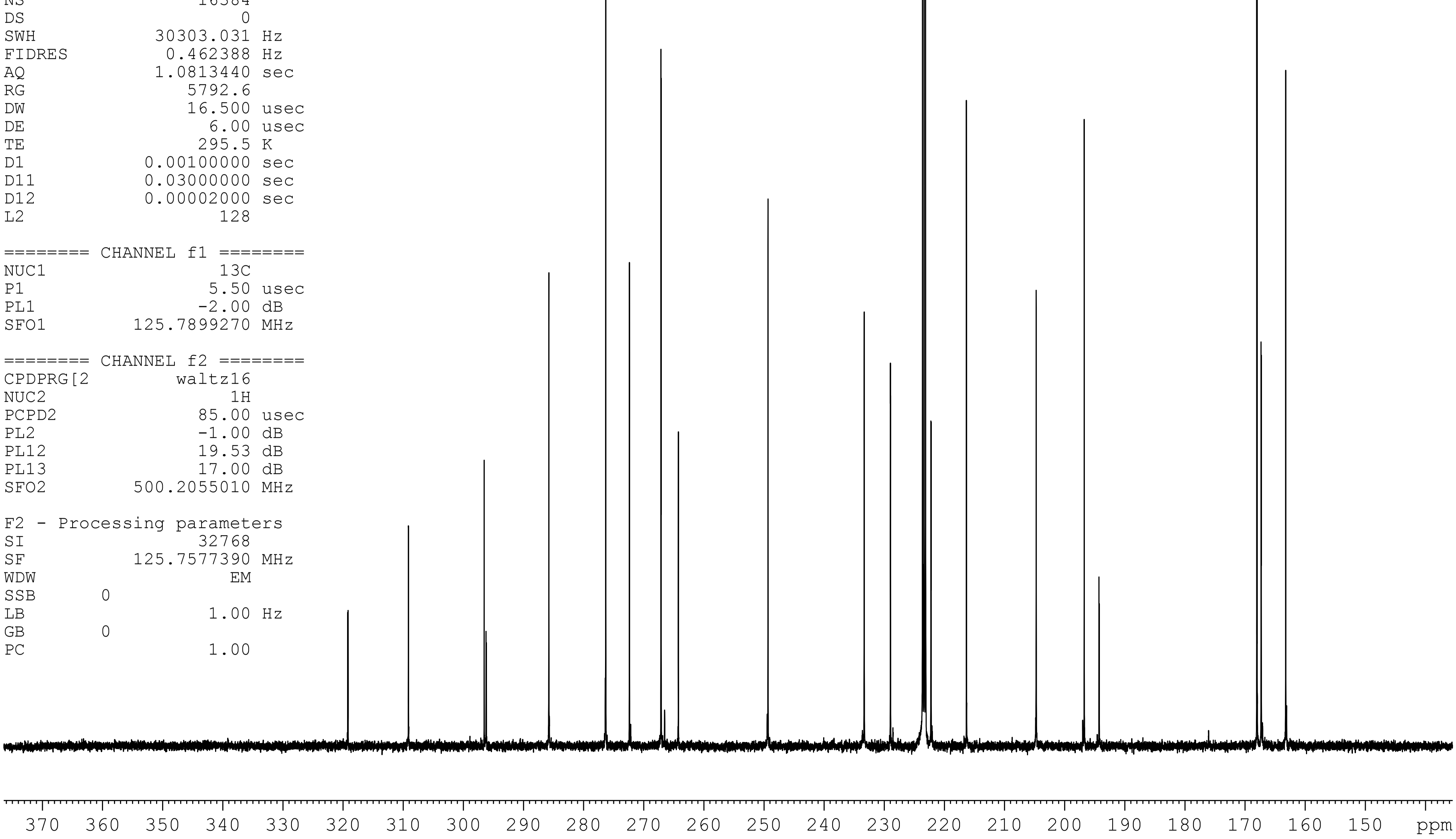

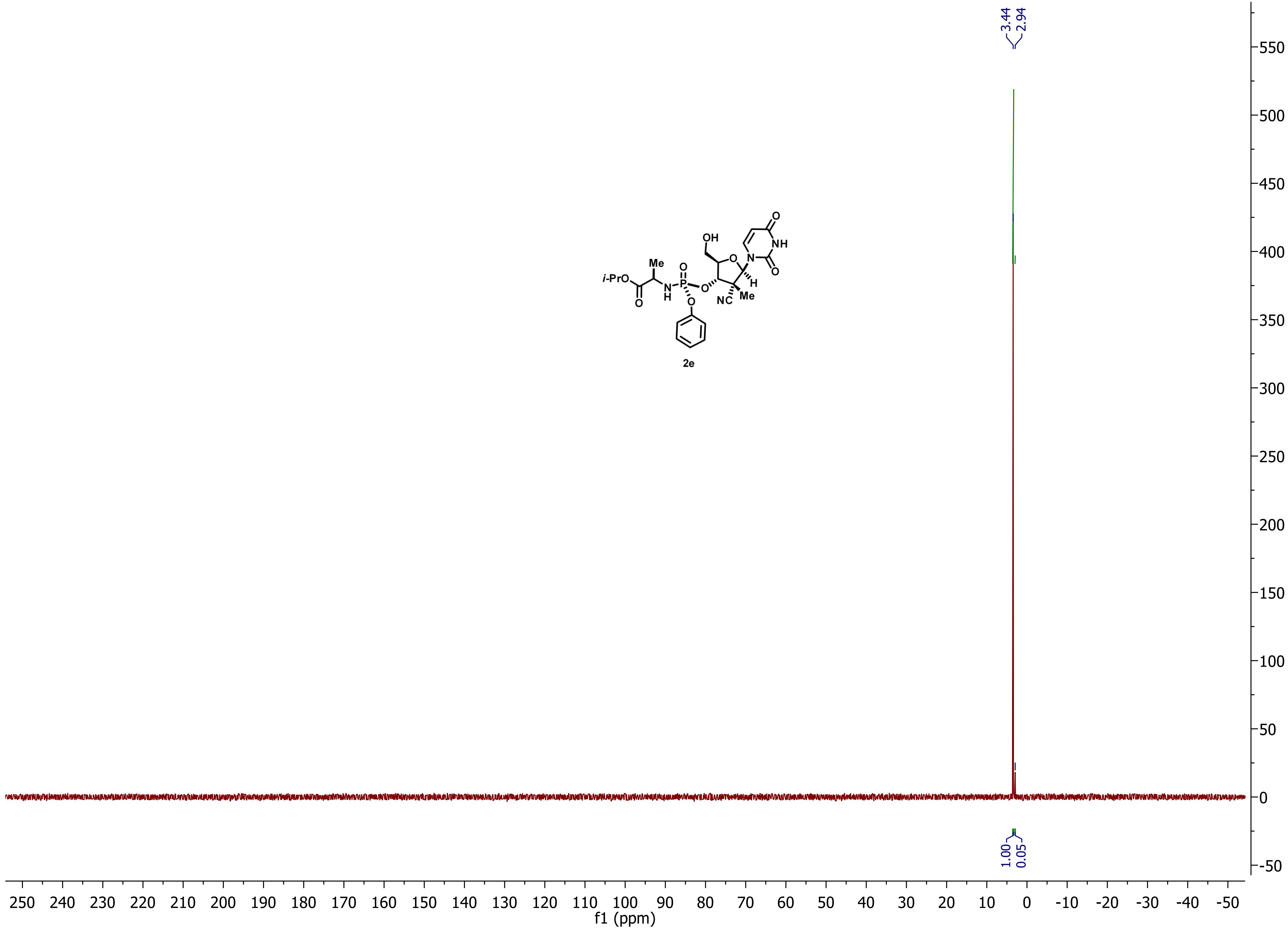
3.44
2.94
1.00
0.05
i-PrO
Me
N
H
P
O
OH
NH
NC
Me
H
2e
f1 (ppm)

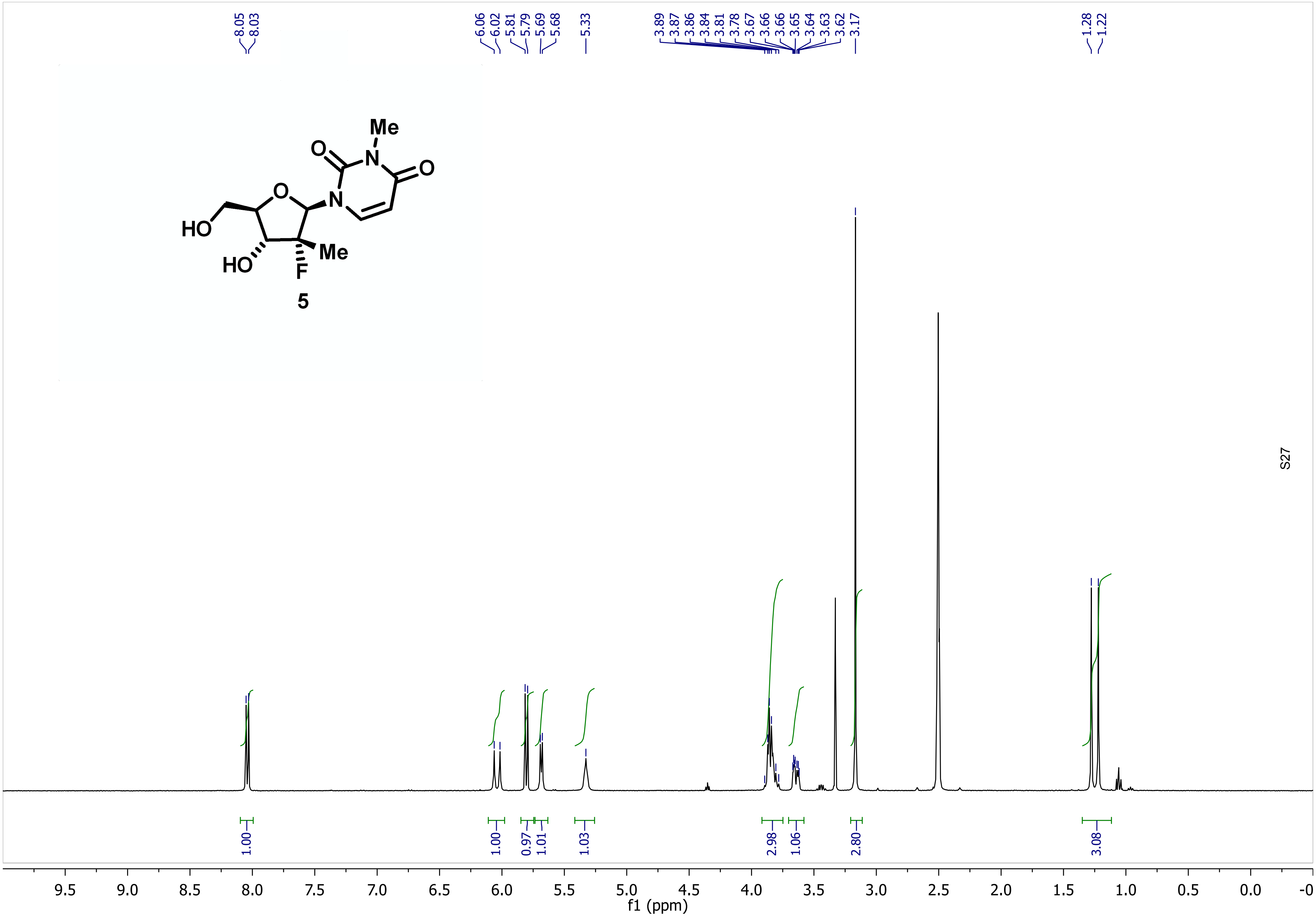

8.05
8.03
6.06
6.02
5.81
5.79
5.69
5.68
5.33
3.89
3.87
3.86
3.84
3.81
3.78
3.67
3.66
3.66
3.65
3.64
3.63
3.62
3.17
1.28
1.22
Me
O
N
O
O
N
HO
HO
Me
F
5
1.00
1.00
0.97
1.01
1.03
2.98
1.06
2.80
3.08
9.5
9.0
8.5
8.0
7.5
7.0
6.5
6.0
5.5
5.0
4.5
4.0
3.5
3.0
2.5
2.0
1.5
1.0
0.5
0.0
-0
f1 (ppm)

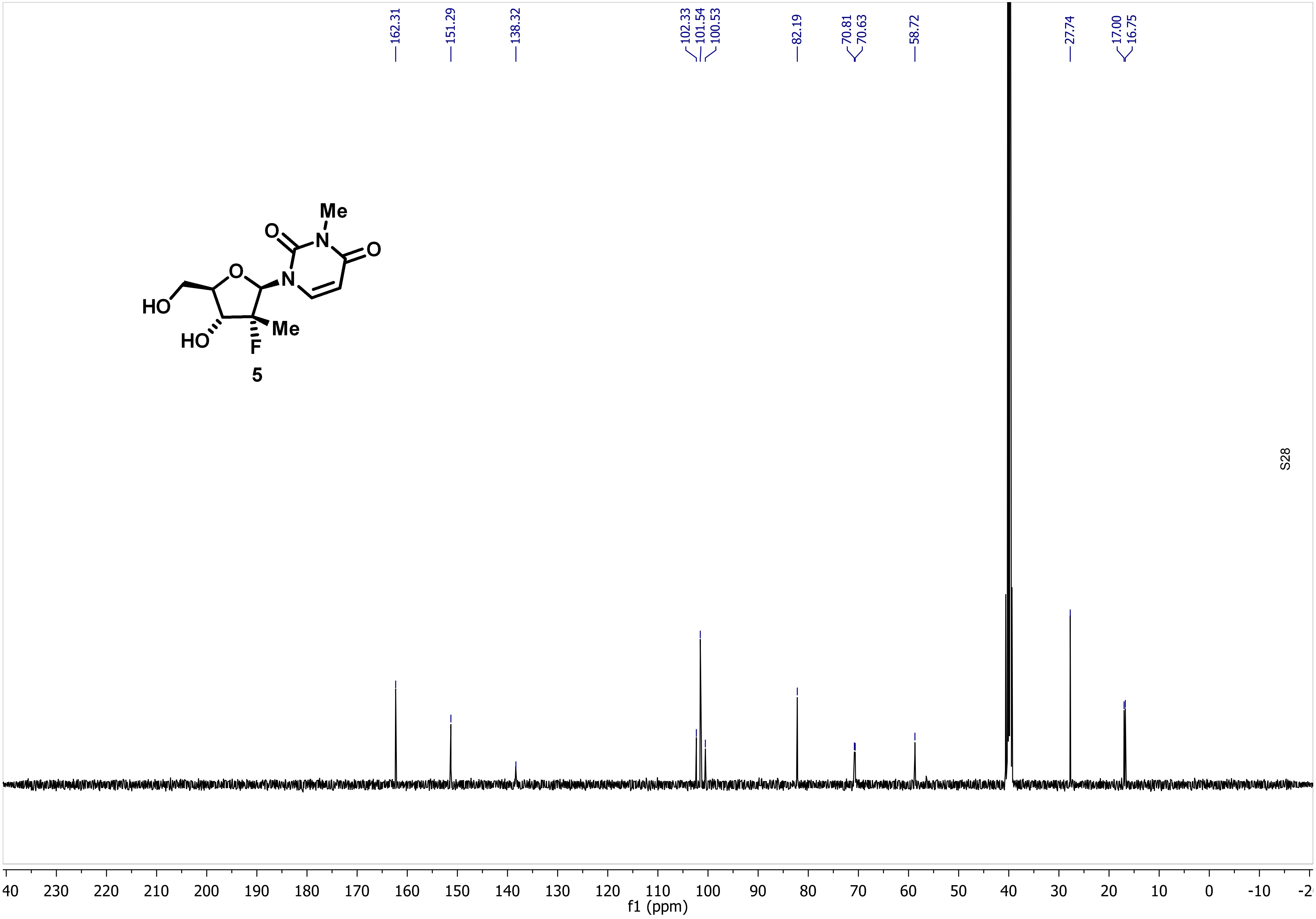

5
162.31
151.29
138.32
102.33
101.54
100.53
82.19
70.81
70.63
58.72
27.74
17.00
16.75
f1 (ppm)

```
Current Data Parameters
NAME     0357314-0195-pdt
EXPNO                   1
PROCNO                  1

F2 - Acquisition Parameters
Date_          20150701
Time               9.02
INSTRUM           spect
PROBHD   5 mm PAQNP 1H/
PULPROG            zg30
TD                65536
SOLVENT           CDCl3
NS                   16
DS                    0
SWH           10000.000 Hz
FIDRES         0.152588 Hz
AQ            3.2767999 sec
RG                   32
DW               50.000 usec
DE                 6.50 usec
TE                294.5 K
D1           1.00000000 sec
TD0                   1

======== CHANNEL f1 ========
SFO1        500.1330885 MHz
NUC1                 1H
P1                10.00 usec
PLW1        39.99399948 W

F2 - Processing parameters
SI                65536
SF          500.1300000 MHz
WDW                  EM
SSB      0
LB                 0.30 Hz
GB       0
PC                 1.00
```

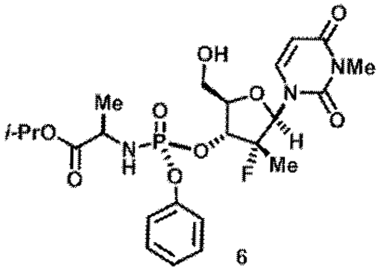

6

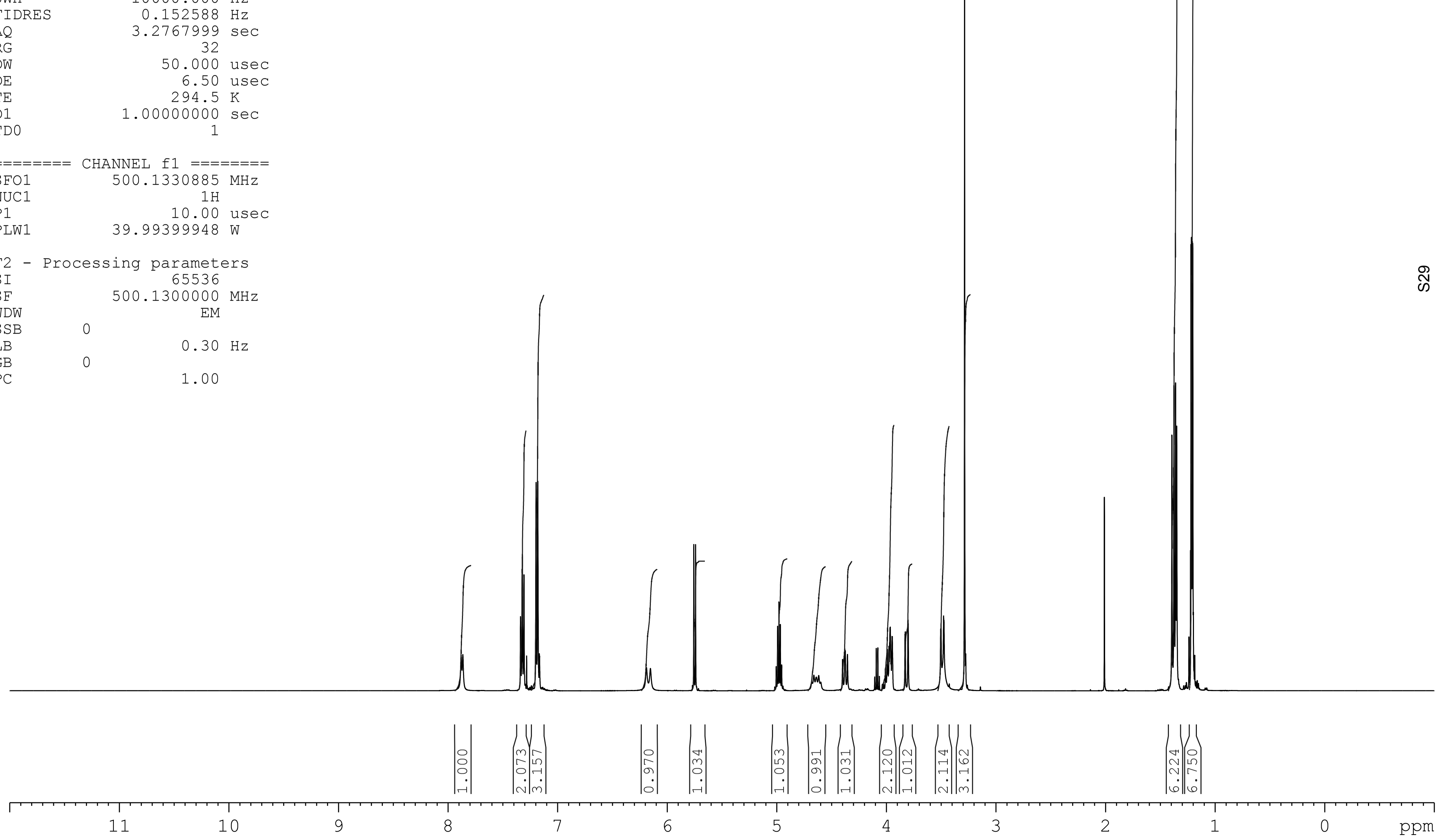

MRL_C13_256 CDCl3 /kwa2i jmccabed 35

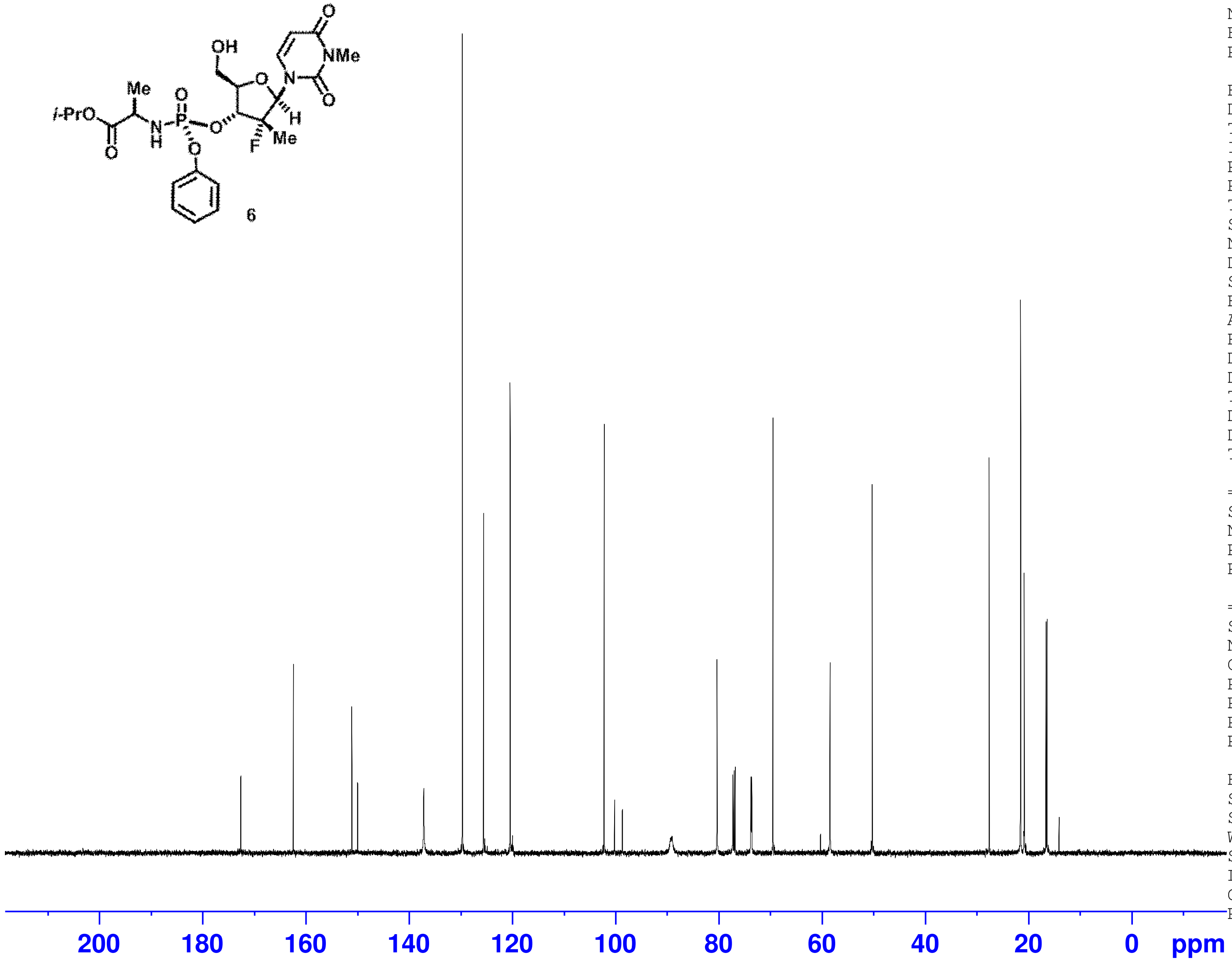


```
Current Data Parameters
NAME      0357314-0195-pdt
EXPNO                    2
PROCNO                   1

F2 - Acquisition Parameters
Date_             20150701
Time                  9.12
INSTRUM              spect
PROBHD    5 mm PAQNP 1H/
PULPROG               zgpg
TD                   65536
SOLVENT              CDCl3
NS                     256
DS                       8
SWH              29761.904 Hz
FIDRES            0.454131 Hz
AQ               1.1010048 sec
RG                     724
DW                  16.800 usec
DE                    6.50 usec
TE                   295.6 K
D1              0.50000000 sec
D11             0.03000000 sec
TD0                      1

======== CHANNEL f1 ========
SFO1           125.7703637 MHz
NUC1                   13C
P1                   13.30 usec
PLW1           29.99200058 W

======== CHANNEL f2 ========
SFO2           500.1320005 MHz
NUC2                    1H
CPDPRG[2           waltz16
PCPD2                80.00 usec
PLW2           30.00000000 W
PLW12           0.67500001 W
PLW13           0.43200001 W

F2 - Processing parameters
SI                   32768
SF             125.7577890 MHz
WDW                     EM
SSB       0
LB                    1.00 Hz
GB        0
PC                    1.40
```

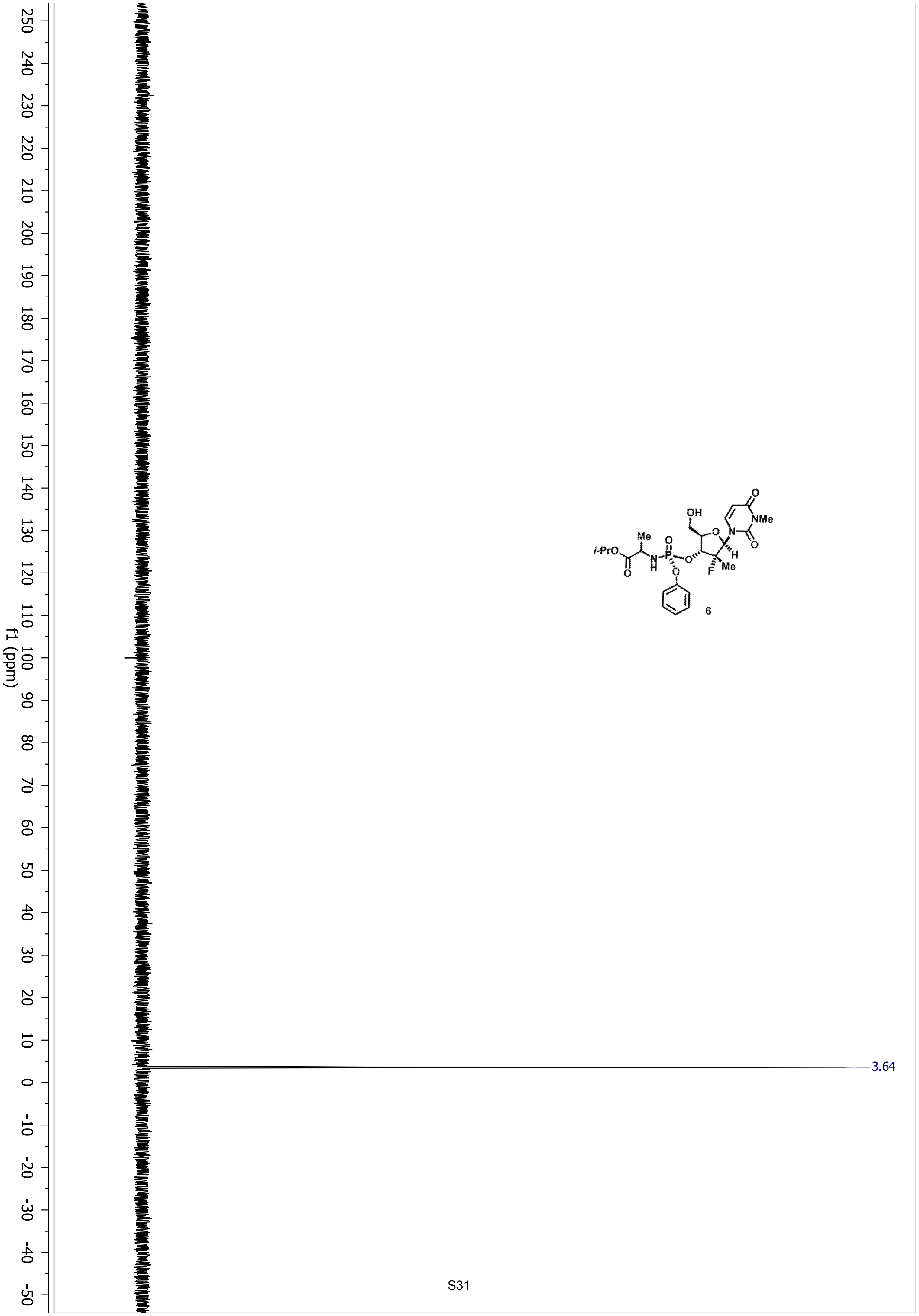

250 240 230 220 210 200 190 180 170 160 150 140 130 120 110 100 90 80 70 60 50 40 30 20 10 0 -10 -20 -30 -40 -50
f1 (ppm)
3.64
i-PrO
Me
NH
P
O
OH
N
NMe
H
F
Me
6

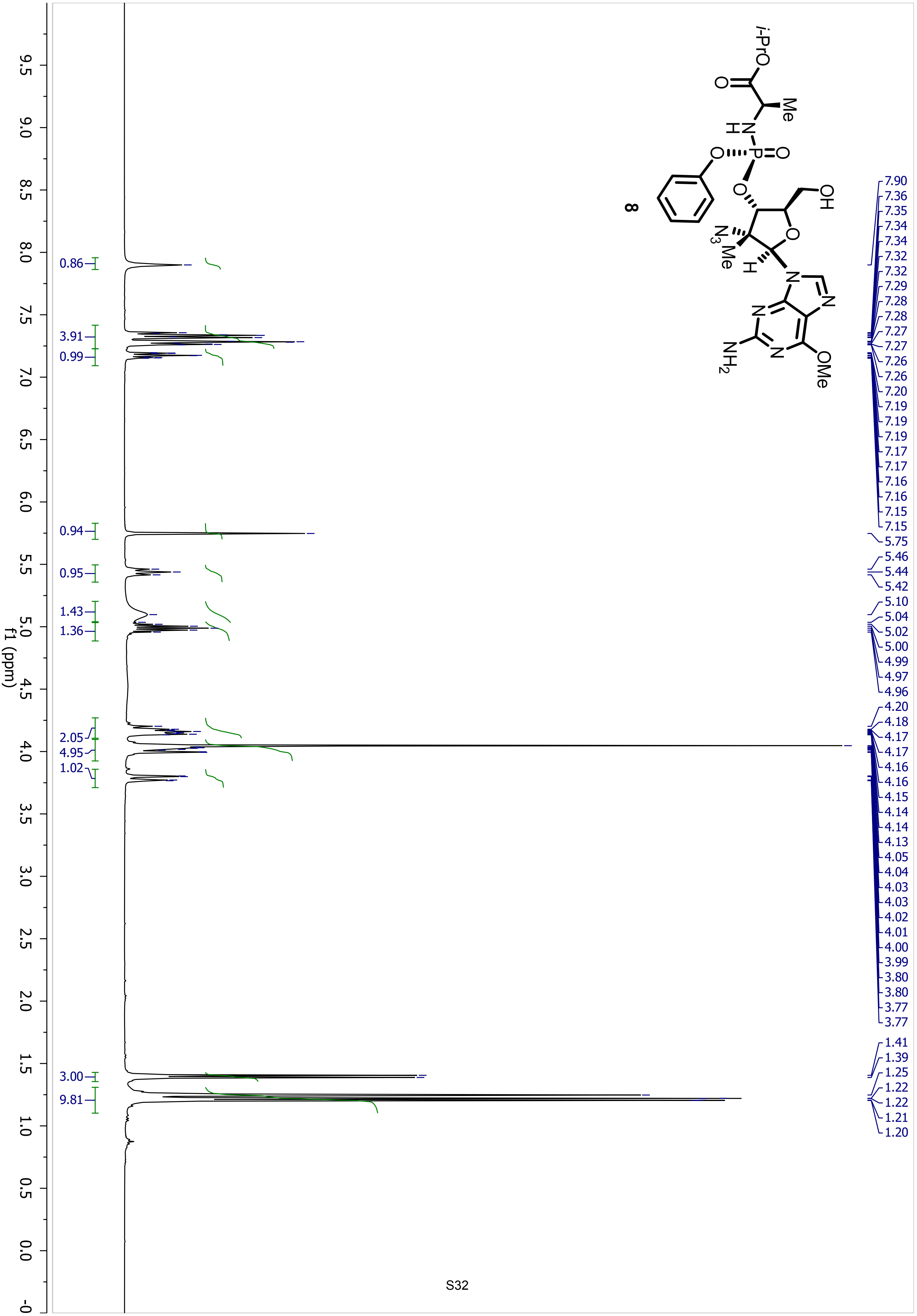
8
f1 (ppm)
7.90
7.36
7.35
7.34
7.34
7.32
7.32
7.29
7.28
7.28
7.27
7.27
7.26
7.26
7.20
7.19
7.19
7.19
7.17
7.17
7.16
7.16
7.15
7.15
5.75
5.46
5.44
5.42
5.10
5.04
5.02
5.00
4.99
4.97
4.96
4.20
4.18
4.17
4.17
4.16
4.16
4.15
4.14
4.14
4.13
4.05
4.04
4.03
4.03
4.02
4.01
4.00
3.99
3.80
3.80
3.77
3.77
1.41
1.39
1.25
1.22
1.22
1.21
1.20
0.86
3.91
0.99
0.94
0.95
1.43
1.36
2.05
4.95
1.02
3.00
9.81

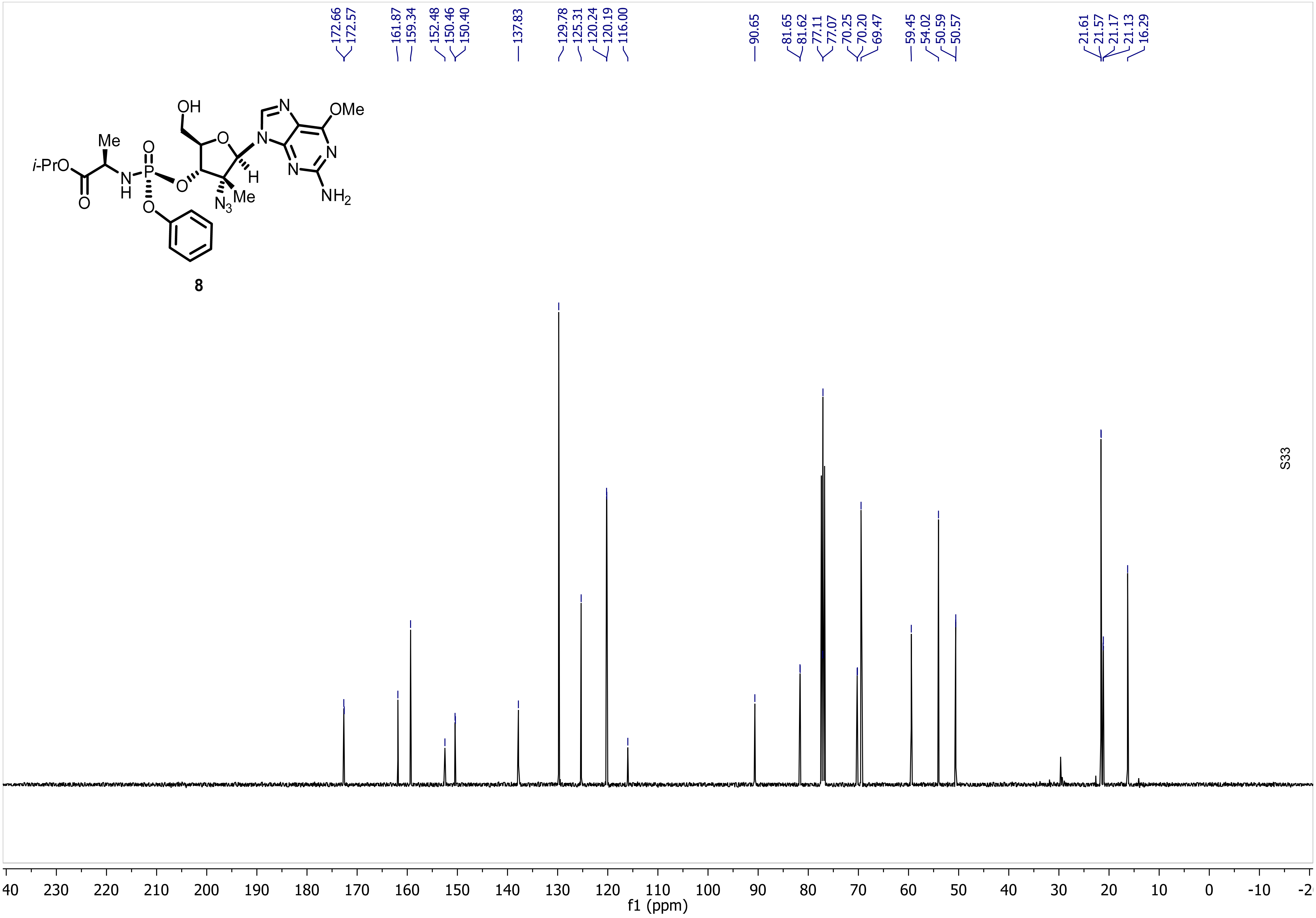

i-PrO
Me
N
H
P
O
O
OH
O
N
N
OMe
N
N
NH2
H
N3
Me
172.66
172.57
161.87
159.34
152.48
150.46
150.40
137.83
129.78
125.31
120.24
120.19
116.00
90.65
81.65
81.62
77.11
77.07
70.25
70.20
69.47
59.45
54.02
50.59
50.57
21.61
21.57
21.17
21.13
16.29
f1 (ppm)

8

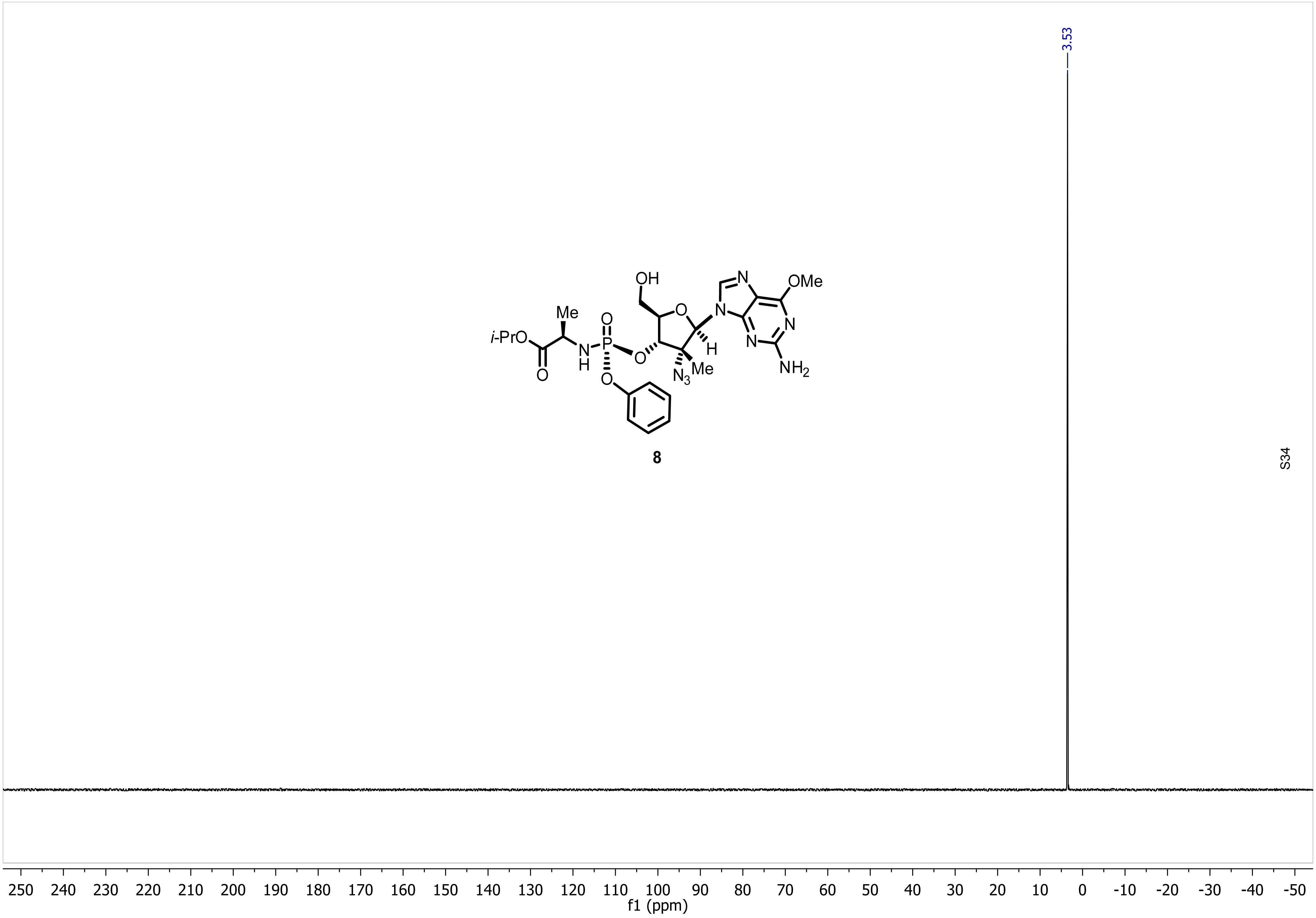

3.53
i-PrO
Me
O
NH
P
OH
N
OMe
N3
NH2
8
250 240 230 220 210 200 190 180 170 160 150 140 130 120 110 100 90 80 70 60 50 40 30 20 10 0 -10 -20 -30 -40 -50
f1 (ppm)

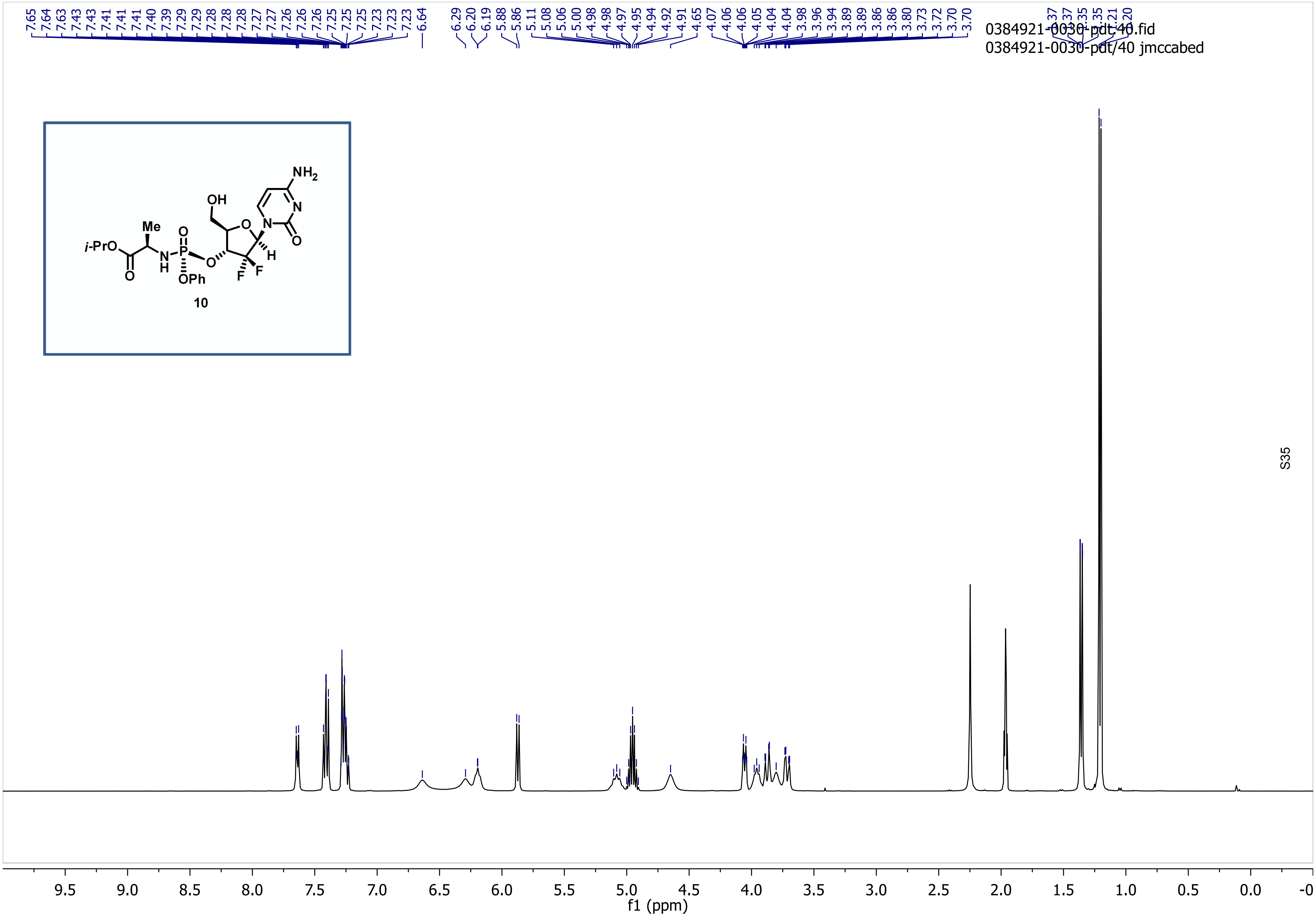
7.65 7.64 7.63 7.43 7.43 7.41 7.41 7.41 7.40 7.39 7.29 7.29 7.28 7.28 7.28 7.27 7.27 7.26 7.26 7.26 7.25 7.25 7.25 7.23 7.23 7.23 6.64 6.29 6.20 6.19 5.88 5.86 5.11 5.08 5.06 5.00 4.98 4.98 4.97 4.95 4.94 4.92 4.91 4.65 4.07 4.06 4.06 4.05 4.04 4.04 3.98 3.96 3.94 3.89 3.89 3.86 3.86 3.80 3.73 3.72 3.70 3.70
1.37 1.37 1.35 1.35 1.21 1.20
0384921-0030-pdt/40.fid
0384921-0030-pdt/40 jmccabed
NH2
OH
Me
i-PrO
OPh
10
9.5 9.0 8.5 8.0 7.5 7.0 6.5 6.0 5.5 5.0 4.5 4.0 3.5 3.0 2.5 2.0 1.5 1.0 0.5 0.0 -0
f1 (ppm)

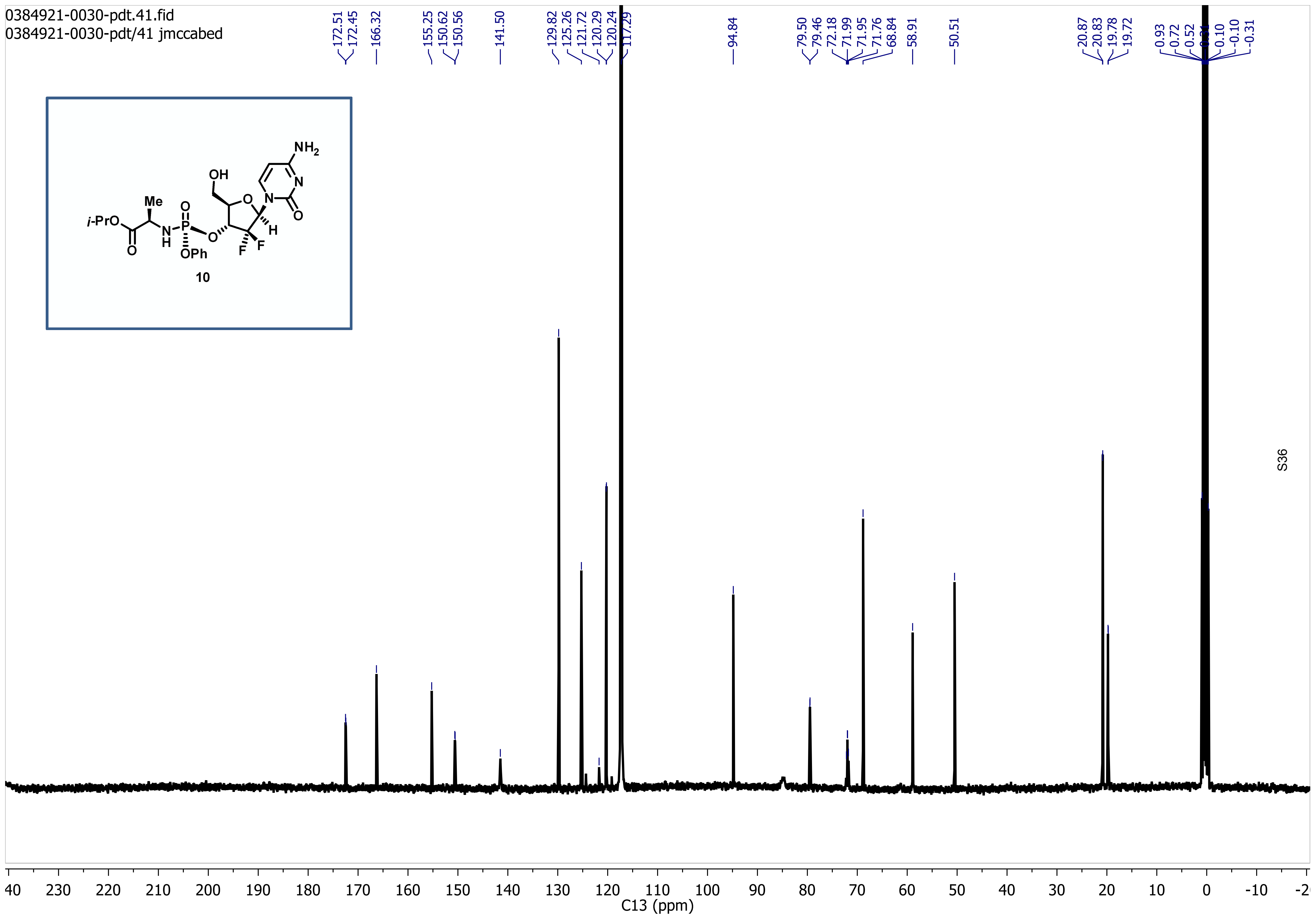
0384921-0030-pdt.41.fid
0384921-0030-pdt/41 jmccabed
172.51
172.45
166.32
155.25
150.62
150.56
141.50
129.82
125.26
121.72
120.29
120.24
117.29
94.84
79.50
79.46
72.18
71.99
71.95
71.76
68.84
58.91
50.51
20.87
20.83
19.78
19.72
0.93
0.72
0.52
0.10
-0.10
-0.31
i-PrO
Me
NH
O
P
OPh
OH
N
NH2
F
H
10
C13 (ppm)

^{31}P NMR (203 MHz, Acetonitrile-d_3) δ 2.94.

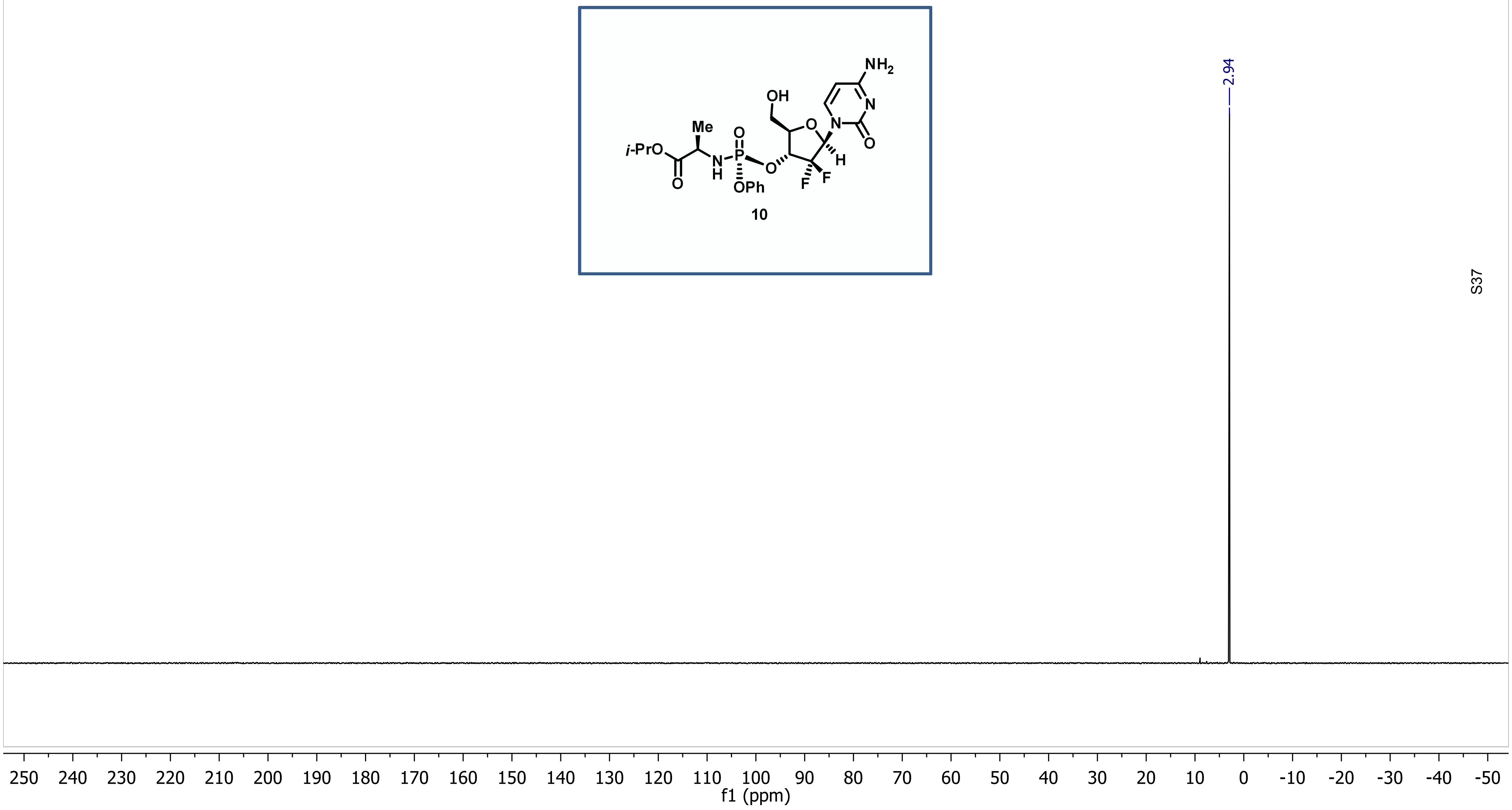

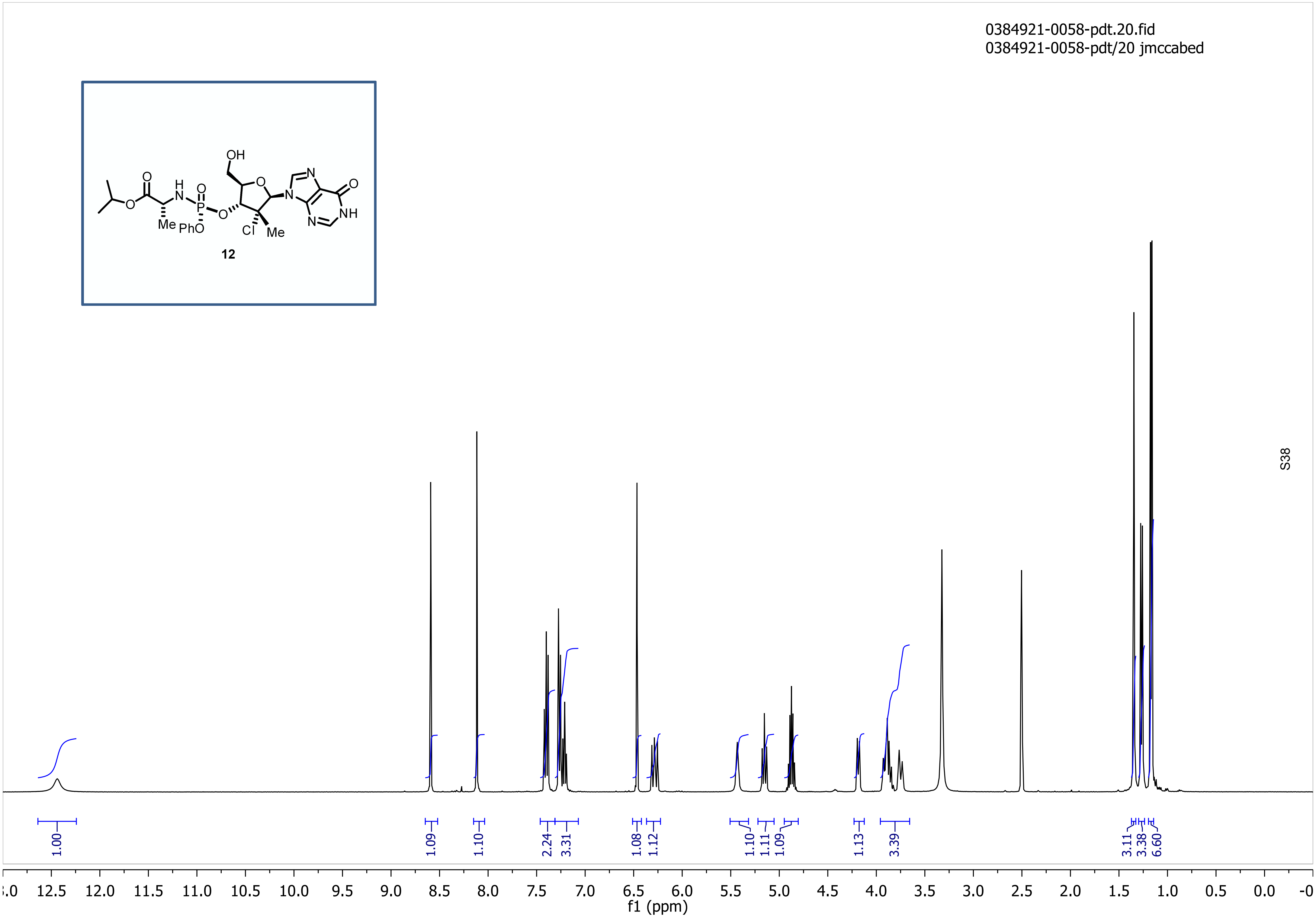

0384921-0058-pdt.20.fid
0384921-0058-pdt/20 jmccabed
12
1.00
1.09
1.10
2.24
3.31
1.08
1.12
1.10
1.11
1.09
1.13
3.39
3.11
3.38
6.60
12.5 12.0 11.5 11.0 10.5 10.0 9.5 9.0 8.5 8.0 7.5 7.0 6.5 6.0 5.5 5.0 4.5 4.0 3.5 3.0 2.5 2.0 1.5 1.0 0.5 0.0
f1 (ppm)

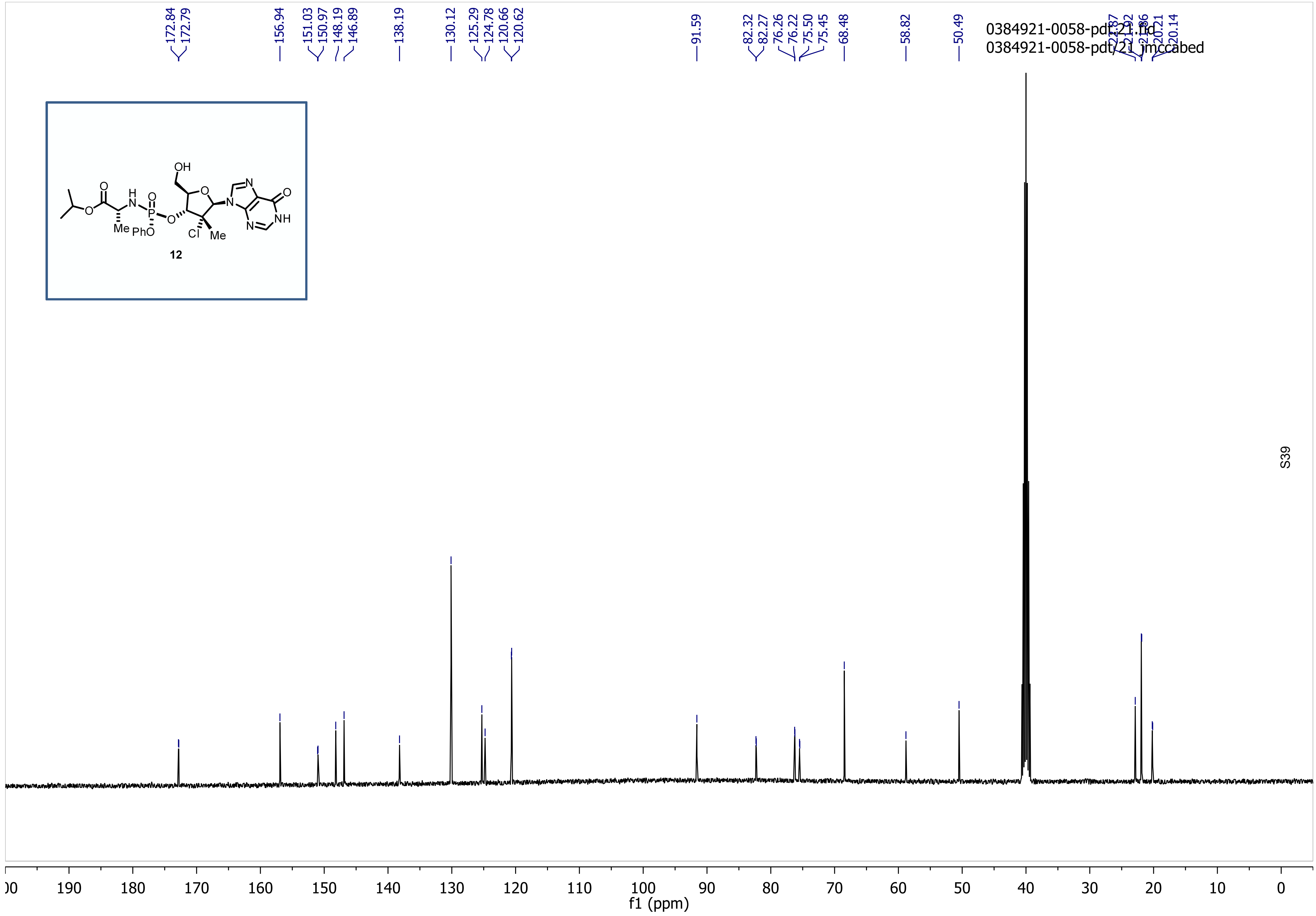
172.84
172.79
156.94
151.03
150.97
148.19
146.89
138.19
130.12
125.29
124.78
120.66
120.62
91.59
82.32
82.27
76.26
76.22
75.50
75.45
68.48
58.82
50.49
20.21
20.14
0384921-0058-pdt/21.fid
0384921-0058-pdt/21_jmccabed
12
f1 (ppm)

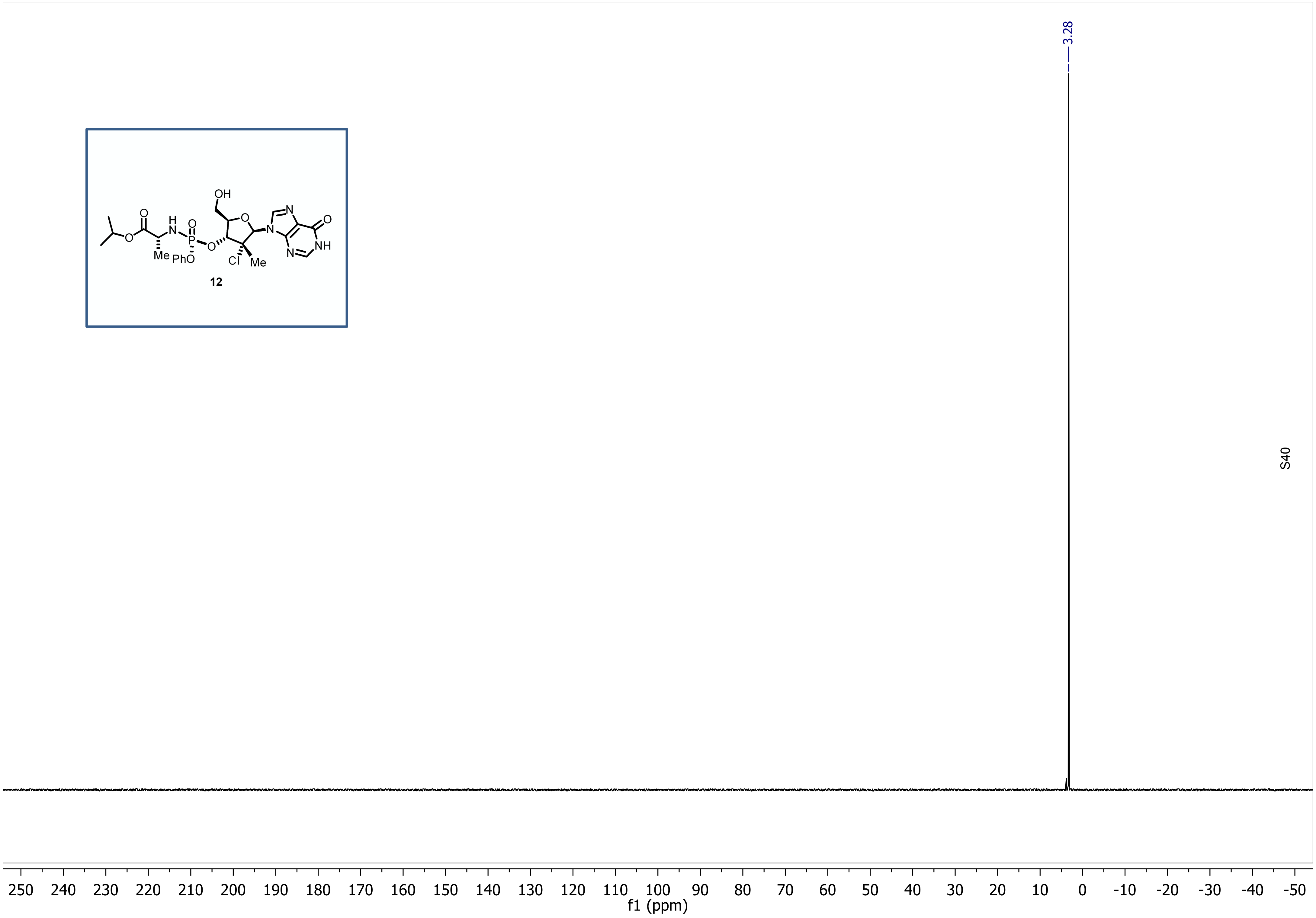
12
3.28
250 240 230 220 210 200 190 180 170 160 150 140 130 120 110 100 90 80 70 60 50 40 30 20 10 0 -10 -20 -30 -40 -50
f1 (ppm)

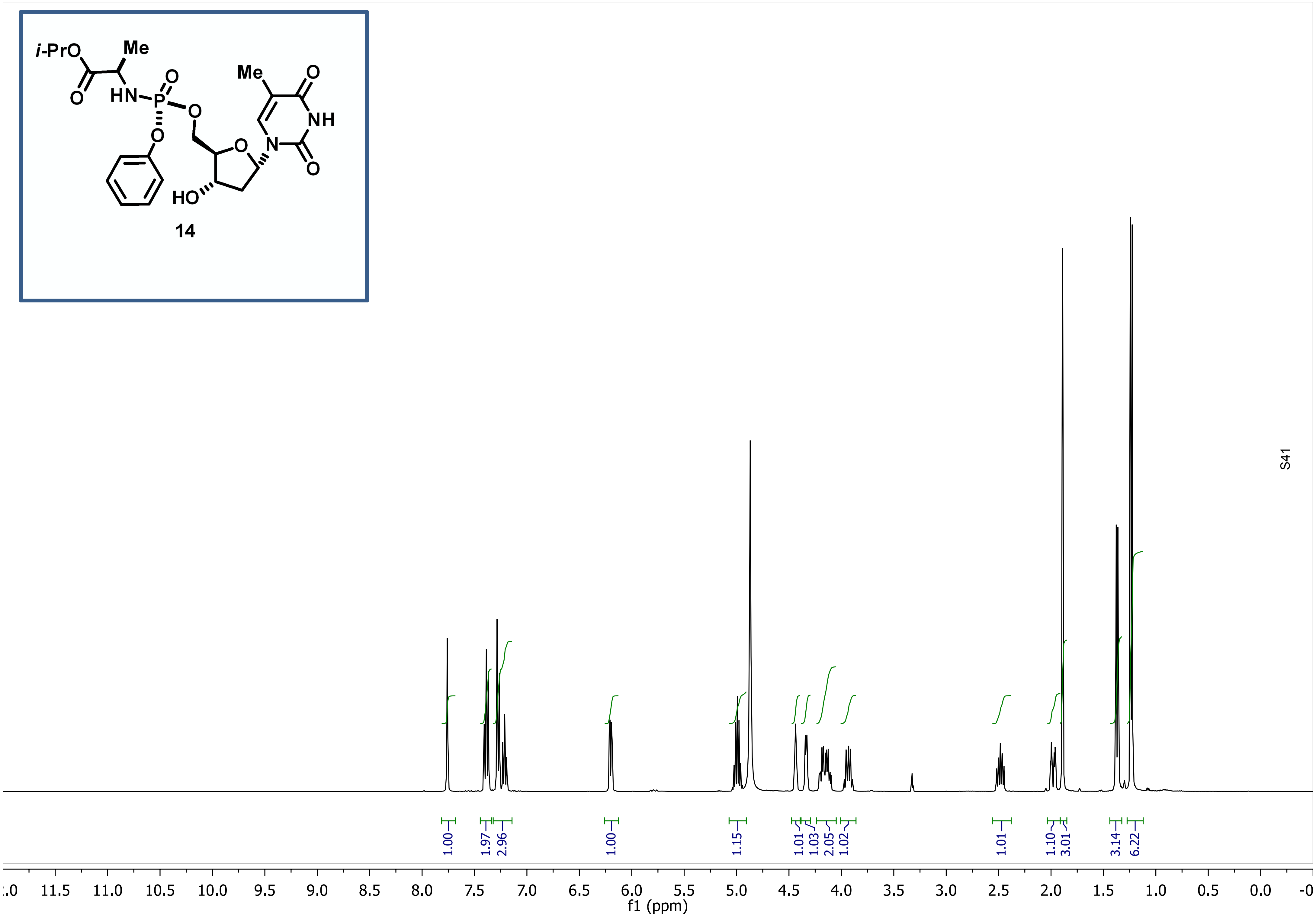
i-PrO
Me
O
HN
P
O
O
O
Me
O
NH
N
O
O
HO
14
1.00
1.97
2.96
1.00
1.15
1.01
1.03
2.05
1.02
1.01
1.10
3.01
3.14
6.22
f1 (ppm)

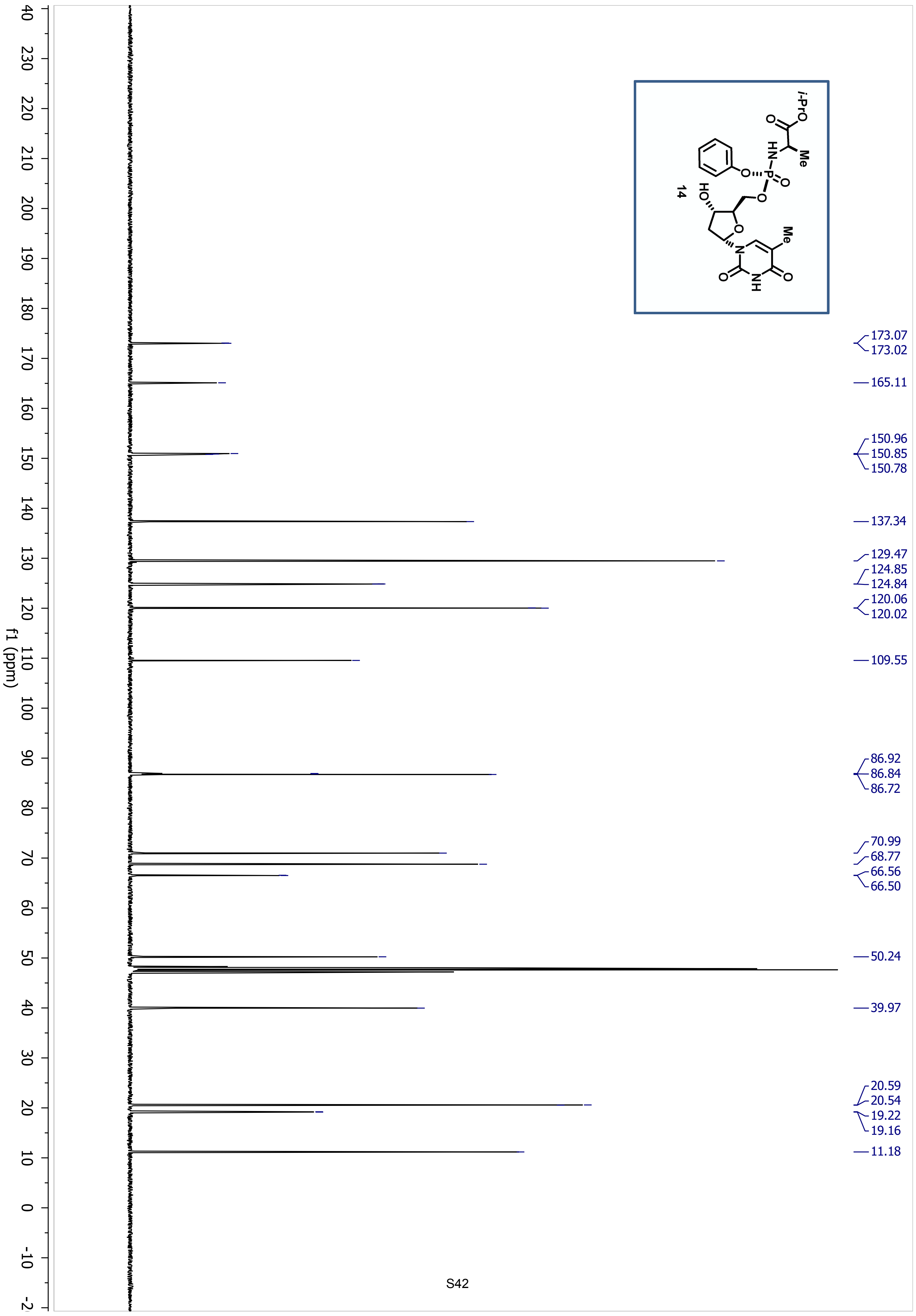

14
i-PrO
HN
Me
P
O
HO
N
NH
173.07
173.02
165.11
150.96
150.85
150.78
137.34
129.47
124.85
124.84
120.06
120.02
109.55
86.92
86.84
86.72
70.99
68.77
66.56
66.50
50.24
39.97
20.59
20.54
19.22
19.16
11.18
f1 (ppm)

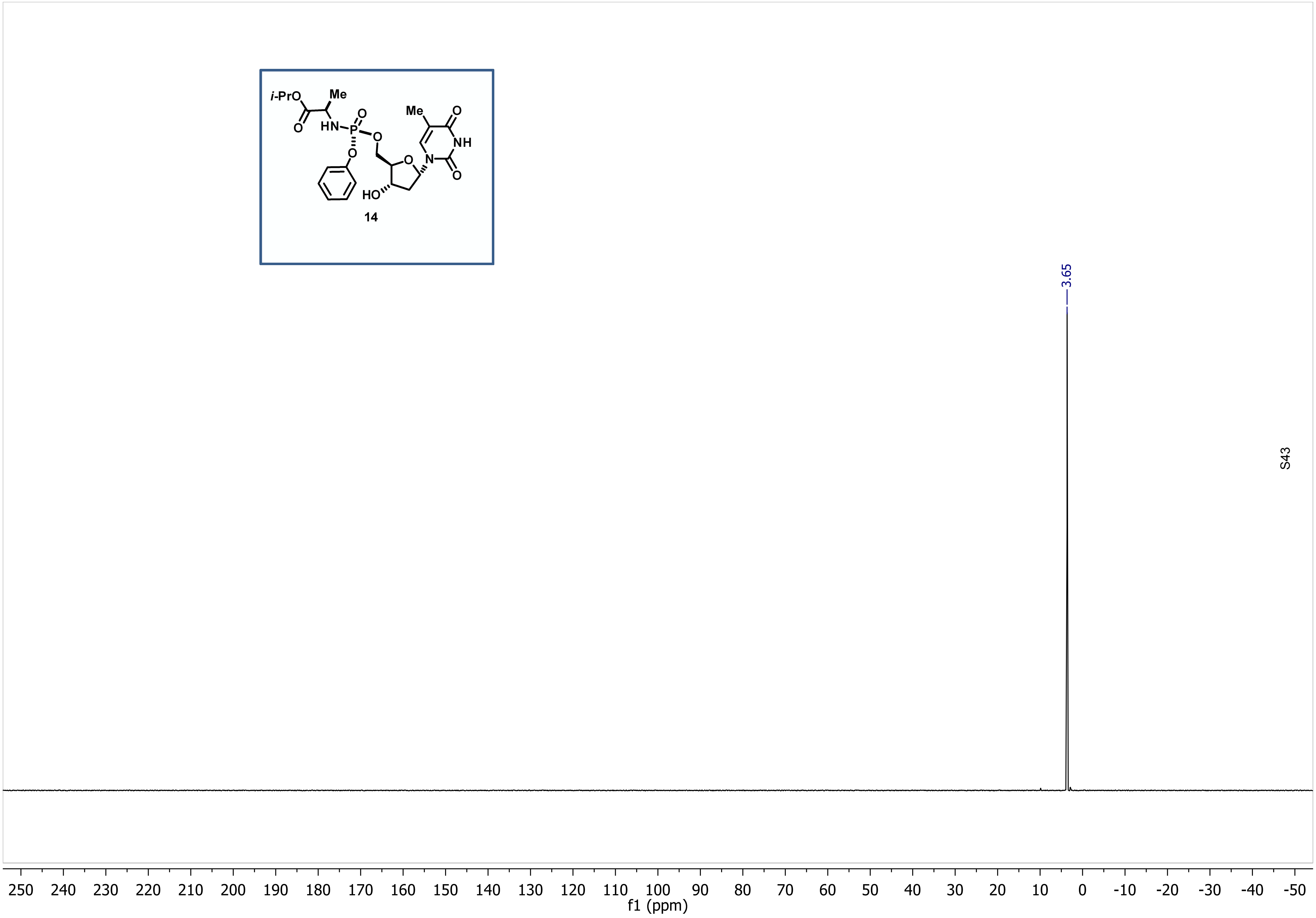

i-PrO
Me
HN
P
O
NH
N
HO
14
3.65
250 240 230 220 210 200 190 180 170 160 150 140 130 120 110 100 90 80 70 60 50 40 30 20 10 0 -10 -20 -30 -40 -50
f1 (ppm)

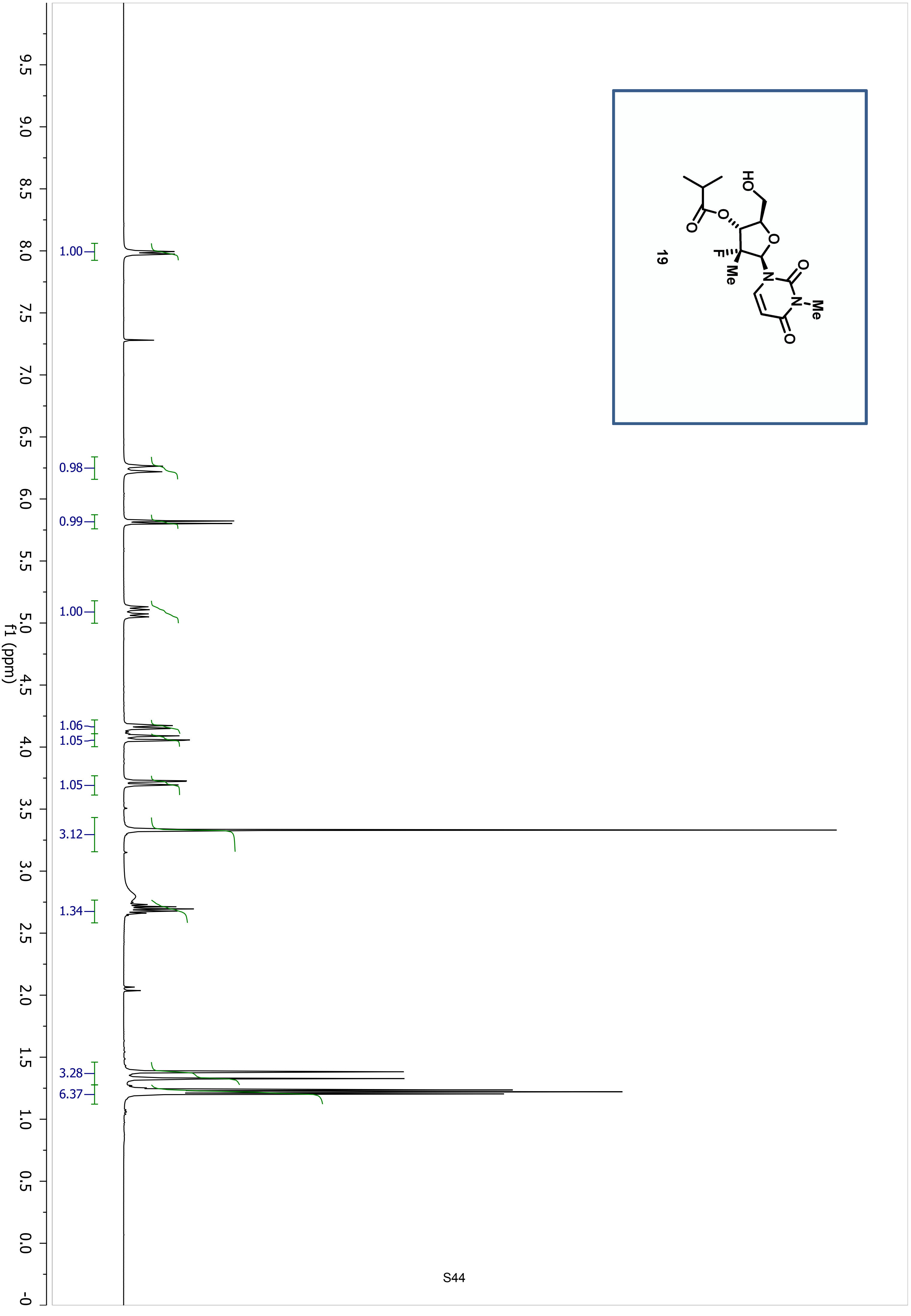

19
HO
O
F
Me
N
O
N
Me
O
O
O
f1 (ppm)
1.00
0.98
0.99
1.00
1.06
1.05
1.05
3.12
1.34
3.28
6.37

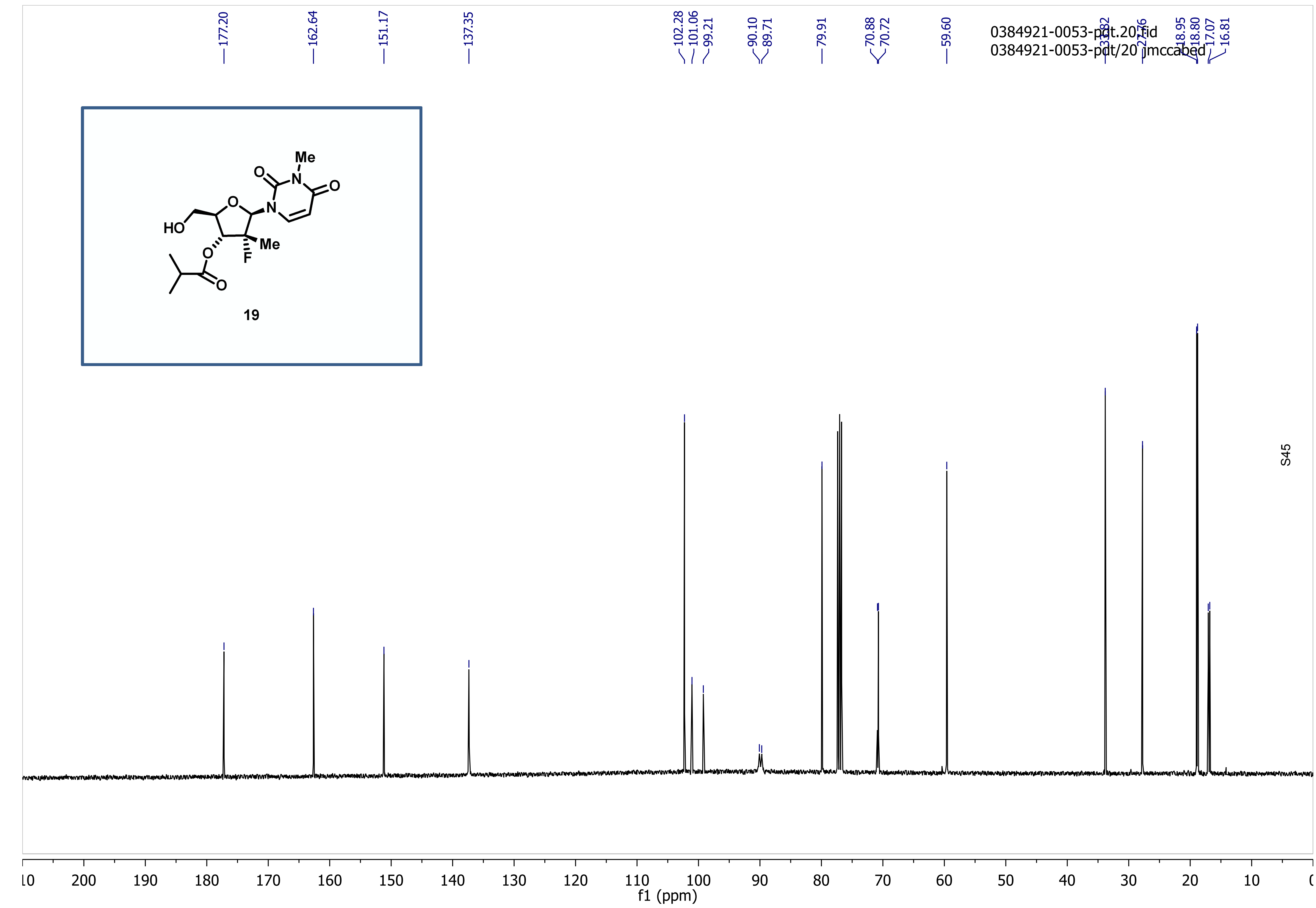

Me
O
N
O
O
N
HO
O
Me
F
O
19
177.20
162.64
151.17
137.35
102.28
101.06
99.21
90.10
89.71
79.91
70.88
70.72
59.60
33.82
27.76
18.95
18.80
17.07
16.81
0384921-0053-pdt.20.fid
0384921-0053-pdt/20 jmccabed
f1 (ppm)

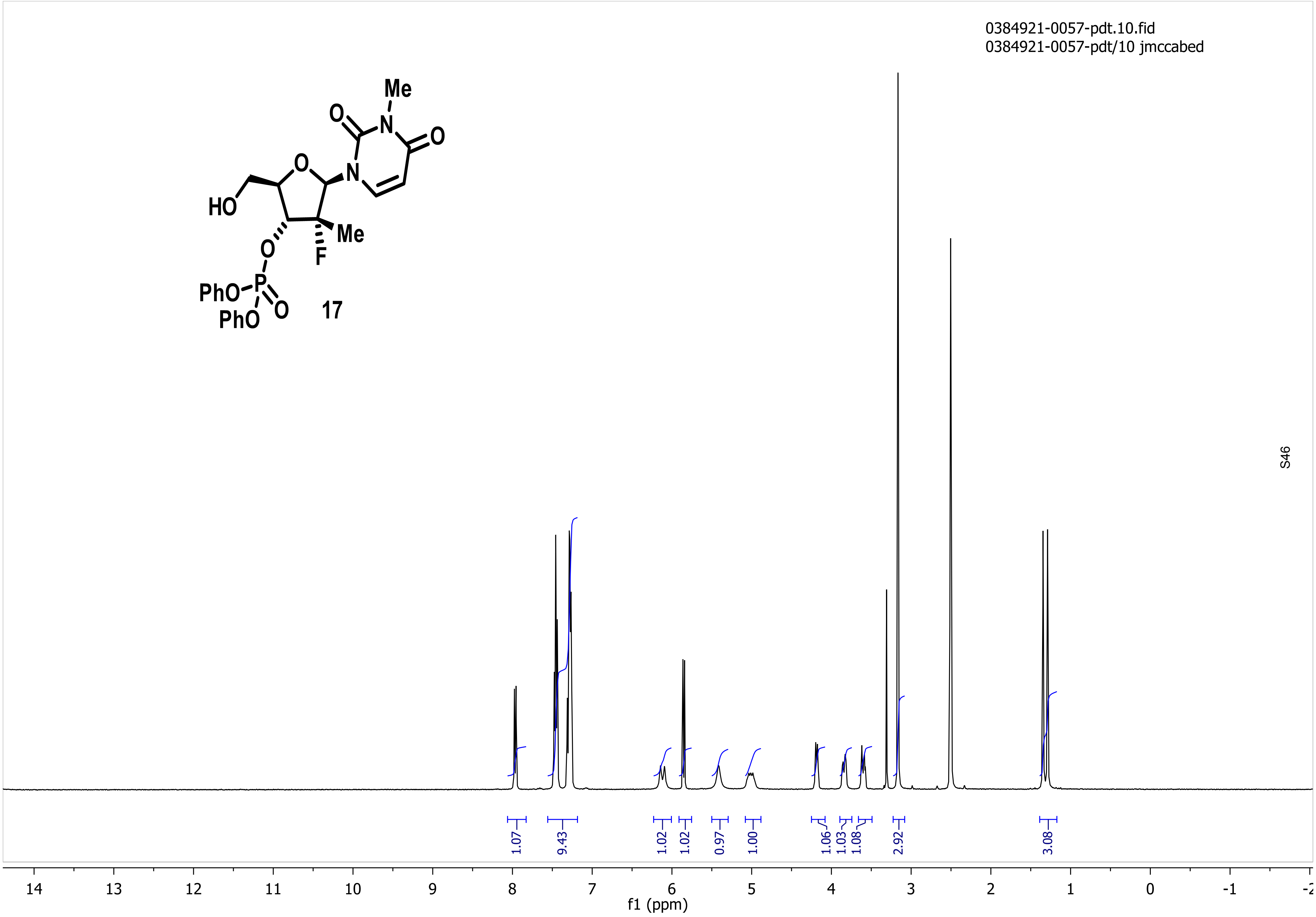
0384921-0057-pdt.10.fid
0384921-0057-pdt/10 jmccabed
Me
O
N
O
O
N
HO
Me
O
F
PhO
P
O
PhO
1.07
9.43
1.02
1.02
0.97
1.00
1.06
1.03
1.08
2.92
3.08
14
13
12
11
10
9
8
7
6
5
4
3
2
1
0
-1
f1 (ppm)

17

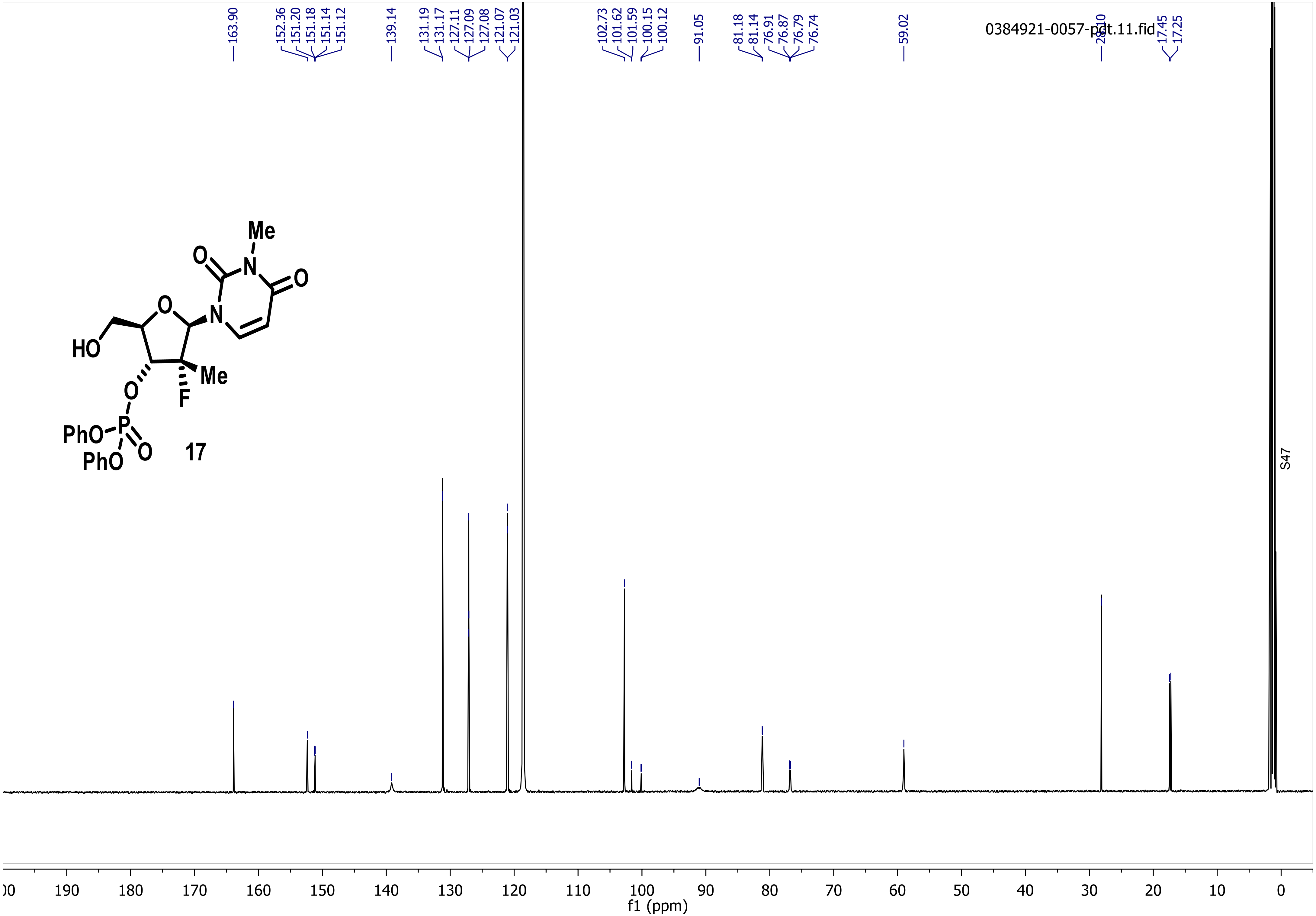

163.90
152.36
151.20
151.18
151.14
151.12
139.14
131.19
131.17
127.11
127.09
127.08
121.07
121.03
102.73
101.62
101.59
100.15
100.12
91.05
81.18
81.14
76.91
76.87
76.79
76.74
59.02
17.45
17.25
0384921-0057-pdt.11.fid
HO
O
N
O
N
Me
O
Me
F
O
PhO
P
O
PhO
17
f1 (ppm)

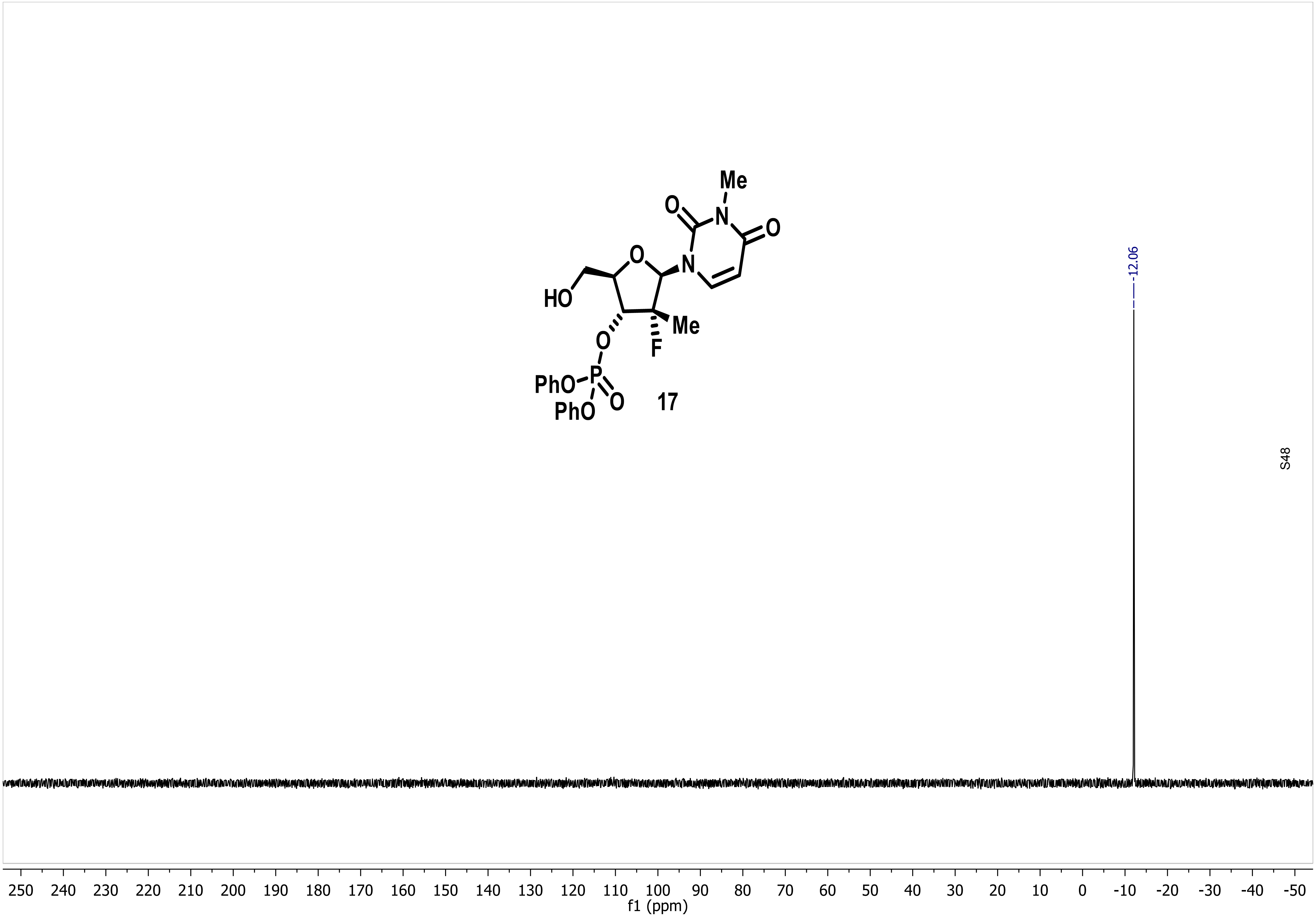
-12.06
HO
O
N
O
N
Me
O
Me
F
O
PhO
P
O
PhO
17
250 240 230 220 210 200 190 180 170 160 150 140 130 120 110 100 90 80 70 60 50 40 30 20 10 0 -10 -20 -30 -40 -50
f1 (ppm)

Crystal Data and Structure Refinement for Compound 4 (CCDC 1525737)

A single crystal grown from isopropanol and water by solvent evaporation was selected for single crystal X-ray data analysis. The crystal was a small colorless rod with dimensions of 0.12 mm x 0.02 mm x 0.02mm. Data collection was performed on a Bruker Apex II system at 100K. The unit cell was determined to be triclinic in space group P1 and the structure contained two molecules of compound **4** in the asymmetric unit. Crystallographic data is summarized in Table 1. Absolute configuration was determined by anomalous-dispersion effects in diffraction measurements on the crystal and confirmed that the stereochemistry at both of the stereogenic centres was *R*. Figure 1 shows a thermal ellipsoid representation of Compound **4** with thermal ellipsoids set at the 50% probability level. Coordinates, refinement details and structure factors have been deposited with the Cambridge Crystallographic Data Centre (CCDC 1525737).

Figure 1: Thermal ellipsoid representation of Compound **4** with thermal ellipsoids set at the 50% probability level.

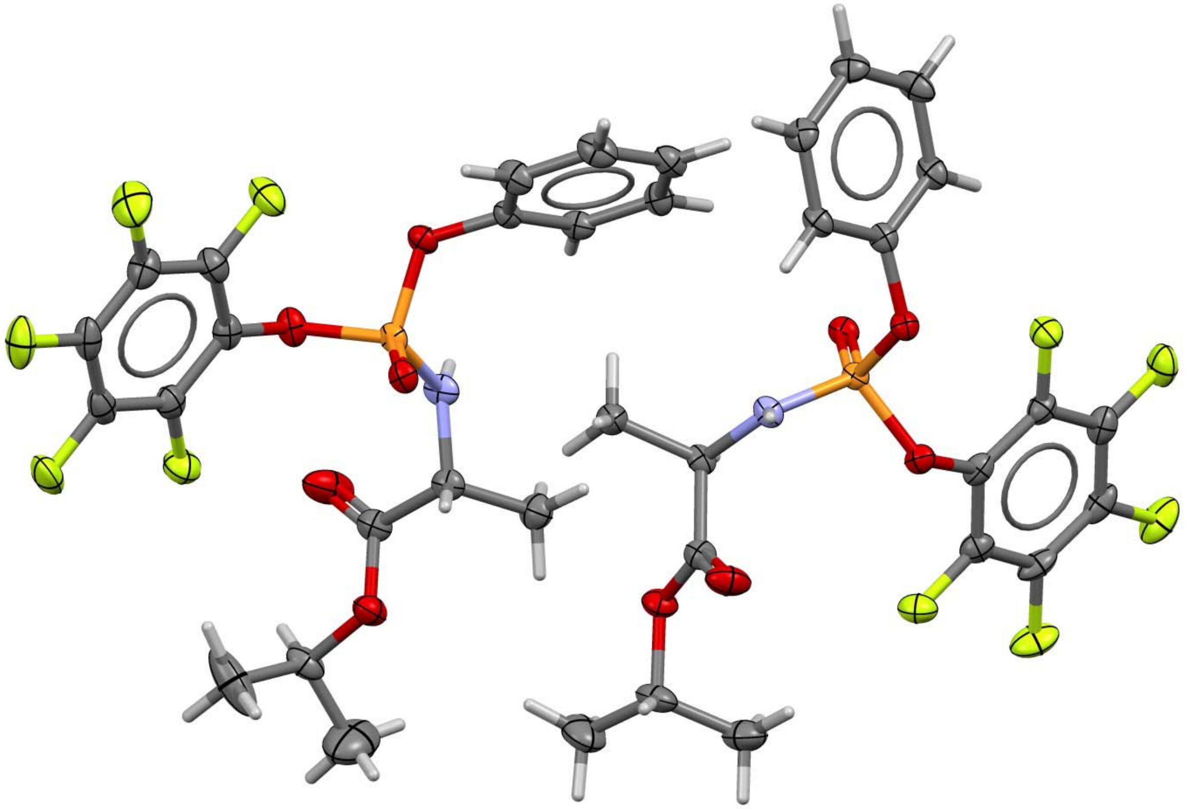

Table 1. Crystal data and structure refinement Compound 4 (CCDC 1525737)

Identification code	mdl087	
Empirical formula	C18 H17 F5 N O5 P	
Formula weight	453.29	
Temperature	100(2) K	
Wavelength	1.54178 Å	
Crystal system	Triclinic	
Space group	P1	
Unit cell dimensions	a = 5.2533(2) Å	α= 74.879(2)°.
	b = 12.0015(5) Å	β= 84.088(2)°.
	c = 16.3874(7) Å	γ = 80.148(2)°.
Volume	980.85(7) $Å^3$	
Z	2	
Density (calculated)	1.535 g/cm^3	
Absorption coefficient	1.976 mm^{-1}	
F(000)	464	
Crystal size	0.120 x 0.020 x 0.020 mm^3	
Theta range for data collection	2.798 to 68.457°.	
Index ranges	-6<=h<=6, -14<=k<=14, -19<=l<=19	
Reflections collected	23764	
Independent reflections	6829 [R(int) = 0.0348]	
Completeness to theta = 68.250°	99.2 %	
Absorption correction	Semi-empirical from equivalents	
Max. and min. transmission	0.961 and 0.845	
Refinement method	Full-matrix least-squares on F^2	
Data / restraints / parameters	6829 / 3 / 553	
Goodness-of-fit on F^2	0.989	
Final R indices [I>2sigma(I)]	R1 = 0.0351, wR2 = 0.0865	
R indices (all data)	R1 = 0.0368, wR2 = 0.0877	
Absolute structure parameter	0.010(18)	
Largest diff. peak and hole	0.230 and -0.278 e.$Å^{-3}$	

Crystal Data and Structure Refinement for Compound 2a (CCDC 1525736)

A single crystal grown from dichloromethane and methanol by solvent evaporation was selected for single crystal X-ray data analysis. The crystal was a small colorless plate with dimensions of 0.12 mm x 0.10 mm x 0.02mm. Data collection was performed on a Bruker Apex II system at 100K. The unit cell was determined to be orthorhombic in space group $P2_12_12_1$. Crystallographic data is summarized in Table 1. Absolute configuration was determined by anomalous-dispersion effects in diffraction measurements on the crystal and confirmed that the stereochemistry at all of the stereogenic centres was *R*. Figure 1 shows a thermal ellipsoid representation of Compound **2a** with thermal ellipsoids set at the 50% probability level. Coordinates, refinement details and structure factors have been deposited with the Cambridge Crystallographic Data Centre (CCDC 1525736).

Figure 1: Thermal ellipsoid representation of Compound 2a with thermal ellipsoids set at the 50% probability level.

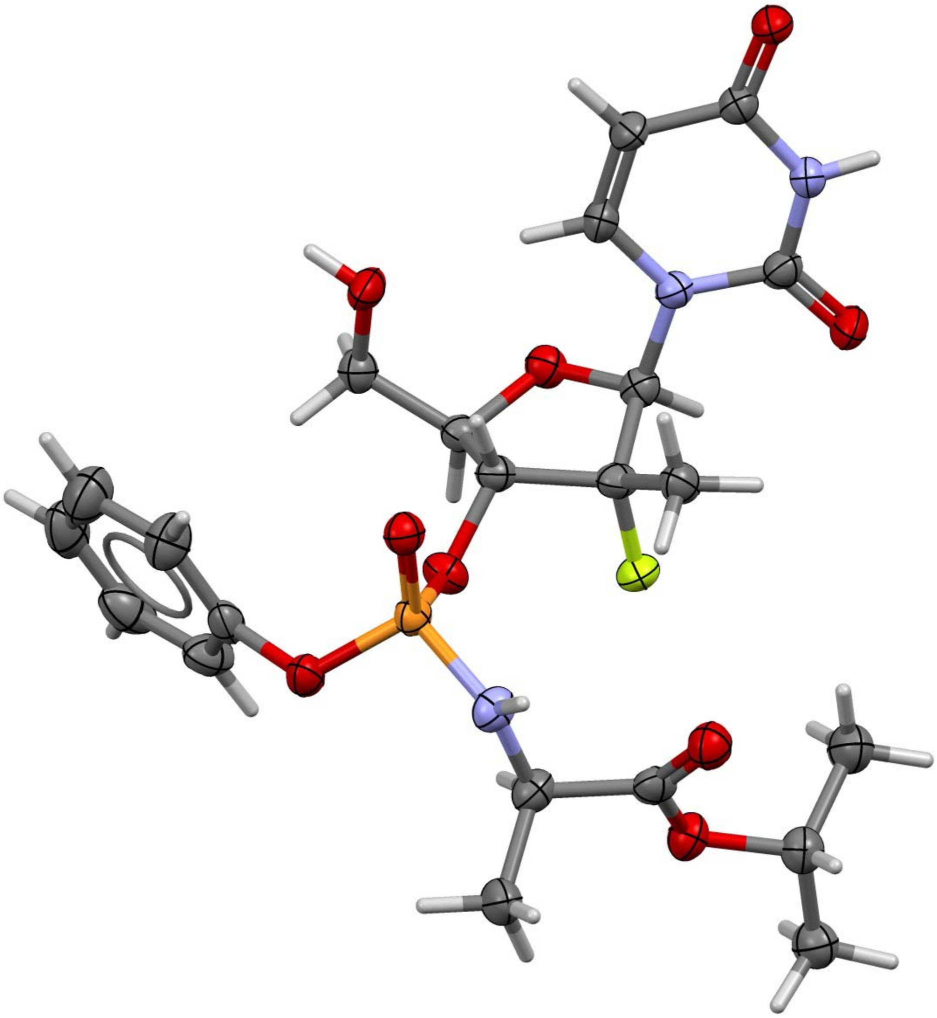

Table 1. Crystal data and structure refinement Compound 2a (CCDC 1525736)

Identification code	mdl086	
Empirical formula	C22 H29 F N3 O9 P	
Formula weight	529.45	
Temperature	100(2) K	
Wavelength	1.54178 Å	
Crystal system	Orthorhombic	
Space group	$P2_12_12_1$	
Unit cell dimensions	a = 11.355(3) Å	α= 90°.
	b = 13.612(3) Å	β= 90°.
	c = 16.835(4) Å	γ = 90°.
Volume	2602.1(10) $Å^3$	
Z	4	
Density (calculated)	1.351 g/cm^3	
Absorption coefficient	1.480 mm^{-1}	
F(000)	1112	
Crystal size	0.120 x 0.100 x 0.020 mm^3	
Theta range for data collection	4.177 to 68.339°.	
Index ranges	-13<=h<=13, -13<=k<=16, -19<=l<=20	
Reflections collected	11896	
Independent reflections	4596 [R(int) = 0.0482]	
Completeness to theta = 68.250°	98.1 %	
Absorption correction	Semi-empirical from equivalents	
Max. and min. transmission	0.971 and 0.778	
Refinement method	Full-matrix least-squares on F^2	
Data / restraints / parameters	4596 / 0 / 336	
Goodness-of-fit on F^2	1.008	
Final R indices [I>2sigma(I)]	R1 = 0.0435, wR2 = 0.1022	
R indices (all data)	R1 = 0.0575, wR2 = 0.1095	
Absolute structure parameter	-0.02(2)	
Largest diff. peak and hole	0.243 and -0.289 e.$Å^{-3}$	

Computational Methodology

The general approach used for conformational sampling, including the detailed computational workflow, has been published elsewhere.[1] This conformational sampling algorithm allows for the identification of a diverse set of conformations used to identify a Boltzmann conformer distribution. While we have used molecular mechanics energy minimization in a pre-filtering stage for many applications of this workflow, herein we chose to ignore any molecular mechanics minimization and instead select diverse conformations based only on maximizing positional variation. A total of 180 conformers for each molecule were sampled; we found hydroxyl rotamers to be most sensitive to conformer generation and clustering. Diverse conformers of each nucleoside were then geometry optimized at the B3LYP/6-31G** level and stationary points were confirmed by performing frequency calculations (scaled by 0.98).[2-6] From this initial conformer search the Boltzmann distribution was calculated and only conformers which contributed more than 0.1% to the *in vacuo* distribution were considered further. Secondary calculations were run using the M06-2X[7] functional with the 6-31+G** basis set and implicit solvent calculations in THF and DMF using either the B3LYP/6-31G** or M06-2X/6-31+G** functional/basis set combinations with the Minnesota solvation model SMD[8]. All calculations were performed using Gaussian 09.[9] Free energy distributions were used to calculate relative percentages for the major *syn* or *anti* conformation of each nucleoside when considering *in vacuo* methods, whereas electronic energies were used to estimate conformer populations when including implicit solvent.

7. References

1) E.C. Sherer, C.H. Lee, J. Shpungin, J.F. Cuff, C.X. Da, R. Ball, R. Bach, A. Crespo, X.Y. Gong, and C.J. Welch, Journal of Medicinal Chemistry, 2014, 57(2), 477.
2) Becke, A. D. Density-functional thermochemistry. III. The Role of Exact Exchange. J. Chem. Phys. 1993, 98, 5648-5652.
3) Lee, C. T., Yang, W. T., Parr, R. G. Development of the Colle-Salvetti Correlation-Energy Formula into a Functional of the Electron-Density. Physical Review B 1988, 37, 785-789.
4) Miehlich, B., Savin, A., Stoll, H., Preuss, H. Results Obtained with the Correlation-Energy Density Functionals of Becke and Lee, Yang and Parr. Chemical Physics Letters 1989, 157, 200-206.
5) Hehre, W. J., Ditchfield, R., Pople, J. A. Self-Consistent Molecular Orbital Methods. 12. Further extensions of Gaussian-type basis sets for use in molecular-orbital studies of organic-molecules. J. Chem. Phys 1972, 56, 2257.
6) Ditchfield, R., Hehre, W. J., Pople, J. A. Self-Consistent Molecular Orbital-Methods. IX. An Extended Gaussian-Type Basis for Molecular-Orbital Studies of Organic Molecules. J. Chem. Phys. 1971, 54, 724-728.
7) Zhao, Y., Truhlar, D. G. The M06 suite of density functionals for main group thermochemistry, thermochemical kinetics, noncovalent interactions, excited states, and transition elements: two new functionals and systematic testing of four M06-class functionals and 12 other functionals. Theoretical Chemistry Accounts 2008, 120, 215-241.
8) A. V. Marenich, C. J. Cramer, and D. G. Truhlar, "Universal solvation model based on solute electron density and a continuum model of the solvent defined by the bulk dielectric constant and atomic surface tensions," J. Phys. Chem. B, 113 (2009) 6378-6396.

9) M.J. Frisch, G.W. Trucks, H.B. Schlegel, G.E. Scuseria, M.A. Robb, J.R. Cheeseman, G. Scalmani, V. Barone, B. Mennucci, G.A. Petersson, H. Nakatsuji, M. Caricato, X. Li, H.P. Hratchian, A.F. Izmaylov, J. Bloino, G. Zheng, J.L. Sonnenberg, M. Hada, M. Ehara, K. Toyota, R. Fukuda, J. Hasegawa, M. Ishida, T. Nakajima, Y. Honda, O. Kitao, H. Nakai, T. Vreven, J. Montgomery, J.A., J.E. Peralta, F. Ogliaro, M. Bearpark, J.J. Heyd, E. Brothers, K.N. Kudin, V.N. Staroverov, R. Kobayashi, J. Normand, K. Raghavachari, A. Rendell, J.C. Burant, S.S. Iyengar, J. Tomasi, M. Cossi, N. Rega, J.M. Millam, M. Klene, J.E. Knox, J.B. Cross, V. Bakken, C. Adamo, J. Jaramillo, R. Gomperts, R.E. Stratmann, O. Yazyev, A.J. Austin, R. Cammi, C. Pomelli, J.W. Ochterski, R.L. Martin, K. Morokuma, V.G. Zakrzewski, G.A. Voth, P. Salvador, J.J. Dannenberg, S. Dapprich, A.D. Daniels, Ö. Farkas, J.B. Foresman, J.V. Ortiz, J. Cioslowski, and D.J. Fox. Gaussian 09, Revision A.02. Wallingford, CT: Gaussian, Inc.; 2009.

Table S1: Syn and Anti conformational distributions (%)

		Free Energy THF	Free Energy in vacuo	Free Energy in vacuo	Elec Energy DMF	Elec Energy THF
		**M06-2X/6-31+G****	**B3LYP/6-31G****	**M06-2X/6-31+G****	**B3LYP/6-31G** DMF**	**B3LYP/6-31G** THF**
2'MeF Uracil (1a)	Syn	**56**	**21**	**19**	**85**	**76**
	Anti	**44**	**77**	**81**	**16**	**24**
2'MeF Uracil anion (1a)	Syn	**100**	**100**	**100**	**100**	**100**
	Anti	**0**	**0**	**0**	**0**	**0**
2'MeF Uracil methylated (5)	Syn	**86**	**24**	**50**	**64**	**63**
	Anti	**14**	**73**	**46**	**35**	**36**
12'MeCl Uracil (1b)	Syn	**27**	**16**	**10**	**52**	**49**
	Anti	**73**	**82**	**90**	**47**	**51**
2'MeCl Uracil anion (1b)	Syn	**100**	**100**	**100**	**100**	**100**
	Anti	**0**	**0**	**0**	**0**	**0**
2'MeAlkyne Uracil (1c)	Syn	**9**	**5**	**5**	**54**	**47**
	Anti	**91**	**93**	**95**	**46**	**53**
2'MeAlkyne Uracil anion (1c)	Syn	**100**	**100**	**100**	**100**	**100**
	Anti	**0**	**0**	**0**	**0**	**0**
2'MeAzide Uracil (1d)	Syn	**7**	**1**	**1**	**3**	**2**
	Anti	**93**	**96**	**93**	**88**	**91**
2'MeAzide Uracil anion (1d)	Syn	**100**	**100**	**100**	**100**	**100**
	Anti	**0**	**0**	**0**	**0**	**0**
2'MeCyano Uracil (1e)	Syn	**13**	**3**	**2**	**28**	**25**
	Anti	**87**	**96**	**97**	**71**	**75**
2'MeCyano Uracil anion (1e)	Syn	**100**	**100**	**100**	**100**	**100**
	Anti	**0**	**0**	**0**	**0**	**0**

Table S1 (cont.): Syn and Anti conformational distributions (%)

		Free Energy THF	Free Energy in vacuo	Free Energy in vacuo	Elec Energy DMF	Elec Energy THF
		M06-2X/6-31+G**	B3LYP/6-31G**	M06-2X/6-31+G**	B3LYP/6-31G** DMF	B3LYP/6-31G** THF
alpha thymidine (13)	Syn	45	53	86	93	93
	Anti	55	44	14	7	7
alpha thymidine anion (13)	Syn	100	100	100	100	100
	Anti	0	0	0	0	0
cytidine (15)	Syn	14	0	0	7	4
	Anti	86	97	100	94	96
2' Me azide Me guanosine (7)	Syn	99	96	98	98	99
	Anti	1	1	2	2	1
2' diF cytosine (9)	Syn	98	95	97	99	99
	Anti	2	2	3	1	1
2' MeCl inosine (11)	Syn	90	82	56	96	96
	Anti	10	17	44	4	4
2' MeCl inosine anion (11)	Syn	100	100	100	100	100
	Anti	0	0	0	0	0

Table S2: Detail for conformer distributions for any conformer >0.1% from original B3LYP/6-31G conformer search. All methods tabulated.**

					M06-2X/6-31+G** THF		B3LYP/6-31G**		M06-2X/6-31+G**		B3LYP/6-31G** DMF		B3LYP/6-31G** THF
**Compound 1a**					G		G		G		E		E
Neutral	2'MeF Uracil	2	Anti	-973.88519	38	-974.19454	77	-973.86096	80	-974.41583	15	-974.41294	24
		5	Syn O5'	-973.88538	47	-974.19334	21	-973.85955	18	-974.41747	84	-974.41404	75
		10	Syn O5'	-973.8838	9	-974.18943	0	-973.85701	1	-974.41289	1	-974.40951	1
		6	Anti	-973.88338	6	-974.18961	0	-973.85667	1	-974.41284	1	-974.40914	0
2'MeF Uracil			Sum Syn		56		21		19		85		76
			Sum Anti		44		77		81		16		24
**Compound 1a**					M06-2X/6-31+G** THF		B3LYP/6-31G**		M06-2X/6-31+G**		B3LYP/6-31G** DMF		B3LYP/6-31G** THF
					G		G		G		E		E
Anion	2'MeF Uracil	5	Syn O5'	-973.41142	100	-973.64703	100	-973.33366	100	-973.33366	100	-973.91677	100
2'MeF Uracil anion			Sum Syn		100		100		100		100		100
			Sum Anti		0		0		0		0		0
**Compound 5**					M06-2X/6-31+G** THF		B3LYP/6-31G**		M06-2X/6-31+G**		B3LYP/6-31G** DMF		B3LYP/6-31G** THF
					G		G		G		E		E
Methylated	2'MeF Uracil	Mol_5	Syn O5'	-1013.15	75	-1013.4807	23	-1013.1264	48	-1013.7276	64	-1013.725	62
		Mol_16	Anti	-1013.1482	11	-1013.4816	59	-1013.1262	39	-1013.7268	28	-1013.7243	31
		Mol_14	Anti	-1013.1465	2	-1013.4798	9	-1013.1242	5	-1013.7252	5	-1013.7222	3
		Mol_6	Anti	-1013.1461	1	-1013.4792	5	-1013.1232	2	-1013.7245	2	-1013.7216	2
		Mol_12	Syn no hbond	-1013.1469	3	-1013.4786	2	-1013.124	4	-1013.7237	1	-1013.7213	1
		Mol_13	Syn O5'	-1013.1479	8	-1013.4767	0	-1013.1234	2	-1013.723	0	-1013.7204	0
		Mol_69	Syn O5'	-1013.142	0	-1013.4756	0	-1013.1204	0	-1013.7209	0	-1013.7187	0
2'MeF Uracil methylated			Sum Syn		83		24		50		64		63
			Sum Anti		14		73		46		35		36
			Sum Syn no Hbond		3		2		4		1		1

					M06-2X/6-31+G** THF		B3LYP/6-31G**		M06-2X/6-31+G**		B3LYP/6-31G** DMF		B3LYP/6-31G** THF
**Compound 1b**	2'MeCl Uracil				G		G		G		E		E
		Mol_4	Anti	-1334.2424	44	-1334.5539	76	-1334.2177	85	-1334.7755	41	-1334.7722	46
		Mol_3	Syn O5'	-1334.2417	21	-1334.5524	16	-1334.2155	9	-1334.7756	49	-1334.7722	46
Neutral		Mol_14	Anti	-1334.2391	1	-1334.5513	5	-1334.2141	2	-1334.7733	4	-1334.7696	3
		Mol_12	Syn no hbond	-1334.2376	0	-1334.5498	1	-1334.2125	0	-1334.7708	0	-1334.7677	0
		Mol_101	Syn O5'	-1334.2362	0	-1334.5486	0	-1334.2119	0	-1334.7696	0	-1334.7667	0
		Mol_27	Syn O5'	-1334.2404	6	-1334.5495	1	-1334.2133	1	-1334.7731	4	-1334.7696	3
		Mol_115	Anti	-1334.2419	28	-1334.5502	1	-1334.2147	3	-1334.7726	2	-1334.769	2
2'MeCl Uracil			Sum Syn		27		16		10		52		49
			Sum Anti		73		82		90		47		51
**Compound 1b**					M06-2X/6-31+G** THF		B3LYP/6-31G**		M06-2X/6-31+G**		B3LYP/6-31G** DMF		B3LYP/6-31G** THF
Anion	2'MeCl Uracil				G		G		G		E		E
		Mol_113	Syn O5'	-874.18207	39	-874.39521	81	-874.10144	90	-874.67668	31	-874.66535	48
		Mol_3	Syn O5'	-874.1813	31	-874.39387	19	-874.09939	10	-874.67745	69	-874.66543	52
2'MeCl Uracil anion			Sum Syn		100		100		100		100		100
			Sum Anti		0		0		0		0		0
**Compound 1c**					M06-2X/6-31+G** THF		B3LYP/6-31G**		M06-2X/6-31+G**		B3LYP/6-31G** DMF		B3LYP/6-31G** THF
Neutral	2'MeAlkyne Uracil				G		G		G		E		E
		Mol_16	Anti	-950.77194	71	-951.07926	71	-950.74686	85	-951.3157	25	-951.31296	36
		Mol_4	Anti	-950.77019	11	-951.07755	12	-950.74383	3	-951.3149	11	-951.31155	8
		Mol_179	Anti	-950.76775	1	-951.07703	7	-950.74369	3	-951.31453	7	-951.3113	6
		Mol_11	Syn O5'	-950.76986	8	-951.07672	5	-950.74404	4	-951.31642	53	-951.31319	45
		Mol_12	Anti	-950.76775	1	-951.07558	1	-950.74305	2	-951.31333	2	-951.31009	2
		Mol_41	Anti	-950.76785	1	-951.07506	1	-950.74247	1	-951.31134	0	-951.30861	0
		Mol_115	Anti	-950.76952	5	-951.07459	1	-950.74305	2	-951.31218	1	-951.30896	1
		Mol_10	Anti	-950.76809	1	-951.07428	0	-950.74141	0	-951.31271	1	-951.30931	1
		Mol_156	Syn O5'	-950.76799	1	-951.07415	0	-950.74241	1	-951.31279	1	-951.30967	1
2'MeAlkyne Uracil			Sum Syn		9		5		5		54		47
			Sum Anti		91		93		95		46		53

**Compound 1c**					M06-2X/6-31+G** THF		B3LYP/6-31G**		M06-2X/6-31+G**		B3LYP/6-31G** DMF		B3LYP/6-31G** THF
					G		G		G		E		E
Anion	2'MeAlkyne Uracil	18	Syn O5'	-950.496	100	-950.52434	99	-950.21194	100	-950.81099	100	-950.8	100
2'MeAlkyne Uracil anion			Sum Syn		100		100		100		100		100
			Sum Anti		0		0		0		0		0
**Compound 1d**					M06-2X/6-31+G** THF		B3LYP/6-31G**		M06-2X/6-31+G**		B3LYP/6-31G** DMF		B3LYP/6-31G** THF
Neutral	2'MeAzide Uracil				G		G		G		E		E
		Mol_102	Syn O5'	-1038.1882	5	-1038.5275	1	-1038.1664	1	-1038.7556	3	-1038.7517	2
		Mol_108	anti	-1038.1898	29	-1038.5281	1	-1038.1674	4	-1038.7567	8	-1038.7532	7
		Mol_116	anti	-1038.1871	2	-1038.5271	0	-1038.1665	1	-1038.753	0	-1038.75	0
		Mol_119	Syn no hbond	-1038.1873	2	-1038.5266	0	-1038.1667	2	-1038.7528	0	-1038.7498	0
		Mol_124	anti	-1038.1896	23	-1038.5295	5	-1038.1673	3	-1038.7578	26	-1038.7543	23
		Mol_141	anti	-1038.1872	2	-1038.5271	0	-1038.1656	1	-1038.7542	1	-1038.7503	0
		Mol_2	anti	-1038.19	35	-1038.5321	82	-1038.1702	74	-1038.7585	56	-1038.7552	62
		Mol_7	anti	-1038.1874	2	-1038.53	9	-1038.1686	13	-1038.7563	6	-1038.7529	5
2'MeAzide Uracil			Sum Syn		5		1		1		3		2
			Sum Anti		93		98		97		97		98
			Sum Syn w/o Hbond		2		0		2		0		0
**Compound 1d**					M06-2X/6-31+G** THF		B3LYP/6-31G**		M06-2X/6-31+G**		B3LYP/6-31G** DMF		B3LYP/6-31G** THF
Anion	2'MeAzide Uracil				G		G		G		E		E
		Mol_11	Syn O5'	-1037.715	56	-1037.9853	85	-1037.6413	53	-1038.258	75	-1038.2469	69
		Mol_107	Syn O5'	-1037.7148	42	-1037.9836	14	-1037.6412	46	-1038.2569	24	-1038.2461	30
		Mol_48	Syn O5'	-1037.7117	2	-1037.9804	0	-1037.6364	0	-1038.2537	1	same as 1	same as 1
		Mol_1	Syn O5'	N/A	0	-1037.9803	0	-1037.6347	0	-1038.2527	0	-1038.2425	1
		Mol_117	Syn O5'	-1037.71	0	-1037.9798	0	-1037.6371	1	-1038.2522	0	-1038.2414	0
2'MeAzide Uracil anion			Sum Syn		100		100		100		100		100
			Sum Anti		0		0		0		0		0

**Compound 1e**					**M06-2X/6-31+G** THF**		**B3LYP/6-31G****		**M06-2X/6-31+G****		**B3LYP/6-31G** DMF**		**B3LYP/6-31G** THF**
Neutral	2'MeCyano Uracil				**G**		**G**		**G**		**E**		**E**
		Mol_179	anti	-966.87657	20	-967.18498	51	-966.85059	62	-967.41422	12	-967.41049	20
		Mol_13	anti	-966.87512	4	-967.18455	32	-966.8496	22	-967.41484	23	-967.41075	26
		Mol_15	anti	-966.87472	3	-967.18261	4	-966.84668	1	-967.41315	4	-967.40858	3
		Mol_113	anti	-966.87701	32	-967.18228	3	-966.84856	7	-967.41386	8	-967.40962	8
		Mol_104	anti	-966.87641	17	-967.18179	2	-966.84748	2	-967.4129	3	-967.40868	3
		Mol_4	anti	-966.87203	0	-967.18176	2	-966.84624	1	-967.4125	2	-967.40804	2
		Mol_38	Syn O5'	-966.87618	13	-967.18165	1	-966.84716	2	-967.41498	26	-967.41058	22
		Mol_20	Syn O5'	-966.87215	0	-967.18145	1	-966.84631	1	-967.41254	2	-967.40865	3
		Mol_10	anti	-966.87468	3	-967.18144	1	-966.84646	1	-967.4138	8	-967.40915	5
		Mol_1	anti	-966.875	4	-967.1813	1	-966.84656	1	-967.41204	1	-967.40797	1
		Mol_174	anti	-966.874	1	-967.18061	0	-966.84587	0	-967.41387	8	-967.40917	5
		Mol_112	anti	-966.8747	3	-967.18049	0	-966.8465	1	-967.41292	3	-967.40819	2
		Mol_103	Syn no hbond	-966.87341	1	-967.17956	0	-966.84604	1	-967.41019	0	-967.40629	0
2'MeCyano Uracil			Sum Syn		13		3		2		28		25
			Sum Anti		87		96		97		71		75
**Compound 1e**					**M06-2X/6-31+G** THF**		**B3LYP/6-31G****		**M06-2X/6-31+G****		**B3LYP/6-31G** DMF**		**B3LYP/6-31G** THF**
Anion	2'MeCyano Uracil				**G**		**G**		**G**		**E**		**E**
		Mol_12	Syn O5'	-966.40291	58	-966.6422	82	-966.3266	66	-966.91563	63	-966.90456	66
		Mol_109	Syn O5'	me as Mol_1	N/A	-966.64006	9	-966.32542	19	same as 100		same as 100	
		Mol_100	Syn O5'	-966.40322	42	-966.64014	9	-966.32522	15	-966.91512	37	-966.90392	34
2'MeCyano Uracil anion			Sum Syn		100		100		100		100		100
			Sum Anti		0		0		0		0		0

**Compound 13**					**M06-2X/6-31+G** THF**		**B3LYP/6-31G****		**M06-2X/6-31+G****		**B3LYP/6-31G** DMF**		**B3LYP/6-31G** THF**
					**G**		**G**		**G**		**E**		**E**
alpha thymidine		Mol_11	Syn 03'	-874.66608	25	-874.95692	25	-874.6409	40	-875.18815	28	-875.18525	38
		Mol_178	Anti	-874.66574	17	-874.95642	15	-874.63849	3	-875.18534	1	-875.18239	2
		Mol_103	Syn 03'	-874.66502	8	-874.95632	13	-874.64075	34	-875.18655	5	-875.18383	8
		Mol_179	Syn 03'	-874.66527	10	-874.95623	12	-874.63949	9	-875.18869	51	-875.18529	40
		Mol_14	Anti	-874.66494	7	-874.9559	8	-874.63618	0	-875.1852	1	-875.18218	1
		Mol_9	Anti	-874.66497	8	-874.95566	7	-874.63821	2	same as 14		same as 14	
		Mol_105	Anti	-874.66504	8	-874.95512	4	-874.63752	1	-875.1842	0	-875.18133	1
		Mol_169	Anti	-874.66501	8	-874.95501	3	-874.63644	0	-875.18594	3	-875.18238	2
		Mol_174	Anti	-874.6637	2	-874.95498	3	-874.63893	5	-875.18431	0	-875.18147	1
		Mol_1	Anti	N/A	0	-874.95461	2	N/A		N/A		N/A	
		Mol_180	Syn 03'	-874.66336	2	-874.9543	2	-874.63651	0	-875.18653	5	-875.18316	4
		Mol_102	Anti	-874.66358	2	-874.95396	1	-874.63782	2	-875.18421	0	-875.18129	1
		Mol_109	Anti	-874.66405	3	-874.95386	1	-874.63663	0	-875.18354	0	-875.18074	0
		Mol_10	Syn 03'	-874.66205	0	-874.95384	1	-874.63713	1	-875.1857	2	-875.18199	1
		Mol_153	Syn 03'	-874.66239	0	-874.95277	0	-874.63638	0	-875.18516	1	-875.1816	1
		Mol_158	Syn 03'	-874.66181	0	-874.95208	0	-874.6365	0	-875.184	0	-875.18054	0
alpha thymidine			Sum Syn		45		53		86		93		93
			Sum Anti		55		44		14		7		7
**Compound 13**					**M06-2X/6-31+G** THF**		**B3LYP/6-31G****		**M06-2X/6-31+G****		**B3LYP/6-31G** DMF**		**B3LYP/6-31G** THF**
					**G**		**G**		**G**		**E**		**E**
alpha thymidine anion		Mol_144	Syn 03'	-874.18738	96	-874.40593	91	-874.1085	87	-874.68506	92	-874.67382	96
		Mol_15	Syn 03'	-874.18396	3	-874.40375	9	-874.10673	13	-874.68178	3	-874.66953	1
		Mol_9	Syn 03'	-874.18294	1	-874.40015	0	-874.1025	0	-874.6824	5	-874.67069	3
alpha thymidine anion			Sum Syn		100		100		100		100		100
			Sum Anti		0		0		0		0		0

**Compound 15**					**M06-2X/6-31+G** THF**		**B3LYP/6-31G****		**M06-2X/6-31+G****		**B3LYP/6-31G** DMF**		**B3LYP/6-31G** THF**
cytidine					**G**		**G**		**G**		**E**		**E**
		Mol_100	Anti	-890.7047	33	-890.9845	11	-890.67581	50	-891.20926	13	-891.20531	14
		Mol_163 or Mol_127	Anti	-890.70413	18	-890.98548	32	-890.67524	27	-891.20925	12	-891.20529	14
		Mol_7	Anti	-890.70372	12	-890.98325	3	-890.67432	10	-891.20896	9	-891.20482	9
		Mol_11	Anti	-890.70209	2	-890.98307	2	-890.67308	3	-891.21015	32	-891.20587	26
driver is Hbond to O2'		Mol_1	Anti	-890.70336	8	-890.98439	10	-890.67303	3	-891.2096	18	-891.20517	12
		Mol_10	Anti	-890.70301	6	-890.98165	1	-890.67294	2	-891.20786	3	-891.20346	2
		Mol_175	Anti	-890.70015	0	-890.98177	1	-890.67285	2	-891.20501	0	-891.20134	0
		Mol_106	Anti	-890.70238	3	-890.98228	1	-890.67214	1	-891.20747	2	-891.20309	1
		Mol_174	Anti	-890.70227	3	-890.98247	1	-890.67179	1	-891.20822	4	-891.2038	3
		Mol_107	Syn O5'	-890.70334	8	-890.97977	0	-890.67126	0	-891.20864	7	-891.20405	4
		Mol_46	Anti	same as 100		-890.98465	13	same as 100		same as 100		same as 100	
		Mol_180	Anti	same as 163		-890.98514	22	same as 163		same as 163		same as 163	
		Mol_128	Anti	same as 163		Same as 163		Same as 163		Same as 163		-891.20529	14
		Mol_178	Syn O5'	-890.70315	6		0		0		0		0
		Mol_116	Anti	-890.70151	1		0		0		0		0
cytidine			Sum Syn		14		0		0		7		4
			Sum Anti		86		97		100		94		96
**Compound 7**					**M06-2X/6-31+G** THF**		**B3LYP/6-31G****		**M06-2X/6-31+G****		**B3LYP/6-31G** DMF**		**B3LYP/6-31G** THF**
					**G**		**G**		**G**		**E**		**E**
2' Me azide Me guanosine		Mol_4	Syn O5'	-1205.1322	96	-1205.5268	52	-1205.1061	92	-1205.8132	87	-1205.8099	97
#2 from table		Mol_65	Syn O5'	ame as Mol_	0	-1205.5266	42	same as 4		same as 4		same as 4	
		Mol_20	Syn O5'	me as Mol_1	0	-1205.5232	1	same as 127		same as 127		same as 127	
		Mol_127	Syn O5'	-1205.1287	2	-1205.5227	1	-1205.1034	5	-1205.8085	1	-1205.8055	1
		Mol_5	anti	-1205.1282	1	-1205.5227	1	-1205.1023	2	-1205.8094	2	-1205.8058	1
		Mol_106	Syn O5'	-1205.1266	0	-1205.5224	0	-1205.1007	0	-1205.8112	11	-1205.8056	1
2' Me azide Me guanosine			Sum Syn		98		96		98		98		99
			Sum Anti		1		1		2		2		1

**Compound 9**					**M06-2X/6-31+G** THF**		**B3LYP/6-31G****		**M06-2X/6-31+G****		**B3LYP/6-31G** DMF**		**B3LYP/6-31G** THF**
					**G**		**G**		**G**		**E**		**E**
2' diF cytosine		Mol_1	anti	-1013.9472	1	-1014.2494	2	-1013.9164	2	-1014.4528	0	-1014.4482	1
		Mol_100	Syn O5'	-1013.9464	0	-1014.2475	0	-1013.9154	1	-1014.4535	1	-1014.4488	1
		Mol_13	Syn O5'	-1013.9514	52	-1014.2504	5	-1013.9197	57	-1014.4558	10	-1014.4512	14
		Mol_181	Syn O5'	-1013.9513	46	-1014.252	26	-1013.9193	39	-1014.4579	89	-1014.4529	84
		Mol_7	anti	-1013.9473	1	-1014.2485	1	-1013.9162	1	-1014.4524	0	-1014.4478	0
		Mol_77	Syn O5'	me as Mol_	0	-1014.2515	15	same as 13		same as 13		same as 13	
		Mol_8	Syn O5'	me as Mol_1	0	-1014.2526	49	same as 181		same as 181		same as 181	
2' diF cytosine			Sum Syn		98		95		97		99		99
			Sum Anti		2		2		3		1		1
**Compound 11**					**M06-2X/6-31+G** THF**		**B3LYP/6-31G****		**M06-2X/6-31+G****		**B3LYP/6-31G** DMF**		**B3LYP/6-31G** THF**
2' MeCl inosine					**G**		**G**		**G**		**E**		**E**
		Mol_1	anti	-1406.5913	3	-1406.9118	4	-1406.555	4	-1407.1567	1	-1407.1518	1
		Mol_10	Syn O5'	-1406.5927	13	-1406.911	2	-1406.555	4	-1407.158	3	-1407.1532	3
		Mol_107	Syn O5'	-1406.5915	4	-1406.9119	4	-1406.5555	6	-1407.1576	2	-1407.1535	4
		Mol_15	anti	-1406.5919	6	-1406.9127	10	-1406.5572	39	-1407.158	3	-1407.1533	3
		Mol_18	anti	-1406.5896	1	-1406.9113	2	-1406.5536	1	-1407.1559	0	-1407.1511	0
		Mol_180	Syn O5'	-1406.5943	73	-1406.9146	76	-1406.5574	46	-1407.1612	91	-1407.1566	90
2' MeCl inosine			Sum Syn		90		82		56		96		96
			Sum Anti		10		17		44		4		4
**Compound 11**					**M06-2X/6-31+G** THF**		**B3LYP/6-31G****		**M06-2X/6-31+G****		**B3LYP/6-31G** DMF**		**B3LYP/6-31G** THF**
2' MeCl inosine anion					**G**		**G**		**G**		**E**		**E**
		Mol_10	Syn O5'	-1406.1251	82	-1406.3791	96	-1406.0403	89	-1406.6668	96	-1406.6556	95
		Mol_108	Syn O5'	-1406.1236	17	-1406.3759	3	-1406.0383	11	-1406.6636	3	-1406.6526	4
		Mol_123	Syn O5'	-1406.1212	1	-1406.374	0	-1406.0351	0	-1406.6623	1	-1406.6513	1
2' MeCl inosine anion			Sum Syn		100		100		100		100		100
			Sum Anti		0		0		0		0		0

Coordinates of Minima

Compound 1a: 2’ MeF Uracil

Conf 2

6 2.18976 -0.078723 -0.679894

6 1.24455 -1.22422 -0.288528

6 0.373701 -0.60536 0.829915

8 1.10259 0.489202 1.34928

6 2.35629 0.65781 0.662861
7 -0.957031 -0.135578 0.394001
6 2.68226 2.13452 0.554456
6 0.554974 -1.95618 -1.41545
8 3.39644 -0.518556 -1.25464
9 2.08704 -2.1565 0.381148
6 -1.16536 1.15693 -0.048401
6 -2.38305 1.60963 -0.421904
6 -3.54855 0.743727 -0.350425
7 -3.23878 -0.546993 0.136142
6 -2.01134 -1.05121 0.513194
8 1.73008 2.75642 -0.302961
8 -1.84931 -2.19727 0.90699
8 -4.69584 1.0268 -0.654441
1 1.70691 0.582816 -1.40453
1 0.191881 -1.36365 1.5942
1 3.15497 0.169692 1.23995
1 3.70192 2.22258 0.151439
1 2.66654 2.57854 1.55946
1 -0.026348 -2.79422 -1.02618
1 1.31212 -2.32623 -2.11151
1 -0.113034 -1.28435 -1.96036
1 3.67079 -1.29197 -0.73787
1 -0.281524 1.78497 -0.058059
1 -2.52189 2.62539 -0.765377
1 -4.01278 -1.19602 0.211955
1 1.90677 3.70464 -0.323252

Conf 5
6 2.29396 -0.148987 -0.505107
6 1.26175 -1.26883 -0.29131
6 0.314237 -0.710218 0.806373
8 0.918016 0.443194 1.33761
6 2.27128 0.593172 0.844407
7 -1.07785 -0.414078 0.4302
6 2.58519 2.08208 0.768639
6 0.657972 -1.882 -1.53234
8 3.56051 -0.63204 -0.884131
9 1.97884 -2.30312 0.385012
6 -2.11133 -1.07851 1.07029
6 -3.41617 -0.822514 0.847031
6 -3.80441 0.21888 -0.092775
7 -2.68874 0.862335 -0.676882
6 -1.34908 0.613215 -0.479518
8 1.89865 2.75289 -0.265036
8 -0.472875 1.23194 -1.07117
8 -4.9366 0.55591 -0.388279
1 1.95284 0.528964 -1.2879
1 0.235666 -1.49623 1.56698
1 2.96587 0.105337 1.54513
1 3.65668 2.19461 0.569431
1 2.38035 2.52535 1.75654
1 -0.014586 -2.70684 -1.28041
1 1.46852 -2.26779 -2.15602
1 0.112073 -1.12992 -2.1045
1 3.73949 -1.40006 -0.321065

1 -1.79172 -1.83686 1.77593
1 -4.19492 -1.36717 1.36232
1 -2.89338 1.60241 -1.33853
1 0.977675 2.44551 -0.27235

Conf 10
6 -2.24607 -0.169457 0.509947
6 -1.25359 -1.3124 0.269566
6 -0.301595 -0.733605 -0.819303
8 -0.926558 0.401573 -1.36514
6 -2.2758 0.535212 -0.855275
7 1.0836 -0.403386 -0.433467
6 -2.62136 2.0186 -0.814488
6 -0.636169 -1.93305 1.5031
8 -3.5046 -0.665798 0.921989
9 -1.9554 -2.32687 -0.412757
6 2.13031 -1.08622 -1.03083
6 3.43132 -0.820094 -0.795552
6 3.80168 0.253329 0.113882
7 2.67434 0.911517 0.657103
6 1.33804 0.651455 0.447963
8 -1.98953 2.71891 0.237517
8 0.455792 1.28993 1.01045
8 4.92773 0.605185 0.416672
1 -1.81686 0.514311 1.24544
1 -0.198552 -1.52253 -1.57325
1 -2.9718 0.002672 -1.51773
1 -3.70212 2.11898 -0.66042
1 -2.38905 2.45261 -1.79973
1 0.035978 -2.75331 1.23544
1 -1.44185 -2.33297 2.12397
1 -0.084496 -1.18929 2.08172
1 -3.89673 -0.008296 1.509
1 1.8244 -1.8692 -1.7152
1 4.2189 -1.38032 -1.27979
1 2.86581 1.67348 1.29734
1 -1.05388 2.45772 0.256628

Conf 6
6 -2.17329 -0.104069 0.713617
6 -1.23859 -1.2603 0.306916
6 -0.372135 -0.607412 -0.801892
8 -1.12075 0.489811 -1.30136
6 -2.39548 0.583598 -0.633371
7 0.955906 -0.134989 -0.370532
6 -2.8118 2.04619 -0.569816
6 -0.508667 -1.98601 1.41703
8 -3.41617 -0.491532 1.25184
9 -2.03681 -2.1978 -0.367321
6 1.12755 1.0861 0.25124
6 2.33444 1.54898 0.643427
6 3.53544 0.76161 0.406879
7 3.26356 -0.465429 -0.237412
6 2.04484 -0.982165 -0.630152
8 -1.81152 2.87829 0.008725
8 1.92115 -2.08114 -1.14778

8 4.67904 1.06227 0.705204
1 -1.63444 0.579485 1.38607
1 -0.185447 -1.35152 -1.57783
1 -3.1549 0.010908 -1.18265
1 -3.70177 2.11945 0.062921
1 -3.08209 2.39817 -1.57398
1 0.136825 -2.76306 1.00075
1 -1.24061 -2.46268 2.07606
1 0.101864 -1.29973 2.00978
1 -3.29282 -0.740682 2.17638
1 0.217834 1.6573 0.389722
1 2.43892 2.50988 1.12804
1 4.06121 -1.06309 -0.418735
1 -1.13732 3.00801 -0.671352

Compound 1a: 2' MeF Uracil Anion

Conf 5
6 2.26832 -0.072691 -0.480541
6 1.28073 -1.23481 -0.296431
6 0.287452 -0.763393 0.796701
8 0.889234 0.361298 1.42824
6 2.16904 0.681621 0.862135
7 -1.07661 -0.486411 0.412168
6 2.29299 2.2024 0.752827
6 0.718081 -1.85323 -1.55158
8 3.58001 -0.514085 -0.793962
9 2.05625 -2.25999 0.366114
6 -2.1115 -1.10139 1.0896
6 -3.39418 -0.786395 0.83463
6 -3.70585 0.255905 -0.165886
7 -2.64331 0.881377 -0.788968
6 -1.38288 0.561685 -0.538536
8 1.58209 2.78252 -0.30894
8 -0.376633 1.07913 -1.09246
8 -4.88419 0.544762 -0.415796
1 1.93265 0.572682 -1.28924
1 0.229931 -1.59148 1.51541
1 2.96352 0.31047 1.53284
1 3.35716 2.44239 0.611044
1 1.99484 2.62049 1.73168
1 0.071941 -2.7034 -1.31377
1 1.5454 -2.19807 -2.17947
1 0.142829 -1.10066 -2.09198
1 3.71455 -1.30034 -0.243906
1 -1.81427 -1.84812 1.82245
1 -4.21003 -1.27429 1.35553
1 0.747257 2.27831 -0.462238

Compound 5: 2' MeF Uracil methylated

Conf 5
6 -2.45354 0.020868 0.539197
6 -1.52578 -1.20404 0.468322
6 -0.594742 -0.910161 -0.740157

8 -1.12687 0.196187 -1.42591
6 -2.43262 0.548426 -0.908316
7 0.83643 -0.691989 -0.474502
6 -2.60683 2.05569 -1.04939
6 -0.909283 -1.66511 1.7673
8 -3.73738 -0.288177 1.02768
9 -2.35747 -2.26595 -0.003803
6 1.76764 -1.53093 -1.05468
6 3.09506 -1.34746 -0.925588
6 3.61537 -0.217019 -0.176007
7 2.60552 0.626096 0.372116
6 1.24257 0.430659 0.254375
8 -1.81355 2.80431 -0.154707
8 0.431941 1.1907 0.77751
8 4.79905 0.029558 -0.010161
6 3.02197 1.80544 1.1414
1 -0.316998 -2.57336 1.62414
1 -1.71433 -1.87886 2.47515
1 -0.27777 -0.881467 2.18889
1 2.64859 2.71346 0.66372
1 4.10897 1.80377 1.16333
1 2.61406 1.75319 2.15243
1 -3.65307 2.30029 -0.834474
1 -2.4103 2.32477 -2.09992
1 -2.02461 0.775684 1.19847
1 -0.624599 -1.80861 -1.36829
1 -3.20483 0.03258 -1.49924
1 1.3516 -2.35541 -1.62227
1 3.80814 -2.01955 -1.38205
1 -3.99817 -1.11835 0.601451
1 -0.927453 2.40795 -0.124348

Conf 16
6 -2.4668 -0.189571 0.688877
6 -1.39612 -1.24242 0.363949
6 -0.563905 -0.574105 -0.756854
8 -1.37439 0.458141 -1.30206
6 -2.6776 0.462828 -0.687621
7 0.715747 0.00053 -0.315638
6 -3.2025 1.89219 -0.661676
6 -0.651367 -1.85985 1.52432
8 -3.63213 -0.728931 1.26199
9 -2.1184 -2.284 -0.283508
6 0.776371 1.22847 0.306849
6 1.94285 1.77051 0.71061
6 3.20171 1.07851 0.490208
7 3.06627 -0.181343 -0.157141
6 1.87101 -0.761828 -0.552023
8 -2.28665 2.79771 -0.056987
8 1.81698 -1.87049 -1.06747
8 4.29979 1.50362 0.816864
6 4.2755 -0.9702 -0.416329
1 0.037958 -2.62904 1.16769
1 -1.37378 -2.31152 2.20889
1 -0.085093 -1.10342 2.07318
1 4.2135 -1.92996 0.100792

1 5.11962 -0.390597 -0.050447
1 4.3736 -1.16109 -1.48684
1 -4.11875 1.90874 -0.062834
1 -3.45875 2.21428 -1.67974
1 -2.07441 0.551741 1.39259
1 -0.312556 -1.32558 -1.50684
1 -3.36892 -0.164053 -1.27053
1 -0.175643 1.72907 0.433229
1 1.98028 2.73488 1.19805
1 -3.82071 -1.54302 0.770031
1 -1.58847 2.95368 -0.706762

Conf 14
6 -2.37336 -0.468665 0.860935
6 -1.24849 -1.32538 0.250746
6 -0.529457 -0.346556 -0.715139
8 -1.40247 0.757685 -0.893894
6 -2.69157 0.486157 -0.298026
7 0.772623 0.158883 -0.247125
6 -3.32321 1.81236 0.082133
6 -0.407356 -2.12963 1.21402
8 -3.46194 -1.22294 1.32885
9 -1.93168 -2.24187 -0.592967
6 0.882598 1.30465 0.507869
6 2.07384 1.77789 0.925409
6 3.30947 1.09934 0.571355
7 3.12446 -0.065986 -0.224817
6 1.90402 -0.573467 -0.638912
8 -3.56839 2.63173 -1.04397
8 1.80403 -1.60076 -1.2971
8 4.42741 1.46395 0.902795
6 4.30771 -0.827301 -0.640087
1 0.294004 -2.76343 0.667546
1 -1.06713 -2.75473 1.82108
1 0.154065 -1.47189 1.88257
1 4.24701 -1.84876 -0.259058
1 5.17588 -0.315486 -0.231871
1 4.36143 -0.8682 -1.72984
1 -2.68248 2.31793 0.825064
1 -4.29004 1.61668 0.556203
1 -1.98505 0.098972 1.71573
1 -0.335003 -0.870419 -1.6533
1 -3.32904 -0.024431 -1.03285
1 -0.053935 1.80467 0.714387
1 2.14986 2.68113 1.5146
1 -3.62453 -1.90507 0.659034
1 -2.72387 2.73584 -1.50423

Conf 6
6 -2.32221 -0.21724 0.68968
6 -1.19918 -1.18949 0.30518
6 -0.418737 -0.394661 -0.781455
8 -1.2587 0.677874 -1.1852
6 -2.57719 0.497381 -0.645107
7 0.870305 0.167227 -0.344955

6 -3.29875 1.83277 -0.511726
6 -0.416567 -1.81923 1.4339
8 -3.45727 -0.83006 1.26192
9 -1.86464 -2.23167 -0.39225
6 0.97204 1.43588 0.180674
6 2.15211 1.9562 0.57325
6 3.3834 1.19637 0.436979
7 3.20842 -0.095275 -0.135739
6 1.99874 -0.647292 -0.522683
8 -4.63872 1.62495 -0.105329
8 1.90388 -1.77861 -0.981353
8 4.49084 1.59295 0.767044
6 4.38895 -0.945393 -0.324875
1 0.29856 -2.54324 1.03887
1 -1.11161 -2.32502 2.10918
1 0.125007 -1.05982 2.0039
1 4.28085 -1.86834 0.248745
1 5.2499 -0.377887 0.020188
1 4.49539 -1.20538 -1.37983
1 -3.32858 2.34354 -1.47864
1 -2.74482 2.48087 0.189255
1 -1.9503 0.505289 1.42656
1 -0.197512 -1.07326 -1.60816
1 -3.16727 -0.158602 -1.30072
1 0.041012 1.98446 0.225329
1 2.223 2.95528 0.980139
1 -3.64777 -1.61152 0.719251
1 -4.63101 1.00647 0.641563

Conf 12
6 -2.32499 -0.247223 0.771101
6 -1.46116 -1.37461 0.176695
6 -0.580897 -0.668648 -0.8866
8 -1.16286 0.590149 -1.13653
6 -2.39384 0.759231 -0.391752
7 0.854774 -0.515386 -0.609135
6 -2.48319 2.21795 0.021514
6 -0.809067 -2.32656 1.1505
8 -3.58004 -0.696337 1.22526
9 -2.37429 -2.14039 -0.612774
6 1.77117 -0.967421 -1.53861
6 3.0954 -0.755125 -1.42205
6 3.62665 0.003448 -0.302126
7 2.63252 0.467214 0.604636
6 1.27083 0.237273 0.499289
8 -2.53828 3.08551 -1.09854
8 0.476829 0.649244 1.33492
8 4.80913 0.247031 -0.119862
6 3.06023 1.24856 1.77101
1 -0.275383 -3.12282 0.623984
1 -1.58999 -2.7771 1.7686
1 -0.116264 -1.79256 1.80212
1 2.58548 2.23148 1.75495
1 4.14184 1.34281 1.71286
1 2.76674 0.738094 2.69052
1 -1.63014 2.44786 0.676945

1 -3.40658 2.36842 0.589867
1 -1.80728 0.193043 1.62323
1 -0.624038 -1.29556 -1.7845
1 -3.23855 0.508453 -1.04887
1 1.34641 -1.51456 -2.37259
1 3.79694 -1.12687 -2.15566
1 -3.92347 -1.28679 0.537914
1 -1.73324 2.92084 -1.60873

Conf 13
6 -2.40956 -0.001577 0.541752
6 -1.52647 -1.25146 0.450478
6 -0.583357 -0.935943 -0.748326
8 -1.13299 0.156537 -1.44289
6 -2.43762 0.494505 -0.912448
7 0.844413 -0.687932 -0.473284
6 -2.64225 1.99473 -1.08266
6 -0.904094 -1.7261 1.74459
8 -3.68863 -0.316746 1.05799
9 -2.3419 -2.29312 -0.037074
6 1.78806 -1.53968 -1.01262
6 3.11336 -1.34795 -0.871564
6 3.61913 -0.193492 -0.150708
7 2.59786 0.660496 0.357942
6 1.23649 0.454719 0.23067
8 -1.89339 2.77574 -0.173855
8 0.420896 1.2286 0.72639
8 4.79952 0.064486 0.023205
6 2.99938 1.86314 1.09783
1 -0.312964 -2.63265 1.5864
1 -1.70956 -1.95533 2.44699
1 -0.268582 -0.952058 2.17929
1 2.62218 2.75492 0.593244
1 4.08618 1.87055 1.12719
1 2.58529 1.83521 2.1074
1 -3.69965 2.22348 -0.904647
1 -2.42279 2.2546 -2.1303
1 -1.896 0.742338 1.15375
1 -0.59019 -1.83633 -1.37347
1 -3.21129 -0.057545 -1.46357
1 1.38325 -2.38293 -1.56027
1 3.83431 -2.03127 -1.29799
1 -3.99059 0.444101 1.56822
1 -0.990133 2.41872 -0.139819

Conf 69
6 2.93356 0.371649 0.172074
6 1.56577 0.865388 0.717307
6 0.684639 0.744875 -0.556439
8 1.1808 -0.317812 -1.31795
6 2.56685 -0.582999 -0.981936
7 -0.770183 0.612941 -0.379956
6 2.71 -2.07657 -0.708999
6 1.11732 0.195737 1.99791
8 3.66207 1.44994 -0.390129
9 1.66185 2.25045 0.998214

6 -1.55748 1.71675 -0.650107
6 -2.89935 1.70575 -0.551658
6 -3.59995 0.492658 -0.170879
7 -2.73976 -0.613016 0.078752
6 -1.35485 -0.601404 0.011497
8 1.96795 -2.51842 0.405488
8 -0.705977 -1.60254 0.290766
8 -4.81192 0.385894 -0.067002
6 -3.34732 -1.89119 0.469423
1 0.099593 0.492809 2.26245
1 1.78768 0.518818 2.79969
1 1.17459 -0.887398 1.90777
1 -3.05506 -2.67149 -0.235583
1 -4.4249 -1.74714 0.460843
1 -3.0055 -2.17967 1.46544
1 3.76548 -2.29751 -0.510971
1 2.42811 -2.61587 -1.62668
1 3.50906 -0.139455 0.955366
1 0.818612 1.69921 -1.08383
1 3.19001 -0.309708 -1.84239
1 -1.01024 2.60438 -0.944759
1 -3.49082 2.58495 -0.765297
1 3.54002 2.20823 0.199648
1 1.02772 -2.33411 0.224269

Compound 1b: 2’MeCl Uracil

Conf 4
6 2.01731 -0.336254 0.754062
6 1.20759 0.937726 0.426196
6 0.307415 0.462296 -0.753849
8 0.930738 -0.688241 -1.30408
6 2.163 -0.993126 -0.625644
7 -1.07135 0.124116 -0.354523
6 2.36689 -2.50343 -0.609376
6 0.513474 1.59997 1.60243
8 3.21282 -0.163064 1.46285
17 2.39986 2.17241 -0.288857
6 -1.39245 -1.09257 0.216829
6 -2.65122 -1.4265 0.573999
6 -3.75026 -0.497795 0.352191
7 -3.32857 0.713748 -0.238988
6 -2.05224 1.10146 -0.593659
8 1.24226 -3.20583 -0.093089
8 -1.79313 2.19729 -1.06504
8 -4.92558 -0.677064 0.621795
1 1.38535 -0.974968 1.38261
1 0.202322 1.24948 -1.49981
1 3.00698 -0.528516 -1.15676
1 3.21567 -2.72215 0.046523
1 2.6199 -2.85559 -1.6179
1 -0.030659 2.49236 1.28684
1 1.25559 1.87699 2.35424
1 -0.191519 0.899739 2.06073
1 3.72233 0.52811 1.01101
1 -0.558065 -1.77104 0.345607
1 -2.87388 -2.38629 1.01936

1 -4.04913 1.40599 -0.406428
1 0.583074 -3.22562 -0.799462

Conf 3
6 2.12957 -0.2561 0.556668
6 1.22155 0.989479 0.410521
6 0.249927 0.588274 -0.750015
8 0.761068 -0.577533 -1.34392
6 2.08018 -0.89292 -0.840675
7 -1.16936 0.386142 -0.390417
6 2.25541 -2.40723 -0.866498
6 0.611284 1.49366 1.70416
8 3.41479 -0.036103 1.06903
17 2.27054 2.37108 -0.286604
6 -2.1254 1.22245 -0.946199
6 -3.45136 1.07988 -0.749257
6 -3.95333 -0.015484 0.067092
7 -2.91413 -0.829661 0.573645
6 -1.55385 -0.696814 0.407058
8 1.49801 -3.08701 0.108772
8 -0.755022 -1.4597 0.937069
8 -5.11658 -0.266788 0.324131
1 1.63214 -0.934223 1.25343
1 0.221023 1.40316 -1.47875
1 2.83468 -0.428634 -1.49431
1 3.30936 -2.62998 -0.666856
1 2.02835 -2.75658 -1.8868
1 -0.009908 2.37872 1.54671
1 1.41184 1.74543 2.4033
1 0.006263 0.700765 2.15104
1 3.83583 0.652173 0.531597
1 -1.72466 2.01943 -1.56209
1 -4.16473 1.75709 -1.19778
1 -3.19858 -1.61146 1.15274
1 0.601671 -2.7137 0.119664

Conf 14
6 1.89556 -0.177091 0.731647
6 0.966652 1.00266 0.378009
6 0.118077 0.393737 -0.786884
8 0.831595 -0.730878 -1.27177
6 2.11012 -0.853037 -0.627508
7 -1.23285 -0.049277 -0.379909
6 2.53397 -2.31565 -0.531628
6 0.218042 1.61223 1.5496
8 3.0669 0.11309 1.45502
17 2.02851 2.32616 -0.367316
6 -1.49701 -1.34529 0.015893
6 -2.72985 -1.76977 0.366424
6 -3.86329 -0.857477 0.314723
7 -3.50364 0.434029 -0.133175
6 -2.25612 0.905507 -0.485239
8 3.85088 -2.41 -0.023267
8 -2.0467 2.05366 -0.844874
8 -5.02001 -1.11017 0.603927
1 1.31629 -0.854942 1.37381

1 -0.03889 1.13616 -1.56992
1 2.87291 -0.307869 -1.19967
1 2.53702 -2.77033 -1.52645
1 1.80747 -2.87259 0.085419
1 -0.40236 2.44918 1.22663
1 0.934987 1.95726 2.29789
1 -0.422095 0.855335 2.01381
1 3.53274 0.82382 0.985078
1 -0.639091 -2.00341 -0.004577
1 -2.90921 -2.79225 0.668441
1 -4.25412 1.11161 -0.198234
1 3.89678 -1.87052 0.781281

Conf 12
6 1.95095 -0.153453 0.617728
6 1.04414 1.07616 0.393807
6 0.082261 0.582293 -0.73942
8 0.651163 -0.581388 -1.29617
6 1.96173 -0.826634 -0.762624
7 -1.31851 0.317262 -0.363094
6 2.2401 -2.32597 -0.706556
6 0.424143 1.67194 1.64346
8 3.22216 0.075959 1.17855
17 2.09774 2.40364 -0.390745
6 -2.32765 0.974175 -1.05075
6 -3.63998 0.714286 -0.886574
6 -4.06668 -0.329652 0.033636
7 -2.97814 -0.96455 0.671433
6 -1.62852 -0.709859 0.54289
8 3.58598 -2.55785 -0.323887
8 -0.785088 -1.31527 1.18597
8 -5.21068 -0.671924 0.274296
1 1.4182 -0.798477 1.31964
1 0.008862 1.36616 -1.49808
1 2.72042 -0.351642 -1.40256
1 2.11009 -2.76628 -1.69957
1 1.51685 -2.80263 -0.02809
1 -0.20595 2.53401 1.41178
1 1.21828 1.99081 2.32225
1 -0.173953 0.910646 2.14907
1 3.68662 0.71345 0.613617
1 -1.98204 1.73322 -1.74336
1 -4.39601 1.25668 -1.4369
1 -3.2111 -1.70112 1.3273
1 3.7535 -2.05165 0.485551

Conf 101
6 2.6556 0.26161 -0.000786
6 1.26634 0.77734 0.497717
6 0.352576 0.368157 -0.69887
8 0.880707 -0.816091 -1.22203
6 2.29415 -0.916774 -0.9377
7 -1.08336 0.197133 -0.42557
6 2.55992 -2.32982 -0.425373
6 0.907498 0.236519 1.87426
8 3.36761 1.18307 -0.800302

17 1.24032 2.61869 0.618131
6 -1.94306 1.20572 -0.838152
6 -3.28126 1.16597 -0.684701
6 -3.90799 0.004584 -0.073622
7 -2.96593 -0.977673 0.301492
6 -1.5904 -0.963787 0.183039
8 1.85158 -2.64581 0.751403
8 -0.912329 -1.89503 0.592401
8 -5.09767 -0.172485 0.120427
1 3.25009 -0.069958 0.860486
1 0.408143 1.17362 -1.44168
1 2.85294 -0.75981 -1.86859
1 3.62911 -2.43242 -0.206731
1 2.32319 -3.03015 -1.24141
1 -0.113621 0.502966 2.15414
1 1.58709 0.666437 2.61419
1 1.0222 -0.846933 1.88708
1 3.50122 1.99168 -0.284982
1 -1.44878 2.05373 -1.2967
1 -3.91117 1.97844 -1.01888
1 -3.3404 -1.8134 0.73541
1 0.900146 -2.55449 0.557904

Conf 27
6 2.13503 0.243586 -0.537693
6 1.22752 -1.00884 -0.399769
6 0.249148 -0.595607 0.755362
8 0.760285 0.568172 1.34993
6 2.08205 0.876419 0.852724
7 -1.16947 -0.383537 0.390285
6 2.26641 2.38962 0.876829
6 0.593323 -1.48213 -1.69839
8 3.47431 0.015228 -0.901716
17 2.23332 -2.40601 0.269035
6 -2.13182 -1.21504 0.941204
6 -3.45693 -1.06416 0.7423
6 -3.95055 0.036499 -0.071225
7 -2.905 0.8457 -0.572754
6 -1.54572 0.703325 -0.40433
8 1.53365 3.07083 -0.118926
8 -0.742532 1.46351 -0.933363
8 -5.11163 0.296508 -0.330612
1 1.63596 0.921052 -1.23811
1 0.212856 -1.41062 1.48343
1 2.83622 0.401734 1.4957
1 3.32527 2.60542 0.699261
1 2.01861 2.74479 1.88982
1 -0.064161 -2.34033 -1.53934
1 1.37679 -1.78479 -2.39836
1 0.020999 -0.668122 -2.15037
1 3.53399 -0.008949 -1.86445
1 -1.7369 -2.01612 1.55546
1 -4.17496 -1.73857 1.18757
1 -3.18351 1.63129 -1.14943
1 0.630353 2.71426 -0.132032

Conf 115
6 -1.98169 0.346772 0.725942
6 -1.2351 -0.961861 0.377025
6 -0.310859 -0.48911 -0.788911
8 -0.926407 0.647405 -1.35466
6 -2.11701 1.02278 -0.638377
7 1.06859 -0.134859 -0.371074
6 -2.251 2.53307 -0.60981
6 -0.554403 -1.64999 1.55012
8 -3.24837 0.235154 1.323
17 -2.43596 -2.14716 -0.357422
6 1.42499 1.15609 -0.030828
6 2.68993 1.49842 0.301453
6 3.75343 0.507692 0.287864
7 3.29809 -0.772487 -0.100733
6 2.01767 -1.16454 -0.433551
8 -1.21267 3.08632 0.194446
8 1.72871 -2.31101 -0.744448
8 4.92876 0.685158 0.563814
1 -1.30019 0.943878 1.35012
1 -0.195559 -1.28399 -1.52559
1 -2.9979 0.609588 -1.14696
1 -3.24263 2.77125 -0.199129
1 -2.20243 2.90827 -1.6411
1 -0.017025 -2.54006 1.22034
1 -1.30045 -1.9482 2.29247
1 0.155379 -0.966137 2.02654
1 -3.14345 -0.117662 2.21607
1 0.617298 1.87864 -0.062651
1 2.94361 2.51586 0.565242
1 3.99557 -1.50617 -0.136732
1 -1.24367 4.04759 0.117416

Compound 1b: 2’MeCl Uracil anion

Conf 113
6 2.33735 0.461193 0.400226
6 1.30756 1.5526 0.109895
6 0.335572 0.904366 -0.917429
8 1.02959 -0.205266 -1.48328
6 2.3179 -0.391165 -0.882266
7 -0.994003 0.517709 -0.471165
6 2.56946 -1.88889 -0.682037
6 0.671585 2.18284 1.34519
8 3.62346 1.05085 0.646638
6 -2.09331 1.02006 -1.13693
6 -3.34366 0.626749 -0.831223
6 -3.54957 -0.389105 0.220184
7 -2.42579 -0.902338 0.835332
6 -1.19752 -0.501534 0.535141
8 1.9528 -2.45619 0.443325
8 -0.15007 -0.926306 1.09138
8 -4.696 -0.756084 0.517549
1 2.0198 -0.141172 1.25159
1 0.148501 1.63084 -1.72031
1 3.09212 0.005855 -1.56214

1 3.65627 -2.03677 -0.573371
1 2.27596 -2.3928 -1.62099
1 -0.055022 2.95725 1.07564
1 1.45028 2.6475 1.95998
1 0.163154 1.41903 1.93659
1 4.1331 0.395829 1.13905
1 -1.87592 1.75314 -1.91083
1 -4.20571 1.03128 -1.34953
1 1.07051 -2.02713 0.578363
1 1.85509 2.33085 -0.442654

Conf 3
6 2.36095 0.454296 0.413164
6 1.30845 1.53841 0.130811
6 0.343599 0.893091 -0.904938
8 1.0414 -0.221121 -1.46299
6 2.32505 -0.412551 -0.859711
7 -0.990557 0.515968 -0.474829
6 2.57277 -1.90836 -0.648749
6 0.672953 2.15779 1.37147
8 3.66181 0.968149 0.704517
6 -2.08092 1.01234 -1.16035
6 -3.33493 0.627284 -0.860515
6 -3.55446 -0.37055 0.206236
7 -2.43893 -0.879841 0.839233
6 -1.20648 -0.489376 0.543522
8 1.904 -2.48064 0.441031
8 -0.163296 -0.908873 1.11014
8 -4.70547 -0.725682 0.499282
1 2.07595 -0.142872 1.2771
1 0.170314 1.61792 -1.71269
1 3.10275 -0.038902 -1.55653
1 3.65338 -2.04424 -0.48856
1 2.32386 -2.41016 -1.60249
1 -0.056538 2.93242 1.11083
1 1.45193 2.6164 1.99062
1 0.169975 1.38484 1.95485
1 3.9343 1.49298 -0.061715
1 -1.8531 1.7328 -1.94316
1 -4.19109 1.02617 -1.39271
1 1.03714 -2.02297 0.574113
1 1.83804 2.33377 -0.422039

Compound 1c: 2'MeAlkyne Uracil

Conf 16
6 2.03897 -0.308621 0.737887
6 1.27091 1.00705 0.394292
6 0.351628 0.525704 -0.786814
8 0.974603 -0.609167 -1.35344
6 2.16688 -0.975818 -0.639884
7 -1.02887 0.167841 -0.3911
6 2.31518 -2.48532 -0.619653
6 0.541294 1.607 1.60697
8 3.25816 -0.141681 1.41694
6 2.24282 1.97326 -0.138534

6 -1.38177 -1.11965 -0.036621
6 -2.64692 -1.46187 0.296305
6 -3.71189 -0.473393 0.272937
7 -3.25806 0.804811 -0.124013
6 -1.97743 1.19651 -0.45692
8 1.26734 -3.04863 0.163047
8 -1.68904 2.34211 -0.773184
8 -4.888 -0.650441 0.547241
1 1.41077 -0.92706 1.38456
1 0.241909 1.3164 -1.52936
1 3.0428 -0.558223 -1.15956
1 3.30172 -2.7149 -0.190877
1 2.28919 -2.85967 -1.65253
1 0.013841 2.52228 1.33278
1 1.27318 1.83625 2.3855
1 -0.177782 0.890984 2.0134
1 3.74099 0.572633 0.970029
1 -0.5719 -1.84007 -0.059833
1 -2.89856 -2.47715 0.570107
1 -3.95617 1.53763 -0.162725
1 1.33335 -4.01014 0.11984
6 3.08766 2.73623 -0.546413
1 3.79668 3.43836 -0.921757

Conf 4
6 1.97247 0.023161 0.995359
6 1.08441 1.1479 0.364973
6 0.284986 0.338928 -0.725411
8 1.00567 -0.858171 -0.958115
6 2.24003 -0.883215 -0.209187
7 -1.09827 -0.015292 -0.344448
6 2.57493 -2.33011 0.105648
6 0.227999 1.88573 1.4071
8 3.11912 0.463978 1.67335
6 1.97599 2.09717 -0.316112
6 -1.40637 -1.1909 0.307616
6 -2.66561 -1.53279 0.656593
6 -3.78029 -0.654861 0.332507
7 -3.37271 0.512884 -0.350482
6 -2.09668 0.901529 -0.705227
8 2.79751 -3.09457 -1.06354
8 -1.84949 1.95378 -1.2741
8 -4.95801 -0.842364 0.586514
1 1.37116 -0.515789 1.73801
1 0.190597 0.925983 -1.6394
1 3.04279 -0.454941 -0.826189
1 1.7742 -2.76196 0.730479
1 3.49989 -2.35569 0.690007
1 -0.371832 2.66726 0.937116
1 0.887136 2.34304 2.1491
1 -0.437218 1.18872 1.92368
1 3.56259 1.11138 1.10138
1 -0.557667 -1.83436 0.495706
1 -2.87752 -2.46456 1.1623
1 -4.10627 1.16463 -0.602379
1 2.00691 -3.00218 -1.61339

6 2.75311 2.85968 -0.841761
1 3.40387 3.55077 -1.32736

Conf 179
6 -1.95059 0.140303 0.741154
6 -1.00042 -1.04734 0.395306
6 -0.149592 -0.409074 -0.774787
8 -0.862009 0.726498 -1.23812
6 -2.15743 0.798345 -0.625956
7 1.20762 0.025601 -0.389778
6 -2.64613 2.24135 -0.555818
6 -0.19676 -1.5471 1.6071
8 -3.141 -0.188278 1.42407
6 -1.81997 -2.13794 -0.149903
6 1.47944 1.3126 0.026726
6 2.71794 1.72871 0.369298
6 3.84762 0.814637 0.290186
7 3.47808 -0.469377 -0.170005
6 2.22404 -0.932415 -0.51205
8 -3.97762 2.2827 -0.077905
8 2.00675 -2.07742 -0.87852
8 5.00937 1.05957 0.567916
1 -1.41391 0.833657 1.40032
1 -0.00733 -1.13799 -1.57361
1 -2.88378 0.213305 -1.20911
1 -2.64498 2.68812 -1.55446
1 -1.95818 2.83299 0.072825
1 0.458742 -2.37295 1.32521
1 -0.890515 -1.89314 2.37732
1 0.408117 -0.740163 2.02937
1 -3.54378 -0.940711 0.959088
1 0.621694 1.97124 0.029283
1 2.90319 2.7454 0.68689
1 4.22449 -1.14974 -0.249306
1 -4.02141 1.72782 0.716654
6 -2.5462 -3.0109 -0.564953
1 -3.14777 -3.80295 -0.949342

Conf 11
6 2.17553 -0.206863 0.574594
6 1.26939 1.06452 0.425607
6 0.297266 0.644554 -0.744571
8 0.821092 -0.509929 -1.34726
6 2.13217 -0.827767 -0.828917
7 -1.1242 0.423032 -0.397209
6 2.31897 -2.34025 -0.87945
6 0.598079 1.4709 1.74823
8 3.47214 0.035908 1.06042
6 2.09978 2.17225 -0.072359
6 -2.0804 1.27559 -0.923595
6 -3.407 1.13039 -0.729072
6 -3.90905 0.011556 0.053844
7 -2.87022 -0.819016 0.531104
6 -1.50857 -0.681783 0.368896
8 1.55762 -3.04493 0.074404
8 -0.71387 -1.46252 0.87819

8 -5.07272 -0.2458 0.305737
1 1.7045 -0.891783 1.27976
1 0.259834 1.46073 -1.47394
1 2.89372 -0.350651 -1.46678
1 3.3732 -2.55704 -0.673758
1 2.10542 -2.67314 -1.9085
1 -0.045792 2.34605 1.62351
1 1.37655 1.72027 2.47361
1 0.012034 0.641881 2.1479
1 3.85547 0.755267 0.534452
1 -1.68005 2.09008 -1.51649
1 -4.11957 1.82227 -1.15591
1 -3.15374 -1.61739 1.08742
1 0.663843 -2.66527 0.096865
6 2.81102 3.06134 -0.480093
1 3.41822 3.86213 -0.836071

Conf 12
6 2.62573 0.45323 0.073768
6 1.19022 0.757033 0.641821
6 0.330253 0.201056 -0.544446
8 0.985357 -1.00083 -0.914388
6 2.3967 -0.714422 -0.921881
7 -1.07382 -0.08982 -0.283865
6 3.14815 -2.00877 -0.640328
6 0.979337 -0.003571 1.96702
8 3.17847 1.51502 -0.677222
6 0.989264 2.18955 0.849757
6 -1.46685 -1.26578 0.325689
6 -2.75722 -1.58075 0.564971
6 -3.81861 -0.668363 0.166166
7 -3.32907 0.489288 -0.475678
6 -2.02208 0.838868 -0.755992
8 2.78322 -2.60943 0.593652
8 -1.718 1.85821 -1.34939
8 -5.01709 -0.825426 0.32856
1 3.28947 0.159526 0.898174
1 0.34238 0.933375 -1.35654
1 2.68645 -0.338005 -1.91256
1 4.21888 -1.7896 -0.584364
1 2.99282 -2.70227 -1.47908
1 -0.056432 0.064529 2.30728
1 1.61441 0.437477 2.7396
1 1.26906 -1.05119 1.86214
1 3.10392 2.32412 -0.148537
1 -0.652861 -1.92922 0.580442
1 -3.03252 -2.51201 1.04004
1 -4.02553 1.15029 -0.798323
1 1.87051 -2.90892 0.489291
6 0.86937 3.37623 1.04455
1 0.718478 4.41951 1.20292

Conf 41
6 -2.63493 0.380689 -0.061464
6 -1.20567 0.753939 -0.597794
6 -0.332719 0.264078 0.611956

8 -0.954009 -0.925579 1.05302
6 -2.37502 -0.785463 0.925785
7 1.06672 -0.047902 0.329304
6 -2.97364 -2.1331 0.564806
6 -0.907633 0.003548 -1.91261
8 -3.24045 1.4129 0.693224
6 -1.08953 2.19584 -0.814365
6 1.43222 -1.28189 -0.175159
6 2.7137 -1.61907 -0.435015
6 3.78829 -0.674553 -0.173264
7 3.32375 0.54282 0.369937
6 2.0276 0.921059 0.665365
8 -2.5347 -2.52501 -0.729484
8 1.74446 1.997 1.16355
8 4.98053 -0.847993 -0.367972
1 -3.2733 0.072615 -0.90003
1 -0.325884 1.03837 1.38411
1 -2.79818 -0.47628 1.89103
1 -4.06945 -2.03846 0.609739
1 -2.66469 -2.86147 1.32838
1 0.119174 0.181264 -2.23977
1 -1.57655 0.371395 -2.69531
1 -1.08061 -1.06757 -1.79713
1 -3.15552 2.23646 0.188131
1 0.603949 -1.96193 -0.322158
1 2.97164 -2.59372 -0.825259
1 4.03141 1.23131 0.59564
1 -2.90407 -3.39467 -0.92566
6 -1.04627 3.38686 -1.01561
1 -0.956263 4.436 -1.18092

Conf 115
6 1.98869 0.306113 -0.747126
6 1.27704 -1.02906 -0.401545
6 0.356039 -0.558485 0.784471
8 0.992927 0.560903 1.36946
6 2.1779 0.929484 0.641311
7 -1.02118 -0.174402 0.392475
6 2.35468 2.43513 0.679624
6 0.546607 -1.64243 -1.60666
8 3.19843 0.107243 -1.44936
6 2.24671 -1.99162 0.140996
6 -1.35584 1.12327 0.062306
6 -2.61399 1.49193 -0.269759
6 -3.69323 0.51879 -0.270068
7 -3.2598 -0.771969 0.105878
6 -1.98645 -1.18892 0.44018
8 1.35804 3.05395 -0.131979
8 -1.71994 -2.34281 0.742557
8 -4.86524 0.718839 -0.547096
1 1.29313 0.922862 -1.32821
1 0.23642 -1.36027 1.51272
1 3.05757 0.467856 1.11089
1 3.36513 2.66839 0.31011
1 2.28872 2.76978 1.72327
1 0.029617 -2.56028 -1.32189

1 1.28 -1.87485 -2.38267
1 -0.182922 -0.938483 -2.01749
1 3.30624 0.834515 -2.07354
1 -0.536102 1.8312 0.103761
1 -2.84954 2.51616 -0.523896
1 -3.96899 -1.49465 0.131388
1 1.32916 3.99457 0.080904
6 3.01865 -2.79844 0.599814
1 3.70387 -3.51159 0.996234

Conf 10
6 2.59816 0.627829 0.187272
6 1.12818 0.941915 0.644873
6 0.3562 0.167633 -0.47741
8 1.06863 -1.04989 -0.603454
6 2.47942 -0.744058 -0.517021
7 -1.04945 -0.137517 -0.236253
6 3.16128 -1.91835 0.170557
6 0.867271 0.379043 2.05652
8 3.08681 1.53867 -0.774451
6 0.866608 2.37962 0.62776
6 -1.43504 -1.24114 0.501355
6 -2.72595 -1.55961 0.733587
6 -3.79361 -0.734044 0.188762
7 -3.30875 0.347535 -0.577786
6 -2.00111 0.69806 -0.852561
8 2.96927 -3.12306 -0.544142
8 -1.69783 1.64593 -1.55501
8 -4.99311 -0.902675 0.330126
1 3.25889 0.585964 1.06663
1 0.390045 0.758421 -1.39667
1 2.87708 -0.633671 -1.53257
1 2.80265 -2.0005 1.21018
1 4.24047 -1.73656 0.205634
1 -0.173175 0.528588 2.35343
1 1.50069 0.896779 2.78134
1 1.09641 -0.688371 2.10692
1 2.93819 2.43557 -0.436891
1 -0.614375 -1.84686 0.859425
1 -2.99637 -2.43358 1.30964
1 -4.00863 0.943305 -1.00366
1 2.01653 -3.21516 -0.685189
6 0.699132 3.57633 0.637022
1 0.505594 4.62468 0.631149

Conf 156
6 2.13318 -0.200324 0.577914
6 1.27396 1.08543 0.423905
6 0.296529 0.671382 -0.74709
8 0.833793 -0.462681 -1.37491
6 2.13912 -0.782679 -0.840442
7 -1.12184 0.419482 -0.393048
6 2.34262 -2.29159 -0.927909
6 0.594799 1.50052 1.74027
8 3.42576 0.087979 1.07468
6 2.09755 2.1964 -0.077886

6 -2.08849 1.28482 -0.877256
6 -3.41286 1.12516 -0.677144
6 -3.9014 -0.024944 0.066902
7 -2.85271 -0.865329 0.503803
6 -1.49301 -0.712748 0.33715
8 1.64324 -3.02461 0.055825
8 -0.693201 -1.50856 0.816502
8 -5.061 -0.300488 0.319277
1 1.5989 -0.887868 1.2364
1 0.240843 1.50196 -1.45774
1 2.91022 -0.272052 -1.435
1 3.40867 -2.50356 -0.783894
1 2.08213 -2.61219 -1.94918
1 -0.038012 2.38212 1.60607
1 1.37075 1.74838 2.46877
1 -0.006053 0.681509 2.14081
1 3.72235 -0.682926 1.57395
1 -1.69807 2.12348 -1.44232
1 -4.13248 1.82881 -1.07171
1 -3.12639 -1.68466 1.03374
1 0.731239 -2.69022 0.092202
6 2.72774 3.13318 -0.506337
1 3.31056 3.94738 -0.870808

Compound 1c: 2'MeAlkyne Uracil anion

Conf 18
6 -2.09512 0.269858 0.546294
6 -1.30324 -1.05612 0.422102
6 -0.265075 -0.717492 -0.727958
8 -0.797862 0.392028 -1.43803
6 -2.02945 0.856035 -0.870738
7 1.12057 -0.492025 -0.359941
6 -2.06502 2.38497 -0.936623
6 -0.663313 -1.4866 1.7527
8 -3.43217 0.067461 1.00183
6 -2.1784 -2.13006 -0.077119
6 2.08721 -1.32318 -0.893607
6 3.39774 -1.10972 -0.677136
6 3.82387 0.058648 0.118492
7 2.8345 0.883282 0.614535
6 1.5436 0.655842 0.418269
8 -1.32048 3.03991 0.058214
8 0.613731 1.35998 0.892827
8 5.02824 0.273541 0.316366
1 -1.54359 0.917324 1.22595
1 -0.237125 -1.57906 -1.40454
1 -2.87573 0.454114 -1.45253
1 -3.11322 2.7036 -0.827519
1 -1.74973 2.67306 -1.95553
1 -0.060574 -2.39079 1.62826
1 -1.45405 -1.69688 2.47946
1 -0.030807 -0.680892 2.12779
1 -3.67046 0.864383 1.49045
1 1.71306 -2.15294 -1.48873

1 4.15242 -1.76775 -1.09272
1 -0.488389 2.53144 0.222151
6 -2.85128 -3.04342 -0.495353
1 -3.46815 -3.8303 -0.860152

Compound 1d: 2'MeAzide Uracil

Conf 102
6 1.76466 -0.44939 0.621826
6 0.970993 0.803232 0.185022
6 -0.012538 0.188506 -0.868989
8 0.537689 -1.05854 -1.27401
6 1.83389 -1.23651 -0.689849
7 -1.38853 -0.048255 -0.390848
6 2.11213 -2.7206 -0.513894
6 0.325326 1.5725 1.33476
8 3.00365 -0.111521 1.18439
7 1.84069 1.68191 -0.646417
6 -1.77172 -1.2426 0.181674
6 -3.03084 -1.49071 0.602614
6 -4.0683 -0.48252 0.444169
7 -3.59178 0.6957 -0.171366
6 -2.3116 0.988491 -0.598847
8 3.36528 -2.79014 0.168387
8 -2.00203 2.05603 -1.1032
8 -5.23769 -0.576803 0.777302
1 1.14087 -1.01494 1.33807
1 -0.101383 0.875839 -1.7108
1 2.60737 -0.790228 -1.32782
1 2.15105 -3.22313 -1.48948
1 1.30639 -3.18169 0.077407
1 -0.242931 2.42434 0.955079
1 1.10262 1.93506 2.01368
1 -0.343957 0.926756 1.90939
1 3.53384 -0.924375 1.18594
1 -0.983697 -1.98043 0.243368
1 -3.30174 -2.43971 1.04393
1 3.59503 -3.71531 0.316255
7 2.70374 2.34428 -0.055834
7 3.52147 3.03176 0.341878
1 -4.26986 1.43533 -0.310776

Conf 108
6 -1.78702 -0.657177 -0.606648
6 -1.2117 0.715169 -0.163939
6 -0.162719 0.274936 0.903586
8 -0.57824 -0.987862 1.3838
6 -1.75204 -1.45405 0.698012
7 1.22806 0.164955 0.407669
6 -1.69199 -2.96113 0.542174
6 -0.682397 1.58024 -1.30439
8 -3.10726 -0.614804 -1.10727
7 -2.23216 1.43701 0.647554
6 1.74246 -1.02396 -0.072491
6 3.02396 -1.15049 -0.484341
6 3.93907 -0.02352 -0.412755

7 3.33093 1.13661 0.117528
6 2.02646 1.30989 0.533671
8 -0.644554 -3.29962 -0.36375
8 1.59736 2.3709 0.963451
8 5.11193 -0.004736 -0.749314
1 -1.09996 -1.11529 -1.32911
1 -0.136516 1.01125 1.70764
1 -2.64611 -1.20095 1.28356
1 -2.66848 -3.29851 0.165575
1 -1.52736 -3.40966 1.5311
1 -0.236175 2.49696 -0.914479
1 -1.49995 1.84975 -1.98201
1 0.07097 1.04149 -1.88549
1 -3.07609 -0.458347 -2.05918
1 1.0453 -1.85408 -0.081283
1 3.40198 -2.09231 -0.857436
1 -0.518646 -4.25601 -0.34381
7 -3.18621 1.93297 0.037149
7 -4.10173 2.46544 -0.38792
1 3.92043 1.95635 0.198442

Conf 116
6 -2.69291 0.274378 0.032149
6 -1.25349 0.739577 -0.360123
6 -0.402638 0.054585 0.764277
8 -1.06231 -1.15353 1.06833
6 -2.47155 -1.03982 0.815527
7 0.982958 -0.282684 0.41814
6 -2.9557 -2.32309 0.162484
6 -0.88658 0.306421 -1.77979
8 -3.33456 1.17238 0.91364
7 -1.24731 2.21627 -0.21489
6 1.2978 -1.49019 -0.178486
6 2.56634 -1.85468 -0.463393
6 3.67734 -0.975088 -0.133383
7 3.25885 0.225628 0.482476
6 1.97627 0.638855 0.780079
8 -2.32966 -2.48361 -1.10543
8 1.72107 1.71435 1.29915
8 4.86211 -1.18383 -0.335218
1 -3.29191 0.110864 -0.874619
1 -0.351311 0.730389 1.62285
1 -3.00088 -0.92905 1.77041
1 -4.0508 -2.26583 0.068697
1 -2.71549 -3.16136 0.831772
1 0.146008 0.568614 -2.02617
1 -1.54803 0.807911 -2.49216
1 -1.019 -0.769926 -1.89841
1 -3.10629 2.06932 0.620172
1 0.442088 -2.12069 -0.382204
1 2.78784 -2.80523 -0.92842
1 -2.63173 -3.31454 -1.49227
7 -0.30153 2.83951 -0.711173
7 0.502378 3.52776 -1.13293
1 3.98959 0.882433 0.729034

Conf 119
6 1.80456 0.415939 -0.494934
6 1.04429 -0.888617 -0.167612
6 -0.022381 -0.395368 0.870297
8 0.393119 0.862318 1.35453
6 1.67198 1.21268 0.809234
7 -1.42783 -0.315936 0.430305
6 1.72976 2.71645 0.594426
6 0.502382 -1.62714 -1.38927
8 3.13798 0.157269 -0.86963
7 1.89317 -1.78965 0.672398
6 -2.37765 -1.07322 1.09553
6 -3.70248 -1.00239 0.852901
6 -4.2111 -0.068201 -0.139488
7 -3.18205 0.679638 -0.751618
6 -1.8185 0.616892 -0.542013
8 2.99953 2.95948 -0.022703
8 -1.03178 1.30809 -1.17094
8 -5.37534 0.106232 -0.45558
1 1.25489 0.932407 -1.289
1 -0.032844 -1.12449 1.68572
1 2.47274 0.899707 1.4934
1 1.64014 3.25008 1.55065
1 0.895462 3.01169 -0.05448
1 -0.076935 -2.50654 -1.09386
1 1.33744 -1.95843 -2.01384
1 -0.124244 -0.967355 -1.99148
1 3.57071 1.02481 -0.901998
1 -1.97298 -1.74776 1.84145
1 -4.40887 -1.6185 1.39145
1 3.02332 3.8733 -0.33037
7 2.85268 -2.34307 0.119601
7 3.74694 -2.94596 -0.249366
1 -3.47259 1.34666 -1.45672

Conf 124
6 -1.9385 0.565973 0.422931
6 -1.2078 -0.778844 0.157641
6 -0.12107 -0.365862 -0.891699
8 -0.43735 0.92095 -1.36089
6 -1.7317 1.34334 -0.879575
7 1.29089 -0.396272 -0.459191
6 -1.71674 2.86028 -0.726544
6 -0.700555 -1.47178 1.42018
8 -3.31489 0.394461 0.707489
7 -2.08671 -1.68825 -0.638279
6 2.16138 -1.28105 -1.0746
6 3.48344 -1.3369 -0.8142
6 4.07623 -0.415809 0.14317
7 3.1226 0.465654 0.703007
6 1.76648 0.53072 0.472613
8 -0.967822 3.31205 0.382445
8 1.04533 1.32866 1.06176
8 5.24697 -0.348716 0.471999
1 -1.42224 1.09303 1.22796
1 -0.17162 -1.09244 -1.70901

1 -2.50662 1.04925 -1.60121
1 -2.74765 3.19955 -0.575976
1 -1.35944 3.2958 -1.67298
1 -0.151699 -2.38674 1.1798
1 -1.55134 -1.7444 2.05318
1 -0.055403 -0.805273 1.99521
1 -3.48793 0.721818 1.59722
1 1.69458 -1.94328 -1.79464
1 4.12774 -2.04692 -1.31361
1 -0.119454 2.83837 0.389635
7 -3.05058 -2.19736 -0.051907
7 -3.95149 -2.76552 0.355353
1 3.47323 1.13392 1.37953

Conf 141
6 2.04067 0.19764 -0.993726
6 1.01293 -0.860942 -0.510134
6 0.242972 -0.075726 0.617807
8 0.958868 1.11797 0.859287
6 2.26975 1.03259 0.272973
7 -1.15059 0.283867 0.292688
6 2.82254 2.4162 0.013639
6 0.177278 -1.44459 -1.64557
8 3.19693 -0.332455 -1.58314
7 1.86918 -1.9007 0.134079
6 -1.50318 1.52667 -0.190664
6 -2.78017 1.86622 -0.472246
6 -3.85994 0.915843 -0.252588
7 -3.40355 -0.319348 0.263661
6 -2.10972 -0.704692 0.542047
8 3.14624 3.01819 1.25468
8 -1.80905 -1.81523 0.95821
8 -5.04759 1.09338 -0.463778
1 1.55832 0.826768 -1.7522
1 0.186927 -0.694464 1.51683
1 2.94849 0.50183 0.955189
1 2.07506 3.00178 -0.546618
1 3.707 2.29162 -0.630673
1 -0.491986 -2.23165 -1.29037
1 0.850907 -1.85869 -2.40081
1 -0.427794 -0.667247 -2.11926
1 3.45813 -1.09206 -1.0365
1 -0.673451 2.21172 -0.299979
1 -3.03252 2.84899 -0.845486
1 3.52739 3.88726 1.07801
7 1.3159 -2.94795 0.499405
7 0.938036 -3.95479 0.873706
1 -4.11136 -1.02351 0.435803

Conf 2
6 1.97234 -0.698262 0.774205
6 1.25765 0.646413 0.491974
6 0.329103 0.271652 -0.710976
8 0.864777 -0.902069 -1.29599
6 2.07919 -1.30331 -0.632793
7 -1.07915 0.031138 -0.347037

6 2.1967 -2.82134 -0.686012
6 0.577292 1.2677 1.70709
8 3.1947 -0.584228 1.4543
7 2.36327 1.51815 -0.018144
6 -1.51397 -1.18849 0.133253
6 -2.80191 -1.42894 0.461758
6 -3.8075 -0.387753 0.307679
7 -3.26872 0.822436 -0.185291
6 -1.95758 1.11362 -0.500055
8 1.03481 -3.4807 -0.19444
8 -1.58479 2.21685 -0.87141
8 -4.99727 -0.475759 0.558675
1 1.32539 -1.33033 1.39147
1 0.298394 1.08751 -1.43512
1 2.9473 -0.860615 -1.14338
1 3.03416 -3.11749 -0.046105
1 2.4254 -3.14251 -1.71064
1 0.100783 2.21945 1.45545
1 1.32612 1.43588 2.48548
1 -0.186704 0.598461 2.10941
1 3.66842 0.158493 1.04552
1 -0.742489 -1.94386 0.217496
1 -3.11695 -2.39325 0.835772
1 0.378915 -3.44684 -0.903035
7 2.08258 2.69996 -0.260529
7 1.9555 3.803 -0.51672
1 -3.91894 1.59177 -0.294081

Conf 7
6 2.01252 -0.358706 0.744494
6 0.983494 0.761817 0.455632
6 0.139579 0.114974 -0.71045
8 0.835094 -1.04238 -1.13929
6 2.17698 -1.0181 -0.62925
7 -1.23231 -0.282163 -0.349587
6 2.76243 -2.42449 -0.578872
6 0.215526 1.21535 1.69383
8 3.21199 0.063591 1.35135
7 1.82104 1.86003 -0.109329
6 -1.5627 -1.57291 0.01132
6 -2.82454 -1.94478 0.317287
6 -3.91182 -0.978679 0.258361
7 -3.47876 0.30845 -0.138298
6 -2.20017 0.726013 -0.43663
8 4.12229 -2.37207 -0.191162
8 -1.91566 1.87662 -0.739845
8 -5.08752 -1.18269 0.506569
1 1.55667 -1.08278 1.431
1 0.036547 0.836864 -1.52407
1 2.8152 -0.391384 -1.26813
1 2.72494 -2.88463 -1.57074
1 2.15623 -3.04876 0.100189
1 -0.467146 2.03655 1.46368
1 0.930881 1.54365 2.45276
1 -0.367615 0.390165 2.11057
1 3.48614 0.871669 0.885724

1 -0.731008 -2.26427 0.00405
1 -3.06045 -2.96323 0.592872
1 4.18556 -1.79774 0.588039
7 1.26709 2.95105 -0.311359
7 0.887143 3.99997 -0.538129
1 -4.19274 1.02517 -0.194256

Compound 1d: 2'MeAzide Uracil anion

Conf 11
6 -2.04877 -0.696961 -0.527969
6 -1.31245 0.662848 -0.456006
6 -0.260393 0.466243 0.693307
8 -0.674074 -0.6792 1.42367
6 -1.86541 -1.27293 0.889026
7 1.1385 0.391662 0.332634
6 -1.73219 -2.79572 0.951187
6 -0.775382 1.15763 -1.79119
8 -3.39929 -0.60806 -0.941844
7 -2.37931 1.61102 0.067885
6 2.02996 1.27513 0.913741
6 3.3541 1.16864 0.705421
6 3.87647 0.067753 -0.130884
7 2.95991 -0.816212 -0.665781
6 1.65604 -0.698913 -0.470307
8 -0.896253 -3.36102 -0.022742
8 0.777947 -1.45627 -0.961795
8 5.09434 -0.040349 -0.32672
1 -1.53324 -1.33272 -1.2442
1 -0.328484 1.34053 1.35502
1 -2.72807 -0.971603 1.50917
1 -2.73699 -3.22269 0.817651
1 -1.40755 -3.04737 1.97747
1 -0.26956 2.12428 -1.69497
1 -1.60603 1.25937 -2.49644
1 -0.06574 0.423776 -2.1746
1 -3.78101 0.13415 -0.448049
1 1.58635 2.05176 1.53283
1 4.05337 1.86623 1.15185
1 -0.150293 -2.74404 -0.216093
7 -2.07435 2.79411 0.191464
7 -1.8999 3.91587 0.346356

Conf 107
6 1.86605 0.628787 -0.404981
6 1.21599 -0.755157 -0.149631
6 0.090006 -0.435248 0.889852
8 0.405476 0.833262 1.45262
6 1.59686 1.39714 0.892183
7 -1.28667 -0.484033 0.444277
6 1.3966 2.90353 0.708514
6 0.761023 -1.47638 -1.41367
8 3.26651 0.56987 -0.665281
7 2.17029 -1.61394 0.638967
6 -2.1718 -1.31675 1.1031
6 -3.48367 -1.32006 0.805732
6 -4.00156 -0.394745 -0.222523

7 -3.0949 0.450685 -0.831912
6 -1.80273 0.440448 -0.543875
8 0.620154 3.26805 -0.403705
8 -0.935578 1.16915 -1.09816
8 -5.20678 -0.390653 -0.508029
1 1.32832 1.10999 -1.22125
1 0.167386 -1.19615 1.67482
1 2.44209 1.22877 1.57921
1 2.3885 3.36169 0.584009
1 0.978259 3.28952 1.65526
1 0.264074 -2.42053 -1.17298
1 1.62535 -1.69616 -2.05206
1 0.068734 -0.835384 -1.96099
1 3.38799 0.678921 -1.61521
1 -1.73194 -1.96099 1.86064
1 -4.17604 -1.97978 1.31606
1 -0.103411 2.60691 -0.526744
7 3.16154 -2.05198 0.057383
7 4.10143 -2.55983 -0.355642

Conf 48
6 -2.05966 0.646531 0.519489
6 -1.30995 -0.707854 0.452491
6 -0.261047 -0.479637 -0.699317
8 -0.708859 0.649496 -1.43238
6 -1.91516 1.21028 -0.896502
7 1.13718 -0.360899 -0.340092
6 -1.82901 2.736 -0.973032
6 -0.728665 -1.1634 1.7873
8 -3.43894 0.568536 0.849631
7 -2.32837 -1.70648 -0.041383
6 2.04888 -1.23653 -0.901074
6 3.36978 -1.10361 -0.68595
6 3.86673 0.017023 0.138484
7 2.93021 0.890383 0.656171
6 1.62981 0.746895 0.454067
8 -1.01407 3.33644 -0.000368
8 0.73759 1.49541 0.935218
8 5.08105 0.150732 0.341461
1 -1.51801 1.28994 1.2138
1 -0.30334 -1.35783 -1.3572
1 -2.77533 0.870202 -1.4956
1 -2.84587 3.13444 -0.846989
1 -1.50741 2.98657 -2.00012
1 -0.16562 -2.09688 1.68567
1 -1.54036 -1.33169 2.50455
1 -0.061056 -0.389744 2.16846
1 -3.49853 0.456671 1.80632
1 1.62367 -2.03018 -1.51117
1 4.08381 -1.79521 -1.11812
1 -0.240517 2.75182 0.184449
7 -1.9404 -2.86263 -0.188983
7 -1.70945 -3.97283 -0.365474

Conf 1
6 2.00281 -0.675853 0.519518

6 1.32228 0.698691 0.42776
6 0.252102 0.484508 -0.708138
8 0.676563 -0.652207 -1.44653
6 1.87329 -1.23193 -0.906362
7 -1.14444 0.38449 -0.333236
6 1.76426 -2.75666 -0.989928
6 0.784006 1.20883 1.75862
8 3.34971 -0.596393 0.975462
7 2.37236 1.63251 -0.12355
6 -2.04346 1.28495 -0.875495
6 -3.36606 1.17238 -0.659448
6 -3.88129 0.044413 0.14277
7 -2.95852 -0.852019 0.644899
6 -1.65562 -0.726905 0.446003
8 0.98322 -3.35002 0.016574
8 -0.779154 -1.49951 0.917231
8 -5.09789 -0.075708 0.341029
1 1.39549 -1.28455 1.18707
1 0.30013 1.36207 -1.36573
1 2.74197 -0.897285 -1.4957
1 2.77852 -3.17555 -0.907973
1 1.39913 -3.00044 -2.0033
1 0.285005 2.1776 1.65185
1 1.61492 1.31587 2.46284
1 0.066696 0.487438 2.15223
1 3.5075 -1.4028 1.48059
1 -1.60662 2.0814 -1.47356
1 -4.06821 1.88408 -1.07831
1 0.199818 -2.7757 0.196666
7 2.08093 2.82272 -0.192417
7 1.93711 3.95573 -0.3055

Conf 117
6 -1.86043 -0.563958 -0.659963
6 -1.14733 0.803862 -0.696386
6 -0.155077 0.726012 0.522966
8 -0.63304 -0.312759 1.36924
6 -1.76366 -1.00108 0.814161
7 1.26247 0.581312 0.255982
6 -1.60365 -2.51317 1.03144
6 -0.501902 1.1205 -2.03905
8 -3.20236 -0.46382 -1.13396
7 -2.09318 1.94752 -0.447066
6 2.13595 1.52094 0.77178
6 3.46861 1.36983 0.669625
6 4.01909 0.158389 0.028958
7 3.11754 -0.773735 -0.447491
6 1.8073 -0.608866 -0.361624
8 -0.828501 -3.18385 0.073253
8 0.949273 -1.41207 -0.818573
8 5.24417 0.002391 -0.06302
1 -1.27807 -1.25202 -1.2715
1 -0.242346 1.67892 1.05838
1 -2.67243 -0.674054 1.34323
1 -2.607 -2.96562 1.01051
1 -1.21788 -2.64598 2.05762

1 0.007423 2.08778 -2.00811
1 -1.2806 1.16273 -2.80647
1 0.215961 0.338691 -2.28591
1 -3.46189 -1.35187 -1.40772
1 1.67093 2.37926 1.25083
1 4.15232 2.11189 1.06572
1 -0.029746 -2.63495 -0.128659
7 -2.98554 1.84707 0.392697
7 -3.85649 1.91945 1.13375

Compound 1e: 2'MeCyano Uracil

Conf 179
6 2.05871 -0.260529 0.726425
6 1.25219 1.03216 0.376754
6 0.338995 0.525093 -0.798594
8 0.981683 -0.604608 -1.35129
6 2.18623 -0.94255 -0.64415
7 -1.02953 0.153987 -0.387632
6 2.35614 -2.44966 -0.600436
6 0.533165 1.65012 1.58529
8 3.2722 -0.064143 1.40328
6 2.21672 1.99932 -0.175601
6 -1.36858 -1.13882 -0.032557
6 -2.62991 -1.49094 0.301063
6 -3.7048 -0.511702 0.27738
7 -3.26409 0.771771 -0.120136
6 -1.98851 1.1748 -0.453028
8 1.32255 -3.00928 0.202134
8 -1.70521 2.32184 -0.767672
8 -4.87784 -0.700758 0.552596
1 1.44362 -0.879592 1.38457
1 0.21441 1.30477 -1.55124
1 3.05049 -0.520693 -1.18076
1 3.34968 -2.65914 -0.177871
1 2.32531 -2.84027 -1.62683
1 -0.011669 2.55164 1.30059
1 1.26995 1.90037 2.35241
1 -0.17057 0.928824 2.00745
1 3.81475 0.558033 0.893361
1 -0.551282 -1.85077 -0.054932
1 -2.87305 -2.50801 0.57584
1 -3.96979 1.49759 -0.158521
1 1.40473 -3.9705 0.186153
7 3.04423 2.70561 -0.583361

Conf 13
6 2.0779 -0.245221 0.756553
6 1.23733 1.02964 0.41697
6 0.338982 0.509279 -0.765234
8 0.990623 -0.632309 -1.29887
6 2.23084 -0.899256 -0.622159
7 -1.02966 0.14718 -0.367838
6 2.47894 -2.40354 -0.6065
6 0.488272 1.62553 1.61855
8 3.28106 -0.024417 1.44264
6 2.17767 2.02848 -0.12063

6 -1.33068 -1.07792 0.197906
6 -2.58438 -1.43208 0.552469
6 -3.69745 -0.518244 0.336136
7 -3.29406 0.70496 -0.244826
6 -2.02372 1.11401 -0.594008
8 1.37732 -3.13404 -0.081633
8 -1.77587 2.21894 -1.05057
8 -4.86965 -0.717703 0.603493
1 1.47846 -0.891282 1.405
1 0.219868 1.27987 -1.52738
1 3.06094 -0.411077 -1.15817
1 3.33799 -2.59742 0.043769
1 2.73514 -2.74821 -1.61659
1 -0.093805 2.50134 1.32341
1 1.20978 1.92016 2.38424
1 -0.184889 0.881554 2.05044
1 3.81617 0.607954 0.937524
1 -0.485007 -1.7427 0.323899
1 -2.79327 -2.3974 0.992521
1 -4.02469 1.3886 -0.404246
1 0.72057 -3.1898 -0.788243
7 2.98266 2.76651 -0.5169

Conf 15
6 2.00267 0.084824 0.973676
6 1.05812 1.16494 0.342875
6 0.270955 0.317273 -0.728902
8 1.00886 -0.875092 -0.925334
6 2.26442 -0.843654 -0.214915
7 -1.10335 -0.039876 -0.335046
6 2.65306 -2.27066 0.131446
6 0.207534 1.9105 1.38281
8 3.14473 0.572987 1.62218
6 1.92171 2.12352 -0.366921
6 -1.40495 -1.21532 0.323961
6 -2.6633 -1.55959 0.670791
6 -3.78235 -0.688834 0.337835
7 -3.38002 0.478449 -0.350652
6 -2.10612 0.871431 -0.702517
8 2.89405 -3.05478 -1.01877
8 -1.85619 1.92226 -1.27235
8 -4.95859 -0.882152 0.590054
1 1.42999 -0.448713 1.74168
1 0.171132 0.878339 -1.65935
1 3.03743 -0.411314 -0.867791
1 1.87299 -2.71193 0.775195
1 3.5834 -2.24802 0.707277
1 -0.419672 2.66724 0.908584
1 0.868985 2.39517 2.10499
1 -0.430581 1.2054 1.92097
1 3.65115 1.11458 0.996398
1 -0.553234 -1.85321 0.517288
1 -2.87312 -2.48909 1.18152
1 -4.11685 1.12496 -0.607354
1 2.08994 -3.02917 -1.55592
7 2.6684 2.84335 -0.890101

Conf 113
6 2.04146 -0.268573 0.732419
6 1.26904 1.04001 0.379958
6 0.34888 0.537176 -0.795165
8 0.99504 -0.584694 -1.35479
6 2.1924 -0.931325 -0.636789
7 -1.0196 0.156169 -0.382547
6 2.36647 -2.43774 -0.617807
6 0.537881 1.66753 1.57775
8 3.31487 -0.09634 1.30646
6 2.21613 2.01944 -0.18221
6 -1.35293 -1.14289 -0.046903
6 -2.61204 -1.50692 0.283127
6 -3.69223 -0.533526 0.273593
7 -3.25859 0.756797 -0.107882
6 -1.98503 1.17134 -0.436632
8 1.34082 -3.01895 0.182543
8 -1.71034 2.32415 -0.737172
8 -4.86394 -0.732499 0.548266
1 1.39286 -0.885156 1.36777
1 0.219578 1.32287 -1.54042
1 3.0644 -0.492234 -1.14167
1 3.36348 -2.65288 -0.207566
1 2.32851 -2.80753 -1.65129
1 -0.027657 2.54909 1.27198
1 1.26407 1.96962 2.3384
1 -0.149688 0.943023 2.02184
1 3.21152 0.140067 2.23704
1 -0.532456 -1.85076 -0.080708
1 -2.84942 -2.52934 0.542807
1 -3.9683 1.47904 -0.138789
1 1.38863 -3.9786 0.094134
7 2.95001 2.79736 -0.633004

Conf 104
6 -2.00981 0.262886 0.738772
6 -1.25851 -1.04622 0.384244
6 -0.344971 -0.550653 -0.798046
8 -0.999037 0.565719 -1.36573
6 -2.19781 0.902644 -0.643649
7 1.02076 -0.157216 -0.390526
6 -2.39682 2.40611 -0.657172
6 -0.53608 -1.68026 1.58236
8 -3.21732 0.007927 1.42066
6 -2.21733 -2.01653 -0.17828
6 1.34264 1.14378 -0.053274
6 2.59704 1.51903 0.281853
6 3.68496 0.553959 0.277615
7 3.26357 -0.739499 -0.105566
6 1.99491 -1.16486 -0.441242
8 -1.41398 3.01986 0.173541
8 1.73192 -2.31874 -0.745735
8 4.85368 0.763275 0.55735
1 -1.33512 0.885365 1.33746
1 -0.212549 -1.34078 -1.53776

1 -3.06545 0.43603 -1.13078
1 -3.41336 2.61823 -0.292019
1 -2.32772 2.75916 -1.6943
1 -0.003864 -2.585 1.28472
1 -1.27227 -1.93312 2.34902
1 0.180389 -0.972431 2.00727
1 -3.37528 0.724627 2.0465
1 0.516625 1.84453 -0.091399
1 2.82523 2.54342 0.541969
1 3.97926 -1.45595 -0.133182
1 -1.40552 3.96762 -0.007613
7 -2.95318 -2.78549 -0.640998

Conf 4
6 1.97917 -0.088065 0.733512
6 0.981586 1.05991 0.376146
6 0.141403 0.381408 -0.78053
8 0.867669 -0.759451 -1.20367
6 2.1771 -0.779934 -0.618204
7 -1.20885 -0.049485 -0.382496
6 2.69736 -2.21115 -0.517635
6 0.18732 1.57854 1.58479
8 3.16378 0.287505 1.3965
6 1.77755 2.15628 -0.199306
6 -1.48133 -1.33445 0.045441
6 -2.72131 -1.74374 0.387706
6 -3.85039 -0.828403 0.296347
7 -3.47885 0.452389 -0.173456
6 -2.22418 0.910013 -0.514659
8 4.03766 -2.21685 -0.066919
8 -1.99752 2.05117 -0.886907
8 -5.01206 -1.07066 0.572832
1 1.4654 -0.772397 1.41912
1 -0.002862 1.08502 -1.60231
1 2.87793 -0.197672 -1.23576
1 2.68649 -2.68088 -1.50517
1 2.03109 -2.79837 0.137285
1 -0.485853 2.38635 1.29348
1 0.884098 1.94893 2.34071
1 -0.398269 0.768134 2.02576
1 3.62307 0.95363 0.859651
1 -0.624251 -1.9938 0.056532
1 -2.91009 -2.75693 0.714314
1 -4.22469 1.13279 -0.260197
1 4.07992 -1.69376 0.748148
7 2.47858 2.98036 -0.622311

Conf 38
6 -2.16003 0.168221 0.556885
6 -1.26471 -1.09183 0.412104
6 -0.286295 -0.67004 -0.7565
8 -0.826452 0.469872 -1.36969
6 -2.14456 0.769035 -0.854845
7 1.12349 -0.421181 -0.390172

6 -2.35674 2.27865 -0.918895
6 -0.603386 -1.5184 1.73324
8 -3.45211 -0.173922 1.01295
6 -2.06874 -2.21805 -0.102272
6 2.1039 -1.25752 -0.902788
6 3.42451 -1.07638 -0.701987
6 3.89403 0.064275 0.07008
7 2.83135 0.874725 0.533082
6 1.47604 0.701218 0.366144
8 -1.68155 2.99402 0.093918
8 0.657973 1.46621 0.864853
8 5.04838 0.354579 0.325438
1 -1.6601 0.858578 1.23882
1 -0.227889 -1.49173 -1.4781
1 -2.90051 0.262586 -1.47218
1 -3.42625 2.48243 -0.792303
1 -2.07698 2.61913 -1.92805
1 0.04488 -2.38837 1.59959
1 -1.38606 -1.78257 2.44861
1 -0.022003 -0.692199 2.14525
1 -3.79325 0.567654 1.52772
1 1.72701 -2.08863 -1.48793
1 4.15668 -1.75549 -1.11599
1 3.09315 1.68544 1.08226
1 -0.76212 2.68106 0.127271
7 -2.64351 -3.13055 -0.531731

Conf 20
6 2.67805 0.325767 0.042684
6 1.28084 0.858489 0.53094
6 0.377503 0.429258 -0.686012
8 0.925685 -0.738411 -1.21671
6 2.33503 -0.827815 -0.92809
7 -1.05629 0.236415 -0.417573
6 2.61331 -2.24589 -0.435949
6 0.897063 0.281857 1.90884
8 3.40076 1.27625 -0.71896
6 1.29705 2.32324 0.62365
6 -1.92597 1.24854 -0.803251
6 -3.26293 1.19304 -0.647659
6 -3.87785 0.010414 -0.064441
7 -2.92595 -0.971611 0.286836
6 -1.55107 -0.941631 0.168992
8 1.91005 -2.57427 0.740406
8 -0.862681 -1.87269 0.56094
8 -5.06515 -0.182126 0.126543
1 3.25635 -0.026297 0.905607
1 0.418809 1.24009 -1.4257
1 2.89865 -0.642637 -1.85118
1 3.68374 -2.3438 -0.221641
1 2.37767 -2.93911 -1.25791
1 -0.131238 0.539373 2.17176
1 1.55475 0.712163 2.66908
1 1.0198 -0.799033 1.91218
1 3.63398 2.02479 -0.15128
1 -1.44161 2.11299 -1.24251

1 -3.90072 2.00829 -0.959348
1 -3.29227 -1.81963 0.703716
1 0.957342 -2.49744 0.546651
7 1.315 3.48214 0.702834

Conf 10
6 2.61943 0.452115 0.072142
6 1.18032 0.752155 0.634551
6 0.316469 0.203248 -0.553254
8 0.974934 -0.996528 -0.915317
6 2.38479 -0.710909 -0.928032
7 -1.08411 -0.087113 -0.286658
6 3.14043 -2.00199 -0.644763
6 0.95373 0.009036 1.96596
8 3.17353 1.51069 -0.679617
6 1.00969 2.19213 0.840771
6 -1.48486 -1.28197 0.282734
6 -2.77752 -1.58927 0.515271
6 -3.83131 -0.650933 0.153401
7 -3.33326 0.525853 -0.447079
6 -2.02449 0.872117 -0.715335
8 2.79956 -2.58221 0.605913
8 -1.70256 1.9109 -1.26316
8 -5.02959 -0.803632 0.314978
1 3.27715 0.153595 0.898366
1 0.328995 0.936011 -1.36539
1 2.67192 -0.330943 -1.91803
1 4.21193 -1.78136 -0.613597
1 2.96909 -2.70635 -1.47041
1 -0.085678 0.078818 2.29343
1 1.58277 0.450026 2.7434
1 1.24275 -1.0386 1.86311
1 3.2928 2.28003 -0.103817
1 -0.676406 -1.96267 0.508558
1 -3.06284 -2.53353 0.95763
1 -4.02369 1.2093 -0.734867
1 1.91052 -2.94733 0.509083
7 0.947883 3.33337 1.04653

Conf 1
6 2.6267 0.389669 0.057714
6 1.1916 0.749136 0.590424
6 0.319439 0.257803 -0.619019
8 0.948654 -0.931873 -1.04281
6 2.36877 -0.777436 -0.929805
7 -1.07695 -0.053357 -0.333804
6 2.98346 -2.11613 -0.563951
6 0.885334 0.016538 1.91206
8 3.22284 1.42069 -0.70334
6 1.09848 2.19629 0.804135
6 -1.45477 -1.29702 0.140031
6 -2.73992 -1.6224 0.392757
6 -3.80357 -0.656518 0.158305
7 -3.32555 0.571421 -0.350026
6 -2.02537 0.94234 -0.629032
8 2.56401 -2.48999 0.741744

8 -1.71839 2.03181 -1.08087
8 -4.99636 -0.822461 0.350366
1 3.26357 0.085198 0.897514
1 0.31555 1.02733 -1.39639
1 2.78042 -0.463234 -1.8981
1 4.07758 -2.01398 -0.625672
1 2.66869 -2.85578 -1.3138
1 -0.142595 0.198075 2.23262
1 1.55337 0.382515 2.69621
1 1.05482 -1.05501 1.79667
1 3.32114 2.20729 -0.146691
1 -0.634757 -1.99061 0.268835
1 -3.01124 -2.60295 0.758367
1 -4.0239 1.27915 -0.544197
1 2.94578 -3.35124 0.951115
7 1.11174 3.33825 1.01541

Conf 174
6 1.93112 -0.124038 0.741623
6 1.00913 1.07379 0.404768
6 0.153127 0.463864 -0.778147
8 0.892252 -0.634133 -1.29247
6 2.16971 -0.724955 -0.645684
7 -1.18719 -0.011763 -0.38842
6 2.64781 -2.17164 -0.619482
6 0.214221 1.59995 1.61
8 3.08662 0.269975 1.42793
6 1.82425 2.1661 -0.160167
6 -1.42713 -1.31343 0.002313
6 -2.65265 -1.76554 0.343632
6 -3.80433 -0.876243 0.291337
7 -3.46855 0.425143 -0.144846
6 -2.22884 0.924637 -0.486742
8 3.87181 -2.25147 0.117387
8 -2.04032 2.07989 -0.833547
8 -4.95706 -1.15554 0.57202
1 1.33485 -0.839415 1.33526
1 -0.01646 1.21689 -1.54846
1 2.9069 -0.103661 -1.17711
1 2.75375 -2.57183 -1.63509
1 1.92544 -2.79838 -0.086597
1 -0.440677 2.42224 1.31664
1 0.912688 1.95378 2.3716
1 -0.390866 0.798741 2.04205
1 3.66554 -0.509661 1.44258
1 -0.556135 -1.9537 -0.013282
1 -2.81229 -2.79259 0.641114
1 -4.23134 1.08929 -0.205635
1 4.5927 -1.97161 -0.462837
7 2.44302 3.02594 -0.634087

Conf 112
6 -1.9423 -0.123616 -0.729331
6 -1.01999 1.07758 -0.400677
6 -0.159711 0.479906 0.782941
8 -0.897352 -0.610188 1.31223

6 -2.17154 -0.721463 0.661556
7 1.17898 -0.001887 0.390439
6 -2.60916 -2.17801 0.650656
6 -0.230722 1.59708 -1.61272
8 -3.09947 0.275183 -1.40853
6 -1.83366 2.17378 0.158882
6 1.414 -1.3068 0.009658
6 2.63648 -1.76515 -0.334834
6 3.79044 -0.87828 -0.29687
7 3.46009 0.427347 0.129628
6 2.22321 0.932847 0.474474
8 -3.81451 -2.20276 -0.113023
8 2.04017 2.09151 0.812274
8 4.94097 -1.16303 -0.581849
1 -1.3495 -0.838973 -1.32715
1 0.016306 1.24013 1.54466
1 -2.92498 -0.119851 1.1879
1 -2.76684 -2.53785 1.6759
1 -1.82589 -2.79699 0.186877
1 0.422653 2.4237 -1.3283
1 -0.933377 1.94294 -2.37415
1 0.375274 0.794514 -2.0411
1 -3.71329 -0.475464 -1.36582
1 0.541185 -1.94433 0.034891
1 2.79211 -2.79506 -0.624492
1 4.22454 1.09046 0.18011
1 -4.15743 -3.10413 -0.137726
7 -2.44499 3.0414 0.628113

Conf 103
6 -2.02176 0.079919 0.599116
6 -1.10392 -1.15613 0.417263
6 -0.13634 -0.684389 -0.742095
8 -0.740029 0.418401 -1.37346
6 -2.02914 0.695198 -0.80717
7 1.25311 -0.355741 -0.37067
6 -2.2587 2.19828 -0.794357
6 -0.425714 -1.61593 1.71889
8 -3.29787 -0.291225 1.06177
6 -1.88773 -2.27977 -0.135108
6 2.28448 -1.0853 -0.9416
6 3.5914 -0.809094 -0.75952
6 3.98814 0.329582 0.055176
7 2.87906 1.03011 0.577999
6 1.53314 0.761318 0.431413
8 -3.52047 2.37842 -0.142337
8 0.671777 1.4361 0.974359
8 5.12369 0.698054 0.298356
1 -1.52009 0.763464 1.29144
1 -0.028068 -1.50396 -1.45914
1 -2.81242 0.202529 -1.4007
1 -2.27455 2.59661 -1.81803
1 -1.43893 2.67392 -0.241629
1 0.245328 -2.46164 1.54659
1 -1.19624 -1.92833 2.42762
1 0.135319 -0.790604 2.15917

1 -3.83408 0.516017 1.01417
1 1.9615 -1.91686 -1.55769
1 4.36435 -1.40911 -1.21901
1 3.0907 1.83202 1.16014
1 -3.64727 3.31866 0.031571
7 -2.43865 -3.19049 -0.598193

Compound 1e: 2'MeCyano Uracil anion

Conf 12
6 2.14923 -0.247483 0.532348
6 1.28299 1.0507 0.414393
6 0.256285 0.705356 -0.739706
8 0.787543 -0.422521 -1.42159
6 2.02077 -0.885726 -0.860985
7 -1.12475 0.503873 -0.369353
6 2.03618 -2.41704 -0.870117
6 0.6629 1.49286 1.74873
8 3.48593 -0.045162 0.951561
6 2.16487 2.11817 -0.092468
6 -2.09711 1.29338 -0.957951
6 -3.40444 1.06941 -0.738779
6 -3.81968 -0.061689 0.117286
7 -2.82311 -0.848508 0.660821
6 -1.53682 -0.613881 0.458868
8 1.27116 -3.02784 0.132242
8 -0.591475 -1.27525 0.963947
8 -5.02115 -0.278763 0.321273
1 1.68547 -0.891914 1.2752
1 0.239307 1.55052 -1.43891
1 2.85619 -0.530134 -1.49307
1 3.07921 -2.73849 -0.73565
1 1.72896 -2.73138 -1.88424
1 0.04132 2.38404 1.62541
1 1.46023 1.71743 2.46343
1 0.052873 0.674266 2.13102
1 3.91001 0.555705 0.320286
1 -1.72935 2.09609 -1.59294
1 -4.16719 1.69214 -1.1918
1 0.458268 -2.492 0.298455
7 2.89609 2.92831 -0.495236

Conf 109
6 2.14541 0.24138 -0.517167
6 1.28871 -1.0575 -0.402165
6 0.259834 -0.704255 0.750841
8 0.797524 0.41726 1.43375
6 2.03676 0.869659 0.874286
7 -1.12021 -0.489144 0.375493
6 2.06571 2.40059 0.88717
6 0.648806 -1.48579 -1.73411
8 3.51727 0.027404 -0.825018
6 2.13749 -2.15953 0.090474
6 -2.09957 -1.27565 0.955492
6 -3.40513 -1.04668 0.728958
6 -3.81094 0.08613 -0.128273
7 -2.80751 0.870097 -0.663867

6 -1.52337 0.630708 -0.45313
8 1.31918 3.0207 -0.126539
8 -0.574212 1.29145 -0.953805
8 -5.01004 0.308006 -0.34166
1 1.6662 0.893437 -1.24723
1 0.233727 -1.5519 1.44608
1 2.87234 0.495485 1.48876
1 3.11223 2.7147 0.767163
1 1.7463 2.71626 1.89657
1 0.002947 -2.35814 -1.60353
1 1.43098 -1.74913 -2.4548
1 0.059746 -0.653905 -2.1212
1 3.59442 -0.019524 -1.78551
1 -1.73903 -2.08087 1.59123
1 -4.17237 -1.66774 1.1767
1 0.493923 2.50244 -0.28605
7 2.76179 -3.06011 0.477482

Conf 100
6 -2.11538 0.239779 0.527575
6 -1.29276 -1.06595 0.40611
6 -0.256086 -0.720557 -0.744068
8 -0.79411 0.392085 -1.44274
6 -2.0327 0.845899 -0.88111
7 1.12138 -0.494807 -0.3631
6 -2.0696 2.37674 -0.920268
6 -0.665329 -1.51119 1.73705
8 -3.45314 -0.011474 0.948588
6 -2.15587 -2.15118 -0.102412
6 2.1029 -1.29937 -0.914234
6 3.40797 -1.06465 -0.691104
6 3.81212 0.096456 0.127773
7 2.80728 0.894301 0.639189
6 1.52316 0.647095 0.436756
8 -1.35573 3.01371 0.107897
8 0.57537 1.32098 0.921642
8 5.011 0.328986 0.331368
1 -1.59302 0.887782 1.22907
1 -0.222302 -1.5757 -1.42944
1 -2.87093 0.452357 -1.47957
1 -3.12037 2.69213 -0.834362
1 -1.72597 2.68228 -1.92423
1 -0.050326 -2.40601 1.60838
1 -1.45986 -1.73541 2.45502
1 -0.046373 -0.699328 2.1202
1 -3.73157 0.760727 1.45501
1 1.74415 -2.12397 -1.52565
1 4.1758 -1.70001 -1.1173
1 -0.516228 2.51783 0.270056
7 -2.78589 -3.03742 -0.512327

Compound 13: alpha thymidine

Conf 11
6 -2.41275 -0.006989 -0.690058
6 -2.63908 -0.70809 0.675702
6 -1.5018 -0.160152 1.58053
6 -0.659783 0.764492 0.684452
8 -1.41646 1.02332 -0.473443
8 -2.68249 -2.11175 0.569253
6 -3.64456 0.688741 -1.25385
8 -4.1339 1.68501 -0.370734
1 -0.46931 1.72689 1.16636
7 0.708334 0.26989 0.36077
6 0.902682 -1.01737 -0.12648
7 2.21314 -1.32162 -0.427787
6 3.34766 -0.491613 -0.354063
6 3.05254 0.858369 0.124175
6 1.77329 1.15756 0.441431
8 4.44708 -0.913391 -0.675306
8 0.002615 -1.83862 -0.278649
6 4.19056 1.83081 0.229893
1 4.66979 1.97371 -0.744002
1 3.85152 2.80235 0.598391
1 4.96379 1.45007 0.904974
1 -0.925405 -0.977434 2.0143
1 -1.9115 0.43233 2.4031
1 -4.44179 -0.050196 -1.38239
1 -3.41086 1.10964 -2.2438
1 -2.02854 -0.740269 -1.4081
1 -3.60876 -0.406241 1.0787
1 1.50284 2.14649 0.795577
1 2.36676 -2.26145 -0.774021
1 -1.80811 -2.37814 0.240437
1 -3.38806 2.28328 -0.219749

Conf 103
6 2.47723 0.784109 0.129569
6 2.74658 -0.528061 0.871994
6 1.33713 -1.12757 0.971233
6 0.692112 -0.685904 -0.349307
8 1.38159 0.488388 -0.766244
8 3.36166 -0.226735 2.11232
6 3.63684 1.30192 -0.711617
8 4.01682 0.373849 -1.71178
1 0.7781 -1.45773 -1.11748
7 -0.753551 -0.395364 -0.245995
6 -1.60871 -1.4873 -0.380167
7 -2.94645 -1.16305 -0.256134
6 -3.51795 0.093557 0.018503
6 -2.53978 1.17241 0.158717
6 -1.2264 0.875446 0.019557
8 -4.73029 0.213729 0.117883
8 -1.20666 -2.62486 -0.581899
6 -3.0562 2.55446 0.435505
1 -3.62952 2.57677 1.36816
1 -2.23809 3.27552 0.511369

1 -3.73811 2.88142 -0.35635
1 0.822055 -0.664599 1.81972
1 1.30488 -2.21228 1.09205
1 4.50201 1.4507 -0.058084
1 3.36778 2.27671 -1.1456
1 2.17573 1.54742 0.862687
1 3.39451 -1.1536 0.243274
1 -0.45374 1.63142 0.079919
1 -3.59125 -1.93844 -0.350458
1 3.7531 -1.03637 2.46267
1 3.22245 0.218593 -2.24318

Conf 179
6 -2.73033 -0.042225 -0.806855
6 -2.74801 -0.861903 0.507078
6 -1.62878 -0.191486 1.33667
6 -0.774811 0.583717 0.30605
8 -1.3785 0.423036 -0.962465
8 -2.54538 -2.23815 0.286727
6 -3.70211 1.12943 -0.841099
8 -3.48833 1.9378 0.314728
1 -0.747445 1.64623 0.568529
7 0.64685 0.194575 0.206888
6 0.996902 -1.12533 -0.06111
7 2.35341 -1.33318 -0.209219
6 3.39844 -0.393367 -0.158731
6 2.94301 0.973045 0.091259
6 1.61735 1.1856 0.24677
8 4.5575 -0.743342 -0.319584
8 0.198459 -2.05179 -0.149112
6 3.97714 2.05887 0.151749
1 4.54161 2.10943 -0.784972
1 3.51988 3.03421 0.337342
1 4.70555 1.85722 0.94392
1 -1.05808 -0.939805 1.88757
1 -2.05464 0.521428 2.04425
1 -4.72992 0.733902 -0.869816
1 -3.53005 1.70219 -1.76373
1 -2.94685 -0.701801 -1.6529
1 -3.71704 -0.768335 1.00573
1 1.22629 2.1814 0.424425
1 2.62066 -2.29253 -0.395523
1 -1.61304 -2.34336 0.024094
1 -4.06148 2.71158 0.254344

Conf 14
6 -2.51946 0.161484 -0.358774
6 -2.63887 -0.88222 0.781109
6 -1.43538 -0.566248 1.71244
6 -0.636722 0.548609 1.01485
8 -1.4554 1.0769 0.003314
8 -2.68765 -2.20957 0.317027
6 -3.76521 1.00703 -0.576512
8 -3.57972 1.98326 -1.58432
1 -0.402092 1.36341 1.70512
7 0.70149 0.14716 0.49656

6 0.834546 -0.959261 -0.333493
7 2.11555 -1.17683 -0.793558
6 3.27225 -0.40609 -0.569482
6 3.0399 0.762463 0.278587
6 1.78995 0.971874 0.747873
8 4.33951 -0.730425 -1.06489
8 -0.09316 -1.70294 -0.640136
6 4.20538 1.66124 0.571675
1 4.62668 2.06389 -0.355118
1 3.91316 2.49581 1.21413
1 5.0095 1.10467 1.06377
1 -0.84773 -1.46676 1.89213
1 -1.77914 -0.194685 2.68168
1 -4.0578 1.4658 0.383757
1 -4.58625 0.359606 -0.901636
1 -2.24423 -0.344334 -1.28996
1 -3.57656 -0.733154 1.32767
1 1.56509 1.82756 1.37549
1 2.22513 -1.98644 -1.39302
1 -1.84603 -2.35655 -0.146128
1 -2.78582 2.47782 -1.33651

Conf 9
6 2.45485 0.777159 0.117437
6 2.73834 -0.53412 0.870605
6 1.33593 -1.13136 0.976808
6 0.687192 -0.711664 -0.349141
8 1.38547 0.447789 -0.797908
8 3.3009 -0.332587 2.15502
6 3.60714 1.32982 -0.710637
8 4.06353 0.395966 -1.6723
1 0.762502 -1.49951 -1.10149
7 -0.755501 -0.408227 -0.245132
6 -1.62291 -1.49071 -0.381838
7 -2.95704 -1.15189 -0.255222
6 -3.51475 0.110298 0.02054
6 -2.52499 1.17888 0.159783
6 -1.21514 0.867396 0.020355
8 -4.72558 0.243917 0.121069
8 -1.23459 -2.63185 -0.588257
6 -3.02663 2.56645 0.435783
1 -3.59875 2.59562 1.36898
1 -2.201 3.27908 0.510139
1 -3.7059 2.89969 -0.355729
1 0.821032 -0.661445 1.82189
1 1.31573 -2.21286 1.11821
1 4.45087 1.55012 -0.046922
1 3.30031 2.27727 -1.17862
1 2.11989 1.53295 0.844431
1 3.38232 -1.16316 0.242296
1 -0.435396 1.61617 0.079979
1 -3.61006 -1.92037 -0.349501
1 4.26159 -0.310196 2.06617
1 3.29262 0.178563 -2.21653

Conf 105
6 -2.46975 -0.717949 -0.334649
6 -2.75237 -0.067063 1.03191
6 -1.34723 0.337623 1.47272
6 -0.685317 0.753256 0.153132
8 -1.36599 0.031729 -0.882369
8 -3.3375 -0.954596 1.96865
6 -3.61042 -0.654046 -1.34294
8 -4.00297 0.68041 -1.60756
1 -0.773275 1.8255 -0.028018
7 0.752735 0.476985 0.075071
6 1.62954 1.56607 0.074258
7 2.96035 1.20081 -0.017692
6 3.5122 -0.088894 -0.110947
6 2.51647 -1.16253 -0.119697
6 1.21046 -0.824882 -0.031737
8 4.72239 -0.242581 -0.180106
8 1.26402 2.72844 0.157398
6 3.00974 -2.57469 -0.240441
1 3.68885 -2.82053 0.582413
1 2.18045 -3.28682 -0.234202
1 3.58 -2.70988 -1.16534
1 -0.851749 -0.540447 1.90195
1 -1.32625 1.1385 2.21449
1 -4.484 -1.17458 -0.933847
1 -3.31454 -1.17995 -2.26299
1 -2.1782 -1.76688 -0.167911
1 -3.37867 0.820497 0.87206
1 0.427218 -1.57308 -0.061624
1 3.61882 1.97042 -0.013282
1 -4.29561 -0.927281 1.85535
1 -3.20415 1.13136 -1.91851

Conf 169
6 -2.44264 0.466505 0.348983
6 -2.69725 -0.516757 -0.807645
6 -1.26766 -0.879589 -1.20202
6 -0.550717 -0.909437 0.155918
8 -1.26308 -0.006721 1.0105
8 -3.41413 0.04658 -1.8814
6 -3.5849 0.544793 1.34973
8 -4.73512 0.924814 0.590494
1 -0.548345 -1.90896 0.593913
7 0.862463 -0.514852 0.108276
6 1.82405 -1.51888 0.247462
7 3.12355 -1.04767 0.193187
6 3.57099 0.272894 0.01343
6 2.49185 1.25347 -0.116392
6 1.21485 0.812341 -0.059135
8 4.76676 0.523627 -0.020156
8 1.55112 -2.70065 0.392917
6 2.87224 2.69459 -0.291542
1 3.485 2.82847 -1.18903
1 1.98768 3.33155 -0.374157
1 3.47657 3.04303 0.552422

1 -0.866644 -0.083517 -1.83871
1 -1.17601 -1.82777 -1.73531
1 -3.36021 1.27566 2.13855
1 -3.71461 -0.440067 1.82145
1 -2.27125 1.46422 -0.084087
1 -3.21098 -1.40769 -0.407743
1 0.373489 1.49231 -0.118615
1 3.84206 -1.75532 0.28651
1 -4.24337 0.37374 -1.49916
1 -5.50469 0.931242 1.17211

Conf 174
6 2.48134 0.780705 0.171598
6 2.75304 -0.577368 0.842061
6 1.34299 -1.17876 0.911659
6 0.695952 -0.662942 -0.380389
8 1.38181 0.535206 -0.733175
8 3.45399 -0.486423 2.06364
6 3.6398 1.34208 -0.644096
8 4.00313 0.474311 -1.70098
1 0.786133 -1.3919 -1.18907
7 -0.750562 -0.379758 -0.260234
6 -1.59933 -1.47532 -0.4085
7 -2.93857 -1.16305 -0.270633
6 -3.51712 0.085105 0.026509
6 -2.54574 1.16934 0.174882
6 -1.23105 0.884467 0.023123
8 -4.72931 0.195584 0.136402
8 -1.18892 -2.60536 -0.632218
6 -3.071 2.54376 0.472388
1 -3.63957 2.54961 1.40822
1 -2.2581 3.27027 0.55289
1 -3.75978 2.87574 -0.311331
1 0.810885 -0.76858 1.7802
1 1.31655 -2.26706 0.977514
1 4.51095 1.43997 0.011492
1 3.37822 2.34503 -1.01441
1 2.17874 1.51477 0.938052
1 3.39174 -1.16558 0.17621
1 -0.463623 1.64549 0.086058
1 -3.57838 -1.94145 -0.374353
1 2.88593 -0.03947 2.70717
1 3.20528 0.359824 -2.23732

Conf 1
6 -2.51086 -0.937746 -0.302498
6 -2.22894 -0.564078 1.15881
6 -1.60661 0.830013 1.02685
6 -0.894698 0.778351 -0.33749
8 -1.51067 -0.249437 -1.08071
8 -1.30726 -1.54019 1.64662
6 -3.90425 -0.585767 -0.805139
8 -4.20337 0.762946 -0.471073
1 -0.972506 1.72722 -0.86953
7 0.566394 0.504665 -0.233972
6 1.37203 1.61431 0.00106

7 2.72199 1.32184 0.065694
6 3.34741 0.066211 -0.047622
6 2.41783 -1.03746 -0.274237
6 1.09307 -0.764915 -0.355062
8 4.56364 -0.029252 0.04662
8 0.922478 2.74406 0.145144
6 2.99044 -2.41869 -0.411362
1 3.55078 -2.70077 0.486348
1 2.20259 -3.15809 -0.57704
1 3.69725 -2.46758 -1.24647
1 -0.924266 1.08231 1.84207
1 -2.39818 1.57978 0.986319
1 -4.62475 -1.28354 -0.347923
1 -3.92017 -0.746087 -1.89217
1 -2.36024 -2.01461 -0.439517
1 -3.14679 -0.554337 1.76068
1 0.355537 -1.53638 -0.525863
1 3.33012 2.11424 0.23232
1 -0.982608 -1.24329 2.50702
1 -4.99664 1.02921 -0.951005

Conf 180
6 2.5673 0.426296 0.136288
6 2.75335 -1.01659 0.615228
6 1.30385 -1.52434 0.639881
6 0.666784 -0.793851 -0.55174
8 1.43994 0.377708 -0.768114
8 3.40915 -0.999806 1.86937
6 3.73794 1.03408 -0.618216
8 3.45468 2.34745 -1.06354
1 0.670168 -1.41194 -1.45271
7 -0.748438 -0.420927 -0.335493
6 -1.68644 -1.41781 -0.593357
7 -2.99147 -1.02508 -0.365825
6 -3.45809 0.212515 0.116439
6 -2.39775 1.1869 0.374364
6 -1.11506 0.82477 0.136854
8 -4.65429 0.399762 0.283734
8 -1.37544 -2.53675 -0.978756
6 -2.80038 2.54302 0.876566
1 -3.34369 2.46195 1.82375
1 -1.92853 3.18477 1.02761
1 -3.47815 3.03415 0.170592
1 0.84115 -1.19629 1.57656
1 1.194 -2.60724 0.554543
1 4.00768 0.369468 -1.4553
1 4.59802 1.09929 0.055288
1 2.32115 1.05881 1.00113
1 3.3406 -1.57562 -0.130764
1 -0.285618 1.50604 0.278605
1 -3.69523 -1.73068 -0.546637
1 3.63983 -1.90714 2.1057
1 2.67658 2.27967 -1.63454

Conf 102
6 -2.63747 0.173097 -0.610921
6 -2.63758 -0.69631 0.664656
6 -1.46555 -0.094685 1.48062
6 -0.62101 0.707906 0.463719
8 -1.30021 0.683497 -0.774934
8 -2.50278 -2.07301 0.405031
6 -3.61749 1.35247 -0.55536
8 -4.96563 0.928943 -0.428964
1 -0.507509 1.74522 0.800734
7 0.772504 0.25736 0.264122
6 1.03599 -1.04996 -0.133006
7 2.37019 -1.31544 -0.362117
6 3.46856 -0.4404 -0.278942
6 3.10246 0.921144 0.109613
6 1.79921 1.18922 0.344806
8 4.59768 -0.835327 -0.522897
8 0.181077 -1.91881 -0.26364
6 4.19829 1.94095 0.215486
1 4.72506 2.04385 -0.738647
1 3.80554 2.91893 0.505392
1 4.94602 1.63117 0.952752
1 -0.898934 -0.885344 1.97319
1 -1.83224 0.585298 2.25467
1 -3.46021 1.99063 -1.4372
1 -3.41218 1.96605 0.329661
1 -2.85621 -0.454784 -1.48242
1 -3.59171 -0.57879 1.18479
1 1.47476 2.18512 0.626184
1 2.57465 -2.26734 -0.642795
1 -1.60125 -2.20558 0.061939
1 -5.19823 0.429125 -1.2231

Conf 109
6 -2.59878 0.586446 0.770817
6 -2.41307 1.18207 -0.640703
6 -1.57831 0.112975 -1.34867
6 -0.872539 -0.664274 -0.209522
8 -1.41827 -0.174711 1.0184
8 -1.6378 2.38358 -0.608926
6 -3.8556 -0.263232 0.939311
8 -3.92358 -1.21427 -0.115815
1 -1.03672 -1.73772 -0.292154
7 0.581678 -0.503782 -0.160444
6 1.37061 -1.65883 -0.161528
7 2.72413 -1.4005 -0.032855
6 3.37163 -0.162492 0.119444
6 2.46329 0.983355 0.131637
6 1.13628 0.756096 0.001434
8 4.58884 -0.109359 0.227154
8 0.92535 -2.79035 -0.277302
6 3.06237 2.35016 0.29467
1 3.78028 2.55911 -0.505435
1 2.29043 3.12391 0.282021
1 3.61688 2.42372 1.23623

1 -0.87413 0.569926 -2.04645
1 -2.23172 -0.567047 -1.89479
1 -4.73728 0.39991 0.937484
1 -3.80085 -0.75243 1.92108
1 -2.63494 1.38207 1.52733
1 -3.37323 1.35864 -1.14153
1 0.410133 1.56233 0.006879
1 3.31959 -2.21952 -0.039065
1 -2.12567 3.04971 -0.106659
1 -4.53495 -1.91543 0.138738

Conf 10
6 2.57348 -0.248053 0.617885
6 2.38353 -0.836163 -0.790366
6 1.46291 0.192666 -1.45359
6 0.717612 0.883335 -0.284969
8 1.37198 0.464943 0.91555
8 1.69242 -2.08659 -0.762878
6 3.78067 0.684802 0.725182
8 4.94111 -0.117076 0.544685
1 0.747652 1.97008 -0.368059
7 -0.708479 0.56966 -0.182081
6 -1.60952 1.64047 -0.159344
7 -2.92378 1.25062 0.020003
6 -3.43941 -0.045392 0.197322
6 -2.42167 -1.09531 0.178111
6 -1.12904 -0.739266 0.000635
8 -4.64046 -0.217755 0.348025
8 -1.2821 2.80965 -0.296776
6 -2.87632 -2.51424 0.361544
1 -3.59693 -2.79545 -0.413586
1 -2.03245 -3.20766 0.322117
1 -3.3883 -2.63883 1.32155
1 0.785854 -0.284866 -2.16387
1 2.05156 0.934573 -1.99898
1 3.75689 1.16852 1.71135
1 3.71017 1.47841 -0.036171
1 2.68741 -1.04251 1.36505
1 3.34408 -0.940895 -1.30703
1 -0.3281 -1.47069 -0.023754
1 -3.59784 2.00624 0.032977
1 2.24941 -2.72557 -0.298152
1 5.71928 0.444138 0.647806

Conf 153
6 2.26419 -0.428373 -0.477984
6 2.61615 0.223187 0.88324
6 1.40538 -0.124119 1.7906
6 0.490212 -0.990137 0.905485
8 1.2675 -1.42191 -0.186244
8 2.92549 1.59622 0.763381
6 3.46247 -1.06229 -1.17089
8 4.36635 -0.054498 -1.59593
1 0.152526 -1.88852 1.42615
7 -0.780258 -0.336146 0.474298

6 -0.819912 0.992004 0.060028
7 -2.06788 1.42751 -0.332613
6 -3.26531 0.694526 -0.436124
6 -3.12537 -0.707484 -0.045747
6 -1.91398 -1.13407 0.373741
8 -4.28988 1.23193 -0.824613
8 0.151209 1.74457 0.043629
6 -4.33757 -1.5875 -0.135016
1 -4.72188 -1.61409 -1.15972
1 -4.11152 -2.60938 0.17991
1 -5.14635 -1.19781 0.491573
1 0.923722 0.778132 2.16442
1 1.72127 -0.71927 2.6513
1 3.14283 -1.60845 -2.06402
1 3.93924 -1.7871 -0.490003
1 1.8462 0.340177 -1.13954
1 3.52156 -0.240493 1.29044
1 -1.75785 -2.16559 0.670074
1 -2.11042 2.39756 -0.622434
1 2.11384 2.01959 0.435673
1 4.40087 0.630704 -0.911404

Conf 158
6 -2.64976 0.163495 -0.61946
6 -2.64561 -0.726785 0.64205
6 -1.4758 -0.130983 1.46688
6 -0.632173 0.680652 0.455726
8 -1.30336 0.648587 -0.787084
8 -2.49424 -2.09606 0.358802
6 -3.59367 1.36358 -0.530895
8 -4.91989 0.861498 -0.479561
1 -0.531165 1.71865 0.795778
7 0.766732 0.2457 0.262347
6 1.04782 -1.05976 -0.130657
7 2.38636 -1.30843 -0.355823
6 3.47337 -0.420155 -0.270119
6 3.08915 0.937378 0.114706
6 1.7818 1.18975 0.344591
8 4.60845 -0.801234 -0.50911
8 0.205424 -1.9403 -0.260075
6 4.17203 1.97068 0.223035
1 4.7002 2.07972 -0.729662
1 3.76646 2.9439 0.511413
1 4.92151 1.67049 0.962505
1 -0.908689 -0.92625 1.95124
1 -1.84255 0.541735 2.24728
1 -3.42463 2.00399 -1.40877
1 -3.35084 1.96192 0.364762
1 -2.90396 -0.434235 -1.4986
1 -3.5998 -0.627735 1.1656
1 1.44437 2.18221 0.622806
1 2.60322 -2.25833 -0.633962
1 -1.59089 -2.21393 0.01562
1 -5.52894 1.60985 -0.470576

Compound 13: alpha thymidine anion

Conf 144
6 -2.49799 0.138827 -0.366601
6 -2.56931 -0.948458 0.740915
6 -1.38976 -0.584702 1.68346
6 -0.593442 0.53731 0.988863
8 -1.48663 1.08823 0.013257
8 -2.52433 -2.25863 0.235974
6 -3.77916 0.942839 -0.540127
8 -3.60521 2.02777 -1.44109
1 -0.389462 1.35435 1.6893
7 0.709394 0.173509 0.459377
6 0.907514 -0.978898 -0.386403
7 2.12839 -1.2688 -0.827022
6 3.21868 -0.477922 -0.561039
6 3.01537 0.763135 0.229163
6 1.77851 1.0198 0.696532
8 4.36221 -0.758278 -0.958994
8 -0.118476 -1.65199 -0.653879
6 4.19637 1.65267 0.481333
1 4.6336 1.99704 -0.463432
1 3.92778 2.52957 1.08335
1 4.99399 1.10267 0.994355
1 -0.780045 -1.46644 1.87841
1 -1.7639 -0.206256 2.64169
1 -4.11091 1.3035 0.450676
1 -4.57081 0.300759 -0.943698
1 -2.20939 -0.337948 -1.30879
1 -3.52096 -0.865874 1.28378
1 1.55182 1.90186 1.29306
1 -1.62913 -2.30524 -0.173858
1 -2.73828 2.39467 -1.21007

Conf 15
6 -2.21671 0.469222 -0.473818
6 -2.55889 -0.245821 0.859649
6 -1.36768 0.118206 1.7811
6 -0.440226 0.99067 0.910531
8 -1.28143 1.49267 -0.143343
8 -2.78911 -1.6278 0.661191
6 -3.45049 1.03775 -1.15461
8 -4.35476 -0.017845 -1.47177
1 -0.112469 1.88293 1.45168
7 0.783087 0.351509 0.446012
6 0.867531 -1.02957 0.018158
7 2.04402 -1.52946 -0.347306
6 3.1889 -0.774596 -0.410361
6 3.09765 0.670322 -0.084342
6 1.9094 1.14982 0.329552
8 4.28921 -1.25392 -0.732272
8 -0.199607 -1.69377 0.027614
6 4.3335 1.51222 -0.201031
1 4.72603 1.48898 -1.22463

1 4.1451 2.55614 0.078135
1 5.13474 1.11563 0.433502
1 -0.875114 -0.777752 2.15392
1 -1.7094 0.715405 2.63376
1 -3.18163 1.55655 -2.0838
1 -3.92789 1.77741 -0.489096
1 -1.75096 -0.271359 -1.13554
1 -3.49211 0.158721 1.27241
1 1.76739 2.19373 0.602234
1 -1.90406 -1.94883 0.368835
1 -4.10154 -0.785931 -0.932665

Conf 9
6 2.35577 0.062355 0.660537
6 2.54499 -0.719098 -0.67575
6 1.42664 -0.151672 -1.58775
6 0.582229 0.784561 -0.700377
8 1.41629 1.109 0.410782
8 2.51478 -2.11312 -0.502069
6 3.62641 0.666344 1.24848
8 4.33785 1.54896 0.38456
1 0.397597 1.73614 -1.20978
7 -0.744462 0.299011 -0.339163
6 -0.980894 -1.02919 0.170547
7 -2.22079 -1.41239 0.461711
6 -3.30072 -0.570753 0.355265
6 -3.06365 0.827627 -0.081528
6 -1.80542 1.18418 -0.405839
8 -4.46073 -0.93527 0.613655
8 0.030929 -1.7628 0.307306
6 -4.23389 1.76271 -0.158892
1 -4.71568 1.86806 0.820653
1 -3.93934 2.7585 -0.51251
1 -5.00563 1.36406 -0.82782
1 0.841092 -0.962627 -2.01944
1 1.85707 0.43812 -2.4052
1 4.26766 -0.165172 1.58647
1 3.36575 1.26455 2.12731
1 1.94279 -0.647927 1.38767
1 3.52784 -0.501482 -1.1215
1 -1.55375 2.18736 -0.745053
1 1.59297 -2.27014 -0.188939
1 4.55675 1.04769 -0.411742

Compound 15: cytidine

Conf 100
6 2.53699 0.842406 0.591609
6 2.77508 -0.403829 -0.273531
6 1.3501 -0.862655 -0.626298
6 0.562063 -0.43366 0.635058
8 1.22211 0.6843 1.17796
8 3.40901 -1.39011 0.517031
6 2.60974 2.17456 -0.137094
8 1.67232 2.16645 -1.20857
1 0.535088 -1.26257 1.35137
7 -0.847939 -0.079508 0.353912

6 -1.72806 -1.16146 0.066877
7 -3.04949 -0.888728 -0.076061
6 -3.47511 0.366247 -0.04284
6 -2.60524 1.4848 0.15298
6 -1.28858 1.20279 0.361095
7 -4.80701 0.565239 -0.234341
8 -1.24405 -2.29769 -0.031168
8 1.34437 -2.2522 -0.848205
1 3.64017 2.31617 -0.497705
1 2.38908 2.98018 0.579184
1 3.26919 0.850683 1.40671
1 3.36412 -0.169659 -1.17182
1 0.977113 -0.296963 -1.48946
1 -2.96511 2.5058 0.150471
1 -0.53072 1.95323 0.538307
1 -5.39322 -0.255519 -0.24569
1 -5.22906 1.45395 -0.023685
1 3.01568 -2.23068 0.220381
1 1.77492 2.9836 -1.71149
1 0.431708 -2.54849 -0.634629

Mol 163
6 -2.5867 0.756971 -0.621977
6 -2.75503 -0.53948 0.183771
6 -1.3173 -0.890236 0.616994
6 -0.510251 -0.344794 -0.58569
8 -1.17146 0.84365 -0.963121
8 -3.24414 -1.55018 -0.676798
6 -2.96551 2.03654 0.110727
8 -2.29372 2.17732 1.35393
1 -0.513189 -1.08715 -1.39391
7 0.904718 -0.009412 -0.304442
6 1.79004 -1.09626 -0.034898
7 3.1084 -0.825455 0.127024
6 3.55167 0.421091 0.048284
6 2.69628 1.53804 -0.216799
6 1.37659 1.26195 -0.40217
7 4.88425 0.608267 0.210608
8 1.31321 -2.23594 0.038627
8 -1.22566 -2.27455 0.819939
1 -4.03683 2.0166 0.334069
1 -2.77759 2.89776 -0.548244
1 -3.16246 0.674311 -1.55099
1 -3.41233 -0.395082 1.05187
1 -1.04473 -0.311728 1.51221
1 3.07081 2.55044 -0.298173
1 0.642085 2.01571 -0.649992
1 5.44425 -0.193267 0.456035
1 5.28502 1.52824 0.273316
1 -2.83466 -2.36991 -0.34541
1 -1.35008 2.2333 1.15243
1 -0.296379 -2.51835 0.60441

Mol 7
6 -2.48277 0.841514 -0.232044
6 -2.75447 -0.521337 0.409243

6 -1.32992 -1.07931 0.595455
6 -0.583279 -0.47955 -0.62533
8 -1.2919 0.66505 -1.02891
8 -3.47074 -1.3313 -0.497113
6 -2.29064 1.97396 0.783314
8 -1.70697 3.13572 0.214473
1 -0.548872 -1.21584 -1.43543
7 0.820871 -0.110906 -0.324501
6 1.71833 -1.19193 -0.098851
7 3.03333 -0.906703 0.070842
6 3.44056 0.355525 0.086525
6 2.5569 1.46877 -0.080425
6 1.24375 1.18015 -0.299953
7 4.76627 0.566592 0.297123
8 1.2518 -2.3402 -0.073631
8 -1.37017 -2.48174 0.598733
1 -1.60755 1.66524 1.58171
1 -3.26352 2.19235 1.25207
1 -3.2882 1.09567 -0.930864
1 -3.28524 -0.426859 1.36938
1 -0.881972 -0.681269 1.51879
1 2.90402 2.49361 -0.046765
1 0.477837 1.9318 -0.45556
1 5.36654 -0.243781 0.296644
1 5.176 1.47261 0.14477
1 -3.11441 -2.22795 -0.368206
1 -2.2344 3.39217 -0.554447
1 -0.450537 -2.75753 0.387632

Mol 11
6 -2.52182 0.489135 -0.598706
6 -2.51245 -0.520967 0.570064
6 -1.08678 -1.14488 0.497702
6 -0.378145 -0.291941 -0.593223
8 -1.13899 0.887218 -0.749687
8 -3.54703 -1.46825 0.478925
6 -3.34959 1.74446 -0.378
8 -2.94771 2.45982 0.779159
1 -0.370478 -0.878103 -1.52321
7 1.01284 0.063228 -0.284745
6 1.92788 -1.0306 -0.193611
7 3.23477 -0.748813 0.036871
6 3.63327 0.507915 0.173072
6 2.74391 1.62811 0.086977
6 1.43423 1.34736 -0.153876
7 4.95805 0.710112 0.383093
8 1.47916 -2.17457 -0.326143
8 -1.20372 -2.49205 0.111703
1 -4.39178 1.44816 -0.225792
1 -3.30435 2.37701 -1.27778
1 -2.8601 -0.028759 -1.50883
1 -2.62816 0.037934 1.5042
1 -0.556869 -1.04335 1.45399
1 3.08675 2.64985 0.187966
1 0.671845 2.10689 -0.262242
1 5.53809 -0.103581 0.517413

1 5.3186 1.61553 0.631102
1 -3.1293 -2.28393 0.153386
1 -2.00293 2.63619 0.673435
1 -0.287683 -2.77437 -0.084217

Mol 1
6 -2.61633 0.6142 -0.283672
6 -2.68733 -0.771378 0.362911
6 -1.21091 -1.09595 0.667338
6 -0.498437 -0.396091 -0.513543
8 -1.25207 0.764872 -0.785648
8 -3.18534 -1.69788 -0.57925
6 -2.89116 1.7878 0.647861
8 -2.7009 3.02814 -0.009203
1 -0.473516 -1.07048 -1.37754
7 0.902084 -0.007296 -0.236801
6 1.85842 -1.06515 -0.182512
7 3.16734 -0.739538 -0.055858
6 3.52969 0.527823 0.086956
6 2.59379 1.61149 0.102426
6 1.28493 1.28621 -0.080247
7 4.85465 0.771695 0.238829
8 1.44357 -2.22952 -0.259121
8 -1.04005 -2.48703 0.713731
1 -2.25563 1.70432 1.54596
1 -3.93527 1.7568 0.97658
1 -3.28848 0.657746 -1.14601
1 -3.29805 -0.763731 1.27894
1 -0.897017 -0.606397 1.60313
1 2.89996 2.64146 0.233335
1 0.490642 2.01839 -0.11868
1 5.48702 -0.007515 0.140886
1 5.22236 1.70753 0.223014
1 -2.72495 -2.53079 -0.370719
1 -1.8706 2.95463 -0.499944
1 -0.122681 -2.65832 0.398681

Mol 10
6 -2.47988 0.835305 -0.238406
6 -2.75112 -0.532809 0.392666
6 -1.32774 -1.09055 0.586788
6 -0.577436 -0.484036 -0.628376
8 -1.2826 0.66183 -1.02785
8 -3.45864 -1.33793 -0.524685
6 -2.29591 1.94814 0.790153
8 -1.8547 3.11342 0.108064
1 -0.539103 -1.2171 -1.44175
7 0.827426 -0.112235 -0.322938
6 1.72557 -1.18608 -0.068735
7 3.0393 -0.893694 0.105735
6 3.44644 0.367829 0.082996
6 2.56483 1.47441 -0.131105
6 1.2526 1.17742 -0.346631
7 4.7708 0.586569 0.302236
8 1.26397 -2.33513 -0.023767
8 -1.36722 -2.49294 0.586246

1 -1.57071 1.63841 1.56008
1 -3.26201 2.11084 1.29312
1 -3.27603 1.10988 -0.935467
1 -3.28951 -0.447641 1.34962
1 -0.886031 -0.694743 1.51416
1 2.91386 2.4992 -0.138823
1 0.486959 1.91998 -0.541186
1 5.36936 -0.225247 0.318914
1 5.18311 1.48155 0.098327
1 -3.10472 -2.23541 -0.396626
1 -1.79493 3.83047 0.751769
1 -0.443458 -2.76644 0.392497

Mol 175
6 2.37375 -0.612826 -0.423866
6 2.2731 0.399395 0.73426
6 0.932476 1.11576 0.446346
6 0.248144 0.151041 -0.569415
8 1.01247 -1.03951 -0.599114
8 3.3858 1.25776 0.847926
6 3.29601 -1.78902 -0.144411
8 4.64086 -1.34655 -0.094641
1 0.257456 0.643681 -1.55112
7 -1.14362 -0.162692 -0.246426
6 -2.04165 0.946812 -0.307223
7 -3.35861 0.707153 -0.083702
6 -3.77064 -0.518402 0.210041
6 -2.89187 -1.64719 0.30063
6 -1.57379 -1.41456 0.052233
7 -5.09748 -0.675201 0.450936
8 -1.56382 2.05793 -0.561034
8 1.21395 2.36529 -0.13938
1 3.22577 -2.53032 -0.94689
1 2.98287 -2.28068 0.792413
1 2.72366 -0.091332 -1.3282
1 2.19295 -0.161774 1.67329
1 0.321086 1.22442 1.3501
1 -3.24767 -2.6393 0.547185
1 -0.810628 -2.18196 0.071881
1 -5.69586 0.118339 0.280334
1 -5.5102 -1.58955 0.523967
1 3.16839 2.05087 0.329069
1 4.66134 -0.507521 0.392488
1 0.34179 2.71486 -0.401804

Mol 106
6 2.46666 0.68689 0.508298
6 2.6095 -0.659457 -0.201207
6 1.15852 -0.994678 -0.596246
6 0.380513 -0.398221 0.602458
8 1.10863 0.734989 1.0187
8 3.06855 -1.62292 0.726336
6 2.68297 1.90458 -0.3867
8 4.06334 1.94586 -0.710853
1 0.312493 -1.14551 1.40189
7 -1.00677 0.023547 0.288742

6 -1.94997 -1.01036 0.016267
7 -3.24907 -0.663139 -0.160821
6 -3.6115 0.611021 -0.120035
6 -2.69021 1.67837 0.123858
6 -1.39305 1.32455 0.338782
7 -4.92367 0.881378 -0.342095
8 -1.54032 -2.1774 -0.040487
8 1.02835 -2.38306 -0.747079
1 2.36021 2.80089 0.164604
1 2.0521 1.82274 -1.28676
1 3.15652 0.733692 1.35587
1 3.27712 -0.583194 -1.06992
1 0.869008 -0.449683 -1.50894
1 -2.99874 2.71555 0.15638
1 -0.609436 2.0335 0.568419
1 -5.55289 0.095162 -0.397757
1 -5.30388 1.79454 -0.159046
1 2.64584 -2.45415 0.446642
1 4.21493 2.69052 -1.30544
1 0.084445 -2.58247 -0.55252

Mol 174
6 -2.47601 -0.673846 0.497794
6 -2.59165 0.680537 -0.209432
6 -1.13566 0.995807 -0.60525
6 -0.367376 0.370193 0.582937
8 -1.10089 -0.773033 0.955352
8 -3.05008 1.65411 0.702237
6 -2.77881 -1.89041 -0.382339
8 -4.16727 -2.04783 -0.61669
1 -0.307412 1.09704 1.40226
7 1.02023 -0.043059 0.27088
6 1.96824 0.998372 0.049733
7 3.26913 0.656907 -0.116553
6 3.62947 -0.619103 -0.119152
6 2.70204 -1.69376 0.063638
6 1.40228 -1.347 0.271996
7 4.94468 -0.882471 -0.323295
8 1.55827 2.16722 0.025628
8 -0.984905 2.38274 -0.742611
1 -2.45115 -2.79007 0.146485
1 -2.20424 -1.83255 -1.3211
1 -3.13519 -0.689882 1.371
1 -3.24666 0.626865 -1.09543
1 -0.859021 0.453291 -1.52342
1 3.00824 -2.73208 0.057457
1 0.613816 -2.06336 0.457795
1 5.57666 -0.097094 -0.348667
1 5.31936 -1.80629 -0.189991
1 -2.59938 2.47484 0.432084
1 -4.46541 -1.32135 -1.17942
1 -0.043995 2.57059 -0.517163

Mol 107
6 -2.4731 0.434188 -0.936665
6 -2.75767 -0.501637 0.246165

6 -1.3557 -0.732805 0.843337
6 -0.499714 -0.731279 -0.432533
8 -1.0806 0.209355 -1.29943
8 -3.26551 -1.73247 -0.248114
6 -2.67805 1.91485 -0.626703
8 -2.13274 2.30813 0.612965
1 -0.548482 -1.74418 -0.857237
7 0.92842 -0.444531 -0.260804
6 1.37515 0.77958 0.344723
7 2.71798 0.985244 0.438861
6 3.57737 0.092918 -0.02076
6 3.17356 -1.13154 -0.647208
6 1.8356 -1.33945 -0.743967
7 4.89729 0.367349 0.140331
8 0.547062 1.58457 0.766868
8 -1.23918 -2.00235 1.48475
1 -3.7564 2.11186 -0.580716
1 -2.27195 2.49979 -1.46686
1 -3.09387 0.138804 -1.7911
1 -3.44398 -0.041564 0.969282
1 -1.07034 0.090592 1.49722
1 3.88316 -1.85553 -1.0266
1 1.42168 -2.22858 -1.2067
1 5.13797 1.28432 0.484603
1 5.60232 -0.174314 -0.330344
1 -2.96306 -2.40702 0.382173
1 -1.16369 2.19285 0.569396
1 -1.35628 -1.87028 2.43351

Mol 46
6 -2.53808 0.840372 -0.590906
6 -2.77394 -0.405365 0.275577
6 -1.34807 -0.862372 0.627213
6 -0.561943 -0.433728 -0.635414
8 -1.22288 0.684388 -1.17695
8 -3.40769 -1.39311 -0.513204
6 -2.61347 2.17312 0.136346
8 -1.67704 2.16795 1.20882
1 -0.536235 -1.26265 -1.35175
7 0.848539 -0.079448 -0.357154
6 1.72916 -1.16111 -0.070424
7 3.04933 -0.887493 0.080046
6 3.47583 0.367235 0.038419
6 2.60552 1.48547 -0.157864
6 1.28872 1.20297 -0.364222
7 4.81467 0.561717 0.171743
8 1.24507 -2.29729 0.029346
8 -1.34052 -2.25157 0.850873
1 -3.64444 2.31345 0.495913
1 -2.39346 2.97832 -0.580583
1 -3.27038 0.846417 -1.40598
1 -3.36219 -0.170933 1.17435
1 -0.974823 -0.295122 1.48933
1 2.96709 2.5058 -0.171196
1 0.530623 1.95316 -0.541532
1 5.37176 -0.240072 0.424175

1 5.19041 1.48098 0.332002
1 -3.01287 -2.23291 -0.216339
1 -1.78296 2.98496 1.71126
1 -0.428199 -2.54733 0.634497

Mol 180
6 2.58618 -0.757615 -0.622806
6 2.75545 0.539228 0.181973
6 1.3182 0.890564 0.616369
6 0.509986 0.344774 -0.585403
8 1.17076 -0.84357 -0.963658
8 3.24385 1.54929 -0.679841
6 2.9647 -2.03685 0.110633
8 2.29464 -2.17598 1.35488
1 0.511852 1.08718 -1.39359
7 -0.904774 0.00925 -0.302451
6 -1.78988 1.09604 -0.032426
7 -3.10914 0.825766 0.124355
6 -3.55155 -0.421218 0.050749
6 -2.69632 -1.53813 -0.214359
6 -1.37672 -1.26205 -0.400689
7 -4.87898 -0.613015 0.251184
8 -1.31331 2.23579 0.040479
8 1.22721 2.27503 0.81846
1 4.03636 -2.01752 0.332473
1 2.77537 -2.89852 -0.547378
1 3.16162 -0.675916 -1.5521
1 3.4136 0.395361 1.04951
1 1.04625 0.312678 1.51214
1 -3.06948 -2.55177 -0.285977
1 -0.642199 -2.01573 -0.64871
1 -5.45943 0.206273 0.343719
1 -5.31249 -1.50404 0.079484
1 2.83509 2.36934 -0.348343
1 1.3507 -2.23315 1.15519
1 0.297479 2.51886 0.605186

Mol 128
6 -2.53789 0.84028 -0.591923
6 -2.77419 -0.405612 0.274321
6 -1.34844 -0.862133 0.626973
6 -0.561459 -0.433407 -0.634952
8 -1.22232 0.684668 -1.17703
8 -3.40704 -1.39357 -0.514954
6 -2.61412 2.17286 0.13577
8 -1.67859 2.16734 1.20898
1 -0.53538 -1.26226 -1.35135
7 0.848526 -0.07916 -0.355351
6 1.72933 -1.16103 -0.068963
7 3.04957 -0.888258 0.078525
6 3.47599 0.367152 0.040527
6 2.60538 1.48588 -0.155296
6 1.2889 1.2033 -0.362103
7 4.80873 0.562857 0.198765
8 1.24479 -2.29734 0.029272
8 -1.34048 -2.25144 0.85058

1 -3.64543 2.31283 0.494532
1 -2.39365 2.97839 -0.58068
1 -3.26959 0.846518 -1.40751
1 -3.36324 -0.171295 1.17258
1 -0.975875 -0.294983 1.4894
1 2.96601 2.50663 -0.161609
1 0.530864 1.95367 -0.53893
1 5.39242 -0.245002 0.346329
1 5.21692 1.48104 0.196324
1 -3.01164 -2.23313 -0.218056
1 -1.78319 2.98526 1.71021
1 -0.427907 -2.54673 0.634295

Compound 7: 2' Me azide Me guanosine

Mol 4
6 -2.33508 -1.13618 0.338924
6 -1.19845 -0.315436 0.332018
7 -1.1465 0.959002 -0.078511
6 -2.33333 1.42168 -0.509233
7 -3.50251 0.738209 -0.551847
6 -3.5144 -0.522698 -0.134331
7 -2.04081 -2.39561 0.834901
6 -0.768383 -2.33247 1.11511
7 -0.179347 -1.10178 0.82943
6 1.22642 -0.77707 1.04659
8 1.32036 0.489016 1.66533
6 2.27698 1.33254 0.988992
6 2.32259 0.778438 -0.440135
6 2.1304 -0.740103 -0.231563
6 1.58143 -1.47014 -1.45438
8 3.53322 1.04866 -1.12093
6 1.80633 2.7787 1.10759
8 0.662094 3.05153 0.325424
7 -2.362 2.70288 -0.976915
8 -4.64618 -1.2291 -0.156375
6 -5.82554 -0.57494 -0.645885
7 3.40546 -1.35465 0.245382
7 4.30529 -1.51176 -0.589803
7 5.20986 -1.74611 -1.24319
1 2.28498 -1.36966 -2.28646
1 0.626241 -1.0422 -1.76654
1 1.43769 -2.5345 -1.24885
1 -6.61331 -1.32539 -0.583074
1 -5.68891 -0.247404 -1.67946
1 -6.07357 0.29315 -0.029995
1 2.61428 3.4361 0.762166
1 1.63902 2.9983 2.17373
1 1.61032 -1.56133 1.70865
1 3.26687 1.22077 1.45243
1 1.45892 1.165 -0.991329
1 -0.179888 -3.13659 1.53753
1 -1.57425 3.29874 -0.765607
1 -3.26826 3.12806 -1.08575
1 3.40634 1.83313 -1.66716
1 0.057755 2.27757 0.393884

Conf 65
6 2.34487 -1.12782 -0.346566
6 1.20056 -0.318604 -0.329018
7 1.13307 0.949095 0.097643
6 2.31464 1.41958 0.534228
7 3.49136 0.748377 0.568024
6 3.51741 -0.507058 0.134354
7 2.06153 -2.38379 -0.858261
6 0.788255 -2.32915 -1.13689
7 0.188344 -1.1074 -0.83515
6 -1.21909 -0.78786 -1.04331
8 -1.32167 0.474653 -1.66799
6 -2.28844 1.31502 -1.0045
6 -2.34572 0.777431 0.426098
6 -2.11557 -0.747462 0.239662
6 -1.53957 -1.44642 1.46902
8 -3.58362 1.095 1.03316
6 -1.8288 2.76435 -1.12645
8 -0.670536 3.04203 -0.366606
7 2.32757 2.69521 1.0174
8 4.65731 -1.20064 0.147244
6 5.82943 -0.538862 0.643865
7 -3.37754 -1.39988 -0.215669
7 -4.28389 -1.51831 0.618303
7 -5.1911 -1.72889 1.27712
1 -2.24689 -1.37471 2.3021
1 -0.602615 -0.978558 1.7796
1 -1.35337 -2.50589 1.27309
1 6.62598 -1.27902 0.57057
1 5.69017 -0.226266 1.68172
1 6.06637 0.339975 0.038975
1 -2.63493 3.41133 -0.761072
1 -1.68104 2.98684 -2.19491
1 -1.60511 -1.57684 -1.69866
1 -3.27426 1.19571 -1.4753
1 -1.5037 1.19641 0.986636
1 0.206951 -3.13326 -1.56919
1 1.53173 3.28215 0.810624
1 3.22825 3.13178 1.1279
1 -3.42359 1.35457 1.94772
1 -0.069041 2.26775 -0.435172

Conf 20
6 2.48502 -0.816701 -0.206062
6 1.27046 -0.106666 -0.2438
7 1.07298 1.15064 0.158154
6 2.19816 1.72642 0.633971
7 3.41864 1.16365 0.721937
6 3.58401 -0.090374 0.311219
7 2.31486 -2.10162 -0.712813
6 1.05324 -2.15568 -1.03612
7 0.34773 -0.986113 -0.773511
6 -1.07529 -0.792904 -1.03593
8 -1.26481 0.44917 -1.67852
6 -2.31972 1.20895 -1.04991

6 -2.36684 0.674431 0.386307
6 -2.01639 -0.821589 0.215402
6 -1.43491 -1.4678 1.47005
8 -3.62692 0.837192 1.00962
6 -1.97998 2.69086 -1.17634
8 -0.893244 3.07739 -0.360956
7 2.08019 3.00338 1.09645
8 4.8359 -0.54232 0.441121
6 5.15498 -1.88022 0.030097
7 -3.20835 -1.57079 -0.283178
7 -4.11293 -1.8006 0.529838
7 -5.00865 -2.11225 1.16276
1 -2.17035 -1.41994 2.27898
1 -0.536965 -0.939753 1.79835
1 -1.18147 -2.51714 1.29521
1 6.21977 -1.99205 0.235743
1 4.95638 -2.02447 -1.03404
1 4.57982 -2.61523 0.59735
1 -2.85616 3.27514 -0.868471
1 -1.79599 2.9109 -2.2396
1 -1.36597 -1.61879 -1.69506
1 -3.27712 0.998291 -1.54587
1 -1.56994 1.15444 0.96348
1 0.556868 -3.01082 -1.47587
1 1.24946 3.5225 0.851768
1 2.93665 3.50898 1.25498
1 -3.58001 1.5995 1.59794
1 -0.220343 2.3606 -0.395812

Conf 127
6 2.4934 -0.806485 -0.209362
6 1.27118 -0.110124 -0.238616
7 1.05674 1.13914 0.177375
6 2.17431 1.72359 0.660492
7 3.40169 1.17477 0.742554
6 3.58302 -0.072224 0.316822
7 2.33641 -2.08777 -0.730299
6 1.07498 -2.15217 -1.05298
7 0.357251 -0.993075 -0.776522
6 -1.06729 -0.807218 -1.03156
8 -1.26601 0.428899 -1.68302
6 -2.32821 1.18604 -1.06608
6 -2.38342 0.669277 0.37234
6 -2.00202 -0.829677 0.224478
6 -1.39976 -1.44618 1.48498
8 -3.66709 0.875487 0.930253
6 -2.00091 2.66975 -1.2014
8 -0.898983 3.06597 -0.41117
7 2.03832 2.99346 1.13701
8 4.84056 -0.509834 0.441707
6 5.17622 -1.83792 0.012512
7 -3.18035 -1.6103 -0.25284
7 -4.09665 -1.79924 0.557063
7 -4.99933 -2.08399 1.19375
1 -2.13628 -1.42595 2.29519
1 -0.522136 -0.884018 1.81191

1 -1.10844 -2.48694 1.31869
1 6.24234 -1.93943 0.216583
1 4.97937 -1.97002 -1.05356
1 4.61033 -2.58782 0.569563
1 -2.87505 3.2447 -0.873921
1 -1.83908 2.88799 -2.26868
1 -1.35851 -1.63961 -1.6823
1 -3.2822 0.966469 -1.56537
1 -1.60584 1.17619 0.952946
1 0.587425 -3.00774 -1.50171
1 1.2001 3.50184 0.894621
1 2.88718 3.51075 1.29893
1 -3.56572 1.17007 1.84261
1 -0.228783 2.34852 -0.444059

Conf 5
6 -2.16001 -1.0772 -0.013549
6 -1.31837 0.000805 -0.320497
7 -1.66113 1.283 -0.490377
6 -2.98381 1.46478 -0.353954
7 -3.91499 0.526216 -0.048538
6 -3.5189 -0.73075 0.121995
7 -1.43869 -2.25646 0.092365
6 -0.20093 -1.90431 -0.142783
7 -0.053384 -0.539438 -0.395678
6 1.15486 0.205984 -0.722025
8 2.06141 -0.650043 -1.38767
6 3.29534 -0.786513 -0.662721
6 2.97711 -0.310159 0.762298
6 1.91158 0.781574 0.517942
6 1.06258 1.11571 1.73881
8 4.09254 0.100869 1.51182
6 3.79498 -2.22272 -0.780799
8 2.96782 -3.17031 -0.112938
7 -3.46519 2.72853 -0.570503
8 -4.40784 -1.68142 0.430066
6 -5.77999 -1.28636 0.550837
7 2.72189 1.96407 0.078312
7 2.1026 3.00887 -0.153305
7 1.64473 4.02587 -0.392388
1 1.7166 1.45817 2.54517
1 0.523649 0.230243 2.08288
1 0.326577 1.89208 1.51284
1 -6.32099 -2.20307 0.786056
1 -6.1467 -0.854155 -0.383707
1 -5.90735 -0.552201 1.3509
1 4.82902 -2.26012 -0.413211
1 3.80272 -2.50197 -1.83876
1 0.83641 1.01927 -1.38339
1 4.05184 -0.120127 -1.1045
1 2.50451 -1.1265 1.31827
1 0.654729 -2.56542 -0.175506
1 -2.79053 3.47697 -0.564426
1 -4.39042 2.92486 -0.224211
1 4.36186 0.961758 1.15302
1 3.27907 -3.26231 0.795848

Conf 106
6 2.32155 -1.11427 -0.392065
6 1.21836 -0.254004 -0.305003
7 1.23422 1.00585 0.152144
6 2.45449 1.40784 0.544289
7 3.60093 0.687204 0.496216
6 3.54514 -0.557639 0.03627
7 1.958 -2.34617 -0.91224
6 0.678118 -2.22903 -1.13392
7 0.14967 -0.988396 -0.781372
6 -1.22463 -0.561499 -0.997946
8 -1.18934 0.731062 -1.54249
6 -2.31321 1.52437 -1.12594
6 -3.05561 0.747189 -0.018689
6 -2.1566 -0.522488 0.256933
6 -1.43388 -0.394103 1.6
8 -4.35478 0.45014 -0.499227
6 -1.7641 2.88582 -0.700512
8 -0.916336 2.79499 0.42409
7 2.54726 2.66214 1.07023
8 4.65113 -1.30202 -0.029048
6 5.87798 -0.704842 0.413173
7 -2.91047 -1.80345 0.195105
7 -3.82962 -1.94071 1.00365
7 -4.70565 -2.18112 1.6994
1 -2.16752 -0.427747 2.41287
1 -0.90833 0.559673 1.66333
1 -0.723711 -1.21082 1.74574
1 6.63485 -1.4773 0.278058
1 5.81253 -0.411489 1.46389
1 6.12016 0.177089 -0.185256
1 -2.60182 3.54318 -0.438321
1 -1.25212 3.33175 -1.56763
1 -1.65898 -1.28446 -1.70174
1 -3.00608 1.64206 -1.96925
1 -3.10365 1.3476 0.896673
1 0.039008 -2.99795 -1.54748
1 1.75684 3.27968 0.963049
1 3.46657 3.05702 1.17773
1 -4.93986 0.285248 0.251116
1 -0.189419 2.16168 0.209079

Compound 9: 2' diF cytosine

Conf 1
6 -2.54381 0.512372 -0.566876
6 -2.64308 -0.738059 0.323601
6 -1.18557 -0.9387 0.748551
6 -0.390689 -0.451944 -0.471914
8 -1.13727 0.682116 -0.902227
8 -3.07453 -1.8206 -0.473597
6 -3.00172 1.80675 0.095338
8 -2.75666 2.92292 -0.735529
1 -0.368103 -1.25051 -1.21826
7 0.98355 -0.085206 -0.226709
6 2.01712 -0.940039 -0.786975

7 3.31034 -0.562889 -0.567951
6 3.59092 0.525138 0.124508
6 2.59105 1.39752 0.678431
6 1.29863 1.04545 0.468752
7 4.90411 0.808572 0.321442
8 1.67583 -1.93254 -1.41083
9 -0.91252 -2.22908 1.08298
1 -2.50456 1.90424 1.07449
1 -4.08214 1.76681 0.270726
1 -3.10126 0.337755 -1.49288
1 -3.28152 -0.583116 1.20529
9 -0.917199 -0.1608 1.84762
1 2.84537 2.29355 1.2301
1 0.463375 1.63493 0.821606
1 5.57828 0.236337 -0.163464
1 5.19702 1.6938 0.698533
1 -2.89252 -2.63805 0.01263
1 -1.82859 2.86384 -1.00475

Conf 100
6 -2.42712 0.505908 -0.94658
6 -2.68398 -0.487383 0.199693
6 -1.2663 -0.898023 0.635338
6 -0.418709 -0.698815 -0.641876
8 -1.05523 0.294661 -1.38806
8 -3.36678 -1.60573 -0.340603
6 -2.61945 1.97165 -0.56354
8 -2.06253 2.29945 0.683221
1 -0.443126 -1.66272 -1.17063
7 0.996302 -0.396015 -0.421819
6 1.44235 0.886952 0.081033
7 2.78477 1.04731 0.264331
6 3.63917 0.080937 -0.009299
6 3.23708 -1.19884 -0.521281
6 1.90676 -1.37347 -0.710343
7 4.95579 0.342694 0.187946
8 0.631416 1.77029 0.319562
9 -1.22312 -2.21804 1.02663
1 -3.69797 2.16538 -0.500862
1 -2.22091 2.58851 -1.38427
1 -3.08481 0.24456 -1.78381
1 -3.21274 -0.022134 1.03942
9 -0.818917 -0.171206 1.68507
1 3.9452 -1.98102 -0.762801
1 1.50007 -2.30005 -1.10115
1 5.19305 1.21839 0.628385
1 5.64653 -0.387346 0.149172
1 -3.34483 -2.3084 0.324263
1 -1.09387 2.18724 0.611889

Conf 13
6 -2.31946 0.498818 -0.831191
6 -2.2719 -0.187783 0.539188
6 -1.20576 -1.26988 0.323305
6 -0.324158 -0.761982 -0.851603
8 -1.00186 0.327791 -1.41464

8 -3.55208 -0.689183 0.852269
6 -2.64083 1.9899 -0.795865
8 -1.98087 2.70155 0.223313
1 -0.247362 -1.58796 -1.57039
7 1.0515 -0.409199 -0.502284
6 1.31267 0.672064 0.407693
7 2.61158 0.963204 0.685499
6 3.59453 0.267147 0.140406
6 3.37505 -0.820501 -0.771154
6 2.08111 -1.10904 -1.05812
7 4.85834 0.624994 0.468618
8 0.358853 1.27499 0.893095
9 -1.79601 -2.43769 -0.084327
1 -3.71731 2.09912 -0.620754
1 -2.42521 2.39613 -1.79794
1 -3.05718 -0.023702 -1.45677
1 -1.89428 0.51401 1.28638
9 -0.482275 -1.56395 1.43903
1 4.18707 -1.37883 -1.21889
1 1.80392 -1.90543 -1.74068
1 4.98101 1.38029 1.12475
1 5.66117 0.126457 0.126155
1 -3.58423 -0.859212 1.80207
1 -1.05281 2.40728 0.262576

Conf 181
6 -2.31439 0.522308 -0.821345
6 -2.27317 -0.167241 0.558859
6 -1.21211 -1.24547 0.335948
6 -0.329097 -0.753415 -0.842044
8 -0.997908 0.341039 -1.40716
8 -3.51408 -0.667155 0.992205
6 -2.62433 2.01567 -0.787997
8 -1.94721 2.72528 0.219575
1 -0.260679 -1.58242 -1.55858
7 1.04919 -0.415154 -0.494473
6 1.31891 0.664178 0.416313
7 2.62006 0.950856 0.686814
6 3.59742 0.252642 0.13489
6 3.36931 -0.833627 -0.777166
6 2.07345 -1.1177 -1.05817
7 4.86451 0.609851 0.451
8 0.36808 1.26602 0.907568
9 -1.85787 -2.39456 -0.106009
1 -3.69801 2.13277 -0.599724
1 -2.41872 2.4172 -1.79441
1 -3.05451 0.011733 -1.45694
1 -1.91135 0.535501 1.30825
9 -0.498368 -1.59892 1.42815
1 4.17739 -1.39262 -1.23111
1 1.79019 -1.91166 -1.74113
1 4.99123 1.34593 1.12821
1 5.6606 0.08241 0.136675
1 -3.68882 -1.4773 0.491664
1 -1.02457 2.41541 0.261189

Conf 7
6 -2.44879 0.505314 -0.670797
6 -2.25107 -0.128258 0.705938
6 -1.198 -1.19589 0.388192
6 -0.363707 -0.603564 -0.779517
8 -1.14382 0.476779 -1.28286
8 -3.47851 -0.616298 1.18518
6 -2.92871 1.95094 -0.660296
8 -1.97776 2.8241 -0.065788
1 -0.195724 -1.37739 -1.53084
7 0.953852 -0.131945 -0.387319
6 2.08526 -0.974298 -0.71564
7 3.31868 -0.525497 -0.335103
6 3.45074 0.606735 0.328482
6 2.34356 1.44795 0.695585
6 1.11285 1.02737 0.309236
7 4.71474 0.993171 0.64695
8 1.87729 -2.02381 -1.305
9 -1.8123 -2.33629 -0.038134
1 -3.84419 2.01099 -0.06419
1 -3.16873 2.265 -1.68519
1 -3.15477 -0.11992 -1.23582
1 -1.79895 0.606613 1.38748
9 -0.435807 -1.52016 1.47158
1 2.47519 2.37773 1.23432
1 0.211746 1.59584 0.50873
1 5.45256 0.327178 0.474819
1 4.87784 1.73223 1.30985
1 -3.332 -1.02599 2.04823
1 -1.23126 2.86161 -0.679418

Conf 77
6 -2.31949 0.498542 -0.831226
6 -2.27181 -0.187954 0.539251
6 -1.20564 -1.26989 0.323422
6 -0.324072 -0.761897 -0.851587
8 -1.00186 0.32787 -1.4145
8 -3.55196 -0.68934 0.852482
6 -2.64125 1.98952 -0.795896
8 -1.98161 2.70121 0.223501
1 -0.24736 -1.58791 -1.57035
7 1.05154 -0.40904 -0.502445
6 1.31268 0.672202 0.407576
7 2.61168 0.962781 0.686056
6 3.59458 0.267478 0.140082
6 3.37513 -0.819745 -0.771936
6 2.0812 -1.10847 -1.05877
7 4.85879 0.628489 0.464579
8 0.358929 1.27509 0.893027
9 -1.79574 -2.43781 -0.08422
1 -3.71775 2.09852 -0.620858
1 -2.42559 2.39593 -1.79789
1 -3.05707 -0.024225 -1.45676
1 -1.89418 0.513984 1.2863
9 -0.482118 -1.56391 1.43916
1 4.18721 -1.37686 -1.22105

1 1.80402 -1.90454 -1.74169
1 4.97971 1.36984 1.1369
1 5.6593 0.109712 0.147361
1 -3.584 -0.859423 1.80227
1 -1.05335 2.40756 0.262357

Conf 8
6 2.31316 0.523471 0.821786
6 2.27324 -0.165925 -0.558455
6 1.21335 -1.24542 -0.335828
6 0.32905 -0.754043 0.841479
8 0.996828 0.340859 1.40721
8 3.51498 -0.664582 -0.991103
6 2.62189 2.01715 0.788829
8 1.9445 2.72633 -0.218911
1 0.260716 -1.58313 1.55791
7 -1.0492 -0.416634 0.493198
6 -1.31895 0.662723 -0.41768
7 -2.62006 0.95167 -0.685535
6 -3.59743 0.251534 -0.135811
6 -3.36929 -0.835128 0.775832
6 -2.07344 -1.11905 1.05703
7 -4.86376 0.596017 -0.467162
8 -0.367937 1.26437 -0.908842
9 1.8604 -2.39329 0.107331
1 3.69553 2.13538 0.601172
1 2.41539 2.41832 1.79522
1 3.05362 0.013396 1.45741
1 1.91105 0.536237 -1.30817
9 0.500845 -1.60045 -1.4282
1 -4.17723 -1.39762 1.2257
1 -1.79023 -1.91319 1.73979
1 -4.99366 1.38249 -1.0843
1 -5.66435 0.146324 -0.058104
1 3.69011 -1.47466 -0.490573
1 1.02244 2.415 -0.261669

Compound 11: 2' MeCl inosine

Conf 1
6 -2.78559 0.683303 -0.291332
6 -2.4277 -0.156763 0.940444
6 -1.33132 -1.10148 0.39282
6 -0.619945 -0.200056 -0.659865
8 -1.53737 0.821161 -1.00713
6 -3.32634 2.07376 -0.004057
8 -3.62326 2.78461 -1.18925
7 0.606261 0.425552 -0.165918
1 -0.327401 -0.786388 -1.53413
6 0.756668 1.6138 0.530183
7 2.00507 1.89359 0.816671
6 2.72497 0.847116 0.286032

6 1.87917 -0.073039 -0.33121
6 4.15147 0.604303 0.283107
7 4.41169 -0.626905 -0.411129
6 3.48563 -1.45069 -0.978918
7 2.20031 -1.23275 -0.970111
8 5.07235 1.24745 0.749971
8 -3.50373 -0.759259 1.60251
17 -2.18887 -2.44281 -0.560318
1 -2.60966 2.61957 0.63261
1 -4.26109 1.97625 0.556973
1 -3.50374 0.143914 -0.923491
1 -1.94184 0.488199 1.68518
6 -0.461066 -1.76234 1.44571
1 -0.092363 2.23761 0.765556
1 3.87293 -2.34284 -1.46361
1 5.39383 -0.871634 -0.460388
1 -2.80521 2.82736 -1.70407
1 -3.86606 -1.43989 1.01386
1 0.277423 -2.42584 0.991419
1 -1.09215 -2.33714 2.12701
1 0.063381 -0.996946 2.02561

Conf 10
6 -2.2056 1.10863 -0.785575
6 -2.12029 0.581079 0.651019
6 -1.75393 -0.896378 0.454254
6 -0.879201 -0.834392 -0.84487
8 -1.14503 0.399621 -1.46829
6 -1.96646 2.60583 -0.940803
8 -0.910847 3.09281 -0.139151
7 0.565833 -0.974818 -0.636253
1 -1.14768 -1.66898 -1.50036
6 1.26312 -2.15029 -0.861378
7 2.54173 -2.06851 -0.595582
6 2.71594 -0.770979 -0.176981
6 1.50922 -0.068214 -0.196646
6 3.93088 -0.107202 0.24453
7 3.62576 1.25348 0.590055
6 2.40357 1.84321 0.528879
7 1.31089 1.24032 0.145955
8 5.07073 -0.518624 0.338051
8 -3.28188 0.760951 1.42551
17 -3.27445 -1.84005 0.001171
1 -2.87726 3.13421 -0.634367
1 -1.81085 2.81874 -2.01
1 -3.17509 0.837345 -1.2236
1 -1.25201 1.05656 1.12508
6 -1.13695 -1.56696 1.6702
1 0.755759 -3.03193 -1.2303
1 2.35567 2.8886 0.82057
1 4.42686 1.79386 0.895737
1 -0.147186 2.49292 -0.253749
1 -3.23515 1.64197 1.81834
1 -0.897255 -2.61485 1.47546
1 -1.84497 -1.51858 2.50023
1 -0.220222 -1.04646 1.96311

Conf 107
6 -2.31529 1.31639 -0.894603
6 -2.95624 0.358257 0.13813
6 -1.7864 -0.602371 0.52548
6 -0.921387 -0.568752 -0.772604
8 -1.0664 0.719649 -1.30708
6 -2.0053 2.74417 -0.447558
8 -1.05987 2.8161 0.596123
7 0.499235 -0.839505 -0.611037
1 -1.30178 -1.33352 -1.46201
6 1.10613 -2.0521 -0.894094
7 2.39943 -2.05263 -0.697527
6 2.67821 -0.775391 -0.270996
6 1.51979 0.001264 -0.208944
6 3.9551 -0.194366 0.084361
7 3.75714 1.17621 0.463554
6 2.57077 1.83942 0.491163
7 1.42227 1.31279 0.16732
8 5.07105 -0.676643 0.101803
8 -4.02725 -0.269819 -0.534633
17 -2.38519 -2.3244 0.771955
1 -2.92921 3.21689 -0.094878
1 -1.66946 3.30335 -1.33487
1 -2.99564 1.34934 -1.75445
1 -3.30069 0.892539 1.03374
6 -1.0916 -0.175566 1.81082
1 0.522003 -2.8945 -1.23907
1 2.6063 2.87774 0.808399
1 4.60689 1.66049 0.728756
1 -0.234478 2.38768 0.286371
1 -4.36372 -0.981194 0.029486
1 -0.206383 -0.78406 2.00496
1 -1.78166 -0.29584 2.64949
1 -0.813443 0.877784 1.74799

Conf 15
6 2.765 0.673497 0.659573
6 2.46777 0.05659 -0.714344
6 1.4254 -1.03006 -0.381329
6 0.649019 -0.370021 0.794406
8 1.53113 0.557817 1.39102
6 3.21466 2.1311 0.638738
8 2.36957 2.97675 -0.13485
7 -0.562306 0.334287 0.369554
1 0.327098 -1.1238 1.51673
6 -0.699391 1.65641 -0.03003
7 -1.93728 1.97448 -0.329642
6 -2.65781 0.821294 -0.115955
6 -1.82652 -0.208041 0.323237
6 -4.07212 0.563405 -0.274178
7 -4.34 -0.801946 0.086571
6 -3.42885 -1.72476 0.50801
7 -2.15342 -1.49221 0.643641
8 -4.98138 1.28452 -0.641241
8 3.58974 -0.340718 -1.45505

17 2.34918 -2.47107 0.343743
1 4.25274 2.16629 0.283573
1 3.19595 2.5138 1.6637
1 3.54226 0.083183 1.16758
1 1.94902 0.804304 -1.32616
6 0.615969 -1.54134 -1.55766
1 0.15233 2.32309 -0.049902
1 -3.82005 -2.71227 0.737694
1 -5.31529 -1.06438 0.005562
1 2.7129 3.01504 -1.03593
1 3.96955 -1.1162 -1.01282
1 -0.09747 -2.30613 -1.24494
1 1.29006 -1.95929 -2.30859
1 0.064641 -0.713147 -2.0128

Conf 18
6 2.69969 -0.647712 -0.650993
6 2.39196 -0.033574 0.719996
6 1.27441 0.97773 0.393039
6 0.521384 0.241655 -0.762823
8 1.41374 -0.728838 -1.28523
6 3.3536 -2.02466 -0.57981
8 4.67724 -1.91409 -0.090469
7 -0.691521 -0.447745 -0.32933
1 0.211814 0.954425 -1.53118
6 -0.82596 -1.74211 0.146465
7 -2.0623 -2.05797 0.44753
6 -2.78969 -0.925857 0.157392
6 -1.96157 0.083973 -0.33003
6 -4.20914 -0.6734 0.28107
7 -4.48335 0.665946 -0.162127
6 -3.57407 1.57163 -0.622066
7 -2.29456 1.34435 -0.726621
8 -5.11521 -1.38206 0.676751
8 3.50549 0.441419 1.43637
17 2.08258 2.46902 -0.356431
1 3.41495 -2.45983 -1.5813
1 2.7337 -2.69749 0.03672
1 3.36051 0.020943 -1.21966
1 1.93914 -0.805254 1.35722
6 0.447962 1.44346 1.57757
1 0.02342 -2.40506 0.210147
1 -3.97012 2.54084 -0.912947
1 -5.46193 0.923986 -0.110153
1 4.64715 -1.40515 0.7341
1 3.8533 1.2118 0.958995
1 -0.315693 2.16031 1.26983
1 1.10362 1.91065 2.31574
1 -0.043161 0.58602 2.04734

Conf 180
6 -2.22035 1.12365 -0.789354
6 -2.18053 0.590934 0.649342
6 -1.72459 -0.875388 0.466338
6 -0.867925 -0.807892 -0.842449
8 -1.13534 0.433928 -1.45028

6 -1.99067 2.62415 -0.924939
8 -0.893936 3.09395 -0.17478
7 0.575088 -0.958585 -0.654269
1 -1.14942 -1.63449 -1.50341
6 1.27571 -2.12324 -0.925521
7 2.55505 -2.04438 -0.663861
6 2.72641 -0.761392 -0.199109
6 1.51725 -0.064684 -0.187751
6 3.93793 -0.104264 0.243433
7 3.62306 1.24003 0.641141
6 2.39655 1.82383 0.605108
7 1.30776 1.22682 0.202491
8 5.08117 -0.510097 0.31729
8 -3.34044 0.795182 1.40697
17 -3.2307 -1.88381 0.038763
1 -2.88532 3.13751 -0.554764
1 -1.89113 2.85829 -1.99711
1 -3.17274 0.853388 -1.26998
1 -1.37722 1.11846 1.17379
6 -1.08367 -1.50395 1.68951
1 0.770075 -2.99348 -1.32289
1 2.34124 2.85725 0.935314
1 4.42109 1.7743 0.965228
1 -0.150047 2.47376 -0.297621
1 -4.04298 0.245661 1.02682
1 -0.807152 -2.54654 1.5163
1 -1.78444 -1.45751 2.52605
1 -0.185236 -0.943336 1.96356

Compound 11: 2' MeCl inosine anion

Conf 10
6 -2.16346 1.11536 -0.811493
6 -2.07108 0.619594 0.639357
6 -1.66425 -0.856881 0.480484
6 -0.854742 -0.864085 -0.856836
8 -1.20697 0.327395 -1.54082
6 -1.83736 2.59833 -0.999796
8 -0.758194 3.06413 -0.240117
7 0.575151 -0.965546 -0.709841
1 -1.15157 -1.73751 -1.44843
6 1.3191 -2.10897 -1.00131
7 2.58547 -1.99422 -0.715307
6 2.72088 -0.707542 -0.202171
6 1.49219 -0.050319 -0.194825
6 3.89723 -0.007325 0.306375
7 3.61555 1.2985 0.759358
6 2.39267 1.77797 0.693321
7 1.2463 1.205 0.238208
8 5.03598 -0.479818 0.355826
8 -3.2025 0.874602 1.43711
17 -3.24438 -1.83367 0.135758
1 -2.72696 3.1719 -0.702255
1 -1.6963 2.75959 -2.08431
1 -3.174 0.916699 -1.20698
1 -1.22895 1.13119 1.11548

6 -1.00161 -1.48408 1.68882
1 0.838662 -2.97868 -1.43377
1 2.25502 2.8007 1.05549
1 -0.02916 2.38442 -0.224699
1 -3.91617 0.315739 1.09312
1 -0.754347 -2.53408 1.51589
1 -1.66537 -1.40612 2.55341
1 -0.07767 -0.940348 1.9047

Conf 108
6 -2.14708 1.0836 -0.815494
6 -1.97905 0.606205 0.630975
6 -1.70083 -0.885753 0.47419
6 -0.87403 -0.909399 -0.85579
8 -1.22909 0.265976 -1.56595
6 -1.81423 2.56261 -1.03527
8 -0.796686 3.05852 -0.204859
7 0.559313 -0.992279 -0.688557
1 -1.15581 -1.796 -1.43413
6 1.30242 -2.14772 -0.924524
7 2.56785 -2.02572 -0.636961
6 2.70384 -0.719031 -0.180273
6 1.47734 -0.056621 -0.208244
6 3.88476 -0.006189 0.299232
7 3.61443 1.3212 0.691365
6 2.3956 1.80525 0.598982
7 1.24599 1.22289 0.166325
8 5.01902 -0.486052 0.37337
8 -3.06302 0.907788 1.48852
17 -3.30453 -1.77181 0.115114
1 -2.72131 3.14828 -0.826308
1 -1.59152 2.69129 -2.10886
1 -3.17478 0.881798 -1.15156
1 -1.04583 1.03814 1.0126
6 -1.06757 -1.5599 1.67691
1 0.821828 -3.03443 -1.32068
1 2.26517 2.84472 0.914499
1 -0.035382 2.41165 -0.20586
1 -2.90088 1.80329 1.81265
1 -0.882647 -2.62018 1.48861
1 -1.73059 -1.4613 2.53972
1 -0.113129 -1.07725 1.90443

Conf 123
6 -2.18722 1.3659 -0.928328
6 -2.87772 0.49084 0.139572
6 -1.78886 -0.563995 0.509713
6 -0.901543 -0.623408 -0.783821
8 -1.06729 0.62042 -1.42956

6 -1.69675 2.74512 -0.479026
8 -0.838396 2.72854 0.624903
7 0.501843 -0.876633 -0.592785
1 -1.2801 -1.43696 -1.41661
6 1.13924 -2.07893 -0.896842
7 2.42647 -2.05262 -0.695933
6 2.69181 -0.763317 -0.24492
6 1.51946 -0.012681 -0.178448
6 3.95685 -0.137324 0.130144
7 3.81811 1.20776 0.530026
6 2.63276 1.77774 0.539164
7 1.41484 1.27872 0.207949
8 5.05598 -0.698731 0.113095
8 -4.04092 -0.04465 -0.470292
17 -2.56446 -2.24219 0.751758
1 -2.57974 3.34928 -0.220347
1 -1.23217 3.21739 -1.36204
1 -2.91041 1.49954 -1.74487
1 -3.13555 1.07364 1.03426
6 -1.06353 -0.235828 1.80392
1 0.566051 -2.92522 -1.25543
1 2.60282 2.82282 0.861742
1 0.010497 2.23655 0.380755
1 -4.31983 -0.804741 0.061476
1 -0.238683 -0.928862 1.97529
1 -1.76373 -0.297522 2.6417
1 -0.674166 0.781431 1.74746
